# Protocol for a single-blind randomized controlled clinical trial to investigate the feasibility and safety of in-bed self-exercises based on electromyography sensor feedback in patients with subacute stroke

**DOI:** 10.1371/journal.pone.0310178

**Published:** 2024-12-30

**Authors:** Jung Hyun Kim, Byung-Mo Oh, Han Gil Seo, Sung Eun Hyun, Jong tae Han, Dae hee Kang, Woo Hyung Lee

**Affiliations:** 1 Department of Rehabilitation Medicine, Seoul National University Hospital, Seoul, Republic of Korea; 2 Biomedical Research Institute, Seoul National University Hospital, Seoul, Republic of Korea Korea; 3 Department of Rehabilitation Medicine, Seoul National University College of Medicine, Seoul, Republic of Korea; 4 Institute on Aging, Seoul National University, Seoul, Republic of Korea; Iran University of Medical Sciences, ISLAMIC REPUBLIC OF IRAN

## Abstract

**Background:**

The dosage and intensity of physical therapy are crucial factors influencing the motor recovery of the hemiplegic lower limb in patients with subacute stroke. Biofeedback using wearable sensors may provide opportunities for patients with stroke to effectively guide self-exercises with monitoring of muscular activities in hemiplegic lower limbs. This study aims to explore the feasibility and safety of in-bed self-exercises based on electromyography sensor feedback in patients with subacute stroke.

**Methods:**

This is a pilot randomized controlled trial comparing conventional physical therapy with additional in-bed self-exercises based on electromyography sensor feedback and conventional physical therapy alone. The interventions will be adjusted according to the muscle strength and Brunnstrom recovery stage in the hemiplegic lower limbs. The primary outcome measure is the Pittsburgh Rehabilitation Participation Scale. The secondary outcome measures include the number and percentage of participating sessions, number and percentage of effortful sessions, number and percentage of successful sessions, mean amplitude of muscle contractions in a session, duration and percentage of participating sessions during self-exercises, Rivermead Motor Assessment, Manual Muscle Test, Brunnstrom recovery stage, Fugl–Meyer assessment, Berg Balance Scale, Functional Ambulation Category, modified Rankin scale, and Short-Form Health Survey 36 version 2.

**Results:**

The results will be described in future studies.

**Conclusion:**

This clinical trial will estimate the feasibility and safety of in-bed self-exercises based on electromyography sensor feedback in patients with subacute stroke. If the expected results are achieved in this study, stroke rehabilitation methods will be enriched.

**Trial registration:**

clinicialtrials.gov, NCT05820815.

## 1. Background

Stroke is a major cause of disability worldwide [1]. Stroke survivors frequently experience sensorimotor deficits that result in impaired mobility. Rehabilitation training after stroke is crucial for promoting gait function and preventing falls, thereby improving muscle strength recovery. Multiple preclinical and clinical studies have provided promising evidence that high-dose rehabilitation training can enhance motor recovery after a stroke, resulting in additional clinical benefits [2–4]. This emphasizes the importance of high-dose training in improving the functional outcomes and quality of life in stroke survivors [2, 5–7].

However, despite the clear benefits of high-dose training in stroke patients, its implementation with the assistance of physical therapists remains challenging. Generally, stroke rehabilitation is covered by insurance (e.g., Medicare) only during certain hours of the day, and extension of the rehabilitation duration is limited [8], which can be primarily due to the high cost of stroke care, limited healthcare resources, and predefined amounts of rehabilitation covered by general health insurance [9–11]. In addition, access to rehabilitation services provided by therapists is restricted on weekends due to standard work schedules that are not aligned with the needs of stroke recovery [12]. Even if patients with stroke seek to independently engage in self-exercise without a therapist’s assistance, those with impaired functional mobility are at risk of falling when out of bed, thereby potentially limiting their capacity to participate in active exercise [13]. To address this challenge, home-based training for stroke rehabilitation has been proposed as a cost-effective intervention [14, 15], indicating significant improvements in motor recovery in patients with subacute stroke. However, investigations into methods for increasing the dose of rehabilitation training in patients with subacute stroke during hospitalization are limited. It is essential to develop effective strategies for implementing high-dose training in this clinical setting to optimize outcomes for stroke survivors [3, 4].

Self-exercises under intermittent supervision by physical therapists could serve as an alternative to efficiently augment the training volume for hospitalized stroke patients [16, 17]. Self-exercises are a crucial component of strategies such as telerehabilitation and self-administration to increase the dose of rehabilitation training [18, 19]. A recent systematic review showed that the effect of self-exercises on post-stroke motor function was equivalent to that of conventional rehabilitation [20]. However, although self-exercises can help compensate for the shortage of training during hospitalization [21, 22], its inherent limitations, such as its repetitive nature and lack of feedback, may restrict its effectiveness in improving motor recovery [23, 24].

Wearable electromyography (EMG) sensors can be utilized to acquire data on muscular activity, potentially enabling monitoring of the quantity and quality of self-exercises and tailoring rehabilitation programs for individual patients by adjusting the grades of self-exercises. This study aims to investigate the feasibility and safety of in-bed self-exercises based on EMG sensor feedback in patients with subacute stroke. Towards this goal, we developed a novel self-exercise protocol incorporating wearable EMG sensors to provide biofeedback to stroke patients. In this study, we hypothesize that there will be a statistically significant difference in Pittsburgh Rehabilitation Participation Scales among subacute stroke patients who receive conventional lower extremity rehabilitation compared to those who receive rehabilitation based on feedback using data obtained via electromyography-supervised self-exercise. Specifically, the null hypothesis (H₀) posits no significant difference between the two groups, while the alternative hypothesis (H₁) posits a significant difference in the participation scales.

## 2. Methods

### 2.1. Study design

**Fig 1:**
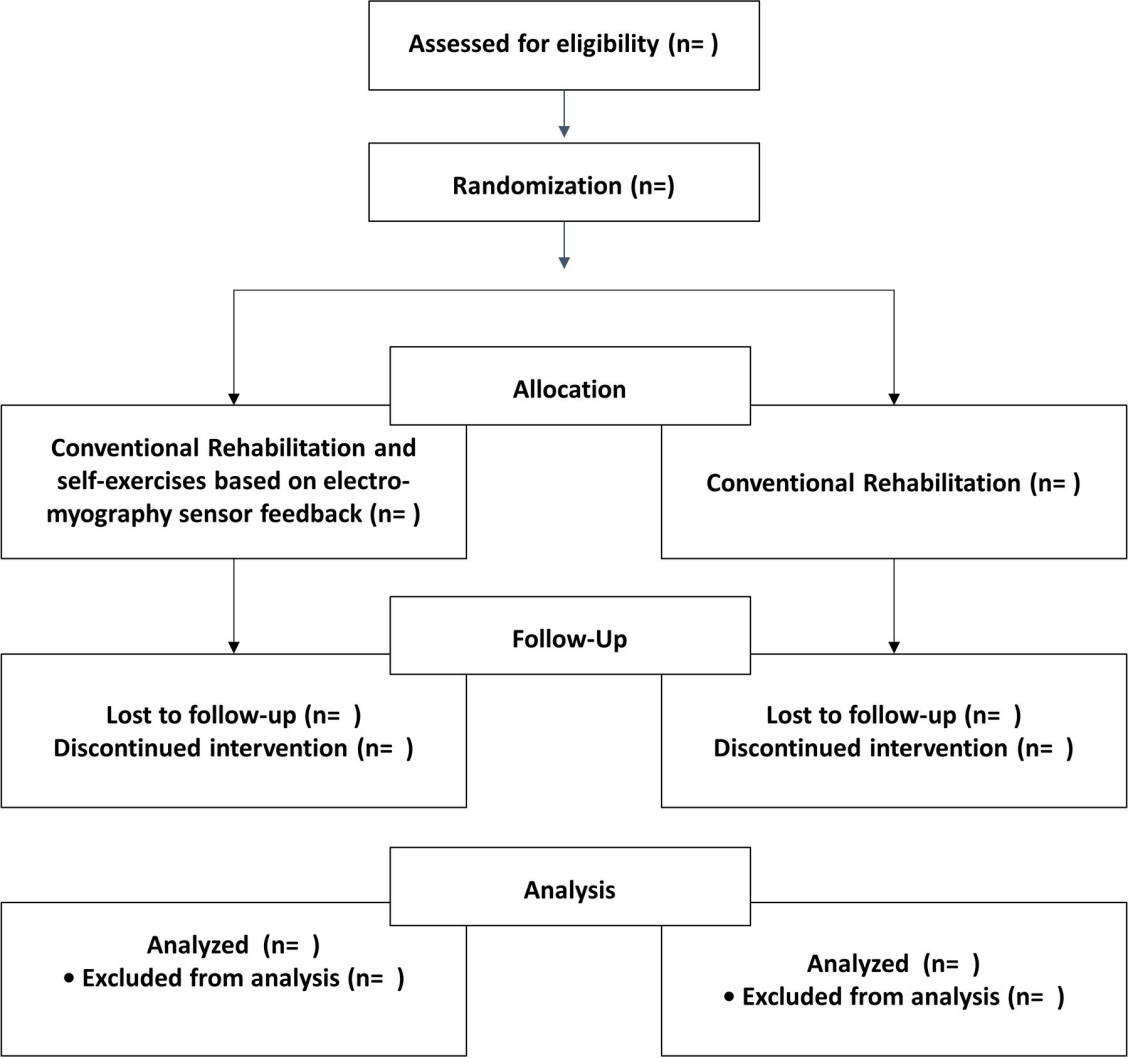
The flow chart.

This study was designed as a single-blind, pilot, randomized controlled clinical trial. This protocol was developed in accordance with the Standard Protocol Items: Recommendations for Interventional Trials (SPIRIT) guidelines (S1 Table) and the TIDier checklist (S2 Table). We designed a stratified random sampling protocol and will conduct a two-arm randomized controlled trial comparing conventional rehabilitation alone with conventional rehabilitation combined with additional in-bed self-exercises based on EMG sensor feedback (Fig 1). Participants will be enrolled from a tertiary hospital and a rehabilitation hospital. This study is a pilot, randomized controlled clinical trial investigating the feasibility and safety of in-bed self-exercises and aims to recruit at least 20 participants. Assuming a 15% loss to follow-up, the required sample size was set at 24 patients (12 patients per group) to be recruited in this study. The study received approval from the Seoul National University Hospital Institutional Review Board (IRB No: D-2208-177-1354, September 21, 2023) (S1 File), and the protocol was registered and approved on the ClinicalTrials.gov (Trial record: NCT05820815).

### 2.2. Eligibility criteria

The inclusion criteria are as follows: (1) age ≥19 years [6]; (2) the onset of stroke ≤3 months [6]; (3) muscle strength at the paretic lower limbs, grade ≤4 as per the Medical Research Council grading scale; (4) modified Rankin Scale 2–5 points; and (5) cognitively able to understand and follow instructions. The exclusion criteria consist of (1) recurrent stroke resulting in neurological deterioration during the study period; (2) other neurological abnormalities that affect balance or gait function (e.g., Parkinson’s disease); (3) severe cognitive impairment; (4) serious or complex medical conditions (e.g., active cancer); and (5) implanted electrical stimulators, which can interfere with measurements of EMG activity (e.g., pacemaker, deep brain stimulator).

### 2.3. Recruitment and allocation

**Fig 2:**
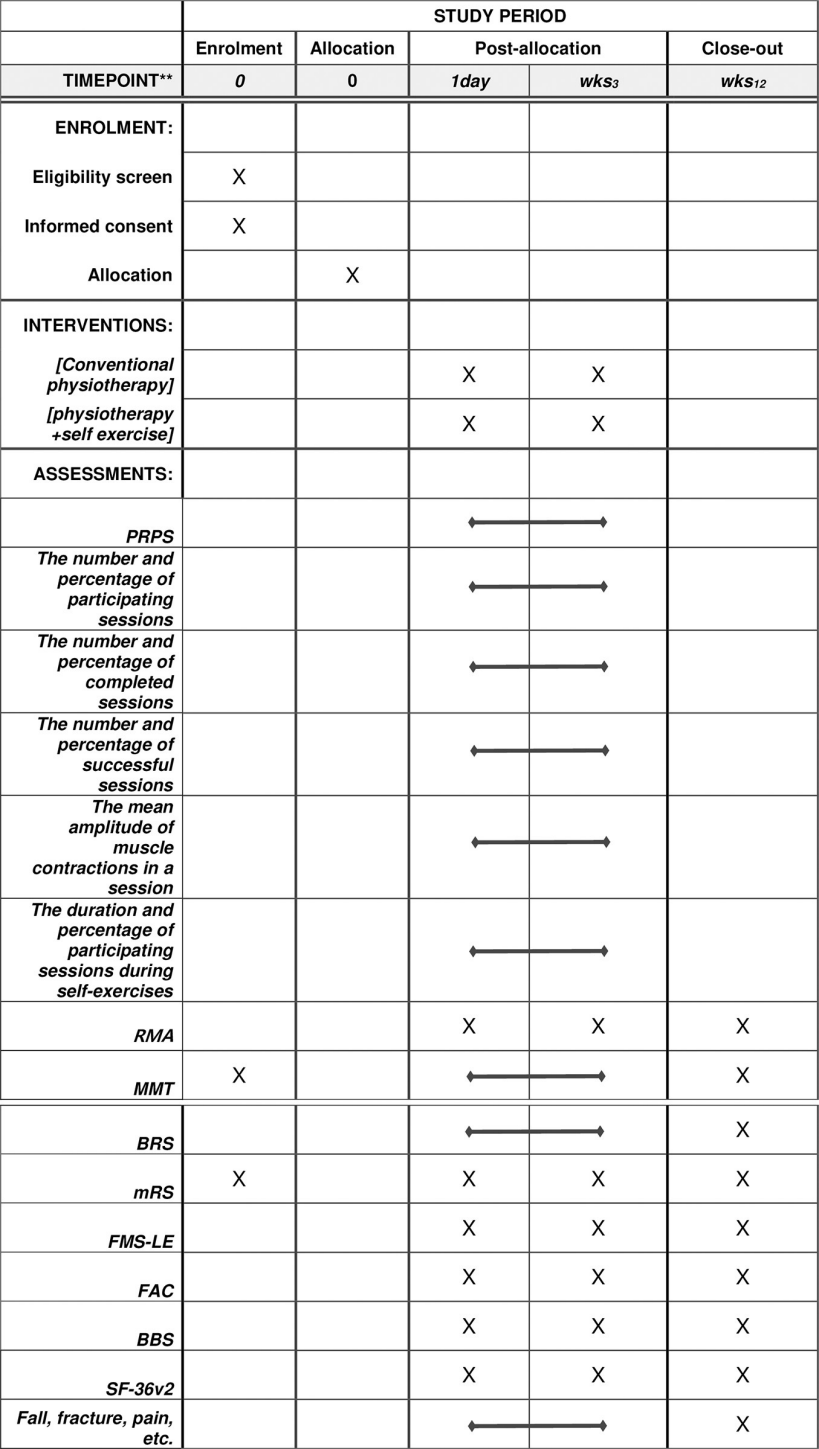
SPIRIT schedule of enrolment, interventions, and assessments of the study. The outcomes include the number and percentage of participating sessions, the duration and percentage of participating sessions during self-exercises, the number and percentage of successful sessions, the mean amplitude of muscle contractions in a session, the number and percentage of completed conventional physiotherapy sessions, the duration and percentage of conventional physiotherapy sessions, and the assessment results for RMA, MMT, BRS, mRS, FMS-LE, FAC, BBS, and SF-36 version 2.0. PRPS: Pittsburgh Rehabilitation Participation Scale; RMA: Revised Motor Assessment; MMT: Manual Muscle Test; BRS: Brunnstrom Recovery Stage; mRS: Modified Rankin Scale; FMS-LE: Fugl–Meyer Assessment of the Lower Extremity; FAC: Functional Ambulation Category; BBS: Berg Balance Scale; SF-36: 36-item Short-Form Survey.

Participants will be recruited by posting a notice on the bulletin boards of the tertiary and rehabilitation hospital. The schedule of enrollment and assessment of the participants are shown in Fig 2. The principal investigator or researchers of this study will provide a detailed explanation of the study to the guardians or patients and will not exclude patients who are likely to participate based on their socioeconomic status. An independent researcher who is not involved in the recruitment, intervention, or evaluation of the patients will generate the allocation sequence using a web-based randomization system to ensure allocation concealment and blinding. Participants will be assigned to the control or intervention group in a 1:1 ratio using the stratified block randomization method, stratified by age and modified Rankin score. Patients will be stratified by age (<65 years and ≥65 years) and by modified Rankin Scale (2–3 and 4–5). Sequence generation will be performed using computer-generated random numbers, and the website will be used to inform the treatment provider when the intervention is assigned. Two therapists, each from a different institution, will assess the primary and secondary outcome measures. These therapists will be blinded to group allocation by blocking access to the randomization table. The patients and treatment providers are instructed not to disclose the allocation to the evaluator. The evaluator and data analyzer are blinded to the group allocation of the patients.

### 2.4. Interventions

#### 2.4.1 Conventional rehabilitation

The conventional stroke rehabilitation program involves a comprehensive and individualized approach that includes range of motion exercises, strengthening exercises, balance and coordination exercises, and exercises for functional mobility such as sitting balance, sit-up, sit-to-stand, standing, and gait [25]. The amount and intensity of the conventional rehabilitation program are equally provided for participants of both intervention and control groups by the same group of physical therapists who are blinded to the group allocation. Physical therapists will provide conventional rehabilitation for 30 minutes each session, two sessions a day, five times a week, in both groups. Patients with stroke may receive physical therapy with walking aids, such as a walker or cane, or a lower-extremity orthosis.

#### 2.4.2 In-bed self-exercises based on EMG-sensor feedback system

**Fig 3:**
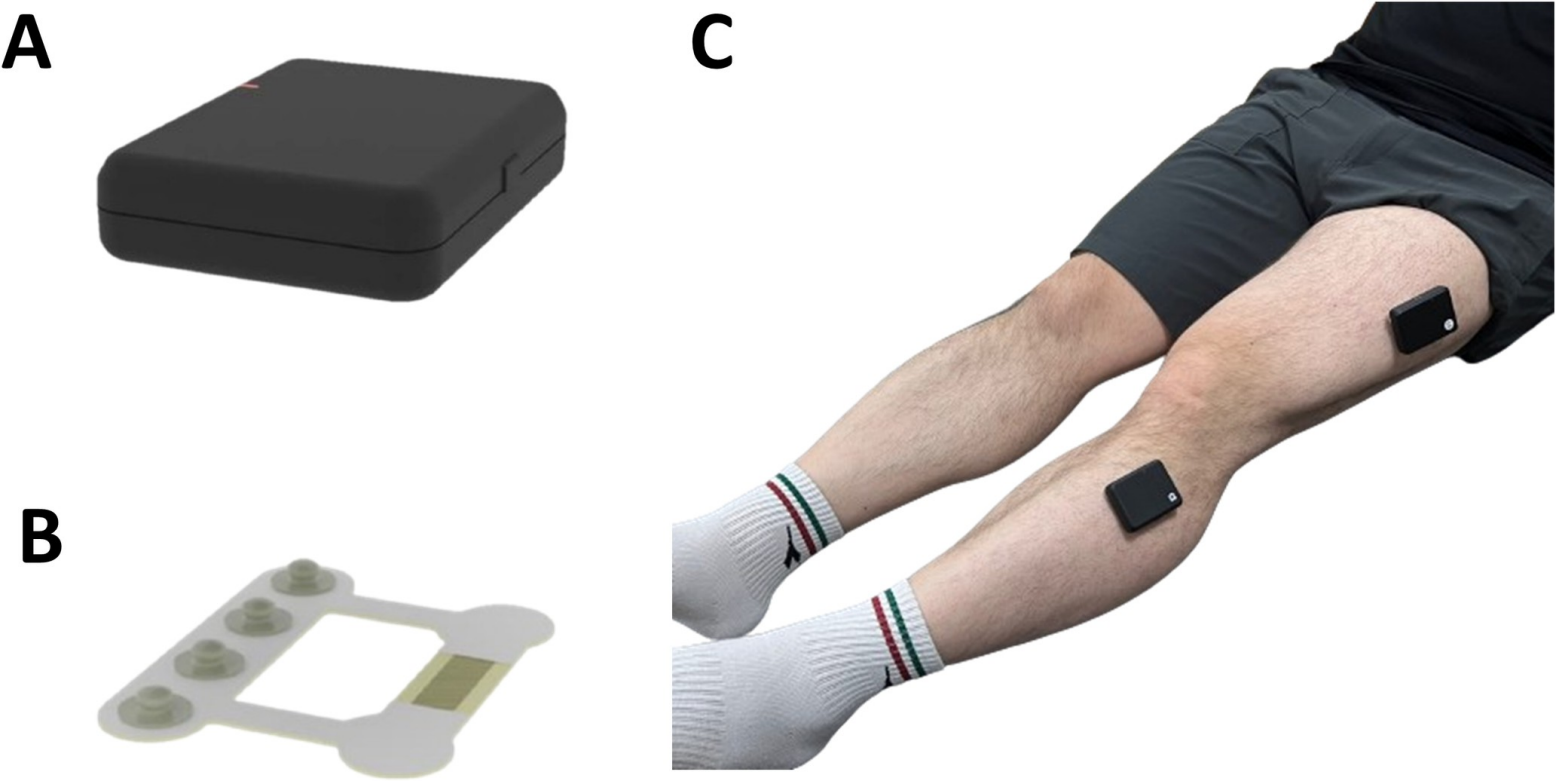
Images of electromyography sensor and sensor patch. (A) An electromyography sensor; (B) electromyography sensor patch; (C) the attachment of EMG sensors to the lower extremities using sensor patches.

In-bed self-exercises based on EMG-sensor feedback are conducted using wireless EMG sensors (HSN2ER3E, SMD Solution, Seoul, Republic of Korea) and mobile applications (HSN2ER3E S/W, Myoverse, SMD Solution, Seoul, Republic of Korea) in Fig 3.

**Fig 4:**
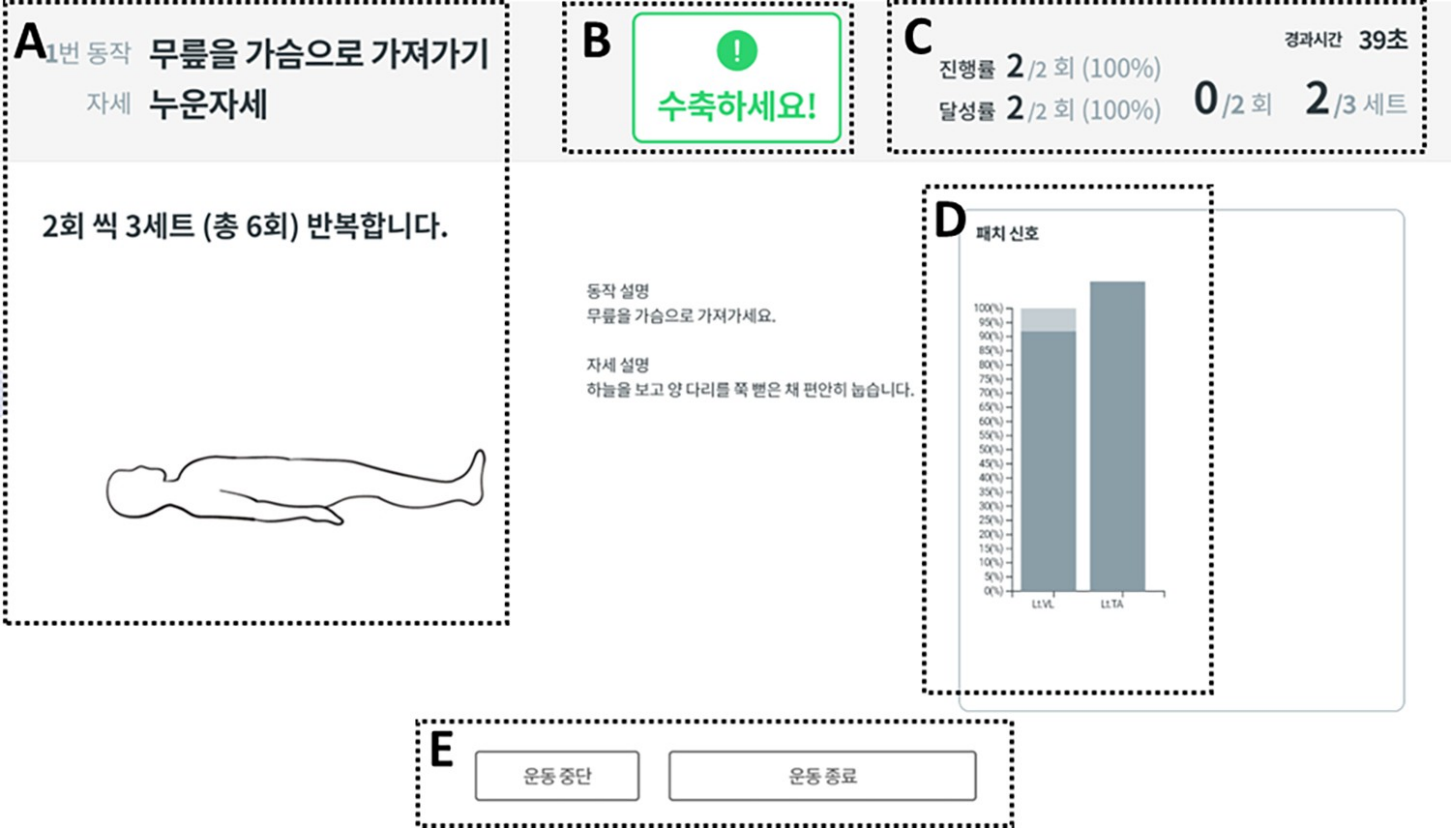
The visualized results of EMG-sensor feedback in the mobile applications for patients with stroke. (A) The type, posture, dose, and instructions of prescribed exercises; (B) a message for participants to contract the target muscles; (C) ongoing exercise performances, including repetitions per set, sessions per day, and sets per session; (D) the bar graph representing the percentage of the peak EMG signals relative to their target amplitude during muscle contractions; (E) buttons for pause and termination of prescribed exercises.

**Fig 5:**
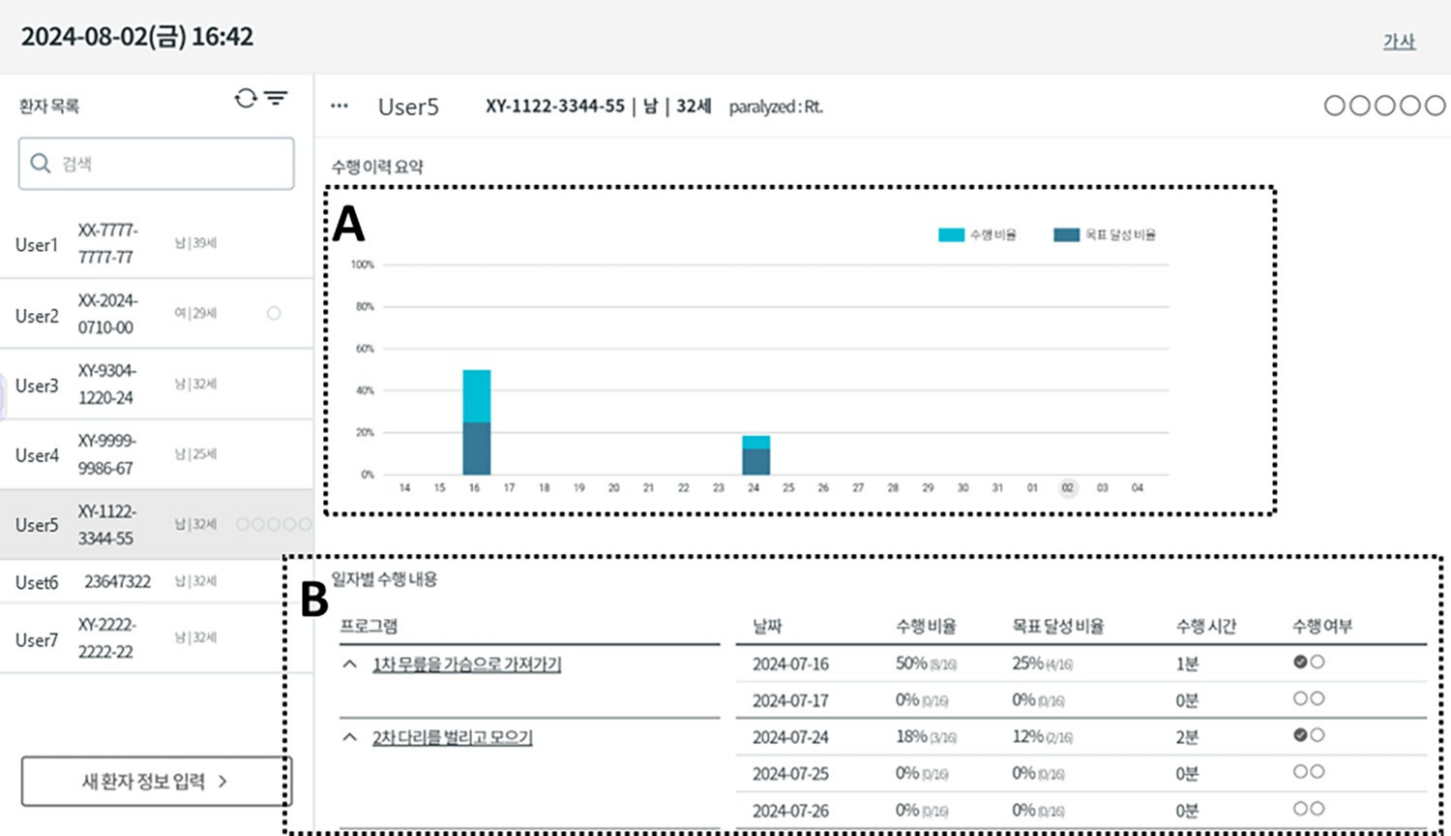
The visualized results of EMG-sensor feedback in the mobile applications for therapists. (A) Visualization of performance history with a Bar Graph; (B) the results of the self-exercises including session frequency and duration, number of sets and repetitions, rest intervals, exercise type, intensity level, and progression.

Two versions of mobile applications are provided for therapists and patients with stroke, respectively, because the contents of information provided for therapists and patients with stroke are different. Fig 4 shows the visualized results of EMG-sensor feedback in the mobile applications for patients with stroke: (A) the type, posture, dose, and instructions of prescribed exercises; (B) a message for participants to contract the target muscles; (C) ongoing exercise performances, including repetitions per set, sessions per day, and sets per session; (D) the bar graph representing the percentage of the peak EMG signals relative to their target amplitude during muscle contractions; (E) buttons for pause and termination of prescribed exercises. The mobile applications for therapists have the following features, including the EMG values of the targeted muscles during self-exercises and the prescription contents of the exercise period, the session, sets, and repetitions for the selected self-exercises, which can be monitored by physical therapists to identify ongoing exercise performances (Fig 5). If the usage frequency of the mobile application is low or the recorded levels of EMG signals are lower than expected, the physical therapists will contact the patients to check their conditions or investigate the reasons for the low performance. Based on the developed an in-bed self-exercise protocol, the exercise program will be adjusted weekly according to the muscle strength and Brunnstrom recovery stage by therapists. Additionally, the exercise regimen is reassessed and prescribed anew each week, with the amplitude of EMG signals during MVC being reassessed each time.

**Fig 6:**
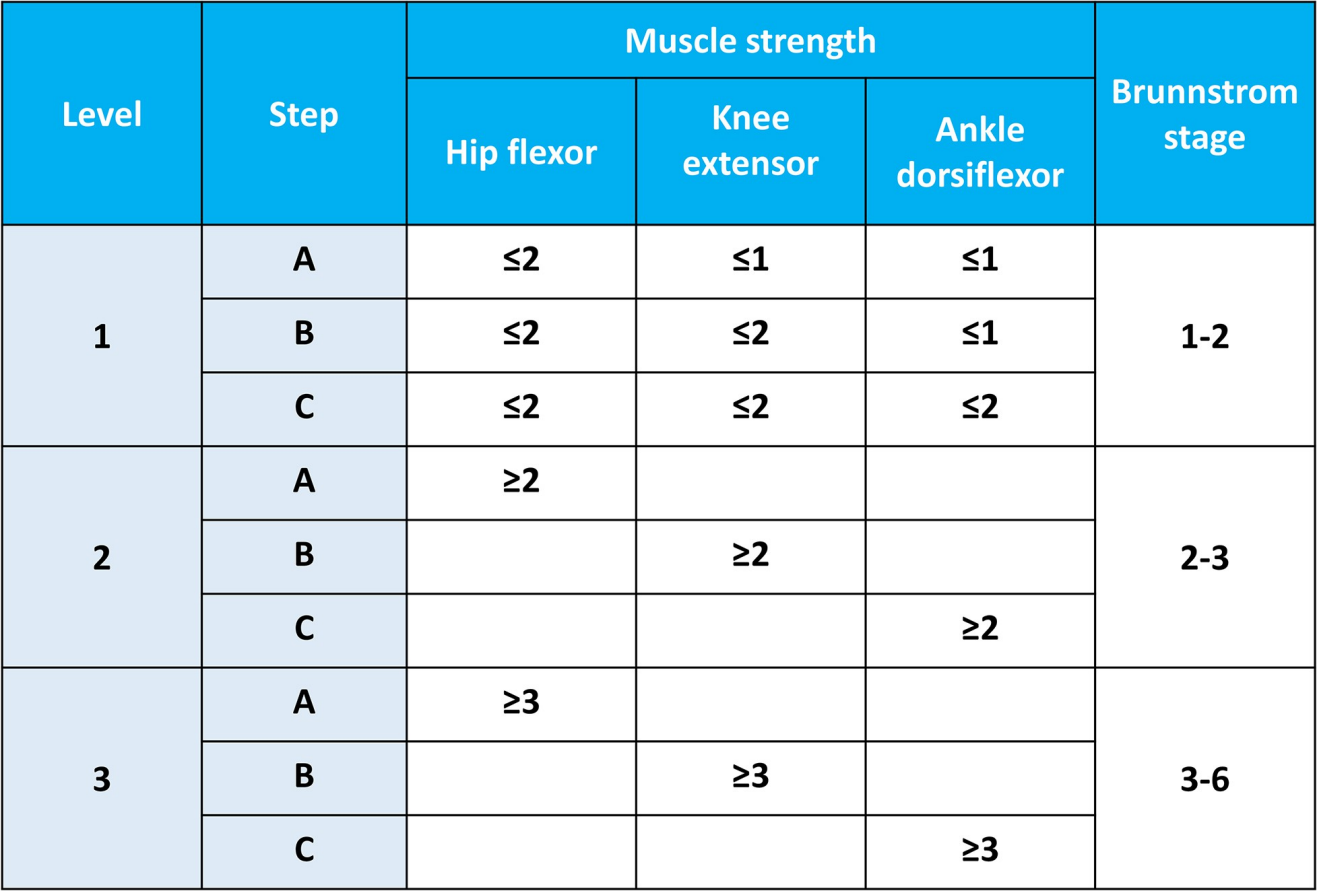
Protocol to determine the level and step based on the Brunnstrom recovery stage and muscle strength.

The participants will be instructed to complete at least two 30-minute sessions per day, 5 days a week, for a duration of 3 weeks of in-bed self-exercises, in addition to their conventional rehabilitation sessions. The participants will be provided with information including the type, dosage (e.g., repetitions, sets, and sessions), duration, intensity (% of EMG activities during maximal voluntary contractions), and body positions of lower-limb exercises, along with real-time feedback on their muscular activities and goal achievement. EMG signals will be measured and collected weekly during the intervention period, and the prescription contents of the self-exercises will be changed considering the results of goal achievement and the levels of Brunnstrom recovery stage (BRS) and muscle strength (Fig 6). Additionally, the exercise information of the participants, including goal achievement, exercise frequency, and exercise duration, will be monitored daily through a mobile applications for therapist. The therapists will provide participants with personal support and education to motivate them and address any barriers to their commitment.

The setting levels of the surface EMG activities at the target muscles are as follows. To acquire EMG signals from the vastus lateralis and tibialis anterior, surface electrodes will be positioned over the muscle bellies along a line parallel to the orientation of the muscle fibers and attached to the skin using a specialized patch. The surface electrode sites will be located at the following positions: (1) at the halfway position on the line from the anterior spina iliac superior to the lateral side of the patella for the vastus lateralis and (2) at the one-third position on the line from the fibular head to the medial malleolus for the tibialis anterior [26, 27]. Surface EMG electrodes will be attached by patients with stroke or caregivers at predetermined locations on the vastus lateralis and tibialis anterior muscles, which will be marked by the therapists. The posture of the self-exercises to activate the lower-limb muscles will be determined and instructed before maximal voluntary contractions. This study adopted the target level of EMG activities for patients with subacute stroke when engaging in self-exercises as 50% of the mean activities during three trials of the maximal voluntary contractions (MVC) according to a previous systematic review [28].

**Fig 7:**
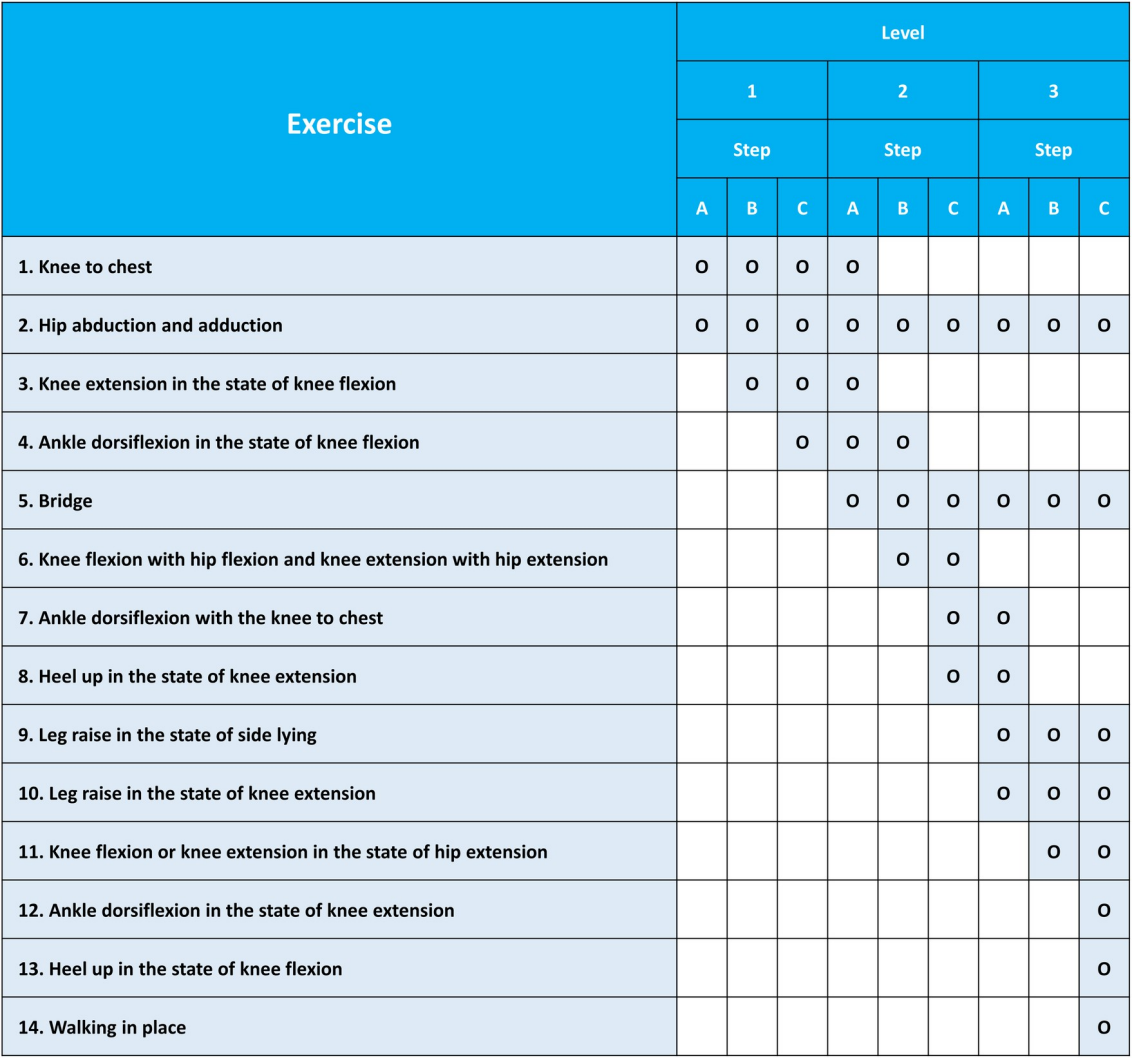
Contents of self-exercises based on the levels and steps in patients with subacute stroke.

**Fig 8:**
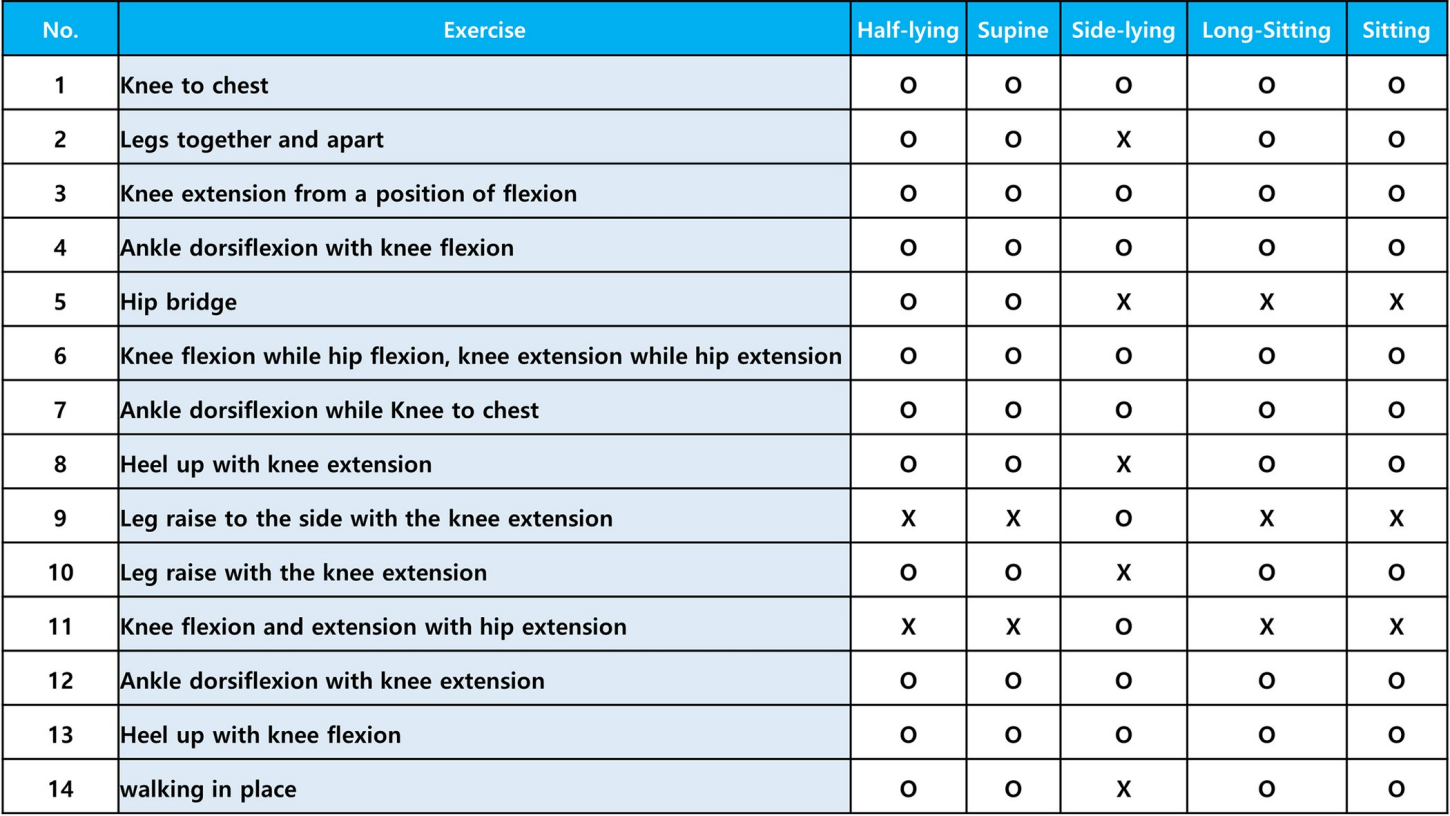
Contents of self-exercises based on postures in patients with subacute stroke.

The principles for determining the level and step of in-bed self-exercises in stroke patients are presented in Figs 6–8. There are three levels according to the BRS, and each level has three steps according to the Medical Research Council scale for muscle strength. The specific principles are as follows: (A) The level and step are determined based on the BRS and muscle strength, and self-exercises are prescribed by physiatrists or physical therapists accordingly. (B) The level or step and exercises prescription can be changed based on the BRS and muscle strength by physiatrists or physical therapists every week. (C) Up to four types of self-exercises can be selected during a 1-week period, and exercises corresponding to the highest level or step are recommended by physiatrists or physical therapists considering the patient’s condition. (D) The level or step may overlap, and exercises corresponding to the highest level are recommended based on the patient’s condition. (E) The physiatrists or physical therapists determine postures that patients can perform safely and effectively during exercises. If possible, postures with high functional mobility are recommended. Caregivers can help participants to adjust and maintain postures during in-bed self-exercises, if necessary. (F) Information on the types of exercises, sessions per day, sets per session, repetitions per set, and target amplitude of the EMG signals during exercises will be provided to the patients. In general, self-exercises will be recommended at 2 types of self-exercises per session, 2–3 sessions per day, 3–5 sets per session, and 5–10 repetitions per set, with an amplitude of at least 50% of the amplitude of EMG signals during maximal muscle contraction. This will be determined by physiatrists or physical therapists based on the patient’s degree of exercises performance. (G) It is recommended to initially perform truncal balance exercises for 10 minutes as a warm-up to ensure safety before initiating sessions of in-bed self-exercises. Truncal balance exercises may include pelvic tilts, seated or standing trunk rotations, leg lifts while maintaining a stable trunk, bridging, and reaching tasks while seated or standing. Truncal balance exercises are also considered as a part of the conventional rehabilitation program in both groups. Thus, truncal balance exercises will be provided for participants in both intervention and control groups as conventional rehabilitation if necessary. (H) It is recommended that two or fewer EMG sensors are used during self-exercises and attached to the sites of paralyzed muscles, where the physiatrists or physical therapists indicate.

### 2.5. Outcome measures

#### 2.5.1. Primary and secondary outcome measures

The baseline clinical characteristics will be collected as follows: age, sex, dominant hand, presence or absence of other diseases (high blood pressure, diabetes, hyperlipidemia, atrial fibrillation, Charlson comorbidity index), level of education; caregiver (spouse, children, others), National Institutes of Health Stroke Scale score, stroke treatment (thrombolysis/thrombectomy), stroke onset (day), lesion type (infarction, hemorrhage), lesion location (supra-tentorial, infra-tentorial), lesion laterality (right, left), stroke causes (TOAST classification: large-artery atherosclerosis/small-vessel occlusion/cardioembolic/other determined etiology/undetermined etiology), medicines for depression, spasticity, and epilepsy, Mini-Mental State Examination score, motor-evoked potential, Hospital Anxiety and Depression Scale score, and orthosis.

**Fig 9:**
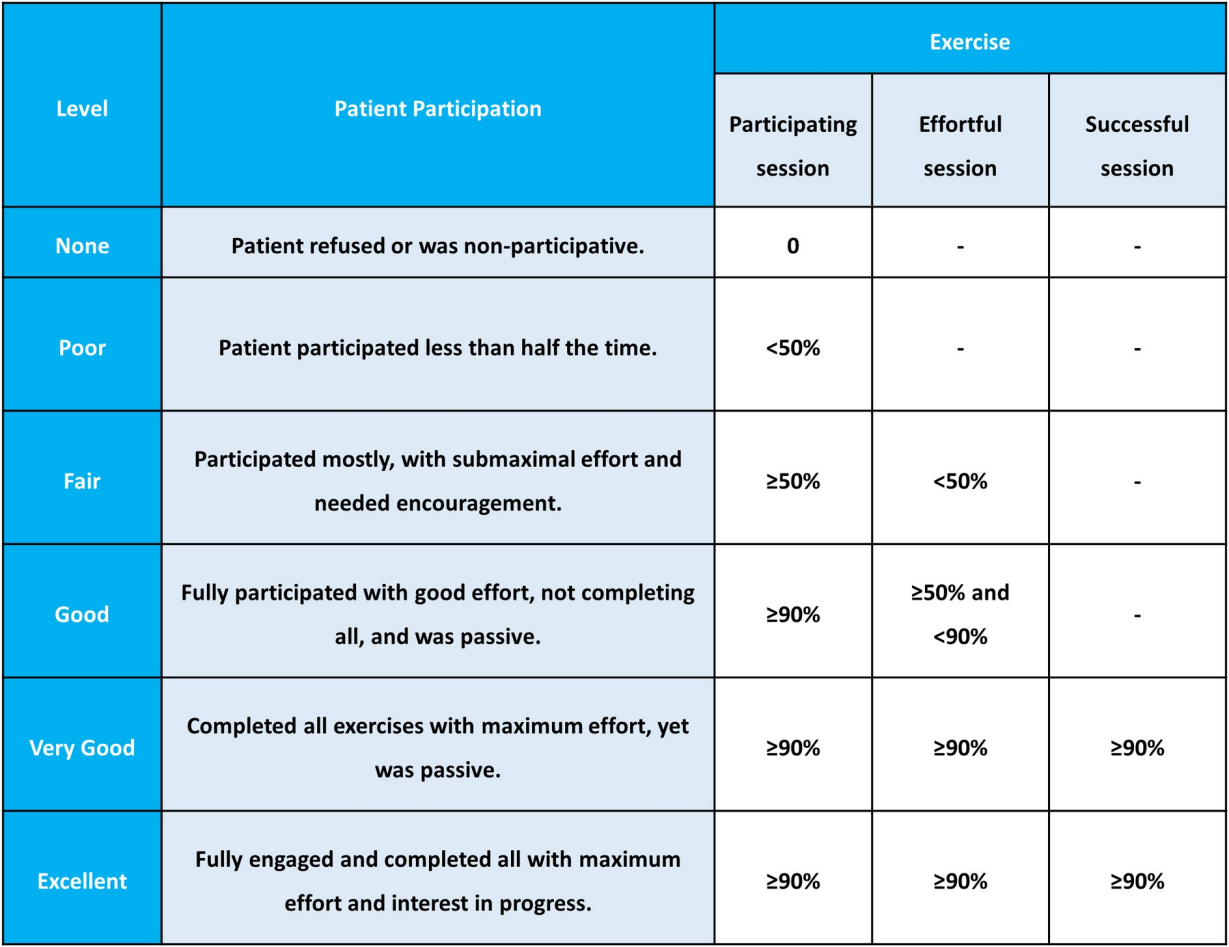
Pittsburgh Rehabilitation Participation Scale and newly defined exercise sessions.

The primary outcome measure is the Pittsburgh Rehabilitation Participation Scale (PRPS). The PRPS is a clinician-administered instrument designed to assess the participation of patients in rehabilitation [29, 30]. It is rated on a scale of 1–6 to measure a patient’s effort and activity to participate in rehabilitation. The patient’s session participation and effort levels were assessed across six distinct categories, ranging from "none" to "excellent." The "none" category signifies that the patient either declined to participate in the entire session or did not engage in any exercises during the session. Conversely, the "excellent" category denotes that the patient actively participated in all exercises with maximal effort, successfully completing each exercise and displaying a keen interest in both the exercises and any future therapy sessions. The remaining categories describe varying degrees of participation and effort between these two extremes [30]. In the PRPS, there are three types of exercise sessions to be newly defined as follows: (i) a participating session, in which the number of contractions of target muscles is more than one; (ii) an effortful session, in which the amplitude of one or more target muscles during contractions is more than 20% of maximal voluntary isometric contraction; (iii) a successful session, a session in which the amplitude of one or more target muscles during contractions is more than 50% of maximal voluntary isometric contraction. The six categories of PRPS using the concept of participating, effortful, and successful sessions are defined as follows: (i) none, 0% of participating sessions; (ii) poor, <50% of participating sessions; (iii) fair, ≥50% of participating sessions and <50% of effortful sessions; (iv) good, ≥90% of participating sessions, ≥50% and <90% of effortful sessions; (v) very good, ≥90% of participating sessions, ≥90% of effortful sessions, and ≥90% of successful sessions; (vi) excellent, ≥90% of participating sessions, ≥90% of effortful sessions, ≥90% of successful sessions, and active involvement in establishing exercise plans for future sessions (Fig 9).

The secondary outcome measures are the number and percentage of participating sessions, the number and percentage of effortful sessions, the number and percentage of successful sessions, the mean amplitude of muscle contractions in a session, and the duration and percentage of participating sessions during self-exercises. Rivermead Motor Assessment, Manual Muscle Test for muscle strength, BRS, Fugl–Meyer Assessment of the Lower Extremities, Berg Balance Scale, Functional Ambulation Category, modified Rankin scale, and Short-Form Health Survey version 2. As a muscle strength measurement test, the Manual Muscle Test is evaluated on a scale of 0–5 according to strength against gravity and resistance. In this study, the Manual Muscle Test of the hip flexor, hip abductor, knee extensor, and ankle dorsiflexor will be measured. Manual Muscle Test has good external and internal efficacy and is not dependent on examiner bias [31]. The BRS is a classification method that models the motor recovery process after stroke-induced hemiplegia on a 6-point ordinal scale. The Brunnstrom approach focuses on the unique patterns associated with stroke recovery, including spasticity development, synergistic patterns, and voluntary movements. The BRS has high inter-rater reliability (0.74–0.98) [32]. The Fugl–Meyer Assessment of the Lower Extremities investigates hip, knee, and ankle movements, and hierarchical recovery is recorded based on Brunnstrom stages of recovery from reflex to synergistic and non-synergistic movements. The Fugl–Meyer Assessment of the Lower Extremities motor domain uses a 3-point ordinal scale: 0, unable to perform; 1, partial performance; and 2, complete performance. Possible scores range from 0 to 34. The intra- and inter-rater reliabilities are excellent in early stroke patients [33]. The Berg Balance Scale was developed to objectively measure balance and fall risk in community-dwelling older adults using a three-step survey of 32 health professionals. The Berg Balance Scale examines 14 movements of daily life on a 5-point ordinal scale from 0–4, and the total score ranges from 0–56. The Berg Balance Scale is widely used to assess stroke patients, and its test-retest reliability and internal consistency are excellent [34]. The Functional Ambulation Category is a 6-point rating scale that assesses the amount of human support required when walking (with or without a personal assistive device). A score of 0 indicates a non-functional ambulator, and a score of 1–3 indicates a dependent ambulator. A score of 1 indicates the need for continuous manual contact, 2 indicate intermittent or continuous light touch, and 3 indicates supervision or verbal cues. Scores of 4–5 indicate an independent ambulator, with a score of 4 indicating independent ambulators on horizontal surfaces only and a score of 5 indicating independent ambulators on any surface, including stairs. There is good inter-rater reliability among examiners in post-stroke patients [35]. The modified Rankin scale is a widely used tool to measure global disability after stroke. The scale classifies disability from 0 (no symptoms) to 5 (severe disability). Scoring is performed by an evaluator based on patients’ functional dependence. The intra-rater reliability is excellent [36]. The Rivermead Motor Assessment consists of three sections, and sections of gross function and leg and trunk are evaluated in this study. The gross function section consists of 13 items and primarily assesses mobility from sitting to running and gait, and the leg and trunk section describes individual movements of the trunk (e.g., rolling to the affected side) and leg (e.g., ankle dorsiflexion with the leg extended while lying down). The intra-class correlation coefficients of the Rivermead Motor Assessment is between 0.88 and 0.95 [37]. The Short-Form Health Survey version 2 is a well-studied self-reported measure of functional health. The items include physical functioning, physical role limitation, pain, general medical health, vitality, social functioning, emotional role limitation, mental health, physical component scale, and mental component scale. There is good internal consistency of >0.7 for all the subscales of the questionnaire [38]. Among the assessments for the baseline status and primary and secondary outcomes, the official Korean version of the Fugl–Meyer Assessment, Berg Balance Scale, Short-Form Health Survey 36 version 2, and the Hospital Anxiety and Depression Scale will be used in this study [39–42]. The assessors who are proficient in English will conduct the English version of the assessments, including the Pittsburgh Rehabilitation Participation Scale, Rivermead Motor Assessment, Manual muscle test, Brunnstrom recovery stage, modified Rankin scale, and Functional Ambulation Category. The Short-Form Healthy Surgery 36 version 2 and the Hospital Anxiety and Depression Scale are self-administered questionnaires, and all other evaluations are clinician-administered assessments. All primary and secondary outcomes will be assessed at baseline, and at 3 weeks and 12 weeks. Assessments will be performed over two days to manage fatigue and reduce potential bias in stroke patients. The Fugl–Meyer Assessment, Berg Balance Scale, Manual muscle test, and Short-Form Health Survey 36 version 2 will be conducted on the first day, and the Rivermead Motor Assessment, Brunnstrom Recovery Stage, modified Rankin Scale, and Functional Ambulation Category will be conducted on the second day. Participants will be instructed and familiarized before the actual assessments to help them understand the evaluation processes and improve compliance. Regular breaks will be provided during assessment sessions to prevent fatigue and maintain the participants’ attention.

**Fig 10:**
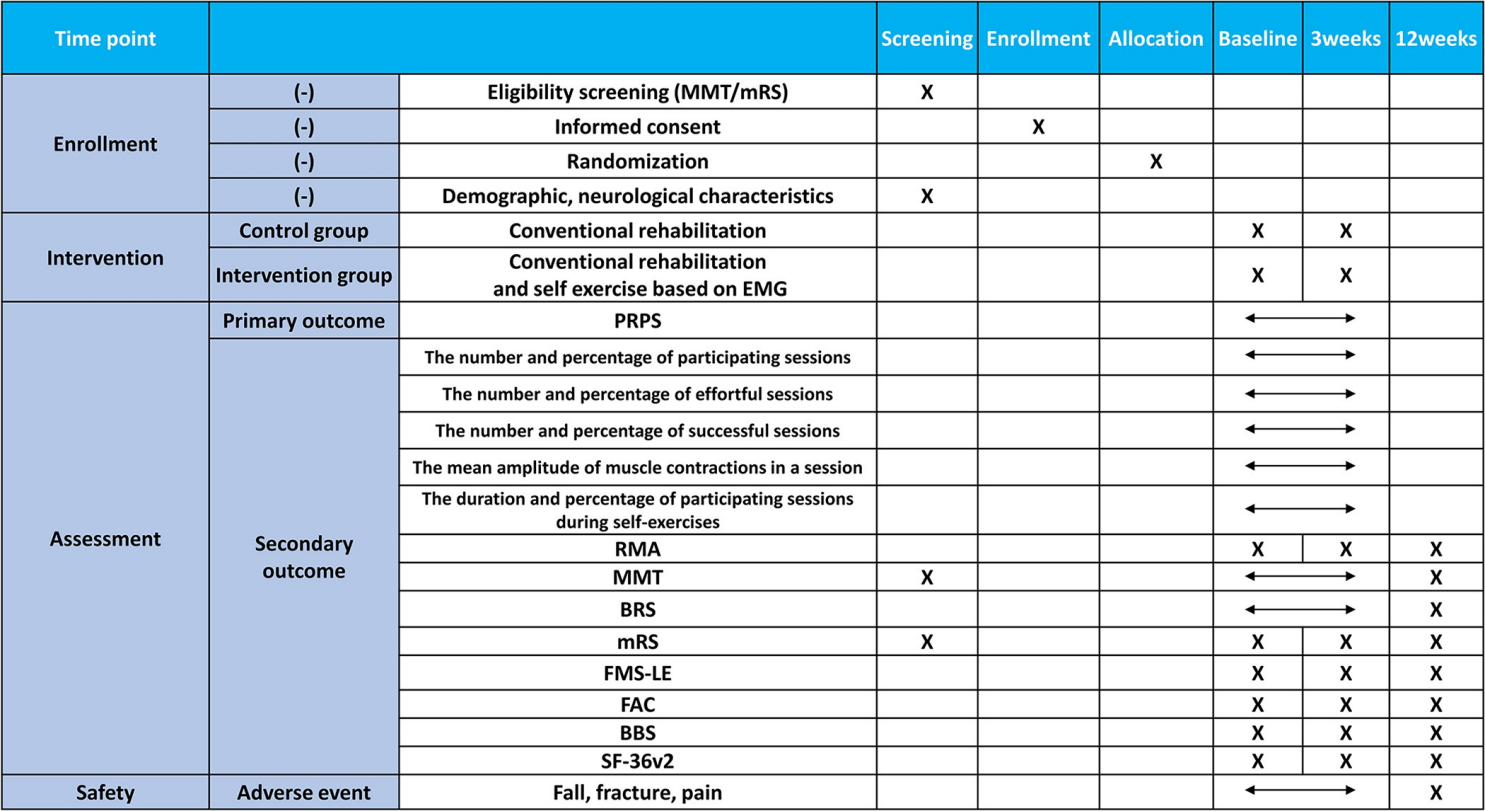
Schedule of enrollment and study assessments. PT, Physical therapy; EMG, Electromyography; PRPS, Pittsburgh Rehabilitation Participation Scale; RMA, Rivermead Motor Assessment; MMT, Manual Muscle Test; BRS, Brunnstrom Recovery Stage; mRS, Modified Rankin Scale; FMA-LE, Fugl–Meyer Assessment of the Lower Extremities; FAC, Functional Ambulation Category; BBS, Berg Balance Scale; SF-36v2 36-item Short-Form Health Survey version 2.

The schedule of enrollment and assessment of the patients is shown in Fig 10. After admission, patients will be screened according to the inclusion criteria. After screening, the guardians of patients who met the inclusion criteria will be given an information sheet and asked to provide written consent to participate in the trial. Patients are then enrolled in the trial, given a trial-specific identification number, and randomly allocated to a group using a computer-generated random sequence listed prior to the start of the trial. After baseline evaluation, patients will be assessed at 3 and 12 weeks. The intervention period consists of 3 weeks of in-bed self-exercises based on EMG sensor feedback. Assessments at 12 weeks serve as a follow-up evaluation for the long-term effects after a 3-week intervention.

### 2.6. Adverse events

Potential adverse events, including falls, fractures, severe pain, symptoms of respiratory or hemodynamic instability (e.g., dyspnea, tachypnea, dizziness, arrhythmia, bradycardia, high or low systolic blood pressure, or oxygen desaturation), and discomfort at the sensor attachment sites, will be monitored during exercises. To prevent falls, in-bed self-exercises should be performed with the bed rails raised, which are instructed to participants and their caregivers. If an adverse event occurs, the principal investigator promptly reports and documents it to the IRB.

### 2.7. Management

The researchers will ensure the protection of participant anonymity and the confidentiality of data related to individuals, safeguarding their identities and any identifying information. Clinical records, research instruments, or any documents containing participant data will be identified by a code instead of the participant’s name, even when submitted to regulatory institutions or sponsors. The researchers will secure the records of the participants and maintain the confidentiality of information regarding codes, names, and addresses, limiting access to only the researchers. All files will be stored in a dedicated, secure location within a single folder for this study.

Participation in this study is entirely voluntary, and non-participation will not impact the patient’s medical follow-up or the relationship between the team and the patient. Upon signing the consent form, patients may choose to withdraw from the study at any time without consequences regarding their treatment or follow-up at the institution.

The risks during the research are expected to be minimal. The evaluation may be temporarily halted to allow for necessary medical interventions. However, if discomfort persists, the participant will be excluded from the research, any collected data will be excluded from analysis, and the patient will not be reassigned to another group.

### 2.8. Statistical methods

All statistical analyses will be performed for intention-to-treat and a per-protocol population. Descriptive statistics, including mean and standard deviation, median and range, or number and percentage, will be provided for all assessed outcomes. To investigate the effects of time and groups on outcome variables, a repeated measures design with a linear mixed model will be employed. The linear mixed model will include time (baseline, 3 weeks, and 12 weeks), group (control and intervention groups), and time*group interaction as fixed effects, while sex, height, and weight will be treated as random effects. Assumptions of normality, independence, and homoscedasticity will be checked to ensure the validity of the model. Effect sizes with 95% confidence intervals will be calculated. Missing data will be handled using multiple imputation or maximum likelihood estimation. A two-tailed approach will be used. The significance level will be set at p<0.05. All statistical analyses will be conducted using SPSS (version 25.0; IBM, Armonk, NY).

## 3. Discussion

This study aims to develop standardized, individualized, semi-supervised, in-bed, and self-exercise protocols and to investigate the feasibility and safety in patients with stroke.

Previous studies have shown a dose-dependent relationship between the intensity of rehabilitation therapies and functional recovery in stroke patients, particularly in walking ability, walking speed, and extended activities of daily living [43–45]. However, owing to the limited resources in the hospital rehabilitation system, it is difficult to achieve the optimal dose.

In-bed self-exercises based on EMG sensor feedback can be a good option for enabling stroke patients to perform the exercises with intermittent supervision. These exercises can be adjusted according to the goal achievement and improvement of function and muscle strength. In addition, they can provide stroke patients with objective and accurate information during practice to induce self-modification of behaviors, increase the rate of motor learning, and improve the time efficiency of therapy [46, 47]. To clarify the exercises description based on the proposed framework developed in a previous study, the exercises results in this study will be described with respect to the type, dosage, duration, intensity, and body positions, as well as the participation scale, rate and duration of exercises performance, and rate of goal achievement. In-bed self-exercises based on EMG-sensor feedback and supervision enable exercises with high dosage and intensity, along with a low fall risk for stroke patients with limited medical resources.

In this study, the PRPS is implemented as the primary outcome to assess patient engagement in in-bed self-exercises based on the EMG activities, addressing the absence of direct supervision by physical therapists. It categorizes patient participation into three types of exercise sessions, including participating, effortful, and successful sessions, which are defined by the number and percentage of contractions of target muscles and EMG amplitude of target muscles during contractions in this study. This approach allows physical therapists to evaluate the degree of patient participation in in-bed self-exercises quantitatively and to aid in planning the dose and intensity of future sessions without direct supervision. Using PRPS measurements based on EMG activities can be beneficial to assess patient participation in various forms of self-rehabilitation where therapist involvement is minimal.

The multicenter setting of this study may lead to potential inconsistencies between sites with respect to outcome assessments and exercises instructions. To mitigate this issue, careful monitoring and standardization of assessment techniques along with exercises instructions will be necessary. The research group will coordinate training sessions to implement proper assessment techniques and standardization at both study sites and hold regular meetings to maintain adherence to standard operating procedures.

## Supporting information

S1 Table SPIRIT checklist. (DOC)

S2 Table Template for Intervention Description and Replication (TIDieR) checklist. (DOCX)

S1 File Detailed protocol submitted to the ethics committee. (PDF)

S1 Protocol (PDF)

S2 Protocol (PDF)

## References

[1] Benjamin EJ, Blaha MJ, Chiuve SE, Cushman M, Das SR, Deo R, et al. Heart disease and stroke statistics—2017 update: a report from the American Heart Association. circulation. 2017;135(10):e146–e603. doi: 10.1161/CIR.0000000000000485 28122885 PMC5408160

[2] Bahouth MN, Zink EK, Ahmad O, Roy P, Zeiler SR, Urrutia VC, et al. Bringing High-Dose Neurorestorative Behavioral Training Into the Acute Stroke Unit. American Journal of Physical Medicine & Rehabilitation. 2023;102(2S):S33–S7. 10.1097/PHM.0000000000002146 36634328

[3] Donnellan-Fernandez K, Ioakim A, Hordacre B. Revisiting dose and intensity of training: Opportunities to enhance recovery following stroke. Journal of Stroke and Cerebrovascular Diseases. 2022;31(11):106789. doi: 10.1016/j.jstrokecerebrovasdis.2022.106789 36162377

[4] Sabo B, Abdullahi A, Badaru UM, Saeys W, Truijen S. Predictors of high dose of massed practice following stroke. Translational Neuroscience. 2022;13(1):181–90. doi: 10.1515/tnsci-2022-0228 35903752 PMC9285765

[5] Nudo RJ, Milliken GW. Reorganization of movement representations in primary motor cortex following focal ischemic infarcts in adult squirrel monkeys. Journal of neurophysiology. 1996;75(5):2144–9. doi: 10.1152/jn.1996.75.5.2144 8734610

[6] Klassen TD, Dukelow SP, Bayley MT, Benavente O, Hill MD, Krassioukov A, et al. Higher doses improve walking recovery during stroke inpatient rehabilitation. Stroke. 2020;51(9):2639–48. doi: 10.1161/STROKEAHA.120.029245 32811378

[7] Hornby TG, Henderson CE, Plawecki A, Lucas E, Lotter J, Holthus M, et al. Contributions of stepping intensity and variability to mobility in individuals poststroke: A randomized clinical trial. Stroke. 2019;50(9):2492–9. 31434543 10.1161/STROKEAHA.119.026254 PMC7241260

[8] Winstein CJ, Stein J, Arena R, Bates B, Cherney LR, Cramer SC, et al. Guidelines for adult stroke rehabilitation and recovery: a guideline for healthcare professionals from the American Heart Association/American Stroke Association. Stroke. 2016;47(6):e98–e169. doi: 10.1161/STR.0000000000000098 27145936

[9] Simoens S, Villeneuve M, Hurst J. Tackling nurse shortages in OECD countries. 2005.

[10] Petrou S, Wolstenholme J. A review of alternative approaches to healthcare resource allocation. Pharmacoeconomics. 2000;18:33–43. doi: 10.2165/00019053-200018010-00004 11010602

[11] Newacheck PW, Hughes DC, Hung Y-Y, Wong S, Stoddard JJ. The unmet health needs of America’s children. Pediatrics. 2000;105(Supplement_3):989–97. 10742361

[12] Clarke DJ, Burton L-J, Tyson SF, Rodgers H, Drummond A, Palmer R, et al. Why do stroke survivors not receive recommended amounts of active therapy? Findings from the ReAcT study, a mixed-methods case-study evaluation in eight stroke units. Clinical rehabilitation. 2018;32(8):1119–32. doi: 10.1177/0269215518765329 29582712 PMC6068965

[13] Batchelor FA, Mackintosh SF, Said CM, Hill KD. Falls after stroke. International Journal of Stroke. 2012;7(6):482–90. doi: 10.1111/j.1747-4949.2012.00796.x 22494388

[14] Saadatnia M, Shahnazi H, Khorvash F, Esteki-Ghashghaei F. The impact of home-based exercise rehabilitation on functional capacity in patients with acute ischemic stroke: a randomized controlled trial. Home Health Care Management & Practice. 2020;32(3):141–7.

[15] Mandigout S, Chaparro D, Borel B, Kammoun B, Salle J-Y, Compagnat M, et al. Effect of individualized coaching at home on walking capacity in subacute stroke patients: A randomized controlled trial (Ticaa’dom). Annals of Physical and Rehabilitation Medicine. 2021;64(4):101453. doi: 10.1016/j.rehab.2020.11.001 33197648

[16] Cunningham P, Turton AJ, Van Wijck F, Van Vliet P. Task-specific reach-to-grasp training after stroke: development and description of a home-based intervention. Clinical Rehabilitation. 2016;30(8):731–40. doi: 10.1177/0269215515603438 26337625

[17] Palmcrantz S, Borg J, Sommerfeld D, Plantin J, Wall A, Ehn M, et al. An interactive distance solution for stroke rehabilitation in the home setting–A feasibility study. Informatics for Health and Social Care. 2017;42(3):303–20. doi: 10.1080/17538157.2016.1253015 27918220

[18] Knepley KD, Mao JZ, Wieczorek P, Okoye FO, Jain AP, Harel NY. Impact of telerehabilitation for stroke-related deficits. Telemedicine and e-Health. 2021;27(3):239–46. doi: 10.1089/tmj.2020.0019 32326849

[19] Lennon S, McKenna S, Jones F. Self-management programmes for people post stroke: a systematic review. Clinical Rehabilitation. 2013;27(10):867–78. doi: 10.1177/0269215513481045 23543340

[20] Everard G, Luc A, Doumas I, Ajana K, Stoquart G, Edwards MG, et al. Self-Rehabilitation for Post-Stroke Motor Function and Activity–A Systematic Review and Meta-Analysis. Neurorehabilitation and neural repair. 2021;35(12):1043–58. doi: 10.1177/15459683211048773 34696645

[21] Chin LF, Hayward KS, Chai ALM, Brauer SG. A Self-Empowered Upper Limb Repetitive Engagement program to improve upper limb recovery early post-stroke: phase II pilot randomized controlled trial. Neurorehabilitation and Neural Repair. 2021;35(9):836–48. doi: 10.1177/15459683211032967 34281405

[22] Jung S-H, Park E, Kim J-H, Park B-A, Yu J-W, Kim A-R, et al., editors. Effects of self rehabilitation video exercises (Save) on functional restorations in patients with subacute stroke. Healthcare; 2021: MDPI. 10.3390/healthcare9050565 PMC8150768 34064979

[23] Hung Y-X, Huang P-C, Chen K-T, Chu W-C. What do stroke patients look for in game-based rehabilitation: a survey study. Medicine. 2016;95(11). doi: 10.1097/MD.0000000000003032 26986120 PMC4839901

[24] Shaughnessy M, Resnick BM, Macko RF. Testing a model of post‐stroke exercise behavior. Rehabilitation nursing. 2006;31(1):15–21. doi: 10.1002/j.2048-7940.2006.tb00005.x 16422040

[25] Nair K, Taly A. Stroke rehabilitation: traditional and modern approaches. Neurol India. 2002;50(50):85–93.

[26] Hemmati L, Piroozi S, Rojhani-Shirazi Z. Effect of dual tasking on anticipatory and compensatory postural adjustments in response to external perturbations in individuals with nonspecific chronic low back pain: electromyographic analysis. Journal of Back and Musculoskeletal Rehabilitation. 2018;31(3):489–97. doi: 10.3233/BMR-170992 29332033

[27] Rainoldi A, Melchiorri G, Caruso I. A method for positioning electrodes during surface EMG recordings in lower limb muscles. Journal of neuroscience methods. 2004;134(1):37–43. doi: 10.1016/j.jneumeth.2003.10.014 15102501

[28] Lee J, Stone AJ. Combined aerobic and resistance training for cardiorespiratory fitness, muscle strength, and walking capacity after stroke: a systematic review and meta-analysis. Journal of Stroke and Cerebrovascular Diseases. 2020;29(1):104498. doi: 10.1016/j.jstrokecerebrovasdis.2019.104498 31732460

[29] Iosa M, Galeoto G, De Bartolo D, Russo V, Ruotolo I, Spitoni GF, et al. Italian Version of the Pittsburgh Rehabilitation Participation Scale: Psychometric Analysis of Validity and Reliability. Brain Sciences. 2021;11(5):626. doi: 10.3390/brainsci11050626 34068212 PMC8153139

[30] Lenze EJ, Munin MC, Quear T, Dew MA, Rogers JC, Begley AE, et al. The Pittsburgh Rehabilitation Participation Scale: reliability and validity of a clinician-rated measure of participation in acute rehabilitation. Archives of physical medicine and rehabilitation. 2004;85(3):380–4. doi: 10.1016/j.apmr.2003.06.001 15031821

[31] Cuthbert SC, Goodheart GJ. On the reliability and validity of manual muscle testing: a literature review. Chiropractic & osteopathy. 2007;15(1):1–23. doi: 10.1186/1746-1340-15-4 17341308 PMC1847521

[32] Shah S. Reliability of the original Brunnstrom recovery scale following hemiplegia. Australian Occupational Therapy Journal. 1984;31(4):144–51.

[33] Hernández ED, Forero SM, Galeano CP, Barbosa NE, Sunnerhagen KS, Murphy MA. Intra-and interrater reliability of Fugl-Meyer Assessment of Lower Extremity early after stroke. Brazilian Journal of Physical Therapy. 2020. doi: 10.1016/j.bjpt.2020.12.002 33358073 PMC8721065

[34] Alghadir AH, Al-Eisa ES, Anwer S, Sarkar B. Reliability, validity, and responsiveness of three scales for measuring balance in patients with chronic stroke. BMC neurology. 2018;18(1):1–7. 30213258 10.1186/s12883-018-1146-9 PMC6136166

[35] Viosca E, Martínez JL, Almagro PL, Gracia A, González C. Proposal and validation of a new functional ambulation classification scale for clinical use. Archives of physical medicine and rehabilitation. 2005;86(6):1234–8. doi: 10.1016/j.apmr.2004.11.016 15954065

[36] Wilson JL, Hareendran A, Hendry A, Potter J, Bone I, Muir KW. Reliability of the modified Rankin Scale across multiple raters: benefits of a structured interview. Stroke. 2005;36(4):777–81. doi: 10.1161/01.STR.0000157596.13234.95 15718510

[37] Kurtais Y, Küçükdeveci A, Elhan A, Yilmaz A, Kalli T, SONEL TUR B, et al. Psychometric properties of the Rivermead Motor Assessment: its utility in stroke. Journal of Rehabilitation Medicine. 2009;41(13). doi: 10.2340/16501977-0463 19894001

[38] Tsai C, Bayliss MS, Ware JE. SF-36 health survey annotated bibliography: 1996 supplement: Health Assessment Lab, New England Medical Center; 1997.

[39] Han C-W, Lee E-J, Iwaya T, Kataoka H, Kohzuki M. Development of the Korean version of Short-Form 36-Item Health Survey: health related QOL of healthy elderly people and elderly patients in Korea. The Tohoku journal of experimental medicine. 2004;203(3):189–94. doi: 10.1620/tjem.203.189 15240928

[40] Kim T-l Hwang SH, Lee WJ Hwang JW, Cho I Kim E-H, et al. The Korean version of the Fugl-Meyer Assessment: reliability and validity evaluation. Annals of rehabilitation medicine. 2021;45(2):83–98. doi: 10.5535/arm.20225 33849084 PMC8137384

[41] Choi J-H, Jeon S, Hong S, Kim A, Park J-Y, Yang H-J. The reliability and validity of the Korean version of hospital anxiety and depression scale using Rasch measurement theory in patients with Parkinson’s disease. Journal of the Korean Neurological Association. 2021;39(4):312–21.

[42] Jung HY, Park JH, Shim JJ, Kim MJ, Hwang MR, Kim SH. Reliability Test of Korean Version of Berg Balance Scale. Journal of the Korean Academy of Rehabilitation Medicine. 2006;30(6):611–8.

[43] Horn SD, DeJong G, Smout RJ, Gassaway J, James R, Conroy B. Stroke rehabilitation patients, practice, and outcomes: is earlier and more aggressive therapy better? Archives of physical medicine and rehabilitation. 2005;86(12):101–14. 10.1016/j.apmr.2005.09.016 16373145

[44] Veerbeek JM, Koolstra M, Ket JC, van Wegen EE, Kwakkel G. Effects of augmented exercise therapy on outcome of gait and gait-related activities in the first 6 months after stroke: a meta-analysis. Stroke. 2011;42(11):3311–5. doi: 10.1161/STROKEAHA.111.623819 21998062

[45] Foley N, McClure JA, Meyer M, Salter K, Bureau Y, Teasell R. Inpatient rehabilitation following stroke: amount of therapy received and associations with functional recovery. Disability and rehabilitation. 2012;34(25):2132–8. doi: 10.3109/09638288.2012.676145 22524794

[46] Stanton R, Ada L, Dean CM, Preston E. Biofeedback improves performance in lower limb activities more than usual therapy in people following stroke: a systematic review. Journal of physiotherapy. 2017;63(1):11–6. doi: 10.1016/j.jphys.2016.11.006 27989731

[47] Genthe K, Schenck C, Eicholtz S, Zajac-Cox L, Wolf S, Kesar TM. Effects of real-time gait biofeedback on paretic propulsion and gait biomechanics in individuals post-stroke. Topics in stroke rehabilitation. 2018;25(3):186–93. doi: 10.1080/10749357.2018.1436384 29457532 PMC5901660

