# Supplementary material for: Protocol for a single-blind randomized controlled clinical trial to investigate the feasibility and safety of in-bed self-exercises based on electromyography sensor feedback in patients with subacute stroke
Source: PLoS One. 2024 Dec 30;19(12):e0310178. doi: 10.1371/journal.pone.0310178 (PMC11684591; doi:10.1371/journal.pone.0310178)
Supplement: S1 File — (PDF) [file pone.0310178.s003.pdf]

아급성기 뇌졸중 환자에서 근전도 센서 피드백에 기반한 침상내 자가운동의 타당성과 안전성: 전향적, 다기관, 공개, 평가자 눈가림, 탐색적 예비임상시험

Feasibility and safety of in-bed self-exercises based on EMG-sensor feedback in subacute stroke patients: a prospective, multicenter, single-blind, open-label, exploratory pilot clinical trial

Version No: 2.5

Department of Rehabilitation Medicine, Seoul National University Hospital
Woo Hyung Lee

Index

Research Overview 3
Research proposal 6
1. Research Title 6
2. Research organization 6
3. Principal Investigators and Co-Investigators 6
4. Funding Institution 7
5. Expected Research period 7
6. Research subjects 7
7. Research Significance 7
8. Overview of experimental medical devices 9
9. Subject Selection and Recruitment 17
10. Experimental Methods 19
11. Data and Safety Monitoring Plan 38
12. Measures for Participant Safety and Protection 39
13. Refference 42

Research Overview

Research Title	(국문) 아급성기 뇌졸중 환자에서 근전도 센서 피드백에 기반한 침상내 자가운동의 타당성과 안전성: 전향적, 다기관, 공개, 평가자 눈가림, 탐색적 예비임상시험
	(영문) Feasibility and safety of in-bed self-exercises based on EMG-sensor feedback in subacute stroke patients: a prospective, multicenter, single-blind, open-label, exploratory pilot clinical trial
Research Institution	National University Hospital
Principal Investigator	Department of Rehabilitation Medicine, Seoul National University Hospital Clinical Assistant Professor Woo Hyung Lee
Funding Institution	Ministry of Trade, Industry & Energy

Research Objectives	The goal of this to investigate the effect of in-bed self-exercises based on EMG-sensor feedback on functional mobility in subacute stroke patients.
Research type	Investigator Initiated Trials
Research design	Medical Device Clinical Trials (a prospective, multicenter, single-blind, open-label, exploratory pilot clinical trial)
Research period	36 months from the approval date
Research subjects	Subacute stroke patient
Subjects	Experimental group stroke patients 12<br>Control group stroke patients 12
Vulnerable Subject	Adults with impaired consent capacity
Research Description	1. subjects: Adult stroke patients with lower extremity muscle weakness within 3 months of stroke<br>2. randomization: Stratified randomization by age(<65/≥65)and initial mRS(2-3/4-5)<br>(1) Group set<br>・ Experimental group: Conventional lower extremity rehabilitation + self-exercise based on feedback using data obtained via electromyography-supervised.<br>・ Control group: Conventional lower extremity rehabilitation

	(2) Self-exercise<br>・ Based on the developed self-exercise protocol, the patient selects the appropriate type and modality of exercise, performed alone or with the help of a caregiver, while maintaining proper posture.<br>・ The control group will receive Conventional lower extremity rehabilitation only, while the Experimental group will receive supervised self-exercise based on electromyographic feedback.<br>3. Clinical assessment items<br>(1) primary outcome<br>■ Assessment: baseline – 3weeks – 12weeks<br>・ Pittsburgh Rehabilitation Participation Scale (PRPS)<br>(2) secondary outcome<br>■ Assessment: baseline – 3weeks – 12weeks<br>・ The number and percentage of participating sessions<br>・ The number and percentage of completed sessions<br>・ The number and percentage of successful sessions<br>・ The mean amplitude of muscle contractions in a session<br>・ The duration and percentage of participating sessions during self-exercises<br>・ Rivermead motor assessment (RMA): Gross function, leg and trunk<br>・ Manual muscle test (MMT): Hip flexor, Hip abductor, Knee extensor, Ankle dorsiflexor<br>・ brunnstrom stages of motor recovery (BRS)<br>・ Fugl Meyer assessment of lower extremity (FMS-LE)<br>・ Berg balance scale (BBS)<br>・ Functional ambulation category (FAC)<br>・ modified Rankin scale (mRS)<br>・ modified Barthel index (MBI)<br>・ Short-form Health Survery 36 version 2(SF-36v2)<br>4. Assessment items for exercise protocols<br>■ Assessment: 1weeks – 2weeks<br>・ Manual muscle test (MMT): Hip flexor, Hip abductor, Knee extensor, Ankle dorsiflexor<br>・ brunnstrom stages of motor recovery (BRS)<br>・ Pittsburgh Rehabilitation Participation Scale (PRPS)<br>5. Medical device application areas<br>(1) Vastus lateralis<br>(2) Tibialis Anterior

	6. Medical Device Usage<br>(1) the device to the patch and attach it to the targeted muscle<br>(2) Log in to the user app and perform the assigned exercise as instructed.<br>(3) Check muscle activity of target muscles while performing self-guided exercises
Medical Device Information	Myoverse, SMD Solution, Inc.
Assessing safety	1. Any adverse event related to rehabilitation: Falls, fractures, serious pain, hemodynamic instability during exercise, other minor adverse events (dizziness, tachypnea, sinus tachycardia, nervousness, etc.).
Expected Research Outcomes	Increasing exercise in subacute stroke patients can improve their mobility, activities of daily living, and quality of life. This could lead to reduced healthcare and societal costs. If self-exercise based on EMG sensor feedback is effective, it may contribute to the development of related industries.

Research proposal

1. Research Title

Feasibility and safety of in-bed self-exercises based on EMG-sensor feedback in subacute stroke patients: a prospective, multicenter, single-blind, open-label, exploratory pilot clinical trial

2. Research organization

Organ1: Department of Rehabilitation Medicine, Seoul National University Hospital
Address: 101, Daehak-ro, Jongno-gu, Seoul, Republic of Korea
Tel: 02-2072-4178
Fax: 02-6072-5244
Department of Rehabilitation Medicine, Seoul National University Hospital Clinical Assistant Professor Woo Hyung Lee

Organ2: National Traffic Accident Rehabilitation Hospital
Address: 260, Jungang-ro, Yangpyeong-eup, Yangpyeong-gun, Gyeonggi-do, Republic of Korea
Tel: 031-580-5555
Fax: 031-580-5785
Department of Rehabilitation Medicine, National Traffic Accident Rehabilitation Hospital Clinical professor Hyunmi Oh

3. Principal Investigators and Co-Investigators

1) Principal Investigators

Department of Rehabilitation Medicine, Seoul National University Hospital assistant professor Woo Hyung Lee

2) Co-Investigators

Department of Rehabilitation Medicine, Seoul National University Hospital Professor Byung-Mo Oh

Department of Rehabilitation Medicine, National Traffic Accident Rehabilitation Hospital professor Hyunmi Oh

Department of Rehabilitation Medicine, Seoul National University Hospital Associate Professor Han Gil Seo

Department of Rehabilitation Medicine, Seoul National University Hospital assistant professor Sung Eun Hyun

Department of Rehabilitation Medicine, Seoul National University Hospital Researcher So Yeon Jeon

3) **Researchers**

Department of Rehabilitation Medicine, Seoul National University Hospital Research Professor Jung Hyun Kim

4) **Monitoring**

Department of Rehabilitation Medicine, Seoul National University Hospital Research Professor Jung Hyun Kim

Department of Rehabilitation Medicine, National Traffic Accident Rehabilitation Hospital Researcher Sumin Oh

5) **Coordinator**

Department of Rehabilitation Medicine, Seoul National University Hospital Clinical assistant professor Woo Hyung Lee

6) **Medical Device management**

Department of Rehabilitation Medicine, Seoul National University Hospital Research Professor Jung Hyun Kim

Department of Rehabilitation Medicine, National Traffic Accident Rehabilitation Hospital Researcher Sumin Oh

4. Funding Institution

Organ: Ministry of Trade, Industry & Energy

Address: 402, Hannuri-daero, Sejong-si

5. Expected Research period

36 months from the approval date

6. Research subjects

onset of the stroke is less than 3months ago

7. Research Significance

1) Background

Stroke is a leading cause of disability worldwide, and its most common symptoms include muscle weakness and contracture, and impaired motor control, resulting in decreased functional independence in performing daily activities, quality of life, and activities of daily living. Recently, the use of multimodal sensor-based wearable systems to observe and analyze patients' posture and movement for post-stroke motor assessment has received great attention [1-3]. One of them is a system that uses both electromyography and IMU sensors. Electromyography measures muscle activity in the upper and lower extremities, while IMU sensors measure a range of motion and activity.

Conventional rehabilitation exercise for stroke involves a therapist working one-on-one with

the patient to teach them to sit, stand, and walk on their own, with appropriate assistance to help them coordinate their muscles and perform movements on their own. The activation of the appropriate muscles for the task is critical to successful movement, and increasing the activation of reduced muscle activity due to brain injury may be essential to improve the effectiveness of therapy [4-6].

Previous research has reported that wearable sensors can be utilized to quantitatively assess movement and use the information obtained for treatment in order to increase the effectiveness of gait training [7]. However, a limitation of this study is that it was conducted in ambulatory stroke patients, which is a very narrow target population. There is a need to expand the scope of care for patients who require lower extremity rehabilitation due to gait or balance disorders. On the other hand, studies have shown that providing appropriate feedback based on information obtained from the patient in stroke rehabilitation can help improve gait and balance.

Recently, it has been reported that high-intensity of rehabilitation is important for the recovery of mobility function in stroke patients [8]. Although a high dose and intensity of rehabilitation are important for motor function, in practice it is not provided at sufficient intensity and dose [9]. Studies have been published on the effectiveness of self-exercise interventions, and a recent systematic review reported that self-exercise programs have similar therapeutic benefits to Conventional rehabilitation [10]. However, most of the studies reported on upper extremity rehabilitation, and only two studies were published on lower extremity rehabilitation and showed similar effects to Conventional lower extremity rehabilitation [11, 12]. Compared to upper extremity rehabilitation, lower extremity self-exercise has not been well studied.

In previous studies, self-exercise was primarily supervised by a therapist, but recent advances in technology have led to reports of self-exercise programs that utilize telecommunication devices such as smartphones, motion-sensing systems, and computer-based communication tools [13]. However, there is still a lack of research to verify the therapeutic effectiveness and validity of these self-exercise programs. Therefore, we would like to conduct a pilot clinical trial to determine the feasibility and safety of adding lower extremity self-exercise based on EMG sensor feedback.

2) Research Objectives and Hypothesis

(1) Hypothesis

Null hypothesis: No statistically significant difference in Pittsburgh Rehabilitation Participation Scales among subacute stroke patients who received Conventional lower extremity rehabilitation versus those who received based on feedback using data obtained via electromyography-supervised self-exercise.

Alternative hypothesis: a statistically significant difference in Pittsburgh Rehabilitation

Participation Scales among subacute stroke patients who received Conventional lower extremity rehabilitation versus those who received based on feedback using data obtained via electromyography-supervised self-exercise.

(2) Objectives

This study would like to investigate the feasibility and safety of adding lower extremity self-exercise based on EMG sensor feedback to Conventional lower extremity rehabilitation in subacute stroke patients.

8. Overview of experimental medical devices

1) Software as a Medical Device

(1) Part name: Electromyography Analysis Software, Electromyograph (제인 22-4998)

(2) Item Code: E10010.01

(3) Item Class: Class 2

(4) Model name: Myoverse s/w

(5) Manufacturer: SMD Solution, Inc.

(6) Intended use

A self-exercise program used for rehabilitation of stroke patients by analyzing electromyography data measured from electromyography sensors when performing rehabilitation exercises prescribed by a doctor and applying it to rehabilitation.

(7) Indications

Large categories	medium categories	small categories
Diseases of the circulatory system	Cerebrovascular disease	[I60- I69]

Table 1 **Indications Classification**

(8) Usage Time

Use point: Bed rest time

Time and Frequency: 30min,1-2 sessions per day

2) Application Principle

(1) Electromyography analysis software

The medical staff enters the exercises required for each patient's rehabilitation treatment into the medical staff app. The patient attaches an EMG sensor to the body for rehabilitation, enters the patient's name, and performs the prescribed rehabilitation exercises. Patients can view real-time electromyographic signals during the exercises and see the results of their exercises performance (time, reps, accuracy, etc.) after completion.

1 Acquiring and analyzing electromyographic data

A. Electrodes attached to the surface of the skin amplify potential differences to obtain electrical signals from muscles.

B. View the analysis results of RMS, MVC, performance rate, goal attainment rate, and fall risk.

2 Measuring the accuracy of rehabilitation exercises

A. The medical staff app measures the patient's MVC and the provider sets a target MVC for the patient.

B. Accuracy reflects the percentage of achievement against the target value, from 50% or more of the set target value to 100%.

C. Measurements below 50% are logged and considered not performed.

3 Real-time feedback

A. The medical staff app and patient app can each monitor signals measured during exercise with biofeedback, but the apps cannot monitor each other in real time.

4 Adjust the software's own difficulty level

A. Medical staff apps can set the difficulty level (number of times, duration, MVC value) on the program settings page.

B. Patient apps cannot set difficulty levels

7 Transferring patient data

A. Patient sends data to the server when they click the Finish button

B. Performance Rate, Goal Attainment Rate

C. Attach sensors to locations such as the vastus lateralis and tibialis anterior and acquire and transmit electromyographic signal data during exercise performance.

3) Shape and Structure

(1) Electromyography analysis program

1 Medical staff apps

A. Patient Registration

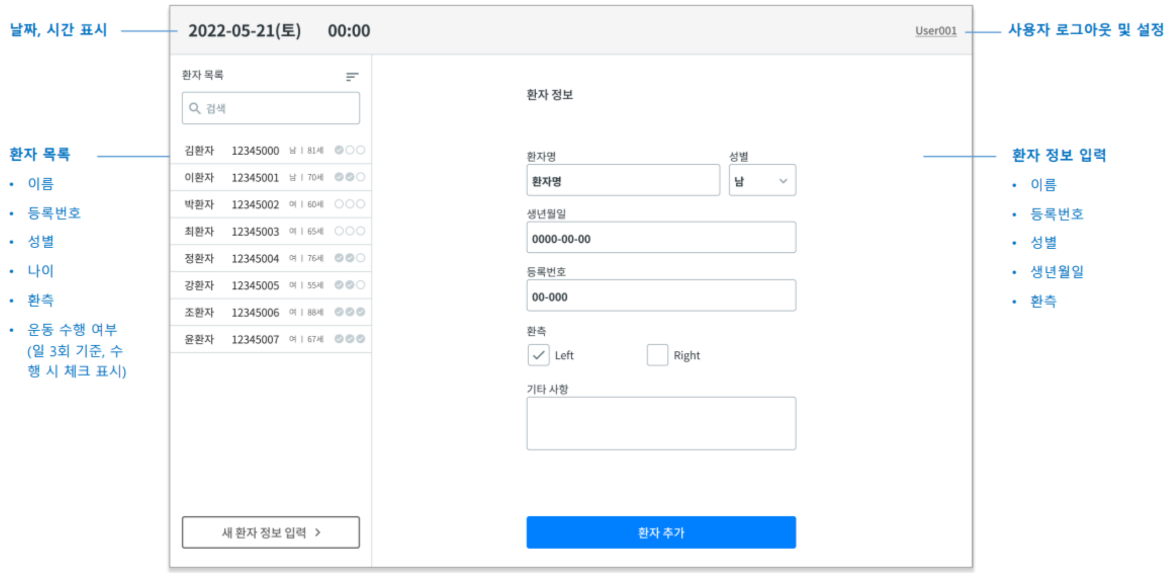

Figure 1 Patient registration screen

B. Connect the Patch

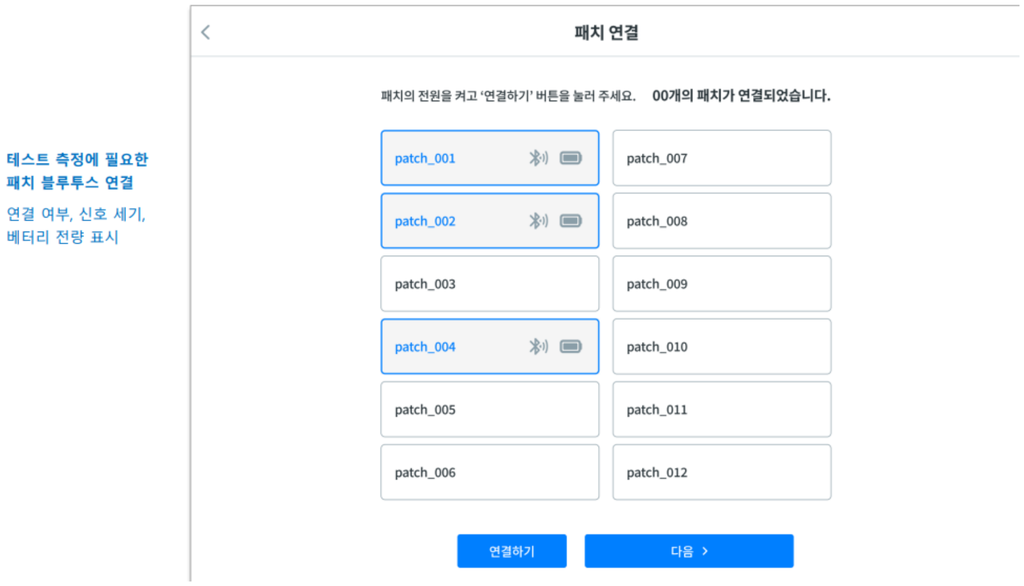

Figure 2 Patch Registration Screen

C. Select Behavior and Patch Attachment Location

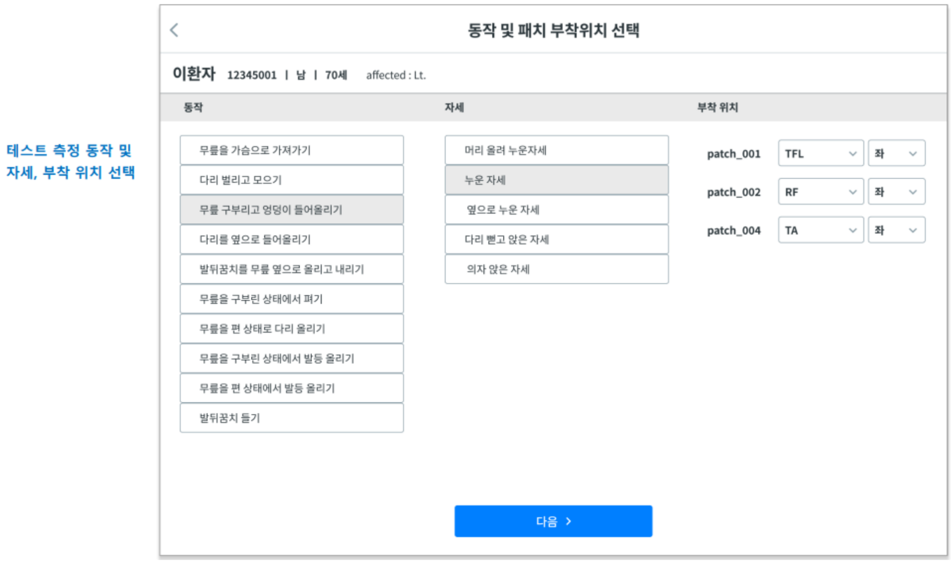

Figure 3 Select Action and Patch Attachment Location Screen

D. Check the analysis results after measuring the signal

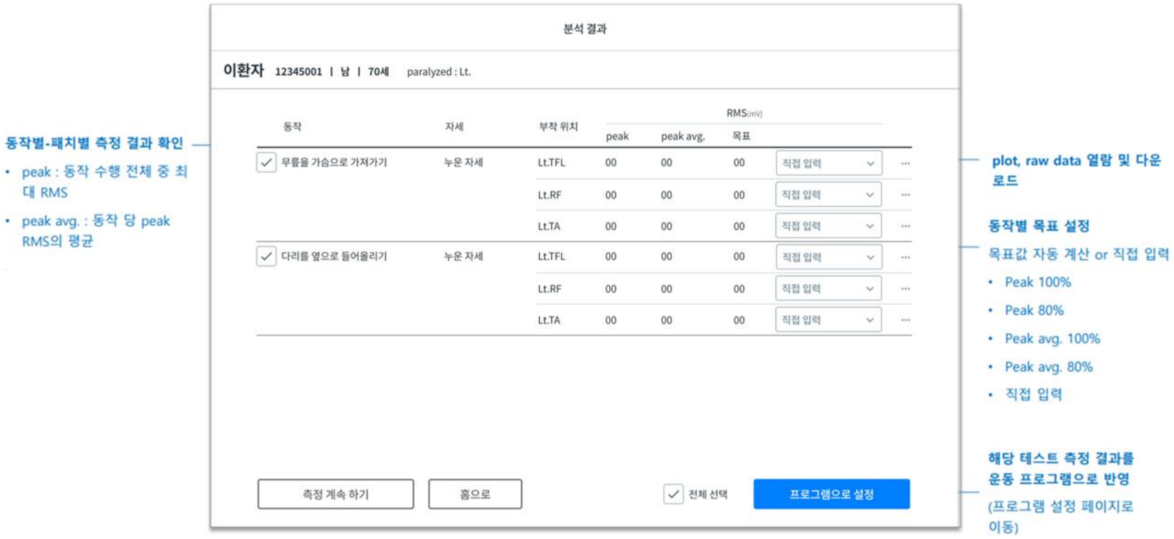

Figure 4 Analysis result screen after signal measurement

E. self-exercise prescriptions

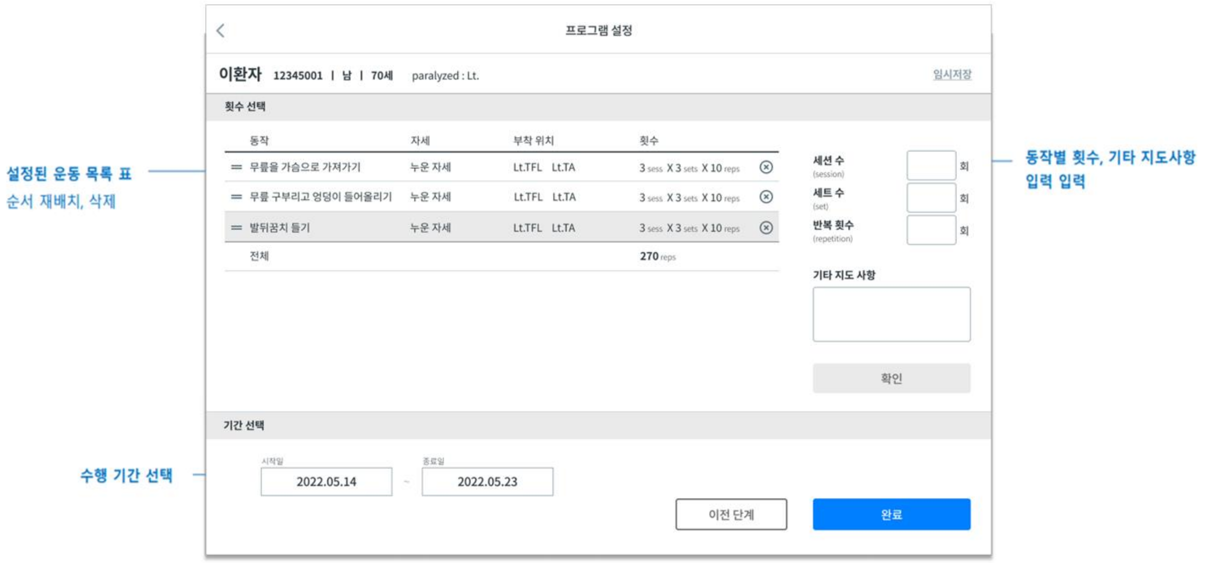

Figure 5 Self-Exercise Prescription Screen

F. View the analysis results

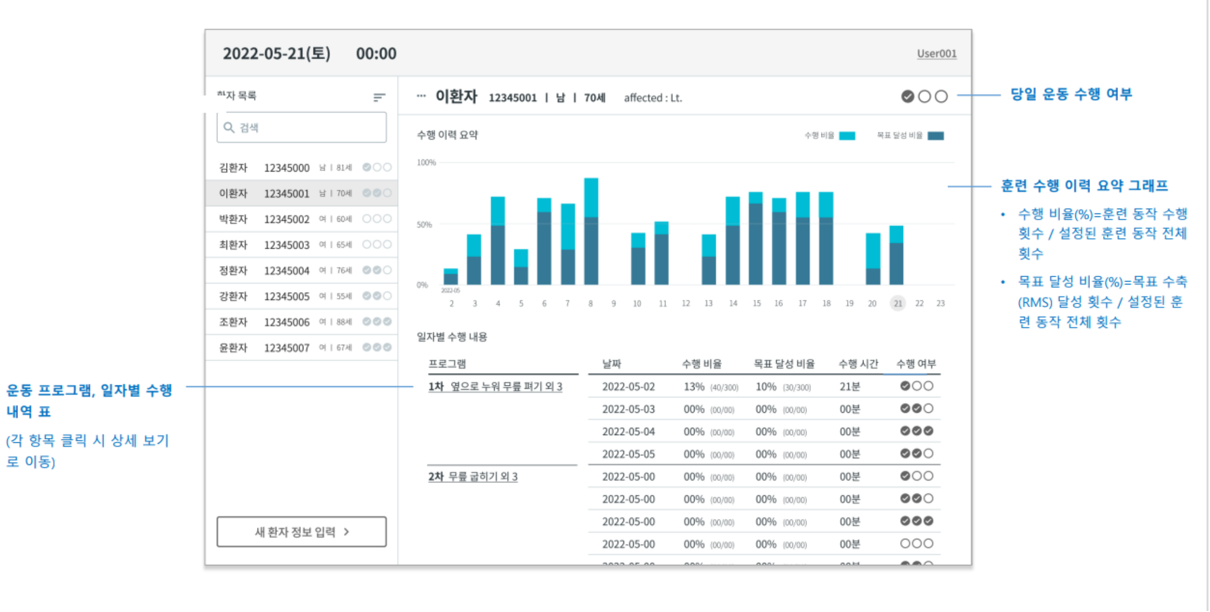

Figure 6 Analysis Results Screen

2 Patient apps

A. Provider logs in with ID, password, and selects patient and device to match patient and device

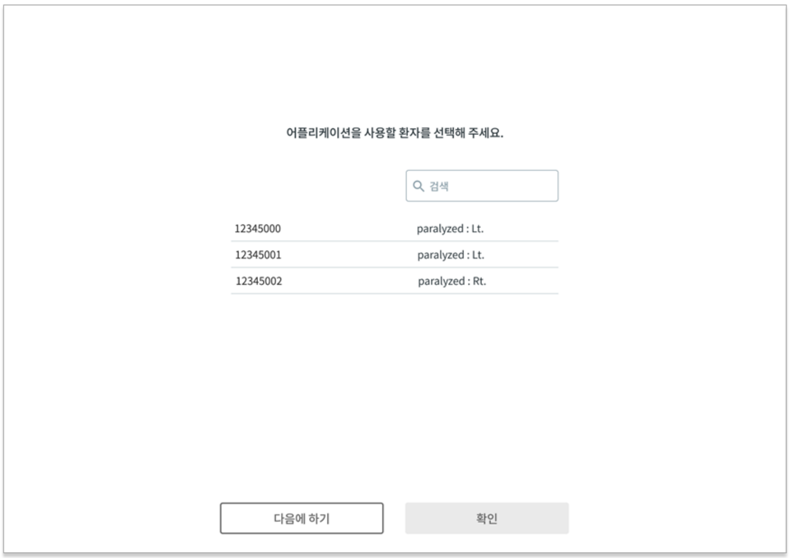

Figure 7 Login screen

B. Show Exercises to Perform

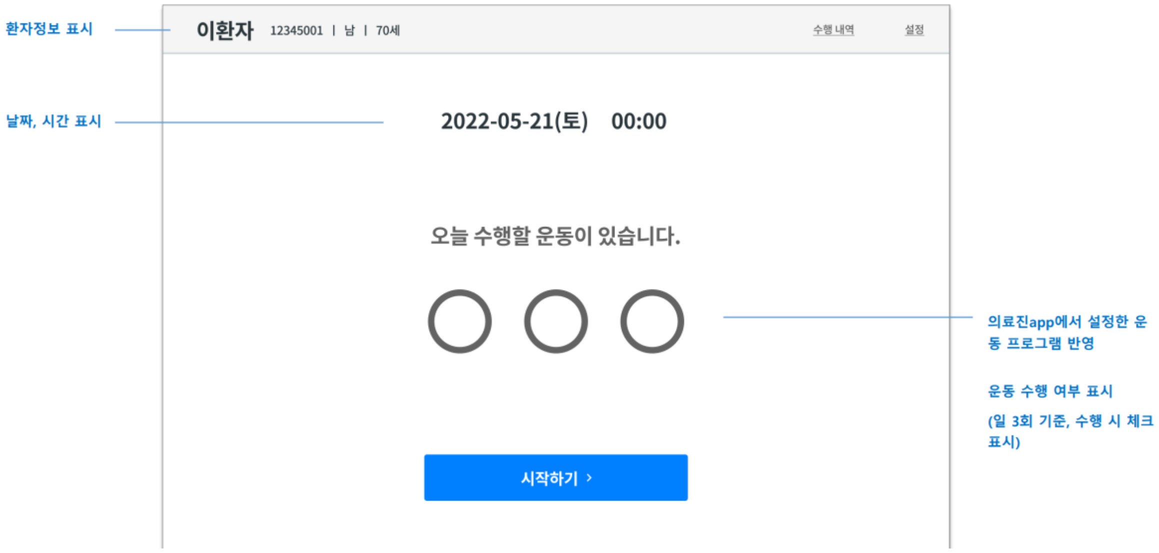

Figure 8 Exercise Display

C. Attaching and Connecting Devices

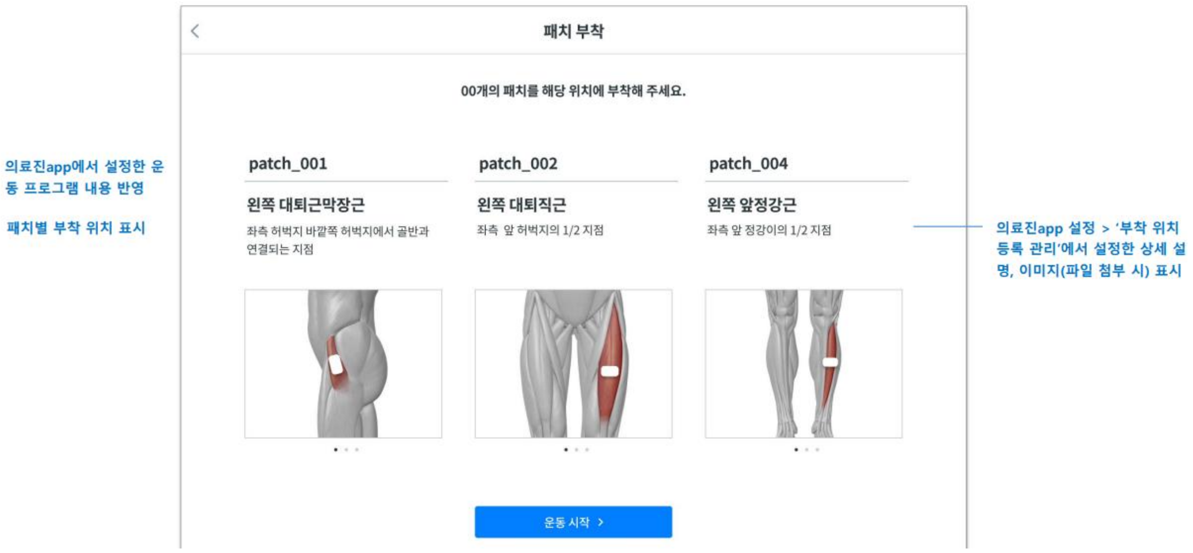

Figure 9 Attaching and Connecting Devices Screen

D. Performing self-exercises

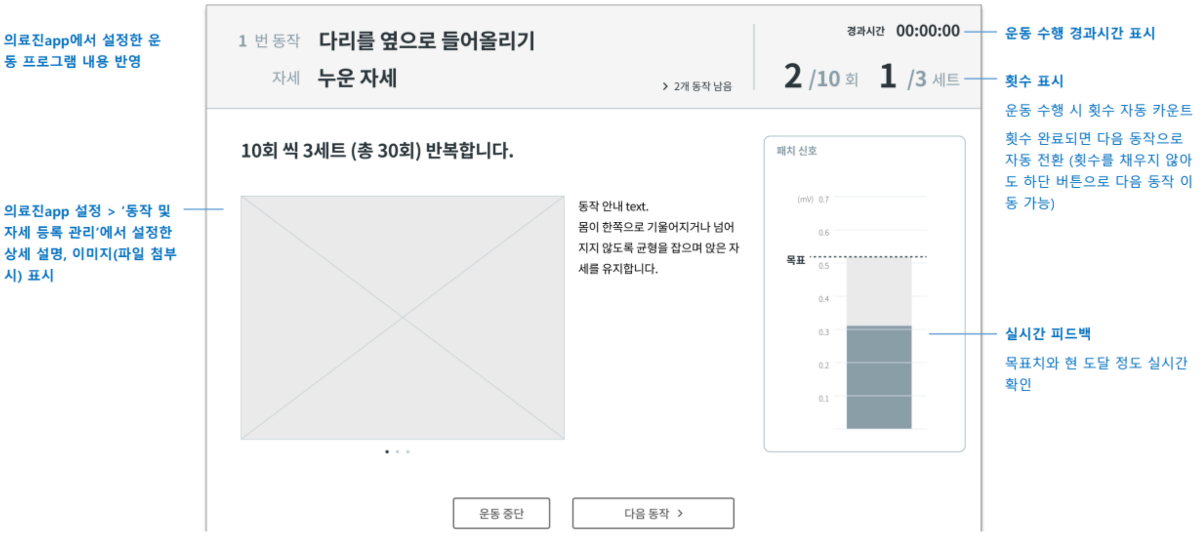

Figure 10 Self-exercise performance screen

E. Complete the Self-Exercise

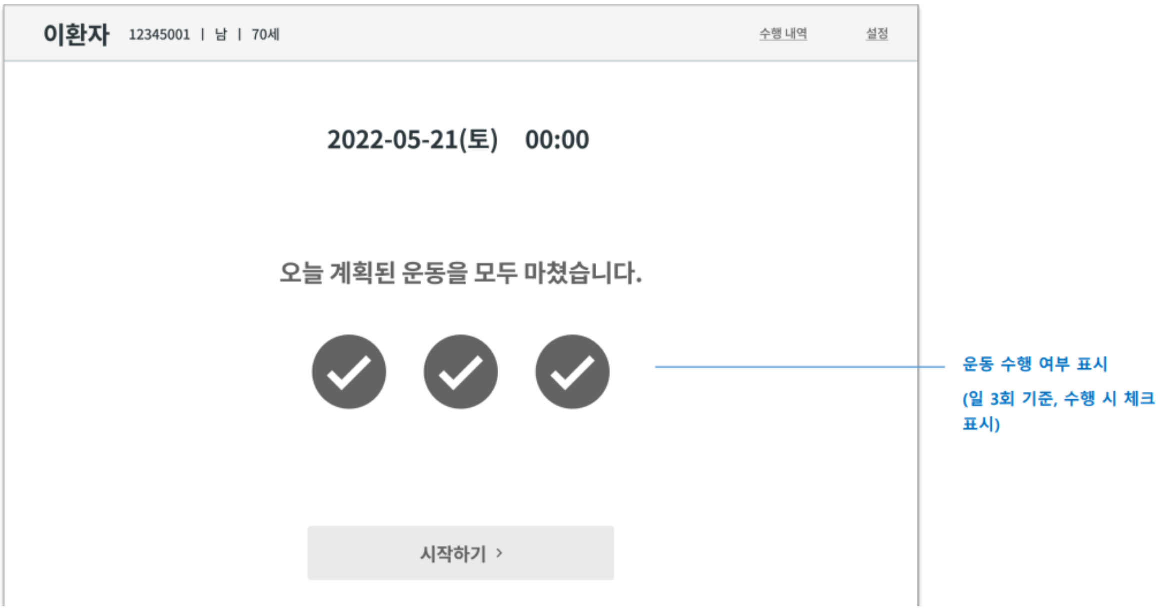

Figure 11 Self-Exercise Completion Screen

4) Performance

(1) Electromyography Analysis Program

1 Medical Staff App

A. Patient registration

B. List display of patients

C. Program setting (select exercise program, set number of repetitions, number of sets, how many times a day (session))

D. Select sensor attachment location, set left and right and target value

E. Testing (real-time signals, target value measurement, position, exercise availability, etc.)

F. Patient monitoring

i. Check exercise performance details (date/performance rate/goal achievement rate/performance time/performance status)

ii. Performance ratio (number of exercises performed / number of sets * repetitions of the program)

iii. Goal achievement rate (number of times the goal is achieved / set of the program * number of repetitions)

2 Patient app

A. Physician selects an ongoing patient from the patient list

B. Press the Get Started button, place the patch on the attachment location, and connect the device and patch.

C. Press the Start Exercise button and the rehabilitation will appear.

D. Perform the prescribed exercises and click the Done button when finished..

E. Click Performance in the upper right corner of the home screen to check the contents.

i. Date/performance rate/goal attainment rate/performance time/performance status

ii. Performance ratio (number of exercises performed / sets * reps)

iii. Goal attainment rate (number of times goal was achieved / sets * reps in the program)

9. Subject Selection and Recruitment

Participants must meet all of the inclusion criteria below to be eligible to participate in the study, but not any of the exclusion criteria.

1) Inclusion criteria

1 19 years and older

2 the onset of the stroke is less than 3months ago

3 Lower extremity weakness due to stroke (MMT =< 4 grade)

4 Modified Rankine Scale 2-5 points

5 Cognitive ability to follow commands

2) Exclusion criteria

1 stroke recurrence

2 other neurological abnormalities (e.g. Parkinson's disease).

3 severely impaired cognition

4 serious and complex medical conditions(e.g. active cancer)

5 cardiac pacemaker or other implanted electronic system

3) Sample size

Subject: 24 stroke patients

산출 근거: As this study is a pilot study for a future clinical trial, we plan to recruit a minimum number of subjects that will allow us to compare the difference in signal modality with the control group.

4) Recruitment

The study will be competitively recruited by printing a notice of recruitment in the form of

a poster on the bulletin boards of the Department of Rehabilitation, Seoul National University Hospital and Department of Rehabilitation National Transportation Rehabilitation Hospital, and promoting it to inpatients. The principal investigator and the sponsoring organization will not exclude patients who may be eligible for this study on the basis of race or socioeconomic status. Every effort will be made to recruit possible patients to participate in this study if they meet the inclusion criteria, and participants will be informed of the purpose of the study.

10. Experimental Methods

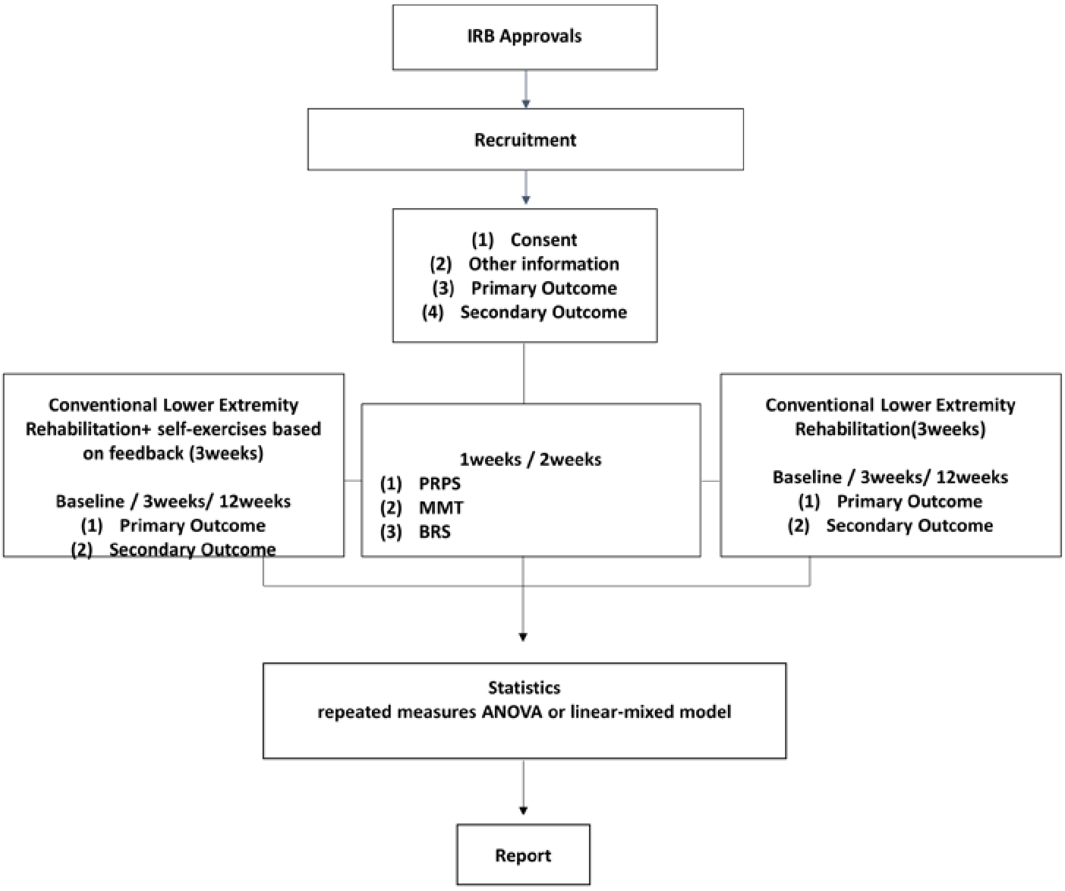

Figure 12 Flow chart

1) Experimental Approaches

(1) Consent

A. Volunteers in the study were explained the purpose of the study and the use of their data, completed a study consent form and pre-screening, and were assigned a subject identification code (site code - year - study serial number - order of enrollment).

(2) Clinical Assessment

1 Primary Outcome

A. Pittsburgh Rehabilitation Participation Scale (PRPS)

The PRPS is a clinician-assessed instrument designed to assess patient participation in treatment. It is rated on a scale of 1 to 6 to measure the patient's effort and activeness to participate in treatment. Score '6' if the patient participated in all exercises and ' 1' if the patient refused the entire session. [21].

2 Secondary Outcome

A. The number and percentage of participating sessions

A participating sessions is defined as a session in which the number of muscle contraction is more than one and the amplitude of the one or more targeted muscles is more than 20% of MVIC in each muscle contraction.

B. The number and percentage of completed sessions

A completed session is defined as a session in which the percentage of targeted muscle contractions is more than 90% of total muscle contractions and the amplitude of the one or more targeted muscles is more than 20% of MVIC in each muscle contraction.

C. The number and percentage of successful sessions

A successful session is defined as a session in which the percentage of targeted muscle contractions is more than 90% of total muscle contractions and the amplitude of the one or more targeted muscle is more than 50% of MVIC in each muscle contraction.

D. The mean amplitude of muscle contractions in a session

Average change in peak amplitude of muscle contractions in self-exercise performed by the subject

E. The duration and percentage of participating sessions during self-exercises

Participation time and ratio of self-exercise performed by the subject

F. Rivermead motor assessment (RMA):

The RMA is scored by the therapist through an interview with the patient and caregiver [14]. The RMA consists of three scales, including gross function, leg and trunk, and arm sections. Each activity must be carried out independently. Allowing his three attempts for each item. The Gross Function section (RMA-gf) consists of 13 items and primarily assesses mobility from sitting to running and gait. The leg and trunk section (RMA-lt) describes individual movements of the trunk (e.g. rolling to the affected side) and leg (e.g. ankle dorsiflexion with the leg extended while lying down). It consists of 10 items to be evaluated. Score '1' if the patient can perform the activity and ' 0' if he cannot. The maximum score equals the number of items. The higher the score, the more normal motor performance is possible [15].

G. Manual muscle test(MMT): As a muscle strength measurement test, it is a scale evaluated on a scale of 0-5 according to strength against gravity and resistance. Score '5' if the patient is of Normal strength, ' 0' if he cannot check contraction palpable. In this study, bilateral hip flexion, hip abduction, knee extension, and ankle dorsiflexion muscle strength were measured. MMT had good external and internal efficacy and

was not dependent on examiner bias.

H. brunnstrom stages of motor recovery (BRS)

The Brunnstrom approach is a classification method that models the motor recovery process after stroke-induced hemiplegia on a six-point ordinal scale. Brunnstrom's stage of recovery covers the progression of complete motor recovery from stage 1 of complete flaccidity and no voluntary movement to stage 6, when spasticity disappears, and near-normal isolated joint activities become possible. The Brunnstrom approach focuses on unique patterns associated with stroke recovery, including motor spasticity development, synergistic patterns, and voluntary movements. Brunnstrom had high inter-rater reliability (0.74 to 0.98).

I. Fugl-Meyer assessment of lower extremity (FMS-LE)

The FMA-LE investigates hip, knee, and ankle movements, and hierarchical recovery is recorded based on Brunnstrom's stages of recovery, from reflex to synergistic and non-synergistic movements. The FMA-LE motor domain uses a 3-point ordinal scale as follows: 0, unable to perform; 1, partially performance; and 2, complete performance [16].

J. Berg balance scale (BBS)

The BBS was developed in 1989 by Katherine Berg in three-step survey of 32 health professionals to objectively measure balance and fall risk in community-dwelling older adults. The BBS examines 14 movements of daily life on a 5-point ordinal scale (range 0-4). A score of 0 indicates the lowest level of functioning and a score of 4 indicates normal performance. The total score range is 0-56 [17].

K. Functional ambulation category (FAC)

The 6-point rating scale assesses how much human support is required when walking (with or without a personal assistive device).A score of 0 indicates a non-functional ambulator, and scores of 1-3 indicate dependent ambulators. A score of 1 indicates the need for ontinuous manual contact, a score of 2 indicates intermittent or continuous light touch, and a score of 3 indicates supervision or verbal cueing. Scores of 4-5 are independent ambulators, with a score of 4 indicating independent ambulators on horizontal surfaces only and a score of 5 indicating independent ambulators on any surface, including stairs [18].

L. modified Rankin scale (mRS)

mRS is a widely used tool to measure global disability after stroke. The scale classifies

disability from 0 (no symptoms at all) to 5 (severe disability). Scoring is performed by the evaluator based on the subject's functional dependence. The mRS is also found to be a psychometrically accepted measure [19].

M. modified Barthel index (MBI)

The MBI measures activities of daily living and includes ten activity domains, including bowel management, urinary management, grooming, toilet use, eating, locomotion, walking, dressing, climbing stairs, and bathing. Each activity is given a score ranging from 0 (unable to perform a task) to a maximum of 5, 10, or 15 (fully independent- the exact score depends on the evaluated activity). A total score is obtained by summing points for each of the items. Total scores may range from 0 to 100, with higher scores indicating greater independence [20].

N. Short-form Health Survery 36 version 2 (SF-36v2)

The SF-36v2 is a well-studied, self-reported measure of functional health. Each item includes physical functioning, physical role limitation, pain, general medical health, vitality, social functioning, emotional role limitation, mental health, physical component scale, and mental component scale. Scores from 0 to 100 can be obtained through the SF-36v2 scoring software. Higher scores indicate better health status [22].

(3) Sensor

1 Patch attachment site

A. Vastus lateralis – The muscle is most palpable at about the 1/2 point when a line is drawn from the patella of the knee to the anterior superior iliac spine.

B. tibialis anterior – The most palpable point of the muscle is about 1/3 of the way from the fibular head to the medial malleolus.

2 Sensor Signal Parameters

Amplitude (mV) of the electromyographic signal for each muscle, and the ratio of the amplitude at maximal voluntary contraction to the amplitude at actual contraction (%).

3 Exercise

A number of muscle contractions, the ratio of muscle contractions to target number, the number and ratio of electromyographic signal amplitudes reaching a target, time spent performing the exercise, whether it was performed, etc.

(4) Other information

1 Clinical information

A. Demographics: age, gender, dominant hand, medical complication (hypertension,

diabetes, hyperlipidemia, atrial fibrillation, Charlson comorbidity index), education (elementary/middle/high school graduate), caregiver (spouse/children/caregiver/other

B. Stroke-related information: initial NIH Stroke Scale (National Institutes of Health Stroke, NIHSS), stroke treatments (thrombolysis/ thrombectomy), duration since stroke onset, stroke type (infarct/hemorrhage), stroke location (right/left, supratentorial/subtentorial, motor area cortex (M1) involvement/internal capsule involvement), cause of strokes (TOAST classification: Large-arteryAtherosclerosis/ Small-vessel occlusion/ Cardioembolism/ Other determined etiology/ Un-determined etiology).

2 Medications: anticonvulsants, antidepressants, anticonvulsants, etc.

3 Cognitive function: Mini-Mental State Examination (MMSE)

4 Depression and anxiety: Hostpial Anxiety and Depression Scale (HADs)

5 Orthoses: knee stabilizer, ankle-foot orthosis

6 Motor Evoked Potential (MEP)

(5) Treatment protocols

1 Conventional Lower Extremity Rehabilitation

A. Description: General rehabilitation for lower extremity motor deficits in stroke patients and is primarily based on functional mobility with joint range of motion exercises. It includes lying down, sitting up, standing, walking, and balance training and is performed either with the assistance of a therapist or with the use of mobility aids (e.g., walker, cane, lower limb orthosis), depending on the patient's condition. 30 minutes per session, twice a day, 5 days a week for 3 weeks.

B. Step Control: Adjusts the type, intensity, and speed of exercises at the recommendation of the therapist to ensure that an appropriate level of exercise is provided for stroke patients with reduced motor function.

2 Self-exercise

A. Description

i. A stroke patient performs exercises for the lower extremities in bed on their own or with the help of a caregiver.

ii. The Self-exercise group performs supervised self-exercises based on feedback utilizing data obtained through electromyography.

iii. Exercise types consist of a variety of exercises based on functional mobility that can be performed in bed, and how to perform each exercise is described below.

A. Knee to chest
B. Legs together and apart
C. Knee extension from a position of flexion
D. Ankle dorsiflexion with knee flexion
E. Hip bridge
F. Knee flexion while hip flexion, knee extension while hip extension
G. Ankle dorsiflexion while Knee to chest
H. Heel up with knee extension
I. Leg raise to the side with the knee extension
J. Leg raise with the knee extension
K. Knee flexion and extension with hip extension
L. Ankle dorsiflexion with knee extension
M. Heel up with knee flexion
N. walking in place

B. exercise protocols

A. principle

i. The doctor/therapist determines the level and step of stroke patients according to muscle strength and B-stage.
ii. Exercise prescription is prescribed according to the level and step by checking the muscle strength and B-stage by the doctor/therapist every week.
iii. Up to four types of exercise can be selected during one week, and exercises corresponding to the highest possible level are recommended, which are determined by the doctor/therapists considering the patient's condition.
iv. If levels or steps overlap depending on the patient's condition, an exercise corresponding to the highest possible level is recommended, which is determined by the doctor/therapist considering the patient's condition.
v. Doctors/therapists determine the safe and effective posture for exercise considering the patient's condition, and a posture with high functional mobility is recommended.
vi. Doctors/therapists must provide patients with target numerical information about the type of exercise, session (/day), set (/session), number of repetitions (/set), and amplitude of the EMG signal during muscle contraction. In general, exercise 2 ways/session, 2-3 sessions/day, 3-5 sets/session, 5-10 times/, the

amplitude is recommended to be more than 50% of the maximum contraction amplitude.

vii. It is recommended to perform trunk balance exercise for 10 minutes as a warm-up before lower extremity exercise.

viii. EMG sensors are used in a maximum of two or fewer, and it is recommended that a doctor/therapist indicate the attachment location

B. Contents

i. Classification

Levels and Steps are determined based on the Brunstrom scale and lower extremity motor power. There are a total of 3 Levels, and each Level is divided into 3 Steps.

Level	Step	LE Motor Power			Brunnstrom stage - LE
		HF	KE	ADF	
1 (Flaccidity)	A	≤2	≤1	≤1	1-2
	B	≤2	≤2	≤1	
	C	≤2	≤2	≤2	
2 (Within synergy)	A	≥2			2-3
	B		≥2		
	C			≥2	
3 (Out-of synergy)	A	≥3			3-6
	B		≥3		
	C			≥3	

Table 2 Level & Step Classification

Level	Step	LE Motor Power			Brunnstrom stage - LE	Exercise													
		HF	KE	ADF		1. Knee to chest (HF: Ant/Lat thigh)	2. Legs together and apart(Abd & Add: Ant/Lat thigh)	3. Knee extension from a position of flexion(KE: Ant/Lat thigh)	4. Ankle dorsiflexion with knee flexion(ADF: Ant lower leg)	5. Hip bridge (HE: Lat thigh)	6. Knee flexion while hip flexion, knee extension while hip extension (KE: Ant/Lat thigh)	7. Ankle dorsiflexion while Knee to chest(ADF: Ant lower leg)	8. Heel up with knee extension(APF: Ant lower leg)	9. Leg raise to the side with the knee extension (Abd: Ant/Lat thigh)	10. Leg raise with the knee extension(HF: Ant/Lat thigh)	11. Knee flexion and extension with hip extension(KE: Ant/Lat thigh)	12. Ankle dorsiflexion with knee extension (ADF: Ant lower leg)	13. Heel up with knee flexion(APF: Ant lower leg)	14. walking in place(ADF & APF: Ant lower leg)
1 (Flaccidity)	A	≤2	≤1	≤1	1-2	O	O												
	B	≤2	≤2	≤1		O	O	O											
	C	≤2	≤2	≤2		O	O	O	O										
2 (Within synergy)	A	≥2			2-3	O	O	O	O	O									
	B		≥2				O		O	O	O								
	C			≥2			O			O	O	O	O						
3 (Out-of synergy)	A	≥3			3-6		O			O		O	O	O	O				
	B		≥3				O			O				O	O	O			
	C			≥3			O			O				O	O	O	O	O	O

Table 3 Classification exercise

ii. Position

No.	Body Position
1	Half-lying Position
2	Supine Position
3	Side-lying Position
3-1	- Right
3-2	- Left
4	Long Sitting Position
5	Sitting Position

Table 4 Position

No.	Exercise	Postural setting				
		Half-lying	Supine	Side-lying	Long-Sitting	Sitting
1	Knee to chest	O	O	O	O	O
2	Legs together and apart	O	O	X	O	O
3	Knee extension from a position of flexion	O	O	O	O	O
4	Ankle dorsiflexion with knee flexion	O	O	O	O	O
5	Hip bridge	O	O	X	X	X
6	Knee flexion while hip flexion, knee extension while hip extension	O	O	O	O	O
7	Ankle dorsiflexion while Knee to chest	O	O	O	O	O
8	Heel up with knee extension	O	O	X	O	O
9	Leg raise to the side with the knee extension	X	X	O	X	X
10	Leg raise with the knee extension	O	O	X	O	O
11	Knee flexion and extension with hip extension	X	X	O	X	X
12	Ankle dorsiflexion with knee extension	O	O	O	O	O
13	Heel up with knee flexion	O	O	O	O	O
14	walking in place	O	O	X	O	O

Table 5 Position & Exercise

C. Treatment Duration

i. Increase usage gradually based on the patient's condition.

ii. Once daily for days 1-2 of participation, then twice daily.

iii. Perform at least 30 minutes per session, at least 2 sessions per day, 5 days per week for 3 weeks.

iv. Total self-exercise time: 60 minutes

v. If the participant does not experience any significant adverse events during the self-exercise, increase the number of sessions; if significant adverse events occur,

decrease the number of sessions in consultation with a healthcare provider.

D. Feedback
   i. Information on muscle contraction activation: When performing an exercise, sensors attached to the muscles you want to contract can quantitatively check the degree of muscle contraction and provide information on muscle contraction and degree.
   ii. information on the number of muscle contractions and exercise duration per session: Check the density of a participant's exercise to provide quantitative information about the amount of exercise, which can be used to check adherence to self-exercise.
   iii. Once a week, the researcher will check the results of the exercise performance to select and explain the self-exercise program.

2) Control Group and Randomization

1 Control group: Conventional lower extremity rehabilitation

2 Experimental group: Conventional lower extremity rehabilitation + self-exercise based on feedback using data obtained via electromyography-supervised.

3 Randomization

A. All subjects will be assigned to the control and test arms in a 1:1 ratio. Because this is an open-label study and single-blind, participants will be able to determine whether they have been assigned to the control or Experimental group, but the evaluator who will conduct the assessment will be blinded to the group assignment information.

B. stratified randomization
   i. Age (<65/≥65), initial mRS (2-3/4-5)

C. Randomization method
   i. Create a web randomization program by commissioning the Seoul National University Medical Research Collaborating Center.
   ii. screening number, gender, date of consent, date of birth, inclusion criteria, and exclusion criteria are entered by the randomizer at each site
   iii. stratified randomization by the program.

3) Clinical Assessment Items

(1) clinical Assessment

1 primary outcome: 3 trials (baseline - 3 weeks - 12 weeks)

A. Pittsburgh Rehabilitation Participation Scale (PRPS)

2 secondary outcome: 3 trials (baseline - 3 weeks - 12 weeks)

A. The number and percentage of participating sessions

B. The number and percentage of completed sessions

C. The number and percentage of successful sessions

D. The mean amplitude of muscle contractions in a session

E. The duration and percentage of participating sessions during self-exercises

F. Rivermead motor assessment (RMA): Gross function, leg and trunk

G. Manual muscle test (MMT): Hip flexor, Hip abductor, Knee extensor, Ankle dorsiflexor

H. brunnstrom stages of motor recovery (BRS)

I. Fugl Meyer assessment of lower extremity (FMS-LE)

J. Berg balance scale (BBS)

K. Functional ambulation category (FAC)

L. modified Rankin scale (mRS)

M. modified Barthel index (MBI)

N. Short-form Health Survery 36 version 2(SF-36v2)

(2) Assessment for exercise protocols

1 Evaluation: (1 week - 2 week)

A. Pittsburgh Rehabilitation Participation Scale (PRPS)

B. Manual muscle test (MMT: Hip flexor, Hip abductor, Knee extensor, Ankle dorsiflexor

C. brunnstrom stages of motor recovery (BRS)

(3) Characteristic information

1 Clinical information

A. Demographics: age, gender, dominant hand, medical complication (hypertension, diabetes, hyperlipidemia, atrial fibrillation, Charlson comorbidity index), education (elementary/middle/high school graduate), caregiver (spouse/children/caregiver/other

B. Stroke-related information: initial NIH Stroke Scale (National Institutes of Health Stroke, NIHSS), stroke treatments (thrombolysis/ thrombectomy), duration since stroke onset, stroke type (infarct/hemorrhage), stroke location (right/left, supratentorial/subtentorial, motor area cortex (M1) involvement/internal capsule involvement), cause of strokes (TOAST classification: Large-arteryAtherosclerosis/ Small-vessel occlusion/ Cardioembolism/ Other determined etiology/ Un-determined etiology).

2 Medications: anticonvulsants, antidepressants, anticonvulsants, etc.

3 Cognitive function: Mini-Mental State Examination (MMSE)

4 Depression and anxiety: Hostpial Anxiety and Depression Scale (HADs)

5 Orthoses: knee stabilizer, ankle-foot orthosis

6 Motor Evoked Potential (MEP)

(4) Assessment point

1 Screen

Screen Test, MMT, mRS, Clinical information, Medications, Cognitive function, Depression and anxiety, Orthoses, MEP

2 Baseline

PRPS, The number and percentage of participating sessions, The number and percentage of completed sessions, The number and percentage of successful sessions, The mean amplitude of muscle contractions in a session, The duration and percentage of participating sessions during self-exercises, RMA, FAC, mRS, BBS, MMT, BRS, MAS, FMS-LE, MBI, SF-36v2

3 3 weeks

PRPS, The number and percentage of participating sessions, The number and percentage of completed sessions, The number and percentage of successful sessions, The mean amplitude of muscle contractions in a session, The duration and percentage of participating sessions during self-exercises, RMA, FAC, mRS, BBS, MMT, BRS, MAS, FMS-LE, MBI, SF-36v2

4 12 weeks

RMA, FAC, mRS, BBS, MMT, BRS, MAS, FMS-LE, MBI, SF-36v2

5 1-week and 2-week tests for exercise protocol implementation

PRPS, MMT, BRS

Time point			Screening	Enrollment	Allocation	Baseline	3weeks	12weeks
Enrollment	(-)	Eligibility screening (MMT/mRS)	X					
	(-)	Informed consent		X				
	(-)	Randomization			X			
	(-)	demographic, neurological characteristics	X					
Intervention	Control group	Conventional PT				X	X	
	Training group	PT + self exercise base EMG				X	X	
Assessment	Primary outcome	PRPS				←→		
	Secondary outcome	The number and percentage of participating sessions				←→		
		The number and percentage of completed sessions				←→		
		The number and percentage of successful sessions				←→		
		The mean amplitude of muscle contractions in a session				←→		
		The duration and percentage of participating sessions during self-exercises				←→		
		RMA				X	X	X
		MAS (hip ext, add, knee flex, ankle pantar flex)				X	X	X
		MMT (hip ext, add, knee flex, ankle pantar flex)	X			←→		X
		BRS				←→		X
		mRS	X			X	X	X
		FMS-LE				X	X	X
		FAC				X	X	X
		BBS				X	X	X
		MBI				X	X	X
		SF-36v2				X	X	X
Safety	Adverse event	fall, fracture, pain				←→		X

Table 6 Assessment point

Primary Outcome	Participation	PRPS	Researcher-B
Secondary Outcome	Feasibility	The number and percentage of participating sessions	Researcher-B
		The number and percentage of completed sessions	Researcher-B
		The number and percentage of successful sessions	Researcher-B
		The mean amplitude of muscle contractions in a session	Researcher-B
		The duration and percentage of participating sessions during self-exercises	Researcher-B
	disability	MMT(hip ext, add, knee flex, ankle pantar flex)	Researcher-B
		BRS	Researcher-B
		MAS(hip ext, add, knee flex, ankle pantar flex)	Researcher-B
		FMS-LE	Researcher-B
	Fuction(Gait)	RMA	Researchers-B
		mRS	Researcher-B
		FAC	Researcher-B
		BBS	Researcher-B
	Activities of daily living	MBI	OT
	Quality of life	SF-36v2	Researcher-B
	Cognition	MMSE	OT
	Medison	Depression/Spasticity/Epilepsy	Researcher-B
	Orthotics	Orthotics / Walking aids	Researcher-B
	Neurophysiologic	MEP	MT
	Participation	PRPS	Researcher-B
Safety	Adverse event	fall, fracture, pain	Researcher-B

Table 7 Evaluators

4) Differences

1 Perform self-exercises in addition to Conventional lower extremity rehabilitation

A. Rehabilitation dose is one of the important factors for exercise recovery.

B. Since only two rehabilitation sessions per day are covered by the National Health Insurance Service in Korea, self-exercise is being discussed as a way to increase the amount of rehabilitation.

2 Acquisition and utilization of data through electromyography

A. There are clear limitations to the ability of patients to perform effective self-exercise on their own or with the help of a caregiver by selecting appropriate exercise types, modalities, postures, etc.

B. Supervised self-movement based on feedback using EMG sensors is expected to overcome limitations to the ability of patients to perform effective self-exercise.

5) Benefits and Risks for Study Participants

1 Previous studies have reported that self-exercise is effective in restoring motor function and improving quality of life. Furthermore, the type and modality of supervised self-exercise based on electromyographic feedback is expected to facilitate the recovery of motor function and further improve functional mobility.

2 Expected Risks for Study Participants

The expected risks for study participants participating in this clinical trial are as follows:

- fall, fracture, severe pain, endotracheal tube removal, arterial line removal, central line removal, Adverse events that occurred during exercise included hemodynamic instability such as arrhythmia, bradycardia, systolic blood pressure > 200mmHg, and systolic blood pressure < 90mmHg, as well as desaturation ≥ 88%, Other minor adverse reactions observed during exercise were dyspnea, dizziness, tachypnea, or sinus tachycardia.

3 Compensation Policy

i. The study is covered by clinical trial insurance for compensation.

ii. Compensation Criteria

- Compensation will be provided for damages resulting from the self-exercise intervention conducted according to the clinical trial protocol.

- If a study participant experiences physical harm (including death) due to their participation in the clinical trial, compensation will be provided to the participant.

- In case of physical harm to a study participant due to their participation in the clinical trial, appropriate treatment will be provided through the responsible investigator before monetary compensation is confirmed.

- Damages incurred during the management of adverse events or during the process of addressing adverse reactions will also be considered for compensation.

iii. Exclusions from Compensation

- Cases where physical harm (including death) is not directly related toparticipation in the clinical trial will be excluded from compensation.

- Incidents that are considered to have occurred regardless of the clinical trial, such as accidents or occurrences unrelated to the trial, will not be eligible for compensation.

- Participants who received only conventional rehabilitation as the control group and cannot be provided with therapeutic benefits will also be excluded from compensation.

- Damages resulting from the usual complications of the disease or disease progression will be excluded from compensation.

- If damages occur due to non-compliance with the instructions of the participant or their guardian, or non-adherence to the clinical trial protocol, intentional misconduct, or significant negligence, the compensation amount may be reduced, or the participant may be excluded from compensation.

6) Criteria for Discontinuation or Withdrawal

1 Violation of inclusion/exclusion criteria.

2 Withdrawal of consent by the participant or their guardian.

3 During the clinical trial, if it is deemed difficult to continue the trial due to a significant adverse event or abnormal clinical examination results.

4 Any other circumstance where the trial discontinuation is deemed beneficial to the study participant, as determined by the investigator.

7) Safety evaluation criteria, evaluation methods, and reporting methods, including side effects

All adverse events (AEs) occurring during this clinical trial, including reported abnormal signs, symptoms, or diseases in research participants, are included in the safety evaluation. Vital signs measured during visits, clinical examinations, and adverse events reported by research participants are used to assess safety.

1 Definition of adverse events:

i. "Adverse Event (AE)" refers to all harmful and unintended signs or symptoms or diseases that occur in research participants during the clinical trial, and it does not necessarily imply a causal relationship with the intervention in the trial.

ii. "Adverse Device Effect (ADE)" refers to all harmful and unintended reactions caused by the investigational medical device during the clinical trial, and it is presumed to have a causal relationship with the investigational medical device.

iii. "Serious AE" refers to an adverse event or reaction that meets any of the following criteria:

(1). Results in death or poses a risk to life.

(2). Requires hospitalization or prolongs an existing hospitalization.

(3). Causes persistent or significant disability, long-term impairment, or functional loss.

(4). Causes a congenital anomaly or birth defect.

2 Adverse event evaluation

i. Severity assessment

(1). Grade 1: Mild; the adverse event does not interfere with the research participant's normal activities (functioning) and causes minimal discomfort that the research participants can easily tolerate.

(2). Grade 2: Moderate; the adverse event causes some interference with the research participant's normal activities (functioning) and results in noticeable discomfort.

(3). Grade 3: Severe; the adverse event makes it impossible for the research participant to carry out normal activities (functioning).

i. Causality assessment

(1). Definitely related

(2. Probably related

(3). Possibly related

(4). Probably not related

(5). Definitely not related

(6). Unknown

ii. Safety evaluation criteria

(1). In this clinical trial, adverse events are classified as any undesirable

medical signs or symptoms occurring after the start of the trial that were not observed before the trial.

(2). Predicted side effects are also classified as adverse events, and the severity of adverse events is categorized as mild, moderate, or severe.

(3). Criteria, methods, and responses for the assessment of predicted side effects are provided for each item.

**Predicted Safety Effects**	**Assessment Criteria**	**Assessment Method**	**Response**
Fall	Fall from bed or during mobility training resulting in injury	Confirmed by therapist and medical staff	Report immediately to attending medical staff and rehabilitation prescribing physician; additional tests like X-ray, CT may be performed if necessary.
Fracture	Confirmation of bone fracture from imaging test	Clinical symptoms and X-ray Report	Report immediately to attending medical staff and rehabilitation prescribing physician; additional tests like X-ray, CT may be performed if necessary.
Severe pain	Severe pain strongly suspected by the critical care patient or with bruising or swelling in a specific area	occurring during or shortly after rehabilitation and lasting for more than 3 days	Confirmation of pain in the affected area; report to medical staff and rehabilitation prescribing physician, additional tests like X-ray may be

			performed if necessary.
Hemodynamic Instability During Exercise	Arrhythmia, bradycardia, Systolic blood pressure > 200mmHg, Systolic blood pressure) < 90mmHg, SpO2 ≤ 88% etc	Confirmed by therapist and medical staff	Report immediately to medical staff, request ENT consultation for airway examination, additional tests if necessary, and inform rehabilitation prescribing physician.
Other Mild Adverse Reactions	Dyspnea, dizziness, tachypne or sinus tachycardia, agitation etc	Confirmed by therapist and medical staff	Report immediately to medical staff, request ENT consultation for airway examination, additional tests if necessary, and inform rehabilitation prescribing physician.
Other Adverse Reactions	-	Confirmed by therapist and medical staff	Report immediately to medical staff, request ENT consultation for airway examination, additional tests if necessary, and inform rehabilitation prescribing physician.

3 Safety Evaluation Method

i. All observed predicted side effects and dverse reactions should be listed with

detailed explanations.

ii. Record the frequency of adverse reactions associated with the investigational medical devices during the clinical trial and those unrelated to the investigational medical devices.

4 Adverse Reaction Reporting Method and Measures When Adverse Reactions Occur

i. Adverse Reaction Education:

The principal investigator shall provide education to the research personnel, research participants, or their representatives about all potential adverse reactions that may occur during the trial and educate them to report any events that occur after use.

ii. Serious Adverse Reaction/Medical Device Adverse Reaction Reporting Method: - The principal investigator shall report all significant adverse reactions that occur during the research period to the IRB within 24 hours, regardless of the relationship with the investigational intervention. Other significant adverse reactions, considered by the researcher to be severe or indicating significant risks, contraindications, side effects, or precautions related to the investigational intervention, shall be recorded as serious adverse reactions and reported to the IRB within 7 days from the date the researcher becomes aware of the event. Any other significant or unexpected adverse reactions shall be reported to the sponsor within 15 days from the date the researcher becomes aware of the event.

iii. Measures When Adverse Reactions Occur: - During the clinical trial period, the principal investigator and research personnel must ensure the safety of the research participants, and in the event of an unexpected severe adverse reaction, take prompt and appropriate measures to minimize the adverse reaction. - If any predicted or unpredicted adverse reactions occur due to additional procedures or interventions beyond the standard clinical process during the clinical trial, the research personnel shall directly contact the investigator and provide guidance for applying for compensation, ensuring that the research participants receive the best possible medical treatment, and compensating the research participants when there is a reasonable causal relationship between the clinical trial and the injury.

iv. Tracking and Observing Adverse Reactions: - The researcher must track and observe the research participants who experienced adverse reactions until their

symptoms disappear and their condition stabilizes. If necessary, submit a report on the subsequent course of the adverse reactions to the IRB.

8) Data Analysis and Statistical Plan

1 Primary outcome: Pittsburgh Rehabilitation Participation Scales at baseline, 3 weeks and 12 weeks post-treatment between control and experimental groups, analyzed using repeated measures ANOVA or linear-mixed model.

2 Secondary outcome: Analyzed using repeated measures ANOVA or linear-mixed model for secondary endpoints at baseline, 3 weeks, and 12 weeks post-treatment between control and experimental groups.

9) Data Management Plan

1 Data Recording, Collection, Access, Protection, and Storage

i. When data are modified, the process of modification should be documented, and the original data should not be deleted. In case of changes or corrections in the contents of documented or electronically recorded case report forms, the modifications should be made in accordance with the written modification guidelines, ensuring that the original content can still be identified. The modification date, reason for modification, and the person making the modification should be recorded and signed.

ii. To ensure data management for the clinical trial, access to data by unauthorized individuals should be prevented by using individual IDs and passwords.

iii. A list of authorized personnel for data modification should be maintained.

iv. Copies of the data should be kept.

v. If data blinding (masking) is required for the specific clinical trial, the data entry and processing should be conducted while maintaining the blinding status.

10) Research Schedule

Months	1-4	5-8	9-12	13-16	17-20	21-24	25-28	29-32	33-36
FDA Medical Device Clinical Trial Plan Approval	●								
Medical Device Clinical Trial IRB Approval	●								
Medical Device Clinical Trial	●	●	●	●	●	●	●		
Statistical Analysis and Results Verification for Clinical Trial Data								●	

Writing and publishing									●

11. Data and Safety Monitoring Plan

1) Monitoring Personnel

1 Monitoring Responsible Person: Prof. Woo Hyung Lee, Clinical Assistant Professor, Department of Rehabilitation Medicine, Seoul National University Hospital

2 Monitoring Manager: Prof. Jung Hyun Kim, Research Professor, Department of Rehabilitation Medicine, Seoul National University Hospital

2) Data and Safety Monitoring Items

1 Study accruals items: Clinical information from Case Report Forms (CRFs)

2 Safety items: [14, 16, 22]

① Fall

② Fracture

③ Severe pain

④ Endotracheal tube removal

⑤ Arterial line removal

⑥ Central line removal

⑦ Hemodynamic Instability During Exercise

i. Arrhythmia

ii. Bradycardia

iii. Systolic blood pressure > 200mmHg

iv. Systolic blood pressure < 90mmHg

v. Desaturation ≥ 88%

⑧ Other Mild Adverse Reactions

i. Dyspnea

ii. Dizziness

iii. Tachypnea or Sinus tachycardia etc

3) Data and Safety Monitoring Methods and Frequency

① Data and Safety Monitoring Methods

During the participant's informed consent process, the research team explains the reporting system for safety monitoring. If any adverse reactions related to the research occur during the study, the entire research team will be immediately informed, and

appropriate actions will be taken.

② Data and Safety Monitoring Frequency

Data and safety monitoring will be conducted after every 5 enrolled research participants.

4) Medical Device Adverse Reaction Reporting, Non-compliance with the Study, Reporting of Unexpected Problems

In the event of a serious adverse reaction, significant non-compliance with the study, or an unexpected problem occurring, the researcher must report it to the Institutional Review Board (IRB) through the monitoring responsible person within 15 working days from the date of awareness.

5) Criteria for Study Termination

- This study involves multimodal exercise intervention in stroke patients during routine evaluation and treatment processes commonly performed in the clinical setting. It is anticipated that the additional risks associated with the intervention will be low. Additionally, it is predicted that the likelihood of severe complications occurring, which would not have occurred if the intervention were not performed, is very low. However, if it is determined that the incidence rate of complications is significantly higher compared to before the study initiation, the study will be halted.
- If more than 25% of the intended research data is not acquired within 9 months or if more than 50% is not acquired within 18 months, the research team will conduct a reevaluation through a meeting of involved parties and decide whether to resume the study.

12. Measures for Participant Safety and Protection

1) Basic Approaches to Ensure Research Ethics:

- The research team, including the principal investigator and personnel, will adhere to the Helsinki Declaration (2013 revision) and ICH-GCP guidelines and conduct the study after obtaining IRB approval. Before starting the study, all relevant information about the research, including the study objectives, potential effects, adverse reactions, and safety, will be sufficiently explained to the research participants and their legal representatives in a language they can understand. Voluntary informed consent will be obtained from the research participants and their legal representatives before their participation in the study.
- The purpose and methods of the study will be explained to potential research

participants and their legal representatives through an informed consent form. Only those who understand the study's objectives and provide written consent will be eligible to participate. Records identifying the research participants will be kept confidential, and the identity of the research participants will remain confidential when publishing the study's results.

2) Informed Consent Process for Research Participants:

- For this study, the principal investigator (Prof. Woo Hyung Lee) or the research personnel will explain all relevant information about the research, including the study objectives, potential effects, adverse reactions, and safety, to potential research participants and their legal representatives at Seoul National University Hospital. The explanation will be provided in a language they can understand and will last for at least 10 minutes (if necessary, several days) to ensure sufficient understanding before obtaining voluntary informed consent for study participation.
- Minimizing the possibility of coercion or undue influence: Participation in the clinical trial will be completely voluntary, and adequate time (several days if needed) will be given to consider participation. The decision to participate will not be linked to the medical treatment provided at the institution for potential research participants.

3) Compensation Plan for Research Participants:

- There will be no remuneration for participation in the critical care rehabilitation and multimodal exercise intervention conducted within the hospital.
- For those who participate in the study until the 12th week measurement, the researchers will pay ₩ 400,000 including the evaluation fee within 2 weeks after the end of the 3rd week measurement, and ₩ 200,000 within 2 weeks after the end of the 12th week measurement, for a total of ₩ 600,000.
- The research responsibility includes not only covering all medical expenses for unforeseen complications but also providing compensation for any damages that may occur.

4) Measures for Protection of Research Participants' Personal Information

- Patient medical record numbers and pathology numbers will be kept in a separate file under the responsibility of the principal investigator. The data will be coded so that individual identification is not possible through research data management. Alternatively, research data will be stored in password-protected files and kept in a locked facility with security measures. According to the medical device clinical trial management standards,

research-related records will be retained for three years from the date of approval of the clinical trial results report. If the storage facility needs to retain certain documents related to personal information for more than three years for subsequent research, record-keeping, or accumulation purposes, such information will be managed to prevent individual identification. In cases where the trial results are published, the identity of the research participants will be kept confidential.

5) Additional Protective Measures for Vulnerable Research Participants

- If the research participants' capacity to provide informed consent is deemed difficult due to impaired decision-making abilities, their participation in the research will be obtained through their own assent and the consent of their legal representatives.
- The evaluation of consent capacity will consider factors such as whether the research participants can understand the information related to the study, handle the information logically, and express a clear choice regarding their desire to participate in the study.
- Protective Measures for Adults with Impaired Consent Capacity (Refer to HRPP SOP ver.3.7)

  - This study targets stroke patients.
  - It is anticipated that the expected benefits to the research participants will outweigh the foreseeable risks.
  - The evaluation of the research participants' consent capacity may include assessing their ability to understand the information related to the research, handle the information logically, and express a clear choice regarding their desire to participate. Since the ability to consent may fluctuate based on the research participants' condition, the understanding and willingness of the research participants for research participation will be repeatedly assessed.
  - Even if consent is initially obtained from the legal representatives, if the research participants gain the capacity to provide consent during the course of the study, the researchers must obtain consent from the research participants.
  - In emergency situations where obtaining prior consent from the research participants is not possible, consent from accompanying legal representatives must be obtained, and if there is no accompanying legal representative, written approval from the IRB must be obtained. In such cases, the principal investigator and researchers must inform the research participants or legal representatives about the research as soon as possible and obtain their continued consent for

participation in the research.

13. Refference

1. Hemmatpour, M., et al., *A Review on Fall Prediction and Prevention System for Personal Devices: Evaluation and Experimental Results.* Advances in Human-Computer Interaction, 2019. **2019**.
2. Chung, I.Y., et al., *An Assessment of Physical Activity Data Collected via a Smartphone App and a Smart Band in Breast Cancer Survivors: Observational Study.* Journal of Medical Internet Research, 2019. **21**(9).
3. Barton, J., B. O'Flynn, and S. Tedesco, *A Review of Physical Activity Monitoring and Activity Trackers for Older Adults.* Harnessing the Power of Technology to Improve Lives, 2017. **242**: p. 748-754.
4. Woodford, H. and C. Price, *EMG biofeedback for the recovery of motor function after stroke (Review).* Cochrane Database of Systematic Reviews, 2007(2).
5. Rasool, G., et al., *Spatial Analysis of Multichannel Surface EMG in Hemiplegic Stroke.* Ieee Transactions on Neural Systems and Rehabilitation Engineering, 2017. **25**(10): p. 1802-1811.
6. Gamez, A.B., et al., *The effect of surface electromyography biofeedback on the activity of extensor and dorsiflexor muscles in elderly adults: a randomized trial.* Scientific Reports, 2019. **9**.
7. Klassen, T.D., et al., *Higher Doses Improve Walking Recovery During Stroke Inpatient Rehabilitation.* Stroke, 2020. **51**(9): p. 2639-2648.
8. Hornby, T.G., et al., *Contributions of Stepping Intensity and Variability to Mobility in Individuals Poststroke A Randomized Clinical Trial.* Stroke, 2019. **50**(9): p. 2492-2499.
9. Lacroix, J., et al., *Physical Activity Level Among Stroke Patients Hospitalized in a Rehabilitation Unit.* Pm&R, 2016. **8**(2): p. 97-104.
10. Everard, G., et al., *Self-Rehabilitation for Post-Stroke Motor Function and Activity-A Systematic Review and Meta-Analysis.* Neurorehabilitation and Neural Repair, 2021. **35**(12): p. 1043-1058.
11. Chen, J., et al., *Effects of Home-based Telesupervising Rehabilitation on Physical Function for Stroke Survivors with Hemiplegia A Randomized Controlled Trial.* American Journal of Physical Medicine & Rehabilitation, 2017. **96**(3): p. 152-160.
12. Golla, A., et al., *Home-based balance training using Wii Fit™: a pilot randomised controlled trial with mobile older stroke survivors.* Pilot and feasibility studies, 2018. **4**(1): p. 1-10.

13. Peretti, A., et al., *Telerehabilitation: review of the state-of-the-art and areas of application.* JMIR rehabilitation and assistive technologies, 2017. **4**(2): p. e7511.
14. Hsieh, C.L., I.P. Hsueh, and H.F. Mao, *Validity and responsiveness of the rivermead mobility index in stroke patients.* Scandinavian Journal of Rehabilitation Medicine, 2000. **32**(3): p. 140-142.
15. Hsueh, I.P., et al., *Comparison of psychometric properties of three mobility measures for patients with stroke.* Stroke, 2003. **34**(7): p. 1741-1745.
16. Hernández, E.D., et al., *Intra-and interrater reliability of Fugl-Meyer Assessment of Lower Extremity early after stroke.* Brazilian Journal of Physical Therapy, 2020.
17. Kudlac, M., et al., *Reliability and validity of the Berg balance scale in the stroke population: a systematic review.* Physical & Occupational Therapy in Geriatrics, 2019. **37**(3): p. 196-221.
18. Viosca, E., et al., *Proposal and validation of a new functional ambulation classification scale for clinical use.* Archives of Physical Medicine and Rehabilitation, 2005. **86**(6): p. 1234-1238.
19. Rebchuk, A.D., et al., *Health utility weighting of the modified Rankin scale: a systematic review and meta-analysis.* JAMA network open, 2020. **3**(4): p. e203767-e203767.
20. Ohura, T., et al., *Validity and reliability of a performance evaluation tool based on the modified Barthel Index for stroke patients.* BMC medical research methodology, 2017. **17**(1): p. 1-8.
21. Lenze, E.J., et al., *The Pittsburgh Rehabilitation Participation Scale: reliability and validity of a clinician-rated measure of participation in acute rehabilitation.* Archives of physical medicine and rehabilitation, 2004. **85**(3): p. 380-384.
22. Anderson, C., S. Laubscher, and R. Burns, *Validation of the Short Form 36 (SF-36) health survey questionnaire among stroke patients.* Stroke, 1996. **27**(10): p. 1812-1816.
23. 국가법령전보센터, 의료기기법 시행규칙, 2022.01.
24. 식품의약품안전처, 의료기기 품목 및 품목별 등급에 관한 규정, 2022.03
25. 식품의약품안전처, 의료기기 임상시험 안내서, 2020.11.
26. 식품의약품안전처, 의료기기 임상시험 길라잡이, 2020.03.
27. 식품의약품안전처, 의료기기 임상시험계획 승인에 관한 규정, 2016.11.
