# Supplementary material for: Protocol for a single-blind randomized controlled clinical trial to investigate the feasibility and safety of in-bed self-exercises based on electromyography sensor feedback in patients with subacute stroke
Source: PLoS One. 2024 Dec 30;19(12):e0310178. doi: 10.1371/journal.pone.0310178 (PMC11684591; doi:10.1371/journal.pone.0310178)
Supplement: S2 Protocol — (PDF) [file pone.0310178.s005.pdf]

아급성기 뇌졸중 환자에서 근전도 센서 피드백에 기반한 침상내 자가운동의 기능적 이동성에 대한 효과: 전향적, 다기관, 공개, 평가자 눈가림, 탐색적 예비임상시험

Effects of in-bed self-exercises based on EMG-sensor feedback on functional mobility in subacute stroke patients: a prospective, multicenter, single-blind, open-label, exploratory pilot clinical trial

Version No: 2.5

책임연구자 소속: 서울대학교병원 재활의학과
책임연구자 이름: 이우형

목차

연구 개요 4

연구계획서 7

1. 연구 제목 7
2. 연구 실시기관 명칭 및 주소 7
3. 연구책임자 및 공동연구자 성명 및 직명 7
4. 연구비 지원기관 명칭 및 주소 8
5. 예상연구기간 8
6. 연구 대상 질환 8
7. 연구의 배경 및 목적 8
8. 시험용 의료기기의 개요 9
9. 연구대상자의 선정 기준, 제외 기준, 목표한 대상자 수 및 산출 근거 27
10. 연구 방법 29
11. 자료 및 안전성 모니터링 계획 (Data and Safety Monitoring Plan) 49
12. 연구대상자의 안전보호를 위한 대책 51
13. 인체유래물의 보관 및 폐기 방법 54
14. 참고 문헌 54

[별첨 1] 자료 및 안전성 모니터링 계획서 (Data and Safety Monitoring Plan) 57

[별첨 2] 연구대상자 설명문 및 동의서 58

[별첨 3] 증례기록서 64

[별첨 4] 연구대상자 모집 공고문 79

[별첨 5] 임상시험 피해자 보상규약 80

[별첨 6] 책임연구자의 이력 82

[별첨 7] 평가도구 1 88

[별첨 7] 평가도구 2 94

[별첨 7] 평가도구 3 106

[별첨 7] 평가도구 4 108
[별첨 7] 평가도구 5 113
[별첨 7] 평가도구 6 115
[별첨 7] 평가도구 7 117
[별첨 7] 평가도구 8 122
[별첨 7] 평가도구 9 129
[별첨 7] 평가도구 10 132
[별첨 7] 평가도구 11 136
[별첨 7] 평가도구 12 138
[별첨 7] 평가도구 13 142
[별첨 9] 생체현상 측정기기 의료기기 제조 및 품질관리 기준 적합인정서 144
[별첨 10] 소프트웨어 의료기기 제조 및 품질관리 기준 적합인정서 145
[별첨 11] 연구비 내역서 146
[별첨 12] 임상시험배상보험 가입 증권 148
[별첨 13] 식약처 임상시험계획승인서 150
[별첨 14] 근전도계 의료기기 제조 인증서 152
[별첨 15] 변경된 근전도계 의료기기 제조 인증서 154
[별첨 16] 한국의료기기안전정보원 변경인증 157
[별첨 17] 식약처 임상시험계획 변경보고서 160

연구 개요

연구제목	(국문) 아급성기 뇌졸중 환자에서 근전도 센서 피드백에 기반한 침상내 자가운동의 기능적 이동성에 대한 효과: 전향적, 다기관, 공개, 평가자 눈가림, 탐색적 예비임상시험
	(영문) Effects of in-bed self-exercises based on EMG-sensor feedback on functional mobility in subacute stroke patients: a prospective, multicenter, single-blind, open-label, exploratory pilot clinical trial
임상시험 기관	서울대학교병원
책임연구자	재활의학과 이우형 교수
연구비 지원기관	산업통상자원부

연구 목적	아급성기 뇌졸중 환자에서 표준적인 하지재활에 더하여 근전도센서 피드백에 기반한 하지 자가운동을 추가하는 선행적 임상시험을 수행함으로써, 해당 치료의 기능적 이동성을 개선시키는 데에 대한 효과(effect) 및 타당성(feasibility)을 확인
임상시험 종류	연구자주도 임상시험
연구 설계	의료기기 임상시험 (전향적, 다기관, 공개, 평가자 눈가림, 탐색적 예비임상시험)
연구 기간	임상시험계획 승인일로부터 36 개월
연구 대상	아급성기 뇌졸중 환자
연구 대상자 수	시험군 뇌졸중 환자 12 명<br>대조군 뇌졸중 환자 12 명<br>(탐색임상시험이므로 최소한의 규모로 진행함)
취약한 연구대상자	손상된 동의 능력을 가진 성인
연구 방법	1. 연구대상자: 뇌졸중 발생 3개월 이내의 하지 근력이 저하된 성인 뇌졸중 환자<br>2. 무작위화: 나이(<65/≥65), 초기 mRS(2-3/4-5)에 따라 층화 무작위배정<br>(1) 대조군 설정<br>· 시험군: 표준 하지재활 + 근전도 피드백에 기반한 감독하

(supervised) 자가운동 제공

- 대조군: 표준 하지재활

(2) 자가운동

- 개발된 자가 운동 프로토콜에 따라 환자는 혼자서 또는 보호자의 도움 하에 적절한 운동 유형과 방식을 선정하고, 적절한 자세를 취한 상태에서 수행됨.
- 대조군에는 표준 하지재활만이 제공되며, 시험군에는 근전도 피드백에 기반한 감독하 자가운동에 제공됨

3. 임상평가 항목

(1) 일차 평가 변수(primary outcome)

■ 평가시점: 기준시점(baseline) – 3주후 – 12주후

- 리버미드 운동 평가(Rivermead motor assessment, RMA): Gross function, leg and trunk

(2) 이차 평가 변수(secondary outcome)

■ 평가시점: 기준시점(baseline) – 3주후 – 12주후

- 도수근력검사(Manual muscle test, MMT): Hip flexor, Hip abductor, Knee extensor, Ankle dorsiflexor
- 브룬스트롬 기능회복 단계(brunnstrom stages of motor recovery, BRS)
- 푸글마이어 척도-하지(Fugl Meyer assessment of lower extremity, FMS-LE)
- 버그균형척도(Berg balance scale, BBS)
- 기능적 보행 분류(Functional ambulation category, FAC)
- 수정 랭킨 척도(modified Rankin scale, mRS)
- 수정 바델 지수(modified Barthel index, MBI)
- 피츠버그 재활 참여 척도(Pittsburgh Rehabilitation Participation Scale, PRPS)
- Short-form Health Survery 36 version 2(SF-36v2)

4. 운동프로토콜 적용을 위한 평가 항목

■ 평가시점: 1주후 – 2주후

- 도수근력검사(Manual muscle test, MMT): Hip flexor, Hip abductor, Knee extensor, Ankle dorsiflexor
- 브룬스트롬 기능회복 단계(brunnstrom stages of motor recovery, BRS)
- 피츠버그 재활 참여 척도(Pittsburgh Rehabilitation Participation Scale, PRPS)

5. 의료기기 사용부위

(1) 가쪽넓은근

(2) 앞정강근

6. 의료기기 사용방법

	(1) 본체를 패치와 연결하여 목표가 되는 근육에 부착<br>(2) 사용자 앱에 로그인하여 할당된 운동을 지시에 맞추어 진행<br>· 자가 운동을 진행하는 동안 목표 근육의 근 활성도를 확인하며 운동 수행
의료기기 정보	Myoverse (주)에스엠디솔루션
유효성 평가	1. 1차 유효성 평가 지표: 리버미드 운동 평가<br>2. 2차 유효성 평가 지표: 도수근력검사(MMT), 브룬스트롬 기능회복 단계(BRS), 푸글마이어 척도-하지(FMS-LE), 수정 랭킨 척도(mRS), 기능적 보행 분류(FAC), 버그균형척도(BBS), 수정 바델 지수(MBI), Short-form Health Survery 36 version 2(SF-36v2), 피츠버그 재활 참여 척도(PRPS)
안전성 평가	재활과 연관된 모든 부작용(adverse event): 낙상, 골절, 심각한 통증, 운동 중 발생한 혈역학적 불안정성, 기타 경미한 이상반응(현기증, 빈호흡, 부비동 빈맥, 초조 등)
기대효과 및 예상결과	아급성기 뇌졸중 환자들의 운동횟수를 증가시킴으로써 뇌졸중 환자들의 이동 능력, 일상 생활, 삶의 질이 개선될 수 있음. 이는 의료, 사회적 비용 절감으로 이어질 수 있음. 근전도센서 피드백에 기반한 자가운동이 효과적이라면 관련 산업이 태동하고 발전하는 기여할 수 있음

연구계획서

1. 연구 제목

아급성기 뇌졸중 환자에서 근전도 센서 피드백에 기반한 침상내 자가운동의 기능적 이동성에 대한 효과: 전향적, 다기관, 공개, 평가자 눈가림, 탐색적 예비임상시험

2. 연구 실시기관 명칭 및 주소

기관1: 서울대학교병원 재활의학과
주소: 서울시 종로구 이화동 대학로 101
전화: 02-2072-4178
팩스: 02-6072-5244
서울대학교병원 재활의학과 임상조교수 이우형

기관2: 국립교통재활병원
주소: 경기도 양평군 양평읍 중앙로 260
전화: 031-580-5555
팩스: 031-580-5785
국립교통재활병원 재활의학과 진료교수 오현미

3. 연구책임자 및 공동연구자 성명 및 직명

1) 연구책임자

서울대학교병원 재활의학과 임상조교수 이우형

2) 공동연구자

서울대학교병원 재활의학과 임상부교수 오병모
국립교통재활병원 재활의학과 진료교수 오현미
서울대학교병원 재활의학과 임상부교수 서한길
서울대학교병원 재활의학과 진료조교수 현성은
서울대학교병원 재활의학과 연구원 전소연

3) 연구담당자

서울대학교병원 재활의학과 연구교수 김정현

4) 임상시험 모니터링 요원

서울대학교병원 재활의학과 연구교수 김정현
국립교통재활병원 재활의학과 연구원 오수민

5) 임상시험 조정자

서울대학교병원 재활의학과 임상조교수 이우형

6) 임상시험용 의료기기 관리자

서울대학교병원 재활의학과 연구교수 김정현

국립교통재활병원 재활의학과 연구원 오수민

4. 연구비 지원기관 명칭 및 주소

기관: 산업통상자원부

주소: 세종특별자치시 한누리대로 402 12동, 13동

5. 예상연구기간

임상시험계획 승인일로부터 36개월

6. 연구 대상 질환

뇌졸중 발생 3개월 이내의 하지 근력이 저하된 성인 뇌졸중 환자

7. 연구의 배경 및 목적

1) 연구 배경

뇌졸중은 전 세계적으로 장애의 주요 원인이며 가장 흔한 증상으로 근력 약화 및 수축, 운동 조절 장애 등의 문제가 발생하며 그 결과 일상생활 및 삶의 질, 활동을 수행함에 있어 기능적 독립성이 감소하게 됨. 최근 뇌졸중 후 운동 평가를 위하여 환자의 자세와 움직임을 관찰하고 분석하는 멀티모달 센서 기반 웨어러블 시스템을 사용하는 것이 큰 관심을 받고 있음[1-3]. 그 중 대표적인 것이 바로 근전도와 관성 센서를 동시 사용하는 시스템임. 근전도는 상, 하지의 근육의 활성도를 측정하고 관성 센서는 운동 범위와 활동 정도를 측정하게 됨.

기존의 뇌졸중에 대한 운동 재활치료는 치료사가 환자를 일대일로 지도하며 환자가 스스로 앉고, 서고, 보행을 할 수 있도록 하며, 적절한 보조를 함께 제공하여 본인의 의지로 근육을 조정하며 동작을 수행하도록 함. 이 때 동작에 따른 적절한 근육의 활성도가 성공적인 동작을 수행하는 데에 매우 중요하며, 뇌손상으로 인한 저하된 근육의 활성도를 증가시키는 것이 치료의 효과를 개선시키는 데에 중요할 수 있음[4-6].

기존의 연구에서 보행 치료의 효과를 증대시키기 위하여 웨어러블 센서를 활용하여 운동량을 정량적으로 평가하고 획득한 정보를 치료에 활용하였을 때 효과가 증대된다는 연구 결과가 보고되었음[7]. 그러나 이 연구의 단점은 보행이 가능한 뇌졸중 환자를 대상으로 진행되어 이러한 접근의 치료의 대상 범위가 매우 좁다는 한계가 있음. 보행 또는 균형 장애로 인하여 하지재활이 필요한 환자들을 대상으로 이러한 접근의 치료의 범위를 확장할 필요가 있음. 한편으로 뇌졸중 재활에 있어 환자에게서 획득된 정보를 바탕으로 적절한 피드백을 제공하는 것은 보행과 균형기능의 회복에 도움이 된다는 연구도 보고되었음.

최근 뇌졸중 환자의 이동 기능 회복에 있어 재활치료를 높은 강도로 받는 것이 중요하다고 보고된 바 있음[8]. 재활치료의 높은 양과 강도가 운동기능에 있어 중요하나, 실제로는 충분한 강도 및 양으로 제공이 되고 있지 못함[9]. 자가운동 시행의 효과에 대한 연구들이 발표되고 있으며, 최근 발표된 체계적 고찰 연구에서 자가운동 프로그램은 고식적재활과 유사한 정도의 치료효과가 있다고 보고되었음[10]. 하지만 관련 연구들은 대부분 상지재활에 대

한 보고였으며, 하지재활에 대해서는 2건의 연구가 발표되었고 고식적 하지재활과 유사한 효과를 보였음[11, 12]. 상지재활과 비교하였을 때 하지에 대한 자가운동은 아직까지 충분히 연구되지 않은 상태임.

기존 연구들에서 자가 운동은 주로 치료사에 의해서 감독을 받으나, 최근에는 다양한 기술들의 발전으로 인해서 스마트폰, 움직임 감지 시스템, 컴퓨터 기반 소통 도구 등 원격 소통기기(telecommunication device)를 활용하는 자가 운동 프로그램에 대한 연구들도 보고되고 있음[13]. 하지만 아직까지 이러한 자가운동의 치료적 효과 및 타당성을 검증하기 위한 연구조차 부족한 것이 현실임. 이에 근전도센서 피드백에 기반한 하지 자가운동을 추가하는 선행적 임상시험을 수행함으로써, 해당 치료의 기능적 이동성을 개선시키는 데에 대한 효과(effect) 및 타당성(feasibility)을 확인하고자 함.

2) 연구 가설 및 목적

(1) 연구 가설

귀무가설: 아급성기 뇌졸중 환자 중 표준하지재활 치료를 시행한 군과 추가적인 근전도를 통해 획득된 데이터 및 이를 활용한 피드백에 기반한 감독하 자가운동이 제공된 군 사이의 리버미드 운동평가 점수는 통계적으로 유의한 차이가 없다.

대립가설: 아급성기 뇌졸중 환자 중 표준하지재활 치료를 시행한 군과 추가적인 근전도를 통해 획득된 데이터 및 이를 활용한 피드백에 기반한 감독하 자가운동이 제공된 군 사이의 리버미드 운동평가 점수는 통계적으로 유의한 차이가 있다.

(2) 연구 목적

아급성기 뇌졸중 환자에서 표준적인 하지재활에 더하여 근전도센서 피드백에 기반한 하지 자가운동을 추가하는 선행적 임상시험을 수행함으로써, 해당 치료의 기능적 이동성을 개선시키는 데에 대한 효과(effect) 및 타당성(feasibility)을 확인하고자 함.

8. 시험용 의료기기의 개요

1) 시험기기

(1) 품목명: 근전도분석소프트웨어, 근전도계(제인 22-4998)

(2) 품목코드: E10010.01

(3) 품목등급: 2등급

(4) 모델명: Myoverse s/w

(5) 제조자: ㈜에스엠디솔루션

(6) 사용목적

뇌졸중 환자의 재활 치료에 사용하는 자가운동 프로그램으로 의사의 처방에 의한 재활운동을 시행할 때 근전도 센서로부터 측정된 근전도 데이터를 분석해서 재활치료에 적용

(7) 대상질환 및 적응증

해당 의료기기는 입원 재활 단계의 뇌졸중 환자 중 거동이 불편한 환자에게 적용

대분류	중분류	소분류
순환계통의 질환	뇌혈관질환	[I60- I69]

표 1 의료기기 품목 분류

(8) 사용시간

사용시점: 시험군 참여대상자의 입원기간 중 정규치료시간 이외 침상 휴식

시간사용횟수: 1일 1~2회

(9) 기기구성

① 명칭: Myoverse s/w

② 버전: 1.00.00

③ 사용환경

A. 모바일 어플리케이션 (ver.DOCTOR, ver.PATIENT)

i. 프로그래밍 언어

구분	이름	설명	버전
프로그래밍 언어	TypeScript	마이크로소프트에서 구현한 JavaScript 의 슈퍼셋(Superset) 프로그래밍 언어로써, 확장자로는 .ts 를 사용하며, 컴파일의 결과물로 JavaScript 코드를 출력한다.	4.2.4
화면 구현	Ionic	모바일 앱 구현을 위한 프레임워크로써, UI Component, Native Plugin 등을 제공한다.	5.5.2
기능 구현	Angular	모바일 앱의 기능 구현 시 사용하는 프레임워크로써, module, component, service 등의 기능을 제공한다.	12.0.0

표 2 모바일 앱 버전

B. 하드웨어 플렛폼

i. Mobile Application은 삼성전자 갤럭시 탭 S8 이상의 태블릿에서 모두 동작 가능하며, 주요 필요 사양은 아래와 같다.

구분	이름	설명
Android	프로세서	Qualcomm Snapdragon 8 Gen 1
	메모리	8GB

	디스플레이	Color / LTPS TFT 16M colors, 120Hz 1600 x 2560(11.00") 274ppi
	네트워크	Wi-Fi
	근거리 무선통신	Bluetooth V5.2

표 3 모바일 사양

C. 운영체제: 안드로이드 12 이상

D. 클라우드 서버

i. 프로그래밍 언어

이름	설명	버전
Node.js	Node.js 는 확장성 있는 네트워크 애플리케이션(특히 서버 사이드) 개발에 사용되는 소프트웨어 플랫폼이다. 작성 언어로 자바스크립트를 활용하며 논블로킹(Non-blocking)[3] I/O 와 단일 스레드 이벤트 루프를 통한 높은 처리 성능을 가지고 있다. 내장 HTTP 서버 라이브러리를 포함하고 있어 웹 서버에서 아파치 등의 별도의 소프트웨어 없이 동작하는 것이 가능하며 이를 통해 웹 서버의 동작에 있어 더 많은 통제를 가능케 한다.	12.22.1
MariaDB	MariaDB 는 오픈 소스의 관계형 데이터베이스 관리 시스템(RDBMS)이다. MySQL 과 동일한 소스 코드를 기반으로 하여 사용방법과 구조가 MySQL 과 동일하다. MySQL 과 비교해 애플리케이션 부분 속도가 약 4~5 천배 정도 빠르며, MySQL 이 가지고 있는 모든 제품의 기능을 완벽히 구현하면서도 성능 면에서는 최고 70%의 향상을 보이고 있다.	10.1.48

표 4 서버 프로그래밍 언어

ii. 클라우드 서버가 구동되는 하드웨어 사양은 아래와 같다.

구분	설명
프로세서	Intel(R) Xeon(R) Gold 5220 CPU @ 2.20GHz
메모리	8GB 이상
디스크	50GB 이상
메인보드	Intel 440FX - 82441FX PMC
운영체제	Ubuntu Server 18.04 (64-bit)

표 5 클라우드 서버 사양

2) 적용원리

(1) 근전도분석소프트웨어

의료진이 환자별 재활 치료에 필요한 동작을 의료진 앱에 입력한다. 환자는 재활치료를 위하여 근전도 센서를 인체에 부착하고 환자 이름을 입력한 후 처방받은 재활치료를 자가운동을 진행한다. 진행되는 동안 실시간 근전도 신호들을 볼 수 있고 완료 후 운동 수행 결과(운동시간, 운동횟수, 정확도)등을 확인한다.

① 근전도 데이터 취득 및 분석 여부

A. 피부 표면에 부착된 전극은 운동단위들의 공간 거리에 따라 전위가 형성되는데 부착된 전극 사이의 전위차를 증폭하여 근육의 전기적인 신호를 취득

B. RMS, MVC, 수행률, 목표달성률, 낙상위험도(정상인과 위험군을 함께 비교한 그래프 포함)의 분석 결과를 볼 수 있음

① 센서가 측정하는 인자와 목적

A. 패치 형태의 기기는 피부에 완전히 밀착되어 표면 뒤틀림에도 양질의 전기적인 신호를 측정하고, Mobile App과 Cloud Server에서 측정된 신호를 분석하여 균형 및 종합적인 근기능을 평가함으로써 의료진과 연동한 신경계질환자 모니터링 및 맞춤형 재활 치료에 사용된다.

① 재활운동의 정확도 측정

A. 의료진 앱에서 환자의 MVC를 측정한 후 의료진이 환자의 목표 MVC를 설정한다.

B. 정확도는 설정된 목표값의 50%이상부터 100%까지 목표값 대비 달성률을 반영한다.

C. 50%미만의 측정값은 로그에만 남기고 수행하지 않은 것으로 간주한다.

① 실시간 피드백 여부

A. 의료진 앱, 환자용 앱은 각각 운동 시 측정되는 신호를 바이오피드백 형식으로 모니터링 가능하지만 앱 간의 상호 실시간 모니터링은 불가

① 재활중 의료진 개입여부

A. 현재 원내에서 의료진 개입 하에 수행하도록 만들어지고 향후 통원치료에서도 활용할 수 있도록 수정할 계획이다.

① 소프트웨어의 자체적인 난이도 조절 여부

A. 의료진 앱은 프로그램 설정 페이지에서 난이도 설정 가능(횟수, 기간, MVC 값)

B. 환자용 앱은 난이도 설정 불가

① 환자 데이터 전송 여부

A. 환자는 운동 완료 버튼 클릭 시 서버로 데이터 전송

B. 수행률, 목표달성률

C. 가쪽넓은근(vastus lateralis)과 앞정강근(tibialis anterior) 등의 위치에 센서를 부착하고 운동수행 중의 근전도 신호 데이터를 획득하여 전송

3) 모양 및 구조 – 외형

(1) 근전도 분석프로그램

① 의료진 앱

A. 환자 등록

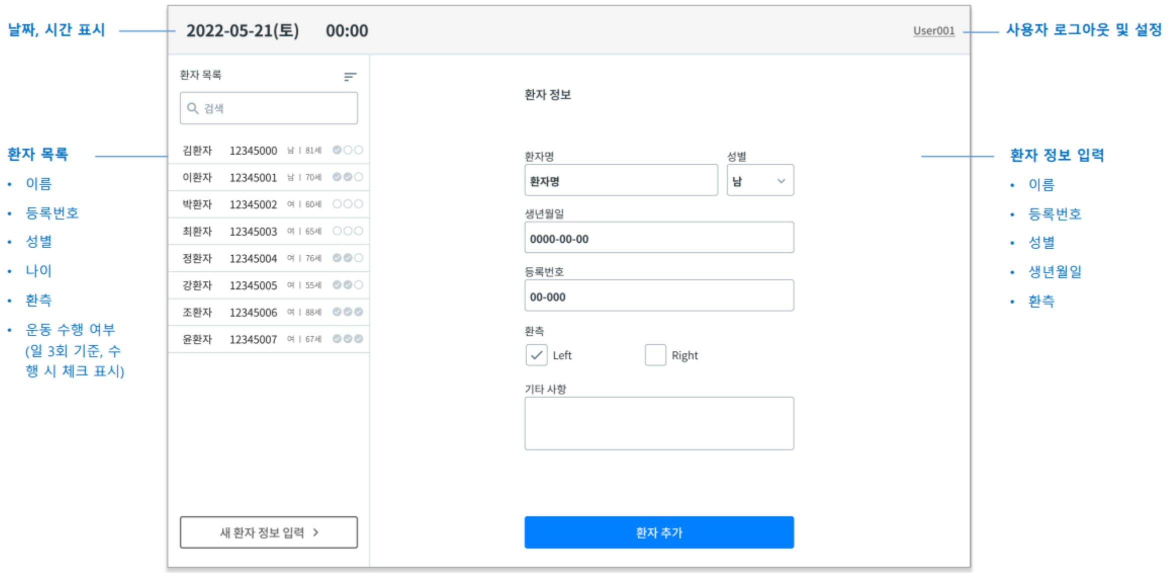

그림 1 환자 등록 화면

B. 패치 연결 테스트

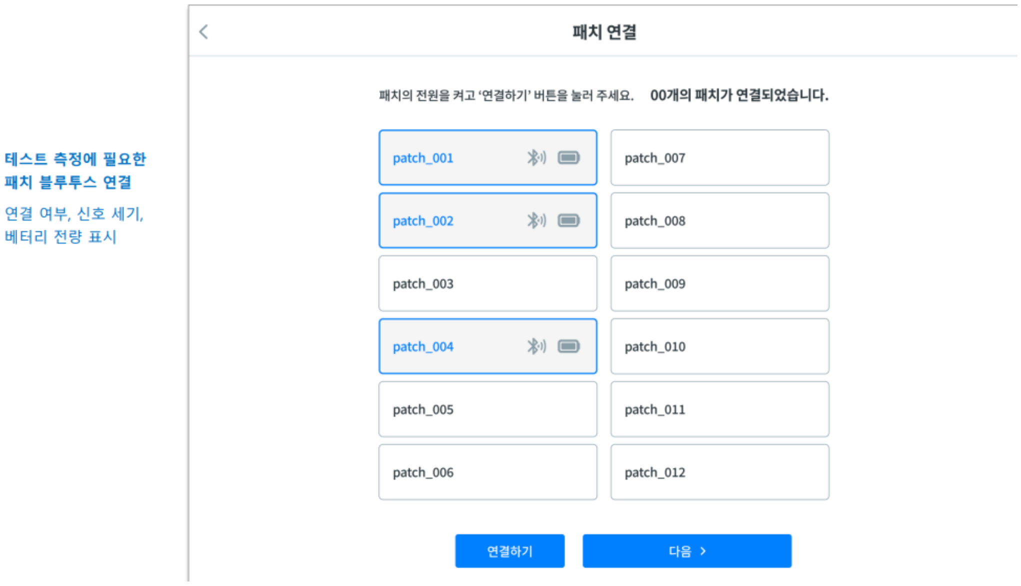

그림 2 패치 등록 화면

C. 동작 및 패치 부착위치 선택

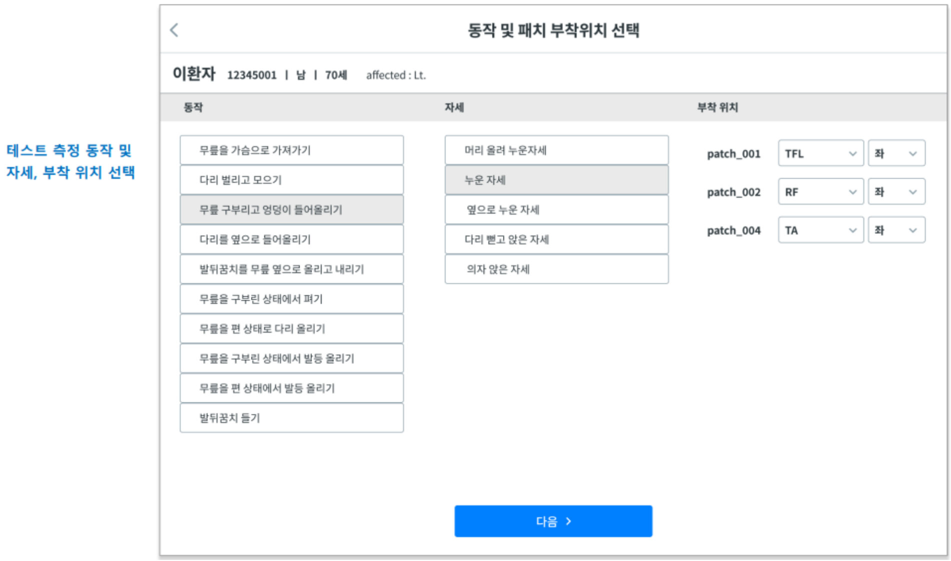

그림 3 동작 및 패치 부착위치 선택 화면

D. 신호 측정 후 분석결과 확인

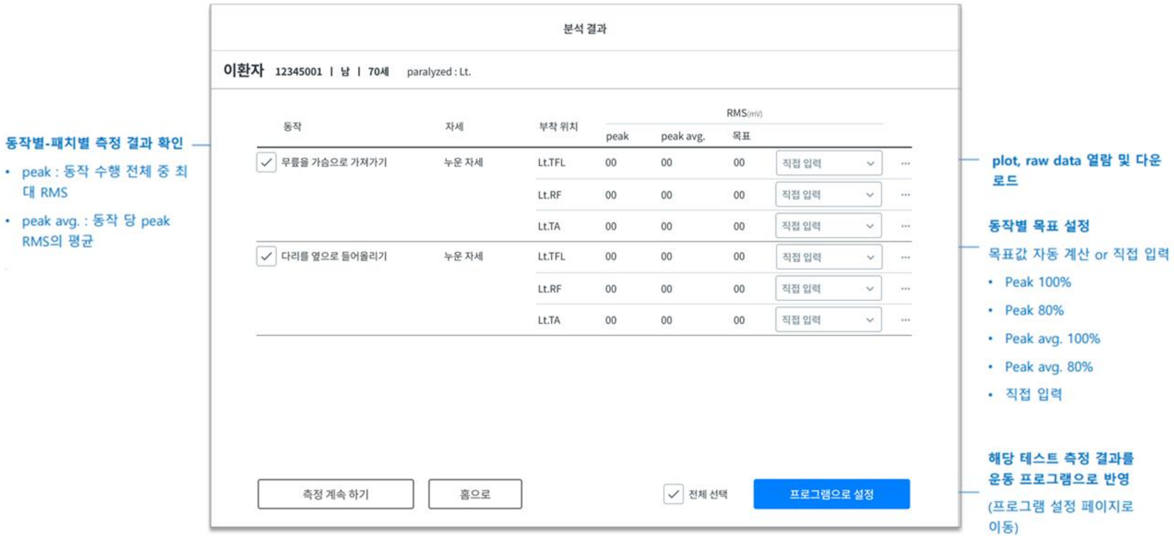

그림 4 신호 측정 후 분석결과 화면

E. 의료진 자가운동 처방

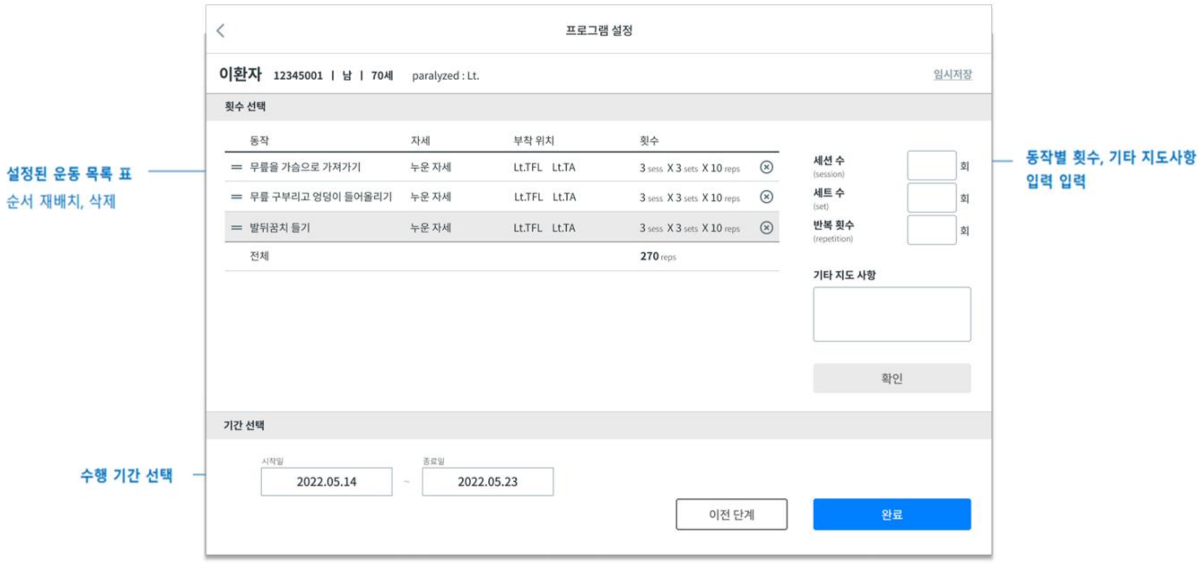

그림 5 의료진 자가운동 처방 화면

F. 환자 분석결과 확인

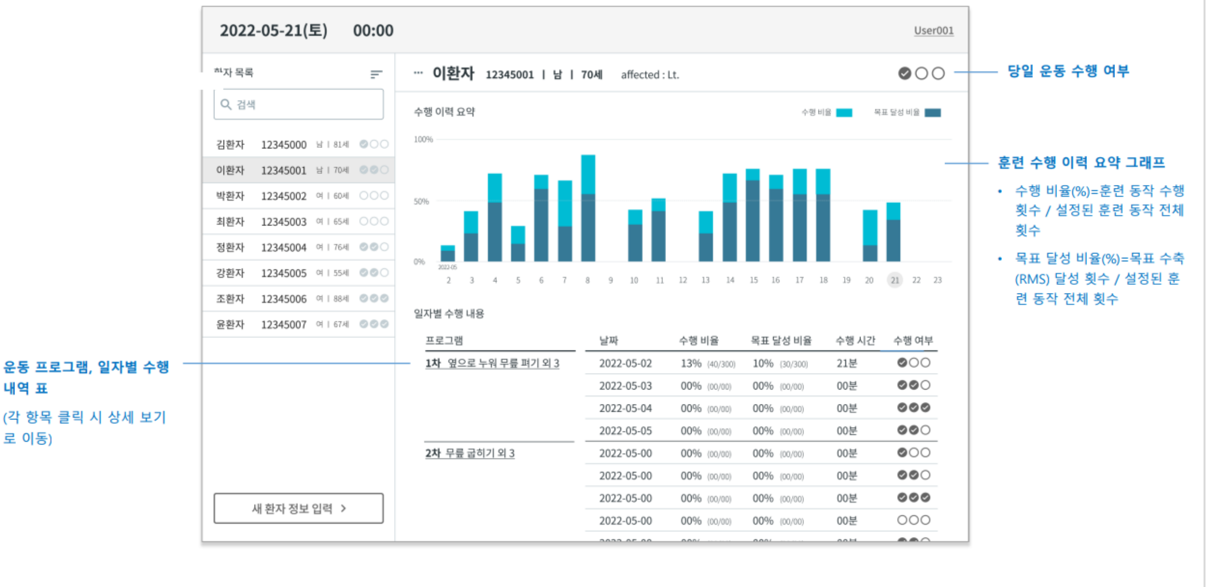

그림 6 분석결과 화면

② 환자 앱

A. 의료진이 ID, 비밀번호 로그인하여 환자와 기기를 선택하여 환자와 기기를 선택하여 매칭

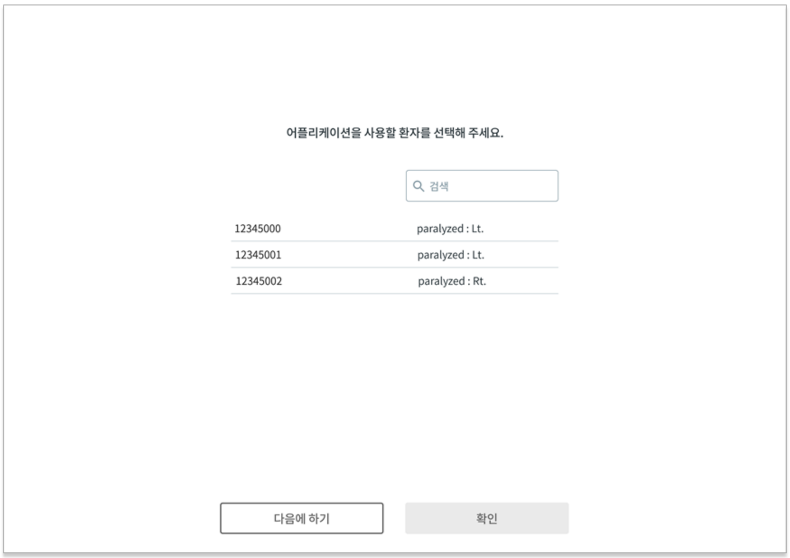

그림 7 로그인 화면

B. 수행해야 할 운동 표시

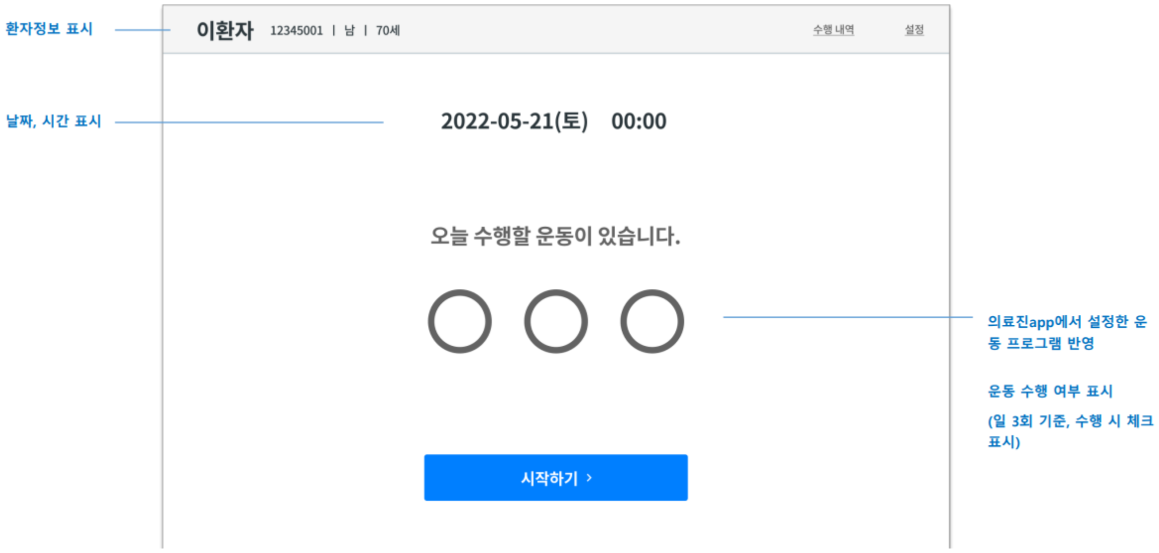

그림 8 운동표시 화면

C. 기기 부착 및 연결

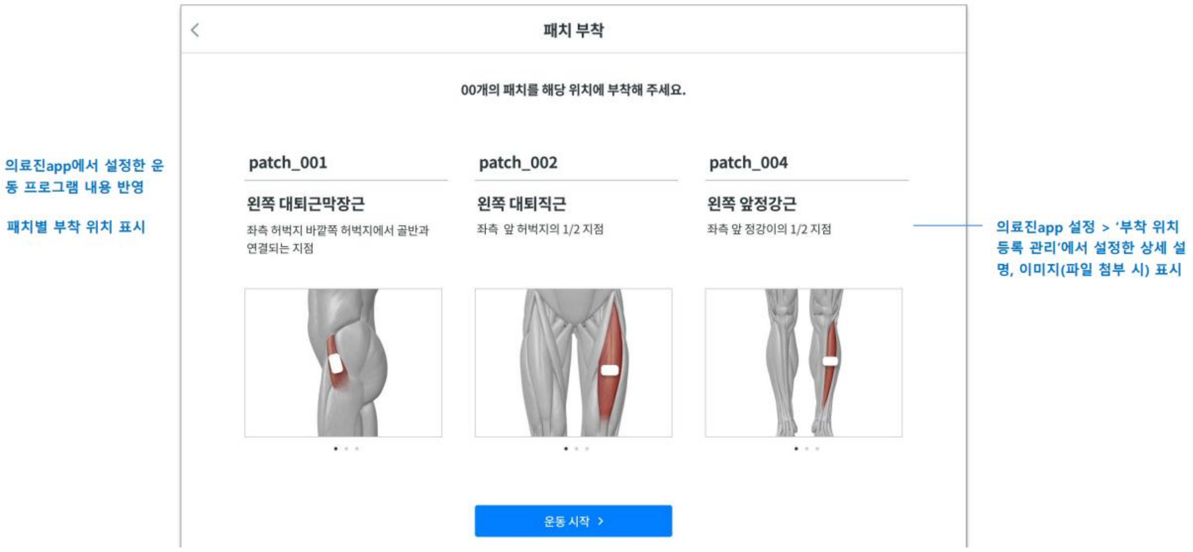

그림 9 기기 부착과 연결 화면

D. 자가 운동 수행

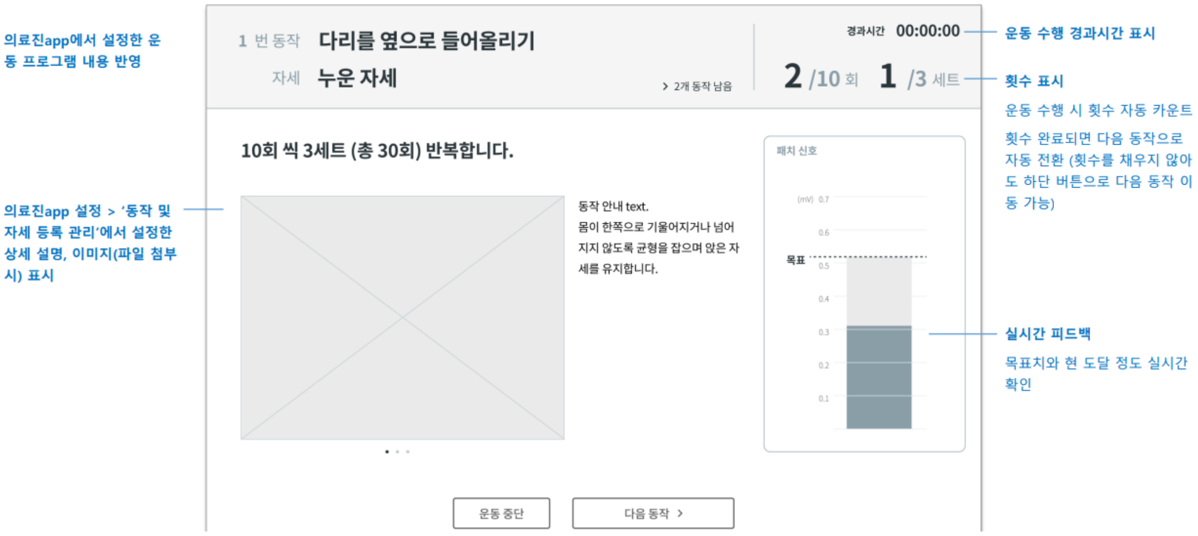

그림 10 자가 운동 수행 화면

E. 재활 자가 운동 완료

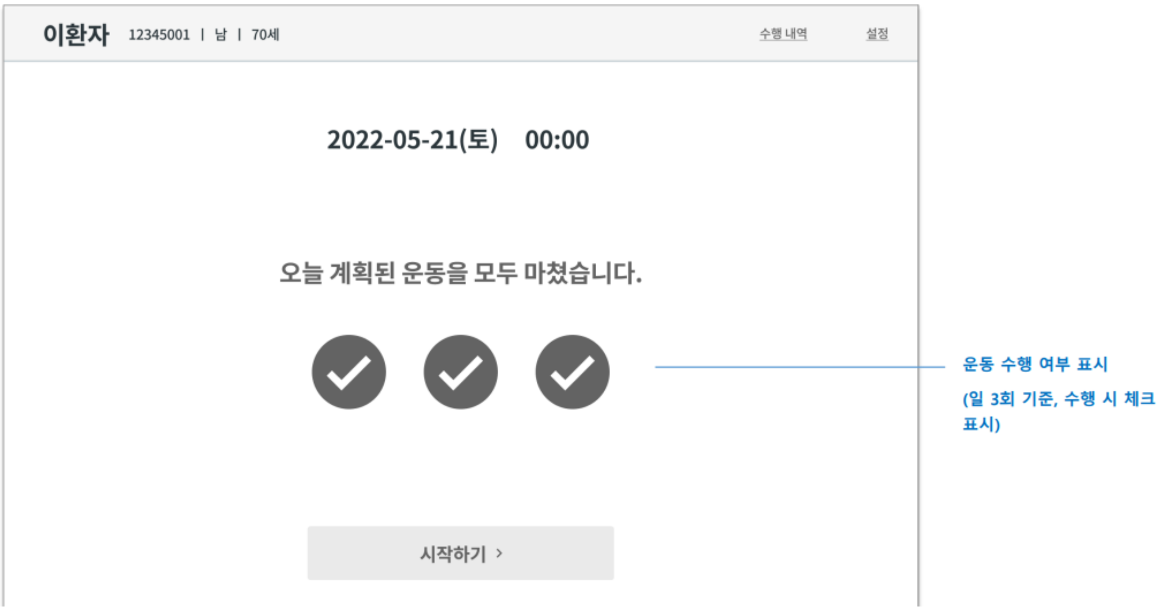

그림 11 자가 운동 완료 화면

4) 성능

(1) 근전도 분석프로그램

① 의료진 앱

A. 환자등록

B. 환자의 목록 표시

C. 프로그램 설정(운동 프로그램 선택, 한 동작의 반복 횟수, 세트수, 하루 몇 회(세션)를 설정)

D. 센서 부착 위치 선정, 좌우 및 목표값 설정

E. 테스트(실시간 신호, 목표값 측정, 위치, 운동가능 여부등 판단)

F. 환자 모니터링

i. 운동 수행내역 (날짜/수행비율/목표달성비율/수행시간/수행여부)확인

ii. 수행비율(운동 수행 횟수 / 프로그램의 세트 * 반복 횟수)

iii. 목표달성율(운동 수행 횟수 중 목표 달성 횟수 / 프로그램의 세트 * 반복 횟수)

② 환자 앱

A. 의사가 환자 목록에서 진행 환자 선택해서 로그인

B. 시작하기 버튼을 누르고 지시된 위치에 패치 붙인 후 기기와 패치를 연결한다.

C. 운동시작 버튼을 누르면 의사가 처방한 재활훈련이 나타난다.

D. 처방된 운동방법으로 운동하고 완료되면 완료 버튼을 누른다.

E. 홈 화면 우측 상단 수행내역을 누르면 모니터링 내용을 확인

i. 날짜/수행비율/목표달성비율/수행시간/수행여부

ii. 수행비율(운동 수행 횟수 / 프로그램의 세트 * 반복 횟수)

iii. 목표달성율(운동 수행 횟수 중 목표 달성 횟수 / 프로그램의 세트 * 반복 횟수)

5) 사용방법

(1) 근전도분석소프트웨어

① 의료진 앱

A. 환자 등록

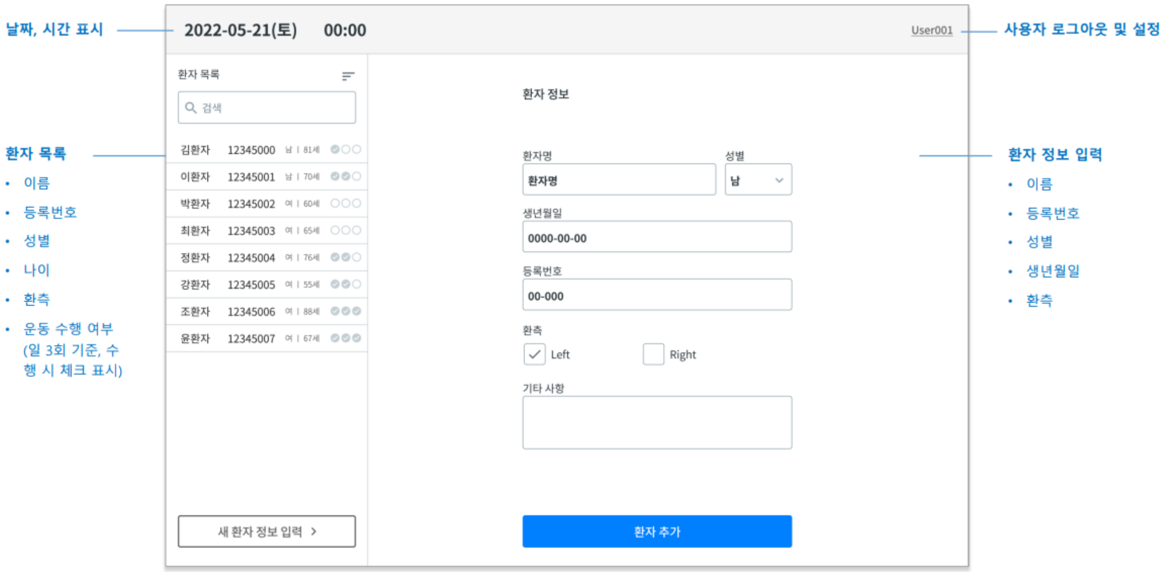

그림 12 환자 등록 화면

B. 패치 연결 테스트

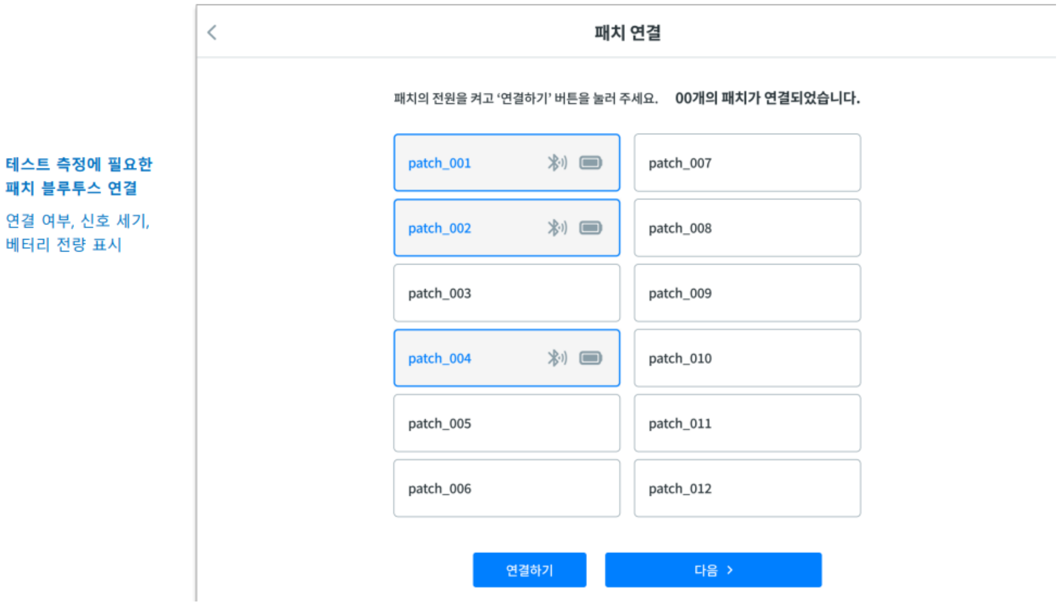

그림 13 패치연결 화면

C. 동작 및 패치 부착부위 선택

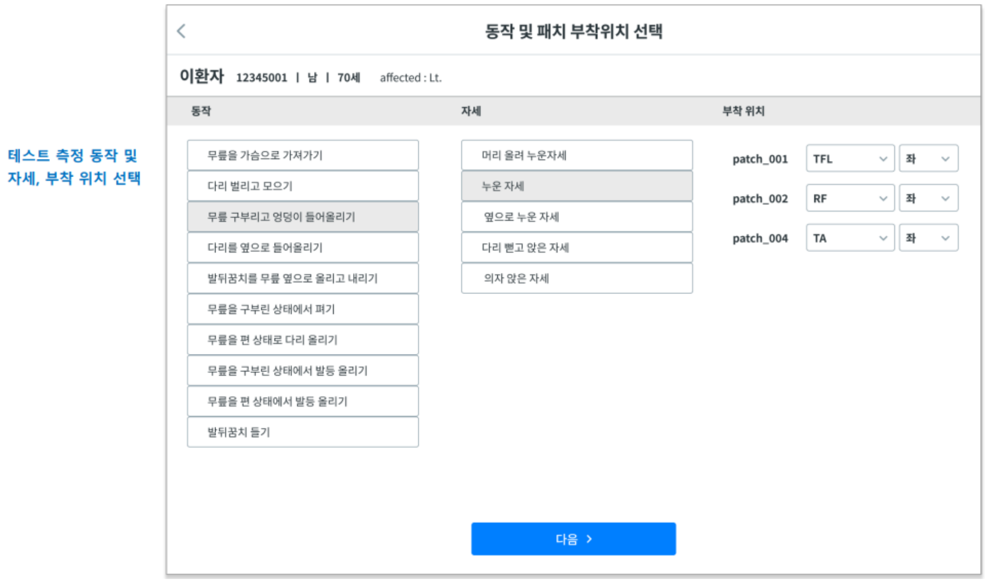

그림 14 동작 및 패치 부착부위 선택 화면

D. 신호 측정 후 분석결과 확인

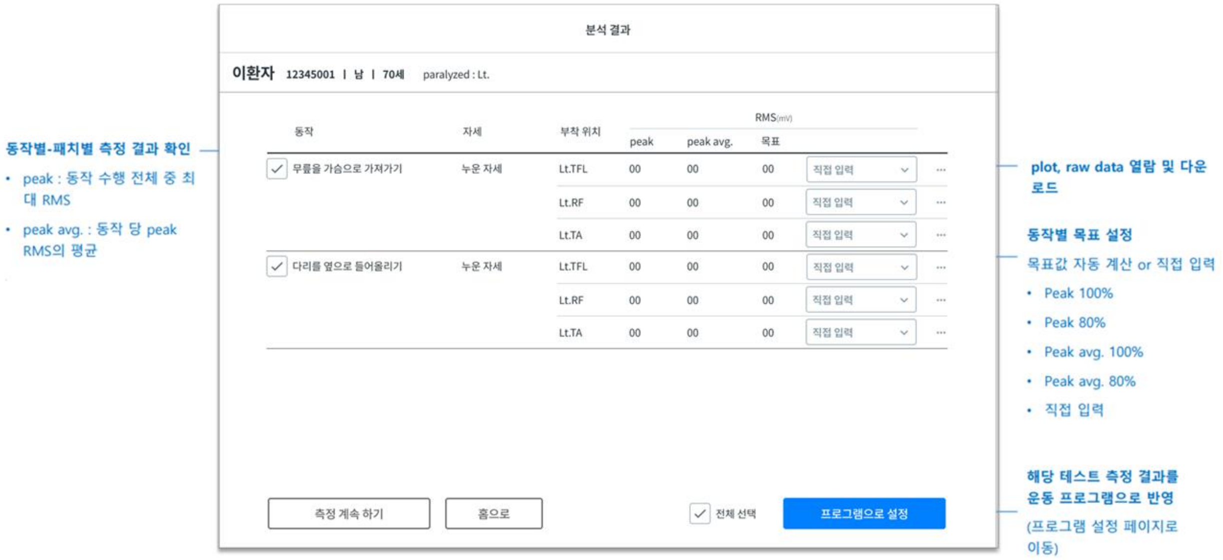

그림 15 분석결과 화면

E. 의료진 자가 운동 처방

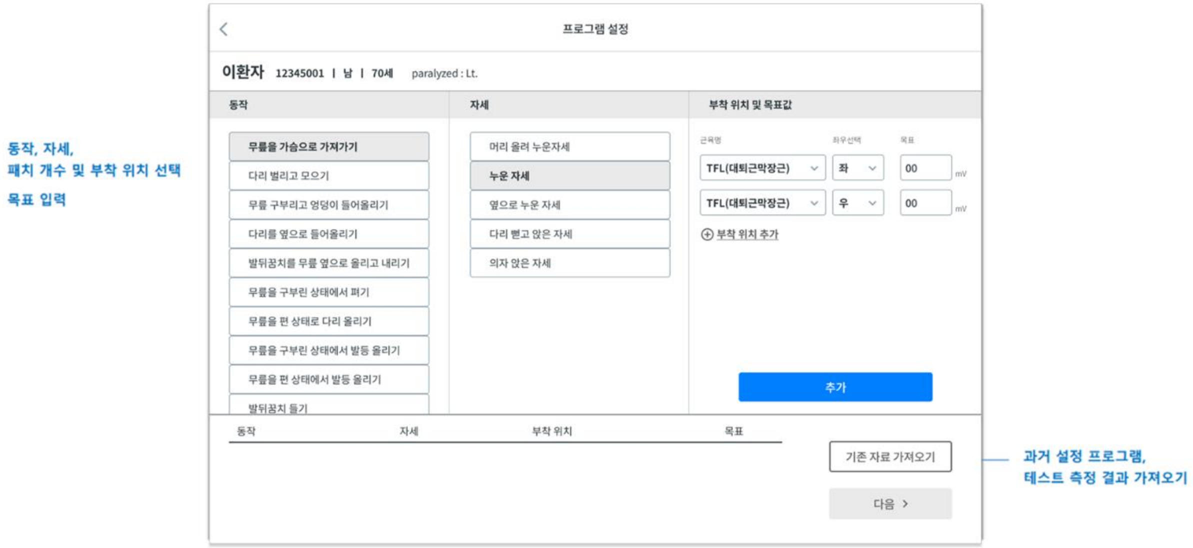

그림 16 동작 및 자세 설정 화면

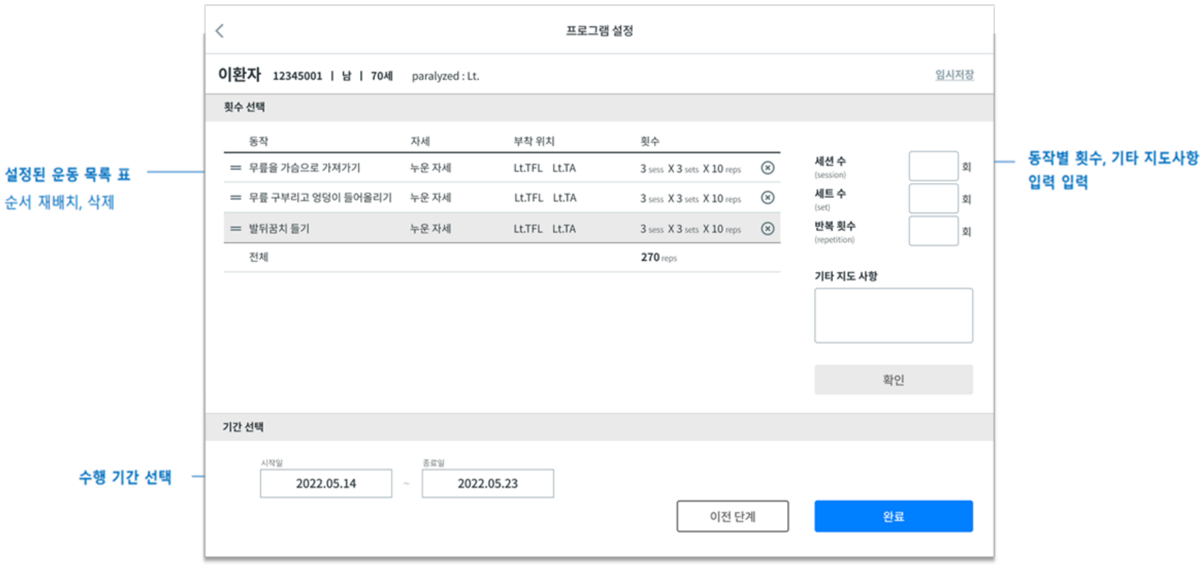

그림 17 세션, 세트, 반복 회수 설정 화면

F. 환자 분석결과 확인

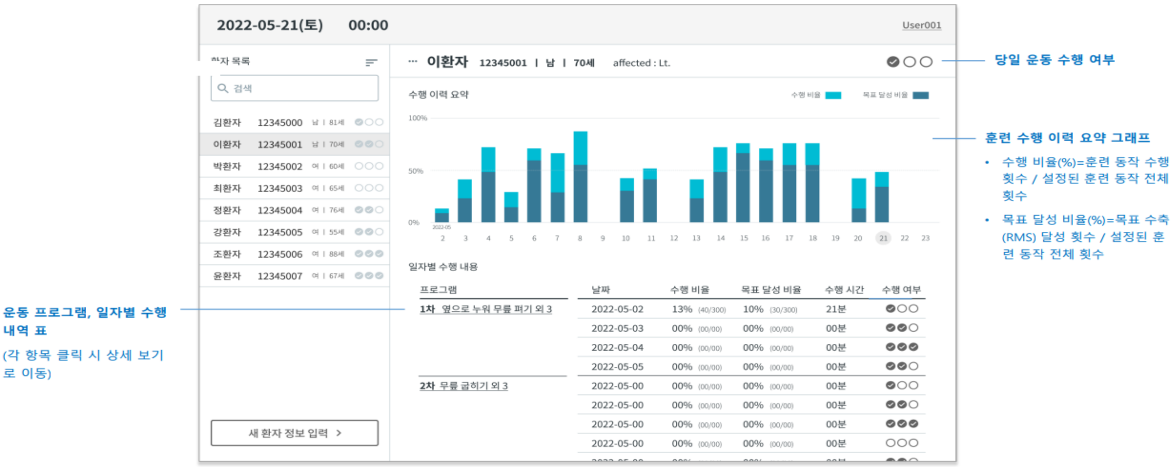

그림 18 분석결과 화면

② 환자 앱

A. 의료진이 ID, 비밀번호 로그인하여 환자와 기기를 선택하여 매칭

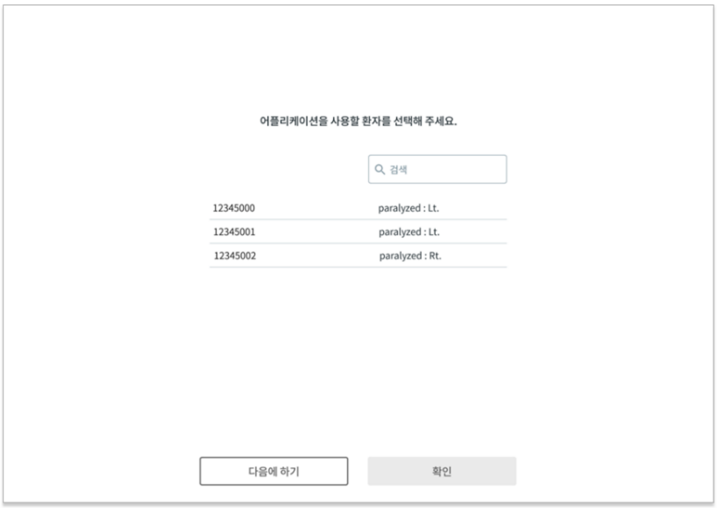

그림 19 로그인 화면

B. 수행해야할 운동 표시

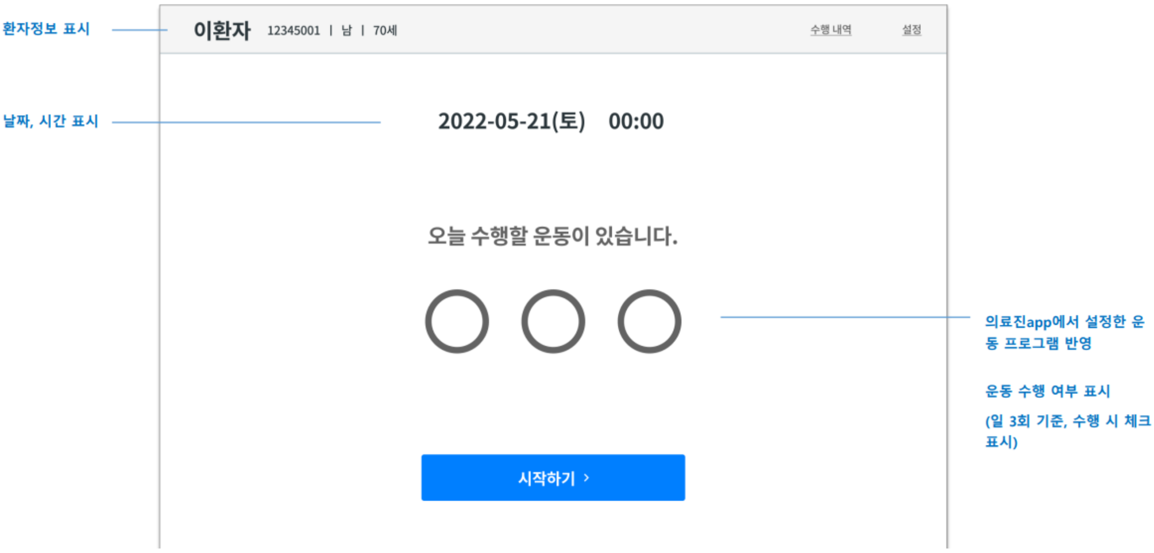

그림 20 수행 운동 표시 화면

C. 기기 부착 및 연결

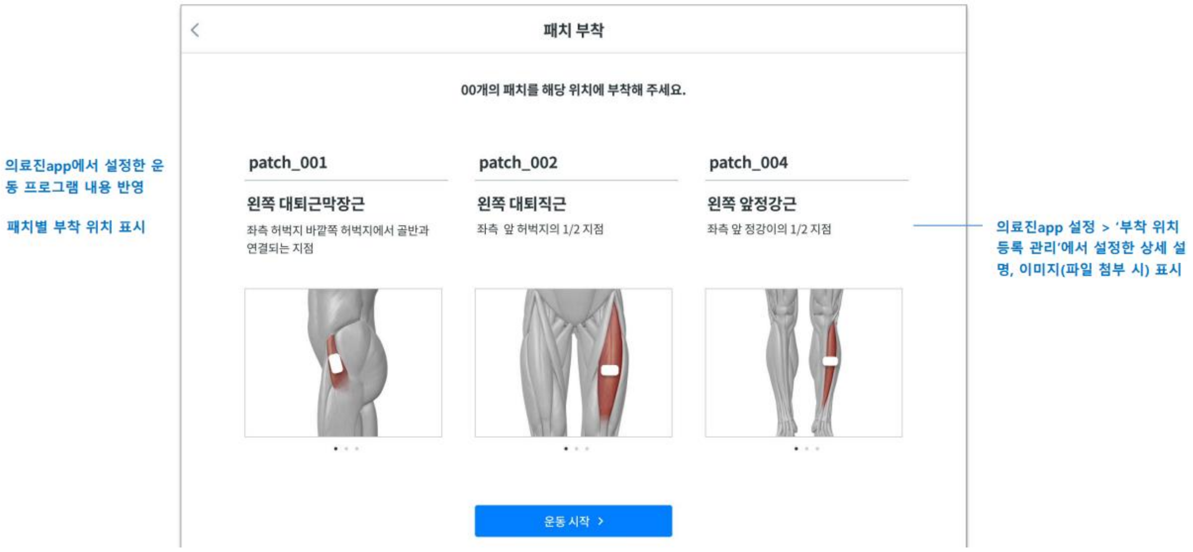

그림 21 기기 부착 및 연결 화면

D. 자가 운동 수행

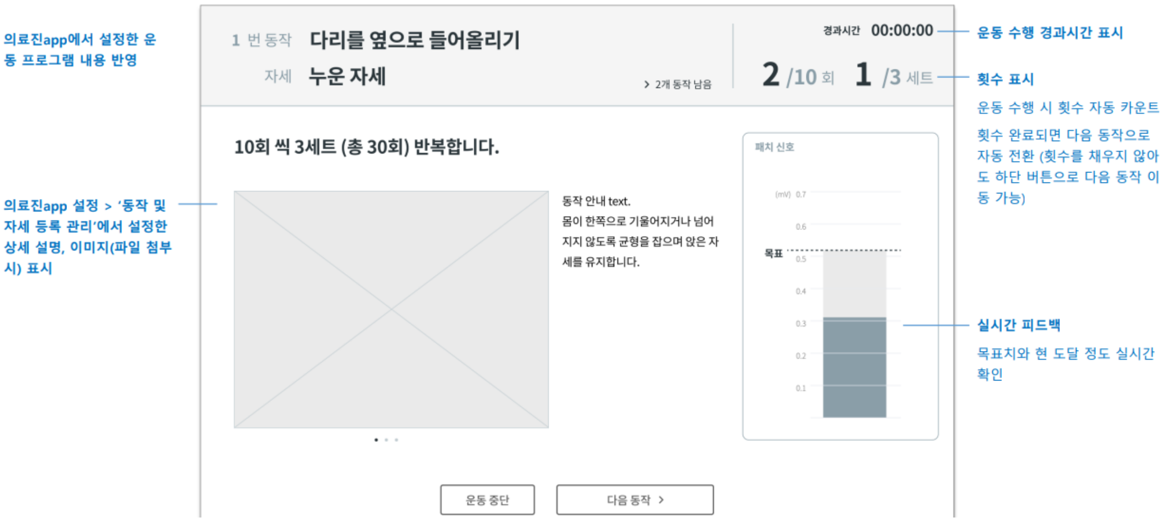

그림 22 자가 운동 수행 화면

E. 재활 자가 운동 완료

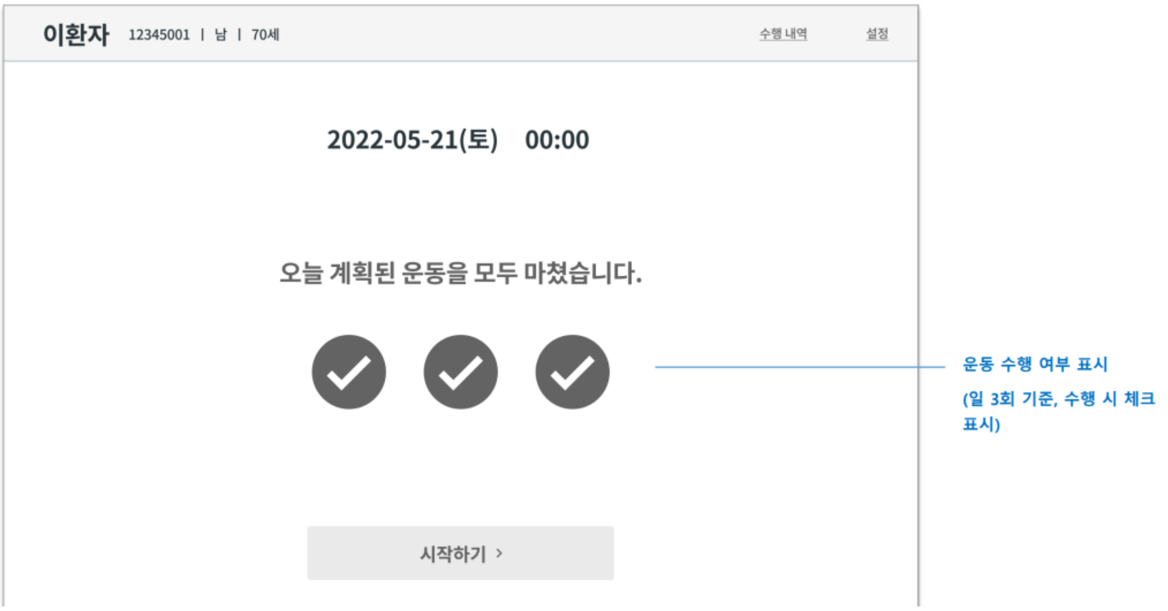

그림 23 자가 운동 완료 화면

6) 사용상 주의사항 및 안전장치

(1) 근전도분석소프트웨어

① 경고

A. 본 제품은 의료소프트웨어로 임의로 본 소프트웨어를 변경하거나 복제 및 불법 유통하는 행위는 치료에 손상을 가하는 행위이므로 본사에 연락하여 해결한다.

② 일반적 주의

A. 본 소프트웨어는 의료기기로 전문가의 교육 및 처방을 받고 사용한다.

③ 안전장치

A. 치료용 범용 소프트웨어

i. 어플리케이션

- 기기와의 연결이 끊어졌음을 표시하고, 재 연결 기능 제공
- 기기 통신보안을 위한 Bonding 기능 제공

ii. 서버

- 의료기기 사이버 보안 요구사항 1
  접근통제 및 인증 기능 제공
- 의료기기 사이버 보안 요구사항 6
  원격접속 차단 기능 제공
- 의료기기 사이버 보안 요구사항 7
  사용자 인증 관리 기능 제공
- 의료기기 사이버 보안 요구사항 11

비밀번호 노출 금지 기능 제공

- 의료기기 사이버 보안 요구사항 15
  네트워크의 의료기기 제어정보 전송 기밀성 및 무결성 보장 기능 제공
- 의료기기 사이버 보안 요구사항 20
  개인의료정보 저장 관리 기능 제공
- 의료기기 사이버 보안 요구사항 21
  사용자가 의료기기에 접속 시 데이터 조회, 생성, 변경, 삭제 등과 같은 로그를 기록하는 기능 제공
- 의료기기 사이버 보안 요구사항 24
  공용 네트워크망에 접속하여 의료기기를 실시간으로 송수신하는 장비의 경우 DDos 공격에 대응할 수 있는 기능 제공

B. 근전도 기기

i. 배터리 팩 내부에서 배터리 셀을 전기적 위험에서 보호

- 과충전 방지 (Over Charge Protection)
  4.28V+/-0.05V 이상의 전압 차단
- 과방전 방지 (Over Discharge Protection)
  2.4V+/-0.1V 이하의 전압 차단
- 과전류 방지 (Over Current Protection)
  150mV+/-30mV 이상의 배터리 전압강하20ms 이내.
- 단락 보호 (Short Protection)
  외부의 단락 된 회로망에서0.5ms 이내에 배터리 셀의 연결을 분리.

ii. 퓨즈

- 충전기 – to – 충전 IC
  임의의 본체 내부 결함에 의해 충전기에서 본체 내부의 충전 IC로 흐르는 과전류를 제한.
- 배터리– to – 본체 내부회로
  임의의 본체 내부 결함에 의해 배터리에서 본체 내부회로로 흐르는 과전류를 제한.

iii. 충전 IC

- 알고리즘에 의한 안정적인 충전 전압, 전류 공급
  4.2V, 최대 256mA, 약 0.57C
- 과열방지 기능
  IC 내부온도가 120 °C 이상으로 상승 시 충전전류를 감소시켜 발열제어

iv. 후크결합을 통해 본체와 패치가 분리되는 것을 방지함

(1) 포장 및 라벨링

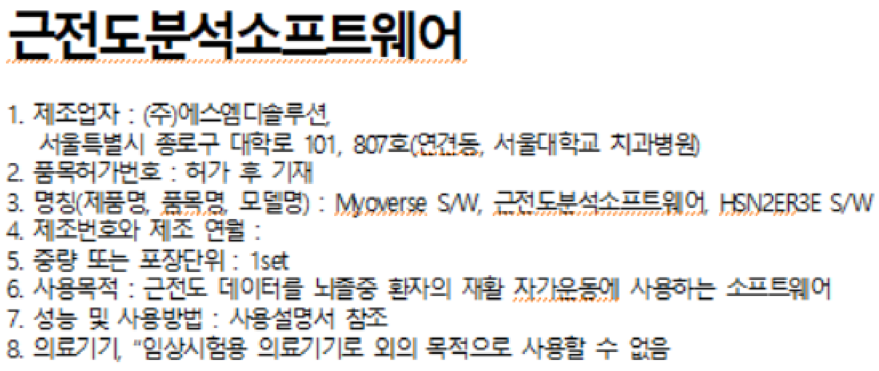
근전도분석소프트웨어

1. 제조업자 : (주)에스엠디솔루션,
   서울특별시 종로구 대학로 101, 807호(연건동, 서울대학교 치과병원)
2. 품목허가번호 : 허가 후 기재
3. 명칭(제품명, 품목명, 모델명) : Myoverse S/W, 근전도분석소프트웨어, HSN2ER3E S/W
4. 제조번호와 제조 연월 :
5. 중량 또는 포장단위 : 1set
6. 사용목적 : 근전도 데이터를 뇌졸중 환자의 재활 자가운동에 사용하는 소프트웨어
7. 성능 및 사용방법 : 사용설명서 참조
8. 의료기기, "임상시험용 의료기기로 외의 목적으로 사용할 수 없음

그림 24 근전도분석 소프트웨어 라벨링

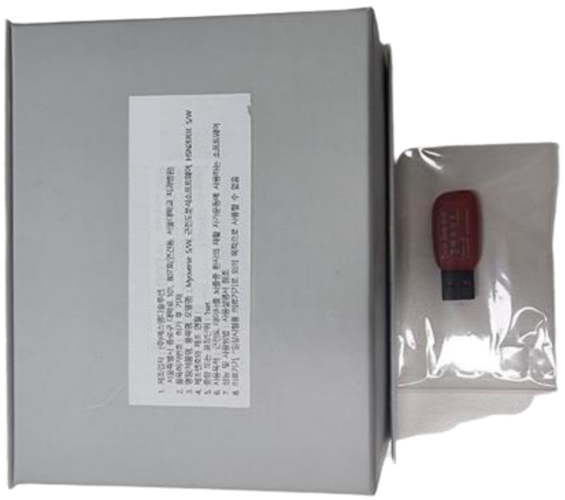

그림 25 근전도분석 소프트웨어 포장

9. 연구대상자의 선정 기준, 제외 기준, 목표한 대상자 수 및 산출 근거

연구대상자는 아래 선정기준에 모두 해당이 되어야 선정될 수 있으며, 제외기준은 어느 하나라도 해당이 된다면 제외됨.

1) 선정기준

① 만 19세 이상

② 뇌졸중 발생 3개월 이내

③ 뇌졸중으로 인한 하지 위약(MMT =< 4 grade)

④ 수정랭킨척도(mRS) 2-5점

⑤ 관절 운동, 균형 및 보행 훈련에 참여할 수 있을 정도의 이해 및 명령에 따를 수 있는 인지 능력

2) 제외기준

① 연구 기간 내 새로운 신경학적 이상을 유발할 정도의 뇌졸중의 재발

② 균형 및 보행 기능에 영향을 줄 정도의 다른 신경학적 이상 (예. 파킨슨 증후군 등)

③ 균형 및 보행 평가 및 수행이 어려울 정도의 인지기능 저하

④ 심각한 내과적 상태 (예. 활동성 암)

⑤ 체내 전기자극기(인공심박기, 심부뇌자극기 등)을 삽입하여 근전도 측정이 어려운 경우

3) 목표한 대상자 수 및 산출 근거

대상자 수: 뇌졸중 환자 24명

산출 근거: 본 연구는 향후 진행될 임상시험의 사전 연구(pilot study)의 성격을 가지고 있으므로 대조군과 신호 양상의 차이를 비교할 수 있을 정도의 최소한의 대상자를 모집할 계획임.

4) 연구 대상자 모집 계획

서울대병원 재활의학과 외래와 병실 게시판, 국립교통재활병원 재활의학과 외래와 병실 게시판에 연구대상자 모집 공고문을 포스터 형태로 인쇄하고 입원 환자 대상으로 홍보하고 경쟁적으로 모집할 예정임. 연구의 책임연구자 및 의뢰기관은 인종이나 사회경제적 상태에 근거해서 이 연구에 참여할 가능성이

있는 환자를 배제시키지 않을 것임. 이 연구의 선정기준에 합당하다면, 가능한 환자들이 이 연구에 참여할 수 있도록 모든 노력을 다할 것이며 본 기관에서 치료받는 뇌졸중 환자의 전체를 대표할 수 있도록 환자들에게 연구의 목적을 주지시킬 것임.

10. 연구 방법

1) 구체적인 연구방법

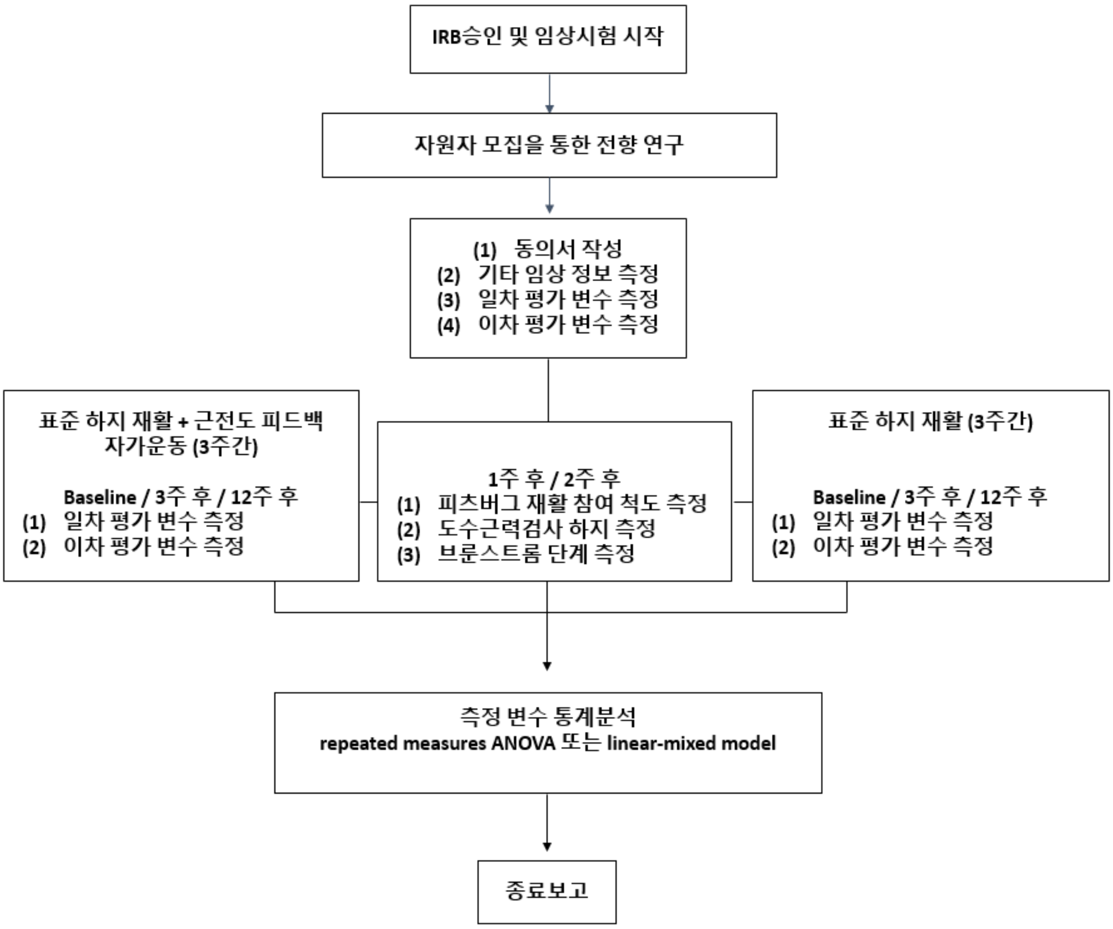

그림 26 연구 흐름도

(1) 동의서 작성

A. 실험에 참여한 자원자는 연구의 목적과 데이터의 활용에 대한 설명을 듣고 사전 검사 및 연구 동의서를 작성하여 대상자 식별코드를 부여 받음(실시기관 코드-실시년도-시험 일련번호-등록된 순).

(2) 임상평가

① 일차 평가 변수

A. 리버미드 운동 평가(Rivermead motor assessment, RMA):
환자 및 보호자와의 면담을 통하여 치료사에 의해 점수화됨[14]. 평가내용은 침대에서 옆으로 누울 수 있는지, 침대에서 일어나 앉을 수 있는지, 서기와 걷기가 가능한지 등이며, 전체 15문항으로 구성되어 있고, 그 동작을 수행하면 1점, 수행하지 못하면 0점을 배점함. 총 점수는 최저 0점, 최고 15점으로 구성됨[15].

② 이차 평가 변수

A. 도수근력검사(Manual muscle test, MMT): 근력 측정 검사로서 중력과 저항에 대한 근력에 따라 0-5등급으로 평가되는 척도임. 본 연구에서는 양측 고관절 굴곡, 고관절 외전, 무릎관절 신전, 족관절 배측굴곡 근력이 측정됨.

B. 브룬스트롬 기능회복 단계(brunnstrom stages of motor recovery, BRS)
브룬스트롬 운동 회복 단계는 뇌졸중 후 편마비 환자의 움직임 조절을 통한 회복과정을 질적으로 평가한 항목으로 상지와 손 그리고 하지의 운동회복과정을 6단계로 구분함.

C. 푸글마이어 척도-하지(Fugl-Meyer assessment of lower extremity, FMS-LE)
푸글마이어 평가척도는 뇌졸중 이후의 운동기능 회복단계를 기초로 하여 뇌졸중 생존자의 기능적 회복 정도를 양적으로 평가하는 척도임. 평가도구는 0점(수행할 수 없음)에서 2(완전하게 수행)의 3점의 서열척도(Likertscale)이며 최대점수는 226점으로, 5개 하위항목인 운동기능(상지66점, 하지34점), 감각기능(24점), 균형(14점), 관절가동범위(44점), 그리고 관절통증(44점)으로 구성됨[16]. 본 연구에서는 하지 34점을 사용할 것임.

D. 버그균형척도(Berg balance scale, BBS)
버그균형척도는 14개의 항목으로 구성되어 크게 앉기, 서기, 자세변화의 3개영역으로 구분됨. 각 항목은 최소 0점에서 최고 4점으로 측정되고 14개 항목에 대한 총합은 56점으로 계산됨. 앉기 항목은 의자의 등받이에 기대지 않고 바른 자세로 앉기, 서기 항목으로는 잡지 않고 서 있기, 두 눈을 감고 잡지 않고 서 있기, 두발을 붙이고 잡지 않고 서 있기, 한 다리로 서 있기, 왼쪽과 오른쪽으로 뒤돌아보기, 바닥에 있는 물건을 집어 올리기, 한 발 앞에 다른 발을 일자로 두고 서 있기, 선 자세에서 앞으로 팔을 뻗쳐 내밀기, 자세변화 항목으로는 앉은 자세에서 일어나기, 선 자세에서 앉기, 의자에서 의자로 이동하기, 제자리에서 360°회전하기, 일정한 높이의 발판 위에 발을 교대로 놓기로 구성됨[17].

E. 기능적 보행 분류(Functional ambulation category, FAC)
기능적 보행 분류는 최소 0점에서 최고 5점을 적용한 6가지 보행기능 수준으로 구성되어 있음. 다음과 같이 기능적 보행 능력을 6가지로 분류됨. 보행기능 수준은 0점 불능 보행, 1 점 비 기능적 보행, 2 점 집안 내 보행, 3 점 이웃 근접 보행, 4 점 독립적 지역사회내의 보행, 5 점 정상 보행으로 구성됨[18].

F. 수정 랭킨 척도(modified Rankin scale, mRS)
뇌졸중 환자의 일상활동에서 장애 정도 혹은 의존도의 정도를 평가하는 데 흔히 이용되는 척도로 mRS 점수는 0~6점으로 정량화 됨. 아무런 증상이 없을 때 0점, 발병 전의 모든 업무와 일상활동의 실행이 가능한 경우는 1점, 발병 전의 모든 일상활동

을 수행할 수는 없지만 도움이 없이 스스로 일상적 용무는 처리할 수 있는 경도의 장애는 2점, 약간의 도움이 필요하지만 다른 사람의 부축을 받지 않고 걸을 수 있는 중등도의 장애는 3점, 보조나 다른 사람의 도움이 없이는 보행이 불가능하고 대소변 처리와 위생관리를 스스로 수행할 수 없는 중등도의 심한 장애는 4점, 침상의존상태이고 대소변실금이 있으며 지속적인 간호와 주의가 필요한 중증장애는 5점, 사망한 경우는 6점으로 배점함[19].

G. 수정 바델 지수(modified Barthel index, MBI)

10개 항목으로 구성되어있으며, 환자의 상태에 따라 5단계의 점수를 배점함. 제 1단계는 다른 사람의 도움에 완전히 의존하는 경우, 제 2단계, 3단계, 4단계는 정도에 따라 다른 사람의 도움이 필요 한 경우 이며, 제 5단계는 다른 사람 도움 없이 혼자서 일상생활을 할 수 있는 경우로 최고 점수는 100점이며, 최저 점수는 0점으로 점수가 높을수록 독립성이 높음 을 의미함[20].

H. 피츠버그 재활 참여 척도(Pittsburgh Rehabilitation Participation Scale, PRPS)

환자의 운동 참여 정도를 평가하는 척도로 전혀 참여하지 못하면 1점, 참여자의 거절이나 절반 이하의 참여도 2점, 참여는 하였으나 운동을 끝마치지 못함 3점, 참여자가 대부분의 운동을 끝냈지만 모든 운동을 끝내지 못하면 4점, 참여자가 모든 운동에 최대노력으로 참여하고 모든 운동을 끝냄 5점, 참여자가 모든 운동에 최대노력으로 참여하고 모든 운동을 끝냈으며 적극적 흥미를 보임 6점으로 배점됨[21].

I. Short-form Health Survery 36 version 2(SF-36v2)

SF-36은 36개의 문항으로 구성된 다차원적 일반적 건강 프로필 건강 관련 삶의 질 측정 도구로서, 어떤 특정 연령, 질환 상태, 또는 치료 그룹 등을 목표로 한 것이 아닌 전반적인 건강 상태를 측정할 수 있는 일반적 측정도구로 개발됨. 36개의 항목, 8개의 척도, 신체적 요소 요약 및 정신적 요소 요약의 2개의 요약 측정, 전반적 건강으로 구성됨. SF-36의 점수는 자료를 변환시켜 얻어지는 0점(가장 나쁜 건강상태)부터 100점(가장 좋은 건강상태) 사이의 한 값으로 나타나게 되며, 8개의 척도값, 2개의 요약 측정값 및 전반적 건강값 등을 사용하여 대상자의 건강 관련 삶의 질을 측정함[22].

(3) 센서 신호 측정

① 패치 부착 부위

A. 가쪽넓은근 (Vastus lateralis) – 무릎 슬개골(patella) 가쪽에서 위앞엉덩뼈가시(anterior superior iliac spine)로 선을 그었을 때 약 1/2 지점에서 근육이 가장 잘 만져지는 지점

B. 앞정강근 (tibialis anterior) – 종아리뼈 머리(fibular head)에서 안쪽 복숭아뼈(medial

malleolus)로 선을 그었을 때 약 1/3지점에서 근육이 가장 잘 만져지는 지점

② 센서 신호 파라미터

각 근육별 근전도 신호의 진폭(amplitude, mV), 최대자발수축(maximal voluntary contraction) 진폭 대비 실제 수축시 진폭의 비율(%)

③ 센서 신호를 활용한 운동 특징

근육 수축 횟수, 목표 횟수 대비 근육 수축 횟수 비율, 근전도 신호 진폭의 목표 달성 횟수 및 비율, 운동 수행 시간, 수행 여부 등

(4) 기타 정보

① 임상 정보

A. 인구학적 정보: 나이, 성별, 우세손, 타질환 동반 유무(고혈압, 당뇨, 고지혈증, 심방세동, Charlson 동반질환지수(Charlson comorbidity index)), 교육수준(초등학교/중학교/고등학교 졸업 여부), 돌봄제공자(caregiver, 배우자(spouse)/자녀(children)/간병인/기타)

B. 뇌졸중 관련 정보: 초기 NIH 뇌졸중 척도 (National Institutes of Health Stroke, NIHSS), 뇌졸중 치료(thrombolysis/ thrombectomy), 뇌졸중 발생으로부터 기간, 뇌졸중 종류(경색/출혈), 뇌졸중 위치(우측/좌측, 천막상부/천막하부, 운동영역피질(M1)침범/내포(internal capsule)침범), 뇌졸중 원인(TOAST classification: large-arteryAtherosclerosis/ small-vessel occlusion/ Cardioembolism/ other determined etiology/ Undetermined etiology)

② 약제: 항경직약, 항우울제, 항경련제 등

③ 인지기능: 간이정신상태검사(MMSE)

④ 우울 및 불안: 병원 불안-우울 척도(Hostpial Anxiety and Depression Scale, HADs)

⑤ 보조기: 무릎 보조기(knee stabilizer), 발목보조기(ankle-foot orthosis)

⑥ 운동유발전위검사(Motor Evoked Potential, MEP): 경두개 자기자극술을 건측과 환측의 두부(10-20 system의 C1-C2 부위)에 제공하며, 기록 전극은 하지의 abductor hallucis 근육에 부착하여 파형을 확인함. 휴식 운동 역치(resting motor threshold)는 최소 50 uv 이상의 진폭(amplitude)의 파형이 10회 중 5회 이상 관찰되는 시점의 자극 강도로 정의됨.

(5) 치료 프로토콜

① 표준 하지재활

A. 치료 내용: 뇌졸중 환자에서 하지 운동기능 저하에 대한 일반적인 재활치료에 해당하며 주로 관절범위운동(range of motion)과 함께 기능적 이동성에 입각하여 수행됨. 누웠다가 앉기, 서기, 걷기, 균형 훈련 등이 이에 포함되고 환자의 상태에 따라 치료

사가 부축 하에 또는 보행보조기기(예. 보행기, 지팡이, 하지 보조기 등)를 활용하여 치료가 제공될 수 있음. 세션 당 30분, 1일 2회, 주 5일 3주동안 제공됨.

B. 제공자: 병원 치료사

C. 단계 조절: 치료사의 판단 하에 운동의 유형, 강도, 속도 등을 조절하여 운동기능이 저하된 뇌졸중 환자에게 적절한 수준의 운동이 제공될 수 있도록 함.

② 자가운동

A. 운동 내용

i. 뇌졸중 환자가 물리치료사가 대동한 상태가 아닌, 스스로 또는 보호자의 도움을 받아 침상에서 하지에 대한 운동을 수행함.

ii. 시험군은 근전도를 통해 획득된 데이터 및 이를 활용한 피드백에 기반한 감독하 자가운동을 수행함.

iii. 운동 종류는 침상에서 수행할 수 있는 기능적 이동성에 기반한 다양한 운동으로 구성되며 각 운동 수행 방법은 하기와 같음.

A. 무릎을 가슴으로 가져가기: 무릎을 구부려 가슴으로 가져감

B. 다리 벌리고 모으기: 다리를 편 상태로 벌리고 모음

C. 무릎 굽힌 상태에서 무릎 펴기: 무릎을 굽힌 상태에서 무릎을 폄

D. 무릎 굽힌 상태로 발등 올리기: 무릎을 굽힌 상태에서 발등만 올림

E. 엉덩이 들어올리기: 무릎을 굽힌 상태에서 엉덩이를 들어올림

F. 고관절 굽히면서 무릎 굽히고, 고관절 펴면서 무릎 펴기: 다리를 들어올리며 무릎을 굽힘, 다리를 펴면서 무릎을 폄

G. 무릎을 가슴으로 가져가며 발등 올리기: 무릎을 구부려 가슴으로 가져가면서 발등을 올림

H. 무릎 편 상태로 발뒤꿈치 들기: 무릎을 편 상태로 발등을 내림

I. 무릎 편 상태로 다리를 옆으로 들어올리기: 다리를 편 상태로 옆으로 들어올림

J. 무릎 편 상태로 다리 올리기: 다리를 편 상태로 들어올림

K. 고관절 편 상태로 무릎 굽히고 펴기: 다리를 편 상태에서 무릎만 굽히고 폄

L. 무릎 편 상태로 발등 올리기: 무릎을 편 상태로 발등만 올리기

M. 무릎 굽힌 상태로 발뒤꿈치 들기: 무릎을 굽힌 상태로 발등을 내림

N. 제자리 걷기: 제자리에서 걷기

B. 제공자: 연구 치료사-A (단, 일반적인 경우 자가운동 수행 중에 대동하지 않으며, 1주일에 1회 근전도 데이터 기반 피드백 내용에 따라 운동의 종류를 변경하고 이에 대한 추가 설명을 제공함)

C. 분류 및 운동 프로토콜

A. 원칙

i. 근력 및 브룬스트롬 단계의 상태에 따라 뇌졸중 환자의 등급(Level)과 단계(Step)를 결정하며, 이에 따라 운동 처방 내용이 의료진/치료사에 의해 결정된다

ii. 등급과 단계는 1주일마다 의료진/치료사가 근력 및 브룬스트롬 단계를 확인하여 결정하며, 이에 따라 운동 처방 내용이 변경될 수 있다

iii. 운동 종류는 1주일 기간 동안 최대 4가지까지 선택될 수 있고, 가능한 높은 등급(Level)에 해당하는 운동이 추천되며, 이는 환자의 상태를 고려하여 의료진/치료사에 의해 결정된다

iv. 환자의 상태에 따라 등급(Level) 또는 단계(Step)가 중복될 수 있으며, 가능한 높은 등급(Level)에 해당하는 운동이 추천되며, 이는 환자의 상태를 고려하여 의료진/치료사에 의해 결정된다

v. 운동을 안전하고 효과적으로 수행할 수 있는 자세를 환자의 상태를 고려하여 의료진/치료사가 결정하며, 가능한 기능적 이동성이 높은 자세가 추천된다

vi. 운동의 종류, 세션(/일), 세트(/세션), 반복횟수(/세트) 및 근수축시 근전도 신호의 진폭에 대한 목표 수치 정보가 환자에게 제공되어야 하며, 일반적으로 운동은 2-4가지/세션, 2-3세션/일, 3-5세트/세션, 5-10회/세트, 진폭은 최대수축시 진폭의 50% 이상을 추천하며, 이는 환자의 운동 수행 정도에 따라 의료진/치료사에 의해 결정된다

vii. 하지 운동 수행 전 준비운동으로 체간 균형 운동을 10분간 시행하는 것이 추천된다

viii. 근전도 센서는 최대 2개 이하로 사용하며 운동 수행에 있어 주된 움직임을 가장 잘 반영하는 환측 근육에 부착하며, 의료진/치료사에 의해 부착 위치가 표시되는 것이 추천된다(단, 운동 수행시 나타나는 근전도 신호 양상의 확인을 건측 근육에서 미리 확인해야 할 수 있다)

B. 프로토콜 내용

i. 분류 프로토콜

등급과 단계는 브룬스트롬 단계와 하지근력을 바탕으로 결정되며, 총 3등급으로 나뉘고, 각 등급은 다시 3단계로 나뉨

Level(등급)	Step(단계)	LE Motor Power (하지근력)			Brunnstrom stage – LE (브룬스트롬 단계-하지)
		HF (고관절 굽힘근)	KE (무릎관절 폄근)	ADF (발목배측굴곡근)	
1	A	≤2	≤1	≤1	1-2
	B	≤2	≤2	≤1	
	C	≤2	≤2	≤2	
2	A	≥2			2-3
	B		≥2		
	C			≥2	
3	A	≥3			3-6
	B		≥3		
	C			≥3	

표 6 운동강도 분류

ii. 운동 프로토콜

등급과 단계에 따라 운동종류가 결정됨

Level (등급)	Step (단계)	Exercise(운동)													
		1. 무릎을 가슴으로 가져가기	2. 다리 벌리고 모으기	3. 무릎 굽힌 상태에서 무릎 펴기	4. 무릎 굽힌 상태로 발등 올리기	5. 엉덩이 들어올리기	6. 고관절 굽히면서 무릎 굽히고, 고관절 펴면서 무릎펴기	7. 무릎을 가슴으로 가져가며 발등 올리기	8. 무릎 편 상태로 발뒤꿈치 들기	9. 무릎 편 상태로 다리를 옆으로 들어올리기	10. 무릎 편 상태로 다리 올리기	11. 고관절 편 상태로 무릎 굽히고 펴기	12. 무릎 편 상태로 발등 올리기	13. 무릎 굽힌 상태로 발뒤꿈치 들기	14. 제자리 걷기
1	A	O	O												
	B	O	O	O											
	C	O	O	O	O										
2	A	O	O	O	O	O									
	B		O		O	O	O								
	C		O			O	O	O	O						
3	A		O			O		O	O	O	O				
	B		O			O				O	O	O			
	C		O			O				O	O	O	O	O	O

표 7 강도별 운동종류

iii. 자세

1. 각 자세에 대한 설명

No.	Body Position	몸 자세(신체 위치)	설명	기타 설명
1	Half-lying Position	반 누운 자세	바로누운상태에서 상체를 30-45도 들어올린 자세	바로누운상태에서 어느 정도 상반신을 일으킨 자세
2	Supine Position	바로 누운 자세	똑바로 하늘(천정)을 보고 누운 자세	
3	Side-lying Position	옆으로 누운 자세		
3-1	- Right	오른쪽 옆으로 누운 자세	오른쪽을 아래로 하고 옆으로누운자세	
3-2	- Left	왼쪽옆으로 누운 자세	왼쪽을 아래로 하고 옆으로 누운 자세	
4	Long Sitting Position	다리뻗고 앉기 자세		등을 지지하지 않고 길게 앉은 자세
5	Sitting Position	의자 앉기 자세	앉음(앉은) 자세	침대(침상) 가장자리에 걸터앉은 자세

표 8 자세 설명

2. 운동시 가능한 자세(O)와 불가능한 자세(X) 표시

No.	Exercise(운동)	Postural setting(자세 세팅)				
		Half-lying (반 누운 자세)	Supine (바로 누운 자세)	Side-lying (옆으로 누운 자세)	Long-Sitting (다리뻗고 앉기 자세)	Sitting (의자 앉기 자세)
1	무릎을 가슴으로 가져가기	O	O	O	O	O
2	다리 벌리고 모으기	O	O	X	O	O
3	무릎 굽힌 상태에서 시작 무릎 펴기	O	O	O	O	O
4	무릎 굽힌 상태로 발등 올리기	O	O	O	O	O
5	엉덩이 들어올리기	O	O	X	X	X
6	고관절 굽히면서 무릎 굽히고, 고관절 펴면서 무릎펴기	O	O	O	O	O
7	무릎을 가슴으로 가져가며 발등 올리기	O	O	O	O	O
8	무릎 편 상태로 발뒤꿈치 들기	O	O	X	O	O
9	무릎 편 상태로 다리를 옆으로 들어올리기	X	X	O	X	X
10	무릎 편 상태로 다리 올리기	O	O	X	O	O
11	고관절 편 상태로 무릎 굽히고 펴기	X	X	O	X	X
12	무릎 편 상태로 발등 올리기	O	O	O	O	O
13	무릎 굽힌 상태로 발뒤꿈치 들기	O	O	O	O	O
14	제자리 걷기	O	O	X	O	O

표 9 자세별 운동

D. 치료 시간

i. 자가운동 프로토콜을 기준으로 환자 상태에 따라 사용량을 점진적으로 증가시키고자 함.

ii. 자가운동 1-2일 째는 하루 1회, 이후에 하루 2회 적용.

iii. 한 세션 당 30분 이상, 하루 총 2세션 이상 수행, 주 5일간 3주 동안 시행.

iv. 총 자가운동 시간: 60분

v. 자가운동 진행 중 부작용에 해당하는 중대한 문제가 발생하지 않으면 횟수를 증가시키고, 중대한 문제가 발생할 경우 의료진 상의 하에 횟수를 감소시킴.

E. 피드백

i. 근수축 활성화 여부 및 정도 확인: 운동수행 시 근수축을 하고자 하는 근육에 부착된 센서를 통해 근수축의 정도를 정량적으로 확인 가능하며, 근수축의 여부 및 정도에 대한 정보를 제공함.

ii. 세션 당 근수축의 횟수 및 운동 시간 확인: 운동의 밀도를 확인하여 운동의 양에 대한 정량적 정보를 제공하며 이는 자가운동의 순응도 확인에 사용될 수 있음.

iii. 주 1회 연구원이 운동 수행의 결과를 확인하여 자가운동 프로그램을 선택하여 알려주고 이에 대하여 설명함.

2) 대조군 설정 및 무작위 배정 방법

① 대조군: 표준 하지재활

② 시험군: 표준 하지재활 + 근전도 피드백에 기반한 감독하(supervised) 자가운동 제공

③ 무작위배정

A. 전체 대상자를 대조군과 시험군에 1:1의 비율로 배정할 예정임. 공개 임상시험이고 단일 맹검으로 연구참여자는 본인이 대조군 또는 시험군에 배정이 되었는지를 확인할 수 있으나 평가를 수행할 평가자는 군 배정 정보에 대하여 맹검이 시행됨.

B. 층화 무작위배정(stratified randomization)

i. 층화 무작위배정의 기준: 나이(<65/≥65), 초기 mRS(2-3/4-5)

C. 무작위배정 방법

i. 서울대학교병원 의학연구협력센터에 의뢰하여 웹 무작위배정프로그램 생성

ii. 각 기관 무작위배정 담당자가 스크리닝 번호, 성별, 동의취득일, 생년월일, 선전기준, 제외기준 입력

iii. 프로그램에 의해 층화 무작위배정

3) 시험 의료기기 사용 계획

① 의료기기 사용기간

A. 임상시험에 참여하여 하지재활을 개시한 이후 3주 시점까지

② 전체 의료기기 사용량

A. 자가운동 1-2일 째는 하루 1회, 이후에 하루 2회 적용

B. 자가운동 1회 당 소요 시간은 30분, 하루 총 2회의 자가운동 시행을 한다고 하였을 때 총 적용 시간은 60분 소요 예상

③ 의료기기 사용방법

A. 제품 착용 방법

i. 필름제거

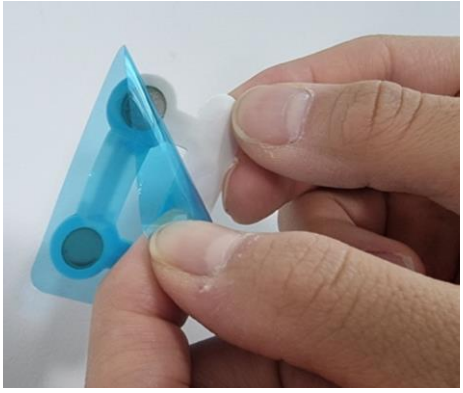

그림 27 패치 필름제거

ii. 부착

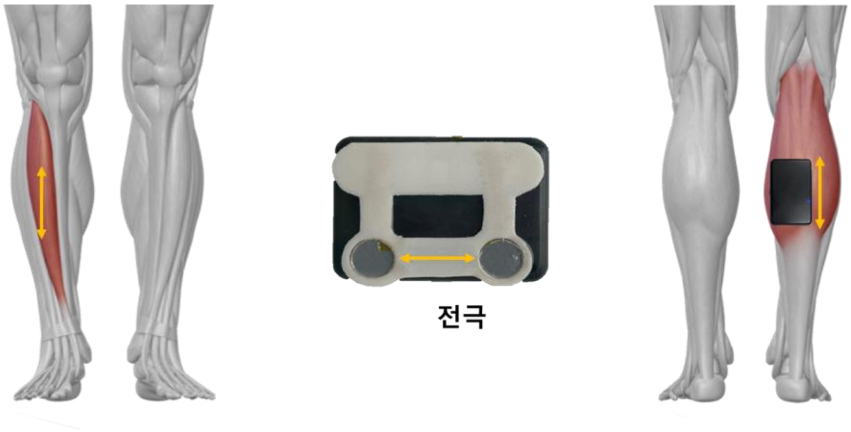

그림 28 부착 방법

패치의 두 전극을 가리키는 노란색 화살표 방향과 부착하고자 하는 근육의 노란색 화살표 방향이 평행하도록 부착함.

B. 제품 조작법

i. 결합

스냅버튼 형태의 패치(②)를 본체(①)와 결합한다.

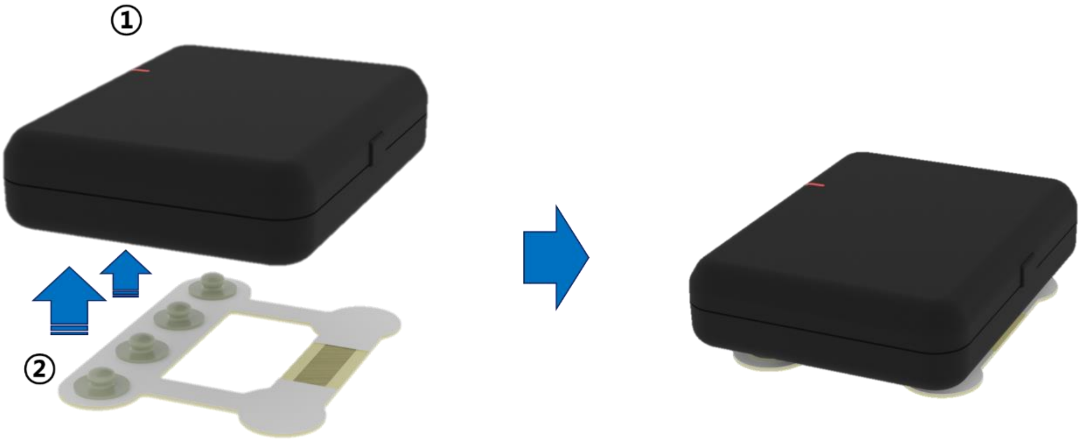

그림 29 결합 방법

ii. 전원

본체의 전원 스위치를 1초 이상 눌러 전원을 켜면 광고모드가 되어 파란색 LED가 점멸된다.

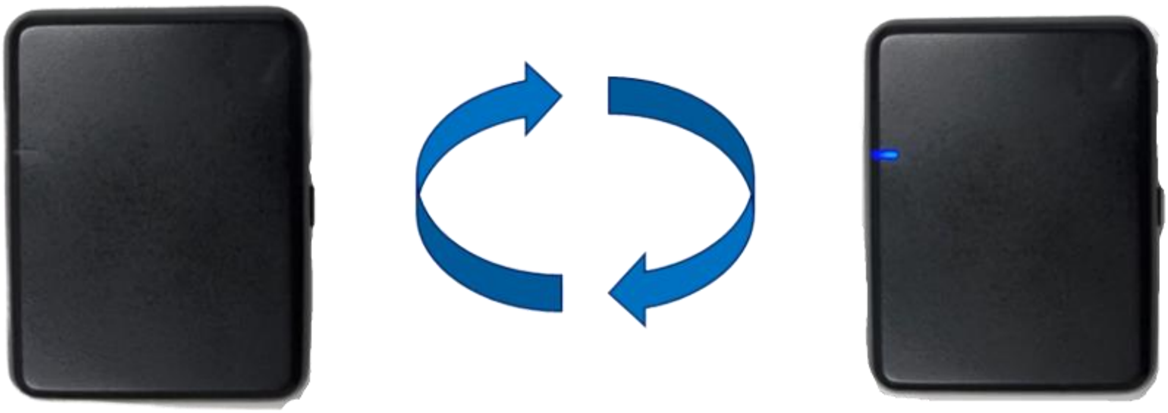

그림 30 전원 조작방법

iii. 충전

- 본체 USB Connector에 USB Cable을 연결하여 충전한다. (DC 3.7V / 450mAh)
- 충전이 진행되면 보라색 불이 들어온다.
- 충전이 완료되면 초록색 불이 들어온다.
- USB Cable을 분리한다.

충전 중에는 신호 측정이 불가능하다.

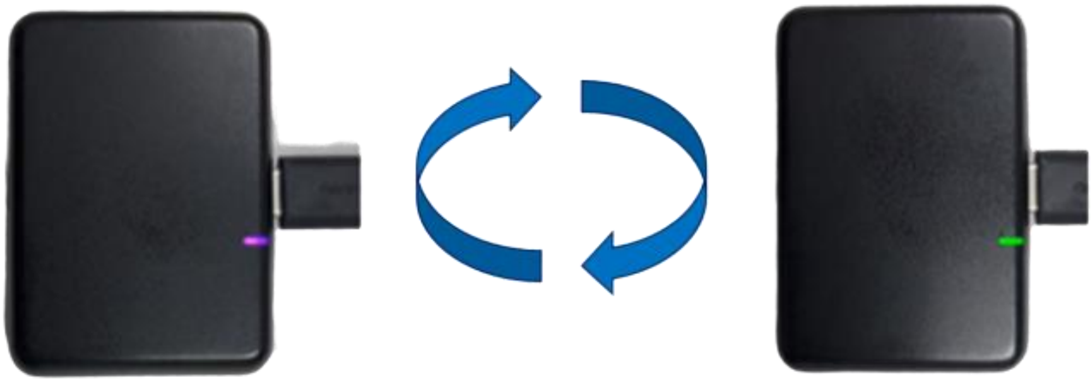

그림 31 충전 방법

④ 근전도 기기의 사용 후 보관 및 관리방법

A. 본체에 묻는 오염물질을 잘 닦은 후 보관.

B. USB cable을 이용하여 충전.

⑤ 병용요법(adjunctive therapy)

A. 자가 운동은 표준 하지재활에 병용요법으로서 적용됨

4) 관찰항목, 임상검사항목 및 관찰검사방법

(1) 임상 평가(clinical evaluation)

① 일차 평가 변수(primary outcome): 3회 시행(기준 시점-3주-12주)

A. 리버미드 운동 평가(Rivermead motor assessment, RMA): Gross function, leg and trunk)

② 이차 평가 변수(secondary outcome): 3회 시행(기준 시점-3주-12주)

A. 도수근력검사(Manual muscle test, MMT): Hip flexor, Hip abductor, Knee extensor, Ankle dorsiflexor

B. 브룬스트롬 기능회복 단계(brunnstrom stages of motor recovery, BRS)

C. 푸글마이어 척도-하지(Fugl Meyer assessment of lower extremity, FMS-LE)

D. 버그균형척도(Berg balance scale, BBS)

E. 기능적 보행 분류(Functional ambulation category, FAC)

F. 수정 랭킨 척도(modified Rankin scale, mRS)

G. 수정 바델 지수(modified Barthel index, MBI)

H. Short-form Health Survery 36 version 2(SF-36v2)

(2) 운동프로토콜 적용을 위한 평가

① 평가 항목: 1주, 2주 시행

A. 피츠버그 재활 참여 척도(Pittsburgh Rehabilitation Participation Scale, PRPS)

B. 도수근력검사(Manual muscle test, MMT): Hip flexor, Hip abductor, Knee extensor, Ankle dorsiflexor

C. 브룬스트롬 기능회복 단계(brunnstrom stages of motor recovery, BRS)

(3) 대상자 정보: 총 1회 시행(기준 시점)

① 임상 정보

A. 인구학적 정보: 나이, 성별, 우세손, 타질환 동반 유무(고혈압, 당뇨, 고지혈증, 심방세동, Charlson 동반질환지수(Charlson comorbidity index)), 교육수준(초등학교/중학교/고등학교 졸업 여부), 돌봄제공자(caregiver, 배우자(spouse)/자녀(children)/간병인/기타)

B. 뇌졸중 관련 정보: 초기 NIH 뇌졸중 척도(NIHSS), 뇌졸중 치료(thrombolysis/thrombectomy), 뇌졸중 발생으로부터 기간, 뇌졸중 종류(경색/출혈), 뇌졸중 위치(우측/좌측, 천막상부/천막하부, 운동영역피질(M1)침범/내포(internal capsule)침범), 뇌졸중 원인(TOAST classification: large-artery Atheros-clerosis/ small-vessel occlusion/ Cardioembolism/ other determined etiology/ Un-determined etiology)

② 약제: 항경직약, 항우울제, 항경련제 등

③ 인지기능: 간이정신상태검사(Mini-Mental State Examination, MMSE)

④ 우울 및 불안: 병원 불안-우울 척도(Hostpial Anxiety and Depression Scale, HADs)

⑤ 경직: 근육경직척도(Modified Ashworth scale, MAS: Hip extensor, Hip adductor, Knee flexor, Ankle plantar flexor)

⑥ 보조기: 무릎 보조기(knee stabilizer), 발목보조기(ankle-foot orthosis)

⑦ 운동유발전위검사(Motor Evoked Potential, MEP): 경두개 자기자극술을 건측과 환측의 두부(10-20 system의 C1-C2 부위)에 제공하며, 기록 전극은 하지의 abductor hallucis 근육에 부착하여 파형을 확인함. 휴식 운동 역치(resting motor threshold)는 최소 50 uv 이상의 진폭(amplitude)의 파형이 10회 중 5회 이상 관찰되는 시점의 자극 강도로 정의됨.

A. 간질 발작의 병력이 있거나 메탈 임플란트 등의 금기에 해당하는 경우 시행하지 않음.

(4) 평가시점 및 평가자

① 스크리닝 시점

참여기준 검사, 도수근력검사(MMT) 및 수정 랭킨 척도(mRS), 임상 정보, 뇌졸중 관련 정보, 약제, 간이정신상태검사(MMSE), 우울 및 불안(HADs), 근육경직척도(MAS), 보조기, 운동유발전위검사(MEP).

② 기준검사 시점

리버미드 운동 평가(RMA), 기능적 보행 분류(FAC), 수정 랭킨 척도 (mRS), 버그균형척도(BBS), 도수근력검사(MMT), 브룬스트롬 기능회복 단계(BRS), 근육경직척도(MAS), 푸글마이어 척도-하지(FMS-LE), 수정 바델 지수(MBI), Short-form Health Survery 36 version 2(SF-36v2), 피츠버그 재활 참여 척도(PRPS).

③ 3주후 검사 시점

리버미드 운동 평가(RMA), 기능적 보행 분류(FAC), 수정 랭킨 척도 (mRS), 버그균형척도(BBS), 도수근력검사(MMT), 브룬스트롬 기능회복 단계(BRS), 근육경직척도(MAS), 푸글마이어 척도-하지(FMS-LE), 수정 바델 지수(MBI), Short-form Health Survery 36 version 2(SF-36v2), 피츠버그 재활 참여 척도(PRPS).

④ 12주후 검사 시점

리버미드 운동 평가(RMA), 기능적 보행 분류(FAC), 수정 랭킨 척도 (mRS), 버그균형척도(BBS), 도수근력검사(MMT), 브룬스트롬 기능회복 단계(BRS), 근육경직척도(MAS), 푸글마이어 척도-하지(FMS-LE), 수정 바델 지수(MBI), Short-form Health Survery 36 version 2(SF-36v2), 피츠버그 재활 참여 척도(PRPS).

⑤ 운동프로토콜 적용을 위한 1주후, 2주후 검사 시점

피츠버그 재활 참여 척도(PRPS), 도수근력검사(MMT), 브룬스트롬 기능회복 단계(BRS)

그림 32 과 같이 스크리닝, 기준시점, 3 주후, 12 주후 시점 및 1 주후, 2 주후 평가를 수행하며, 각 평가에 대한 평가자는 그림 33 와 같음.

시점			스크리닝	등록	배정	기준시점	3주후	12주후
등록	(-)	참여기준 스크리닝 (MMT/mRS)	X					
	(-)	참여동의		X				
	(-)	무작위화			X			
	(-)	대상자 정보	X					
치료	대조군	표준 하지재활				X	X	X
	시험군	표준 하지재활 + 근전도 피드백 기반 감독하 자가운동 교육				X	X	
평가	기능(보행)	리버미드 운동 평가(RMA)				X	X	X
		기능적 보행 분류(FAC)				X	X	X
		수정 랭킨 척도 (mRS)	X			X	X	X
		버그균형척도(BBS)				X	X	X
	장애	도수근력검사(MMT)(고관절굴곡, 고관절외전, 무릎신전, 발등굽힘)	X			↔		X
		브룬스트롬 기능회복 단계(BRS)				↔		X
		근육경직척도(MAS)				X	X	X
		푸글마이어 척도-하지(FMS-LE)				X	X	X
	기능(일상생활)	수정 바델 지수(MBI)				X	X	X
	삶의질	Short-form Health Survery 36 version 2(SF-36v2)				X	X	X
	참여도	피츠버그 재활 참여 척도(PRPS)				↔		X
안전	이상반응	낙상, 골절, 통증 등				↔		X

그림 32 임상평가 종류 및 일정

일차평가	기능(보행)	리버미드 운동 평가(RMA)	연구 치료사-B
이차평가	장애	도수근력검사(MMT)(고관절굴곡, 고관절외전, 무릎신전, 발등굽힘)	연구 치료사-B
		브룬스트롬 기능회복 단계(BRS)	연구 치료사-B
		근육경직척도(MAS)(고관절신전, 고관절내전, 무릎굴곡, 발바닥굽힘)	연구 치료사-B
		푸글마이어 척도-하지(FMS-LE)	연구 치료사-B
	기능(보행)	수정 랭킨 척도 (mRS)	연구 치료사-B
		기능적 보행 분류(FAC)	연구 치료사-B
		버그균형척도(BBS)	연구 치료사-B
	기능(일상생활)	수정 바델 지수(MBI)	작업치료사
	삶의질	Short-form Health Survery 36 version 2(SF-36v2)	연구 치료사-B
	인지	간이정신상태검사(MMSE)	작업치료사
	약제	Depression/Spasticity/Epilepsy	연구 치료사-B
	보조기	보조기 / 보행장치	연구 치료사-B
	신경생리학적 검사	운동유발전위검사(MEP)	근전도실
	참여도	피츠버그 재활 참여 척도(PRPS)	연구 치료사-B
안전	이상반응	낙상, 골절, 통증 등	연구 치료사-B

그림 33 임상평가를 위한 평가자 배정

5) 기존 치료 및 연구와의 차별점

① 표준 하지재활에 추가적으로 추가적으로 자가운동 시행

A. 운동 회복을 위해서 중요한 요인 중 하나로 재활의 양(rehabilitation dose)이 중요하다고 알려져 있음.

B. 국내에서 국민건강보험이 적용되는 재활수가로는 하루 2회의 재활치료만이 가능하기 때문에 재활의 양을 늘리기 위한 방안 중 하나로 자가운동이 거론되고 있음.

② 근전도를 통해 획득된 데이터 및 이를 활용한 피드백에 기반한 감독하 자가운동 유형과 방식 및 단계조절(progression)

A. 환자가 혼자서 또는 보호자의 도움이 있더라도 적절한 운동 유형과 방식, 자세 등을 선정하여 효과적인 자가운동을 시행하는 것은 한계가 명확함.

B. 근전도센서 이용하여 피드백에 기반한 감독하 자가운동은 위와 같은 한계를 극복할 수 있을 것으로 기대됨.

6) 연구대상자의 이익과 위험

① 연구대상자의 예상되는 이익

기존의 연구에서 자가운동이 운동기능 회복 및 삶의 질 개선에 효과적임이 보고되어 있음. 이 뿐만 아니라 근전도를 피드백에 기반한 감독하 자가운동의 유형, 방식 및 단계조절을 시행하면 운동기능의 회복이 촉진되어 기능적 이동성을 더욱 향상시킬 수 있을 것으로 기대됨.

② 연구대상자의 예상되는 위험

본 연구의 임상시험에 참여한 연구 대상자의 예상되는 위험은 다음과 같은 항목에 해당함.

- 낙상(fall), 골절(fracture), 심각한 통증(severe pain), 운동 중 발생한 혈역학적 불안정성인 arrhythmia, bradycardia, Systolic blood pressure > 200mmHg, Systolic blood pressure< 90mmHg, Desaturation≥ 88% 등, 기타 경미한 이상반응 호흡곤란(dyspnea), 현기증(dizziness), 빈호흡(tachypnea) 또는 부비동 빈맥(sinus tachycardia) 등.

③ 피해보상에 대한 규약

A. 본 연구는 임상시험 배상보험에 가입하였으며 배상보험을 통해 피해 보상 예정임.

B. 피해 보상 기준

i. 임상시험의 참여로 인한 손상에 의한 보상 기준은 임상시험계획서에 따라 행해진 자가운동에 의한 손상임.

ii. 환자 또는 보호자는 자가운동 도중 발생할 수 있는 위험을 방지하고자 노력하였음에도 불구하고 발생한 손상이어야 함

iii. 임상시험의 참여로 인하여 대상자의 신체적인 손상(사망포함)이 발생한 경우 대상자에게 보상함.

iv. 임상시험의 참여로 인한 대상자의 신체적 손상이 발생할 경우, 금전적 보상이 확정되기 이전에 책임연구자를 통한 적절한 치료를 우선적으로 제공함.

v. 이상반응이나 이상반응 처치 과정에서 발생된 손상이 있는 경우도 보상의 대상으로 고려함.

C. 피해 보상 제외 기준

i. 임상시험의 참여로 인한 신체적인 손상(사망포함)이 아닌 경우는 보상의 대상에서 제외함.

ii. 임상시험 중이 아니어도 일어났을 것이라고 생각되는 사고 등에 기인한 것은 보상의 대상에서 제외함.

iii. 대조군인 표준 하지재활만을 시행한 대상자에게 치료상의 이익을 제공할 수 없는 경우에도 보상의 대상에서 제외함.

iv. 질병의 악화 또는 진행 결과 중 발생하는 통상적 합병증에 의한 손상은 제외함

v. 대상자 또는 그 보호자의 시험자의 지시사항 및 임상시험계획서 미준수, 고의 또는 중대한 과실로 손상이 발생한 경우 보상액을 줄이거나 보상의 대상에서 제외함.

④ 의료기기 사용 시 주의사항

A. 경고

i. 기기 변경을 포함하여 직접 분해하거나 수리하지 아니함.

ii. 기기에 충격을 주거나 떨어뜨리지 아니함.

iii. 고통이나 손상이 느껴지면 중단함.

iv. 물에 접촉하지 말고 사용 전에는 반드시 물기를 제거해야 함.

B. 의료기기 특성을 고려한 사용대상 연령, 성별 또는 건강상태 등에 대한 주의사항

i. 본 기기는 어린이 손에 닿지 않도록 주의함.

ii. 최근 근육 개선을 위한 외과 수술을 받은 사람이 사용하지 아니함.

iii. 간질이 의심되는 사람에게 사용할 때 특별이 주의하여 사용해야 함.

C. 의료기기 사용결과 발생 할 수 있는 이상반응 사용상 주의사항

i. 전극을 부착한 피부에 염증이 생길 경우 즉시 중지함.

D. 일반적 주의

i. 사용 전에는 반드시 사용자 매뉴얼을 숙지해야 함.

ii. 사용목적 이외의 사용을 금함.

iii. 배터리 충전 중에는 기기가 작동하지 아니함.

iv. 운동이나 샤워 직후에는 측정을 피함.

v. 기타 질환이나 의료적인 문제가 발생하거나 의심될 경우에는 즉시 의료전문가나 담당의사와 상의함.

vi. 사이버 보안 위협 탐지 시 사용설명서에 기재된 제조원 연락처로 연락함.

E. 상호작용

i. Cardiac pacemaker 또는 Defibrillators와 같은 장치를 사용하고 있는 환자는 사용해서는 안됨.

ii. 경동맥(목주위)에 전극을 부착해서는 안됨.

iii. 악성 종양 위에 전극을 부착해서는 안됨.

F. 적용상의 주의

i. 본체와 패치를 연결할 때 꼭 본체의 후크가 패치 후크 홀더에 먼저 연결한 후 자석으로 연결함.

G. 안전사고 예방에 필요한 주의사항

i. 장비 사용이 완료되면 앱에서 측정 종료 버튼을 누르면 본체 전원이 꺼짐.

7) 중지·탈락 기준 및 처리

① 중지: 참여대상자의 이익에 반하는 경우

A. 임상시험 도중, 중대한 이상반응 또는 임상검사의 이상 변동으로 시험 지속이 곤란하다고 판단되는 경우

B. 기타 임상시험중지가 피험자에게 이익이 된다고 시험자가 판단한 경우

② 중지처리

A. 임상시험이 중지된 경우 IRB에 서면으로 통보함.

B. 중지 전까지 진행된 임상시험 관련 자료를 기록 및 보관하며, 중지에 대한 원인이 사라진 경우 연구책임자 판단 하에 다시 개시 가능

③ 탈락: 참여대상자가 시험기준에 부합하지 않거나 더 이상 참여를 원하지 않는 경우

A. 선정/제외 기준 위반

B. 피험자나 법적 대리권자의 요청으로 인한 동의 철회

C. 기타 임상시험의 참여가 연구대상자의 건강과 복지에 해를 끼친다고 연구책임자가 판단하는 경우

④ 탈락처리

A. 연구대상자가 중도 탈락한 경우 임싱시험책임자는 연구대상자에게 이 사실을 즉시 알리고 적절한 조치와 추적 관찰이 이루어질 수 있도록 함.

B. 연구대상자가 중도 탈락한 경우 임상시험책임자는 탈락 사유 및 해당 시점까지 진행된 임상시험관련 연구대상자의 기록, 동의서, 증례기록서 사본 등 임상자료를 생명윤리법 시행규칙 제15조에 따라 연구가 종료된 시점부터 3년간 보관하며, 보관기간이 지난 문서는 개인정보보호법 시행령 제16조에 따라 파기함.

C. 중도 탈락된 임상시험 참여자의 정보는 타당한 이유가 없다면 유효성 및 안전성 평가 통계 처리에 포함함.

D. 임상시험에서 얻어진 자료에서 결측치가 생겼을 때 타당한 이유가 없다면 해당 참여자의 정보를 분석에서 제외하지 않으며, 선형혼합모형(linear mixed model)을 이용하여 결측치를 처리함.

8) 부작용을 포함한 안전성의 평가기준, 평가 방법 및 보고 방법

본 임상시험 중 발생한 모든 이상반응이 안전성 평가에 포함되며, 의료진에 의해 측정한 활력 징후, 이학적 검사 및 피험자에 의해 보고되는 이상반응으로 안전성을 평가한다.

① 이상반응의 정의

i. "이상반응(Adverse Event, AE)"이란 임상시험 중 피험자에게 발생한 모든 유해하고 의도되지 않은 증후(sign), 증상(symptom) 또는 질병을 말하며, 해당 시험 중재와 반드시 인과관계를 가져야 하는 것은 아님.

ii. "이상의료기기반응(Adverse Device Effect, ADE)"이라 함은 임상시험용 의료기기로 인하여 발생한 모든 유해하고 의도하지 않은 반응으로써 임상시험용 의료기기와의 인과관계를 부정 할 수 없는 경우

iii. "중대한 이상반응(Serious AE)"이란 해당 시험 중재로 인하여 발생한 이상반응 또는 이상반응 중에서 다음의 어느 하나에 해당하는 경우를 말한다.

1. 사망하거나 생명에 대한 위험이 발생한 경우
2. 입원할 필요가 있거나 입원 기간을 연장할 필요가 있는 경우
3. 영구적이거나 중대한 장애 및 기능 저하를 가져온 경우
4. 태아에게 기형 또는 이상이 발생한 경우

② 이상반응의 평가기준

i. 중증도 평가

1. Grade 1: 경증(mild); 피험자의 정상적인 일상생활(기능)을 저해하지 않고, 최소한의 불편을 야기하며 피험자가 쉽게 견딜 수 있는 경우
2. Grade 2: 중증도(moderate); 피험자의 정상적인 일상생활(기능)을 유일하게 저하해는 불편을 야기하는 경우
3. Grade 3: 중증(severe); 피험자의 정상적인 일상생활(기능)을 불가능하게 하는 경우

ii. 인과관계 평가

1. 관련성이 명백함(Definitely related)
2. 관련성이 많은(Probably related)
3. 관련성이 의심됨(Possibly related)
4. 관련성이 적음(Probably not related)
5. 관련성이 명백히 없음(Definitely not related)

6. 평가 불가능(Unkown)

iii. 안전성의 평가기준

1. 본 임상시험에서 이상반응은 임상시험 시작 전에 관찰되지 않은 증상이 발생하는 모든 바람직하지 않은 의학적 소견을 이상반응으로 분류
2. 예측되는 부작용도 이상반응으로 분류하며 이상반응의 정도를 경증, 중증도, 중증으로 분류
3. 예측되는 부작용 항목별 판정기준, 판정방법, 대응방법

예측되는 부작용 항목	판정기준	판정방법	대응
낙상(fall)	침상에서 떨어지거나 이동훈련 중 넘어져서 다친 상태	치료사 및 의료진 확인	즉시 담당 의료진 및 재활처방 의사에게 보고 및 필요시 X-ray, CT 등 추가 검사 시행
골절(fracture)	영상 검사에서 뼈의 골절 발생 확인	임상 증상 및 X-ray	즉시 담당 의료진 및 재활처방 의사에게 보고 및 필요시 X-ray, CT 등 추가 검사 시행
심각한 통증 (severe pain)	환자가 호소하거나 특정 부위의 멍 또는 부종등과 함께 통증이 강력히 의심되는 상태	재활 중 또는 직후 발생하고 3일 이상 지속되는 통증	통증이 있는 부위의 확인 및 의료진과 재활처방 의사에게 보고, 필요시 X-ray 등 추가 검사 시행
운동 중 발생한 혈역학적 불안정성	부정맥(arrhythmia), 서맥(bradycardia), 수축기혈압(Systolic blood pressure) > 200mmHg, 수축기혈압(Systolic blood pressure) < 90mmHg, 산소포화도(SpO2) ≤ 88% 등	치료사 및 의료진 확인	즉시 의료진에게 보고 및 필요시 추가 검사 시행, 재활처방 의사에게 보고
기타 경미한 이상반응	현기증(dizziness), 빈호흡(tachypnea) 또는 부비동 빈맥(sinus tachycardia), 초조	치료사 및 의료진 확인	즉시 의료진에게 보고 및 필요시 추가 검사 시행, 재활처방 의사에게 보고

	(agitation) 등		
이외의 이상반응	-	치료사 및 의료진 확인	즉시 의료진에게 보고 및 필요시 추가 검사 시행, 재활처방 의사에게 보고

표 10 예측되는 부작용, 판정기준, 판정방법, 대응방법

③ 안전성 평가방법

i. 발현된 모든 예측되는 부작용 및 이상반응은 자세한 설명과 함께 나열함

ii. 임상시험용 의료기기와 연관성이 있는 이상반응과 연관성이 없는 이상반응의 빈도를 기록

④ 이상반응 보고방법 및 이상반응 발생시 조치사항

i. 이상반응 교육

: 연구책임자는 연구담당자 및 피험자 또는 대리인에게 시험 중재 후 나타날 수 있는 모든 이상반응에 대하여 교육을 실시하고 사용 후 나타나는 모든 현상에 대하여 보고 하도록 교육을 실시

ii. 심각한 이상반응/이상의료기기반응 보고방법

: 연구책임자는 연구기간 중 발생한 모든 중대한 이상반응을 시험 중재와 관련성 여부와 상관없이 24시간 이내에 IRB에 보고한다.

: 이외에도 연구자가 중대하다고 간주하거나, 시험 중재와의 연관 지을 수 있는 유의한 위험, 금기, 부작용, 주의사항을 시사하는 사건 등도 심각한 이상반응으로 기록하고 연구책임자가 사실을 보고 받거나 알게 된 날로부터 7일 이내 보고한다. 그 밖의 중대하거나 예상하지 못한 모든 이상반응이 나타난 경우에는 의뢰자가 이 사실을 보고 받거나 알게 된 날로부터 15일 이내 보고한다.

iii. 이상반응 발생시 조치사항

: 본 임상시험 기간 중 연구책임자, 연구담당자는 환자의 안전에 만전을 기하여야 하며, 예측되지 않은 중대한 이상반응 발생시에는 신속하고 적절한 조치를 취하여 이상반응을 최소화 하여야 한다.

iv. 이상반응의 추적관찰

: 연구자는 이상반응이 나타난 피험자에 대해 증상이 소실되고 상태가 안정될 때까지 피험자를 추적 관찰해야 하며, 필요 시 IRB에 이상반응의 이후 진행경과에 대한 보고서를 제출한다.

9) 자료 분석 및 통계 계획

① 일차 평가변수: 대조군과 시험군 사이의 치료 후 기준시점-3주-12주의 리버미드 운동

평가(gross function, leg and trunk) 점수는 repeated measures ANOVA 또는 linear-mixed model을 이용하여 분석함.

② 이차 평가변수: 대조군과 시험군 사이의 치료 후 기준시점-3주-12주의 이차 평가변수에 대하여 repeated measures ANOVA 또는 linear-mixed model을 이용하여 분석함.

10) 자료 관리 계획

자료 기록 및 수집, 접근, 보호, 보관에 대하여 아래와 같이 관리할 계획임

A. 자료의 수정시에 자료의 수정과정을 기록하고 기존에 입력한 자료를 삭제하지 않도록 하며, 문서화 또는 전자화된 증례기록서의 내용을 변경하거나 정정시에 작성한 수정 지침에 따라 원래의 내용을 알아볼 수 있도록 수정하고, 수정일 및 수정 이유를 적고 서명하도록 함.

B. 임상시험 자료관리를 위해 인가되지 않은 자에 의한 자료의 접근을 막을 수 있도록 개인별 ID, password를 통해 이루어지도록 함.

C. 자료 수정이 인가된 자의 명단을 갖추도록 함.

D. 자료의 복사본을 갖추어 둠.

E. 해당 임상시험과 관련하여 눈가림이 필요한 경우 자료입력 및 처리과정에서 눈가림 상태를 유지함.

11) 임상시험 후 연구대상자의 진료 및 치료기준

임상시험에 참여한 연구참여자는 일반적인 뇌졸중 재활의 프로세스에 따라서 진료 및 치료가 이루어질 예정이며, 1달 간의 자가운동 적용 이외에 체계적인 진료 및 치료 프로세스에서 일반적인 뇌졸중 재활과 차이가 나는 부분은 없을 것임

12) 연구수행일정표

Months	1-4	5-8	9-12	13-16	17-20	21-24	25-28	29-32	33-36
FDA Medical Device Clinical Trial Plan Approval	●								
Medical Device Clinical Trial IRB Approval	●								
Medical Device Clinical Trial	●	●	●	●	●	●	●		
Statistical Analysis and Results Verification for Clinical Trial Data								●	
Writing and publishing									●

표 11 연구수행일정표

11. 자료 및 안전성 모니터링 계획 (Data and Safety Monitoring Plan)

1) 모니터링 책임자

***모니터링 책임:** 서울대학교병원 재활의학과 임상조교수 이우형

***모니터링 담당:** 서울대학교병원 재활의학과 연구교수 김정현

2) 자료 및 안전성 정보 모니터링 항목

***자료(study accruals) 항목:** CRF상의 임상 정보

***안전성(Safety) 항목:**

① 낙상(fall)

② 골절(fracture)

③ 심각한 통증(severe pain)

④ 운동 중 발생한 혈역학적 불안정성

i. Arrhythmia

ii. Bradycardia

iii. Systolic blood pressure > 200mmHg

iv. Systolic blood pressure < 90mmHg

v. Desaturation ≥ 88%

⑤ 기타 경미한 이상반응

i. 호흡곤란(dyspnea)

ii. 현기증(dizziness)

iii. 빈호흡(tachypnea) 또는 부비동 빈맥(sinus tachycardia) 등

3) 자료 및 안전성 모니터링 방법 및 주기

① 자료 및 안전성 모니터링 방법

연구참여자가 설명을 듣고 동의서를 받을 때 연구원이 안전성 모니터링의 보고체계를 설명하고 연구 수행 중 연구와 연관된 이상반응 발생 시 즉시 전체 연구진에게 이에 대해서 전달 및 조치를 시행.

② 자료 및 안전성 모니터링 주기

5명 연구대상자 등록마다 자료 및 안정성 모니터링 시행

4) 약물/의료기기 이상반응 보고, 연구 미준수, 예상하지 못한 문제의 보고

중대한 이상반응, 중대한 연구 미준수, 예상하지 못한 문제가 발생한 경우 연구자 인지 일로부터 근무일 15일 이내에 모니터링 책임자를 거쳐 IRB에 보고한다.

5) 연구 중단 기준

- 본 연구는 임상 현장에서 뇌졸중 환자 대상으로 흔히 수행되는 평가 및 치료 과정에서 근전도 피드백 기반의 자가운동만이 추가되는 것으로 이로 인한 추가적인 위험도는 매우 낮을 것으로 예상됨. 추가적으로 시행함으로써 시행하지 않았으면 발생하지 않았을 중증 합병증이 발생할 가능성이 매우 낮을 것으로 예측됨. 만일 연구 개시 이전과 비교하여 합병증의 발생률이 현저히 높다고 판단될 시 본 연구를 중지하기로 함.

- 목표한 연구 자료의 획득의 25% 이상이 9개월 이내에 획득되지 않거나, 50% 이상이 1년 6개월 내에 획득되지 않을 때 연구관계자 회의를 통해 재평가하고, 연구재개여부를 결정함.

12. 연구대상자의 안전보호를 위한 대책

1) 연구의 윤리성 확보를 위한 기본 방안

- 본 연구를 수행하는 연구 책임자 및 담당자들은 헬싱키 선언(2013년 개정) 및 ICH-GCP를 준수할 것이며, IRB 승인 후 연구를 수행할 것임. 본 연구는 헬싱키 선언에 입각하여 환자의 권리와 복지를 기초에 둔 것으로 연구에 들어가기 전에 연구대상자 및 법정대리인에게 연구 내용 및 예측되는 효과, 이상반응 및 안전성에 관한 모든 사항을 환자 및 법정대리인이 이해할 수 있는 언어로 충분히 설명하여 자발적인 동의를 받은 후 연구에 들어감.

- 연구의 목적 및 방법은 연구대상자 및 법정대리인에게 설명문을 통해 설명하며, 본 연구의 목적을 알고 동의서를 작성한 연구대상자만 시험에 참여할 수 있음. 연구대상자의 신원을 파악할 수 있는 기록은 비밀로 보장될 것이며, 연구의 결과가 출판될 경우 피험자의 신원은 비밀 상태로 유지될 것임.

2) 연구대상자의 동의 과정

- 본 연구를 위하여 서울대학교병원에서 연구책임자(교수 이우형) 또는 연구담당자가 임상시험에 들어가기 전에 피험자 및 법정대리인에게 연구 내용 및 예측되는 효과, 이상반응 및 안전성에 관한 모든 사항을 환자 및 법정대리인이 이해할 수 있는 언어로 10분 이상(필요한 경우 수일 이상) 충분히 설명하여 자발적인 동의를 받은 후 연구 개시에 들어감.

- 강제 또는 부당한 영향의 가능성을 최소화시킬 방법: 임상연구 참여 여부는 자율적으로 결정하도록 하고, 필요한 경우 참여 여부에 대해 고려할 시간을 충분히 수일 이상 제공하며, 연구 대상 후보자가 본 과에서 진료를 받고 있는 경우 참여 여부를 진료 내용과 연계하지 않음.

3) 연구대상자의 보상 방안

- 병원 내에서 행해지는 뇌졸중 재활 치료에 대한 사례비 제공은 없음.

- 임상시험 참여 후 12주차 측정시점까지 모두 참여하시는 분께는 3주차 측정이 끝난 후 2주 이내에 평가비용을 포함하여 40만원, 12주차측정이 끝난 후 2주 이내에 평가비용 20만원 총 60만원을 연구진이 연구대상자에게 지급함.
- 모든 평가에서 퇴원 후 이루어지는 방문 측정은 방문횟수 별로 회당 교통비 20만원을 별도로 연구진이 연구대상자에게 지급함.
- 중지 및 탈락이 발생하여 연구를 끝까지 진행하지 못하는 경우 완료된 측정에 대하여 초기 측정 평가비용 포함 20만원, 3주차 측정 평가비용 포함 20만원을 계산하여 3주차 측정이 완료된 후 2주일 내에 연구진이 연구대상자에게 지급함.
- 중지 및 탈락이 발생하여 연구를 끝까지 진행하지 못하는 경우 완료지 않은 측정에 대하여 평가비용과 교통비만을 제공함.
- 임상시험에서 행해지는 평가에 대한 비용을 연구비에서 지급함.
- 연구대상자가 끝까지 참여하지 못했다면 이미 시행한 평가에 해당하는 금액을 지급함.
- 예기치 않게 발생하는 부작용에 대한 모든 치료의 경비뿐만 아니라 보상에 대해서 연구책임자가 책임을 짐.

4) 연구대상자의 개인정보보호 방안

① 환자의 의무기록번호 및 병리 번호는 주관연구자의 책임하에 별도의 파일로 보관하며 이를 코드화하여 연구데이터를 통하여 개인 신상 확인이 불가능하도록 관리한다. 또는 연구데이터는 패스워드가 걸린 파일에 저장하여 잠금 장치가 있는 연구실에 보관하도록 한다. 생명윤리법 시행규칙 제15조에 따라 연구 관련 기록을 연구가 종료된 시점부터 3년간 보관하며, 보관기관이 지난 문서 중 개인정보에 관한 사항은 개인정보보호법 시행령 제16조에 따라 파기할 것이나 후속 연구, 기록, 축적 등을 위하여 필요하다면 3년 이상 보관할 수 있으며, 이러한 경우 개인정보는 확인이 불가능 하도록 관리한다. 임상시험의 결과가 출판될 경우 대상자의 신상은 비밀로 보호한다.

② 치료용 범용 소프트웨어 앱 사용자 정보 보안 관련 개발내용

A. 환자 및 진료 정보의 비식별화 처리
B. 진단 및 처방 데이터의 비대칭 암호화
C. 비대칭 암호화/복호화를 위한 맵핑 키 관리
D. 암호화 키의 안전성 확보를 위한 저장소 분리
E. 이종의 저장소 간 암호화 키 추출 및 맵핑

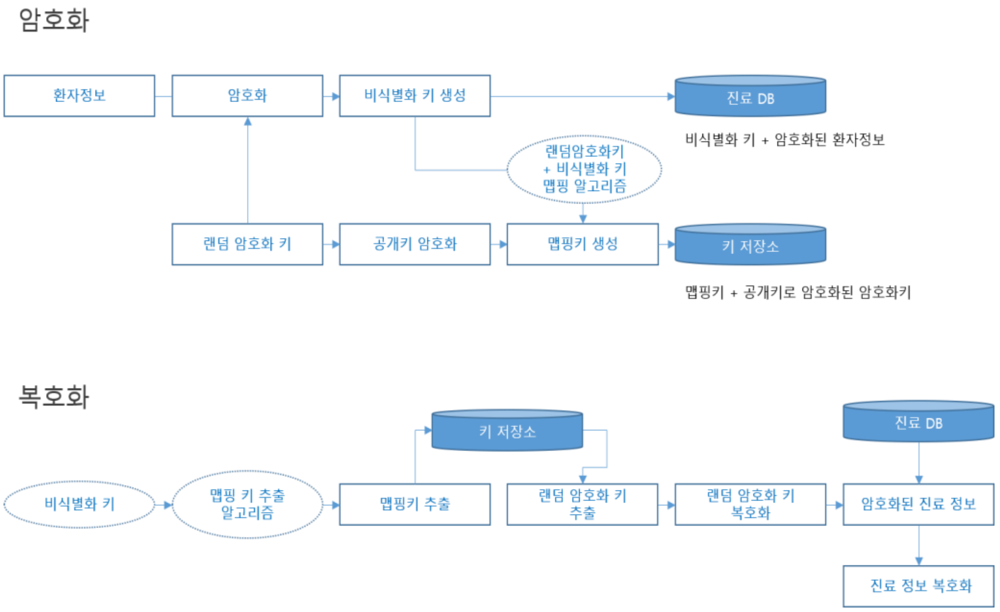

그림 34 익명화된 진료정보의 비대칭 암호화/복호화 방법 예시

5) 취약한 연구대상자를 포함하는 경우 추가적인 보호조치 방안

① 연구대상자의 동의 능력에 대한 평가를 통하여 본인의 판단 하에 연구참여에 대한 동의가 어렵다고 판단되면 연구대상자 본인의 승낙 및 대리인의 동의를 통하여 연구에 참여하도록 함.

② 동의 능력 평가 방법은 1) 연구대상자가 연구와 관련된 정보를 이해하는지 여부 2) 연구에 대한 정보를 논리적으로 다룰 수 있는지 3) 연구에 참여하고 싶어하는지 아닌지에 관한 자신의 선택을 명확하게 의사 표현하는 능력이 있는지 등을 종합적으로 판단

③ 본 연구는 중환자를 대상으로 하며 제한된 동의 능력을 가진 성인으로 연구대상자에게 동의를 받아야 하는 경우에만 법적 대리인에게 동의를 받는 것으로 하며, 아래와 같은 보호조치를 반드시 지킬 계획임.

④ 동의 능력이 손상된 성인을 위한 보호조치 (HRPP SOP ver.3.7 참고)

A. 본 연구는 뇌졸중 환자를 대상으로 하며 대부분 동의능력이 상실된 상태임.

B. 본 연구는 최소한의 위험을 넘지 않는 연구이며, 본 연구의 결과는 치료법 개발을 위한 후속 연구의 기반으로서 사용될 예정으로 후속 연구의 결과에 따라 간접적인 이익을 얻을 수 있음.

C. 연구 진행 중에 인지기능이 손상된 뇌졸중 환자 옆에 항상 대리인 또는 간병인과 같은 제 3자가 대기하도록 할 예정이며, 거동이 불편한 연구대상자의 경우 연구원이 낙상 방지를 위하여 주의를 기울이고 노력할 것임.

D. 참여하는 모든 연구대상자의 대리인에게 동의를 받을 예정임. 연구진이 대리인에게 직접 연구 및 연관되어 발생 가능한 부작용에 관하여 설명하고 대리인의 동의를 통

하여 연구에 참여하도록 함.

⑤ 피고용자를 위한 보호조치 (HRPP SOP ver.3.7 참고)

A. 연구대상자 모집의 목적으로 연구자가 피고용자와 직접적인 상호작용을 하지 않아야 한다.

B. 가능하다면 연구 모집이나 동의 과정은 피고용자의 상급자가 없는 상태에서 진행되어야 한다.

C. 피고용자가 연구에 참여하지 않기로 결정할 수 있으며, 그러한 결정이 그들의 고용이나 직무 평가에 영향을 미치지 않을 것임을 알린다.

D. 피고용자가 부당한 영향이나 강제를 받지 않을 것이며, 피고용자의 사생활이 존중될 것임을 보장할 수 있는 절차를 마련한다.

E. 연구 참여를 거절했다는 사실이 피고용자의 상급자에게 노출되지 않도록 조치를 취해야 한다.

F. 가능하다면 연구 진행은 다른 피고용자가 없는 상태에서 진행되어야 한다. 예를 들어, 조사나 설문 연구에서 모든 피고용자를 한 방에 모아 놓고 현장에서 답을 하도록 요구함으로써 상급자나 동료들이 피고용자를 연구대상자로 인식하도록 만드는 방식 대신, 피고용자가 집에서 자료 작성을 완료하여 연구자에게 메일로 회신하는 방식으로 수행될 수 있다.

G. 의뢰자의 피고용자를 연구대상자로 모집하는 경우, 의뢰자에게 제공될 모든 자료는 요약 또는 모든 개인식별 정보를 제거함으로써 피고용자의 신원이 보호되도록 해야 한다.

13. 인체유래물의 보관 및 폐기 방법

해당 사항 없음

14. 참고 문헌

1. Hemmatpour, M., et al., *A Review on Fall Prediction and Prevention System for Personal Devices: Evaluation and Experimental Results.* Advances in Human-Computer Interaction, 2019. **2019**.
2. Chung, I.Y., et al., *An Assessment of Physical Activity Data Collected via a Smartphone App and a Smart Band in Breast Cancer Survivors: Observational Study.* Journal of Medical Internet Research, 2019. **21**(9).
3. Barton, J., B. O'Flynn, and S. Tedesco, *A Review of Physical Activity Monitoring and Activity Trackers for Older Adults.* Harnessing the Power of Technology to Improve Lives, 2017. **242**: p. 748-754.
4. Woodford, H. and C. Price, *EMG biofeedback for the recovery of motor function after stroke*

(Review). Cochrane Database of Systematic Reviews, 2007(2).

5. Rasool, G., et al., *Spatial Analysis of Multichannel Surface EMG in Hemiplegic Stroke.* Ieee Transactions on Neural Systems and Rehabilitation Engineering, 2017. **25**(10): p. 1802-1811.
6. Gamez, A.B., et al., *The effect of surface electromyography biofeedback on the activity of extensor and dorsiflexor muscles in elderly adults: a randomized trial.* Scientific Reports, 2019. **9**.
7. Klassen, T.D., et al., *Higher Doses Improve Walking Recovery During Stroke Inpatient Rehabilitation.* Stroke, 2020. **51**(9): p. 2639-2648.
8. Hornby, T.G., et al., *Contributions of Stepping Intensity and Variability to Mobility in Individuals Poststroke A Randomized Clinical Trial.* Stroke, 2019. **50**(9): p. 2492-2499.
9. Lacroix, J., et al., *Physical Activity Level Among Stroke Patients Hospitalized in a Rehabilitation Unit.* Pm&R, 2016. **8**(2): p. 97-104.
10. Everard, G., et al., *Self-Rehabilitation for Post-Stroke Motor Function and Activity-A Systematic Review and Meta-Analysis.* Neurorehabilitation and Neural Repair, 2021. **35**(12): p. 1043-1058.
11. Chen, J., et al., *Effects of Home-based Telesupervising Rehabilitation on Physical Function for Stroke Survivors with Hemiplegia A Randomized Controlled Trial.* American Journal of Physical Medicine & Rehabilitation, 2017. **96**(3): p. 152-160.
12. Golla, A., et al., *Home-based balance training using Wii Fit™: a pilot randomised controlled trial with mobile older stroke survivors.* Pilot and feasibility studies, 2018. **4**(1): p. 1-10.
13. Peretti, A., et al., *Telerehabilitation: review of the state-of-the-art and areas of application.* JMIR rehabilitation and assistive technologies, 2017. **4**(2): p. e7511.
14. Hsieh, C.L., I.P. Hsueh, and H.F. Mao, *Validity and responsiveness of the rivermead mobility index in stroke patients.* Scandinavian Journal of Rehabilitation Medicine, 2000. **32**(3): p. 140-142.
15. Hsueh, I.P., et al., *Comparison of psychometric properties of three mobility measures for patients with stroke.* Stroke, 2003. **34**(7): p. 1741-1745.
16. Hernández, E.D., et al., *Intra-and interrater reliability of Fugl-Meyer Assessment of Lower Extremity early after stroke.* Brazilian Journal of Physical Therapy, 2020.
17. Kudlac, M., et al., *Reliability and validity of the Berg balance scale in the stroke population: a systematic review.* Physical & Occupational Therapy in Geriatrics, 2019. **37**(3): p. 196-221.
18. Viosca, E., et al., *Proposal and validation of a new functional ambulation classification scale for clinical use.* Archives of Physical Medicine and Rehabilitation, 2005. **86**(6): p. 1234-1238.
19. Rebchuk, A.D., et al., *Health utility weighting of the modified Rankin scale: a systematic review and meta-analysis.* JAMA network open, 2020. **3**(4): p. e203767-e203767.
20. Ohura, T., et al., *Validity and reliability of a performance evaluation tool based on the modified Barthel Index for stroke patients.* BMC medical research methodology, 2017. **17**(1): p. 1-8.

21. Lenze, E.J., et al., *The Pittsburgh Rehabilitation Participation Scale: reliability and validity of a clinician-rated measure of participation in acute rehabilitation.* Archives of physical medicine and rehabilitation, 2004. **85**(3): p. 380-384.
22. Anderson, C., S. Laubscher, and R. Burns, *Validation of the Short Form 36 (SF-36) health survey questionnaire among stroke patients.* Stroke, 1996. **27**(10): p. 1812-1816.
23. 국가법령전보센터, 의료기기법 시행규칙, 2022.01.
24. 식품의약품안전처, 의료기기 품목 및 품목별 등급에 관한 규정, 2022.03
25. 식품의약품안전처, 의료기기 임상시험 안내서, 2020.11.
26. 식품의약품안전처, 의료기기 임상시험 길라잡이, 2020.03.
27. 식품의약품안전처, 의료기기 임상시험계획 승인에 관한 규정, 2016.11.

[별첨 1] 자료 및 안전성 모니터링 계획서 (Data and Safety Monitoring Plan)

1. 모니터링 책임자

1) 전체 모니터링 책임:

서울대학교병원 재활의학과 임상조교수 이우영

2) 본 기관 모니터링 담당:

서울대학교병원 재활의학과 연구교수 김정현

2. 자료 및 안전성 정보 모니터링 항목

1) 자료(study accruals) 항목

CRF상의 임상 정보

2) 안전성(Safety) 항목

낙상, 골절, 심각한 통증, 운동 중 발생한 혈역학적 불안정성, 기타 경미한 이상반응

3. 자료 및 안전성 모니터링 방법 및 주기

1) 자료 및 안전성 모니터링 방법

연구참여자가 설명을 듣고 동의서를 받을 때 연구원이 안전성 모니터링의 보고체계를 설명하고 연구 수행 중 연구와 연관된 이상반응 발생 시 즉시 전체 연구진에게 이에 대해서 전달 및 조치를 시행.

2) 자료 및 안전성 모니터링 주기

5명 연구대상자 등록마다 자료 및 안정성 모니터링 시행

4. 이상약물반응보고, 연구 미준수, 예상하지 못한 문제의 보고

중대한 이상반응, 중대한 연구 미준수, 예상하지 못한 문제가 발생한 경우 연구자 인지 일로부터 근무일 15일 이내에 모니터링 책임자를 거쳐 IRB에 보고한다.

5. 연구 중단 기준

- 본 연구는 임상 현장에서 뇌졸중 환자 대상으로 흔히 수행되는 평가 및 치료 과정에서 근전도 피드백 기반의 자가운동만이 추가되는 것으로 이로 인한 추가적인 위험도는 매우 낮을 것으로 예상됨. 추가적으로 시행함으로써 시행하지 않았으면 발생하지 않았을 중증 합병증이 발생할 가능성이 매우 낮을 것으로 예측됨. 만일 연구 개시 이전과 비교하여 합병증의 발생률이 현저히 높다고 판단될 시 본 연구를 중지하기로 함.

- 목표한 연구 자료의 획득의 25% 이상이 9개월 이내에 획득되지 않거나, 50% 이상이 1년 6개월 내에 획득되지 않을 때 연구관계자 회의를 통해 재평가하고, 연구재개여부를 결정함.

[별첨 2] 연구대상자 설명문 및 동의서

대상자 일련번호	

연구대상자 설명문 (의료기기 임상시험)

1. 임상시험 제목

아급성기 뇌졸중 환자에서 근전도 센서 피드백에 기반한 침상내 자가운동의 기능적 이동성에 대한 효과: 전향적, 다기관, 공개, 평가자 눈가림, 탐색적 예비임상시험

2. 연구책임자

서울대학교병원 재활의학과 임상조교수 이우형

3. 임상시험의 배경 및 목적

참여하실 시험은 치료 또는 진료가 아닌 연구의 목적으로 수행되며 검증되지 않은 임상시험이라는 사실을 미리 알려드립니다.

뇌졸중 환자의 일상생활 및 삶의 질, 활동을 수행함에 있어 기능적 독립성이 감소하게 됩니다.

근전도 센서는 뇌졸중환자의 하지의 근육의 활성도를 측정할 수 있습니다. 적절한 근육의 활성도가 성공적인 동작을 수행하는 데에 매우 중요하며, 뇌손상으로 인한 저하된 근육의 활성도를 증가시키는 것이 치료의 효과를 개선시키는 데에 중요합니다.

뇌졸중 환자의 이동 기능 회복에 있어 재활치료를 자주 받는 것이 중요하지만 실제로는 다양한 이유로 인해 충분한 강도 및 양으로 제공이 되고 있지 못하고 있습니다. 최근 발표된 연구에서 자가운동 프로그램은 일반적인 재활과 유사한 정도의 치료효과가 있다고 보고되었습니다.

이에 병원에서 제공하는 재활치료와 더불어 근전도 센서의 신호를 눈으로 보며 스스로 수행하는 하지 자가운동을 추가하여 임상시험을 수행함으로써, 기능적 이동성을 개선시키는 데에 대한 효과 및 타당성을 확인하고자 합니다.

4. 임상시험용 의료기기

패치 형태의 기기는 피부에 완전히 밀착되어 표면 뒤틀림에도 양질의 전기적인 신호를 측정하고, Mobile Application 과 Cloud Server 에서 측정된 신호를 분석하여 균형 및 종합적인 근기능을 평가함으로써 의료진과 연동한 신경계질환 환자 모니터링 및 맞춤형 재활 치료를 보조하기 위한 의료기기.

5. 임상시험 참여대상자 수 및 참여기간

임상시험에 참여하는 대략의 대상자 수: 뇌졸중 환자로 진단받은 지 3 개월 이내의 성인 24 명

대상자의 임상시험 예상 참여 기간은 총 12 주로 측정 회수는 초기, 3 주, 12 주마다 1 회씩 3 회 측정하며 운동프로그램 제공을 위해 1 주, 2 주에 근력과 회복단계를 측정 할 예정입니다.

서울대학교병원에서는 5 명의 대상자 모집을 예상하고 있으나 국립교통재활병원과 경쟁적 모집을 할 계획으로 서울대학교병원의 모집대상자는 더 적거나 많을 수 있습니다.

6. 임상시험 방법

임상시험 참여 대상자는 동의서를 작성하고 무작위 층화 배분과정을 거처 시험군과 대조군에 같은 확률로 배정되며 시험군 대조군 모두 12 주동안 3 차례 측정에 참여하게 됩니다. 초기, 3 주, 12 주 측정에는 리버미드 운동 평가(하지기능검사), 기능적 보행 분류(보행능력검사), 수정 랭킨 척도(신체기능장애 평가), 버그균형척도(균형능력 검사), 도수근력검사(근력검사), 브룬스트롬 기능회복 단계(뇌졸중 회복검사), 근육경직척도(근육경직 검사), 푸글마이어 척도-하지(하지의 기능회복검사), 수정 바델 지수(일상생활동작검사), Short-form Health Survery 36 version 2(삶의 질 측정)항목을 측정, 피츠버그 재활 참여 척도(운동참여도검사) 항목을 측정하고, 운동프로토콜 적용을 위해 1 주와 2 주에는 도수근력검사, 브룬스트롬 기능회복 단계, 피츠버그 재활 참여 척도 항목을 측정하게 됩니다.

1) 자가운동 및 하지재활치료

자가운동은 한 세션 당 30 분 이상, 하루 총 2 세션 이상 수행, 주 5 일간 3 주 동안 시행하며 하지재활치료는 세션 당 30 분, 하루 총 2 세션, 주 5 일간 3 주 동안 제공됩니다. 대조군은 관절범위운동과 함께 기능적 이동성에 입각하여 수행되는 표준 하지재활치료 이외 다른 운동은 제공되지 않습니다. 시험군은 표준 하지재활치료와 더불어 아래와 같이 근전도를 통해 획득된 데이터 및 이를 활용한 피드백에 기반한 감독하 자가운동을 수행하게 됩니다.

① 의료진이 환자별 재활치료에 필요한 동작을 의료진 앱에 입력한다.

② 환자는 재활치료를 위하여 근전도 센서를 부착하고 환자 이름을 입력한 후 처방 받은 재활치료 자가운동을 진행한다.

③ 진행되는 동안 실시간 근전도 신호를 볼 수 있고 완료 후 운동 수행 결과(운동시간, 운동횟수, 정확도) 등을 확인한다.

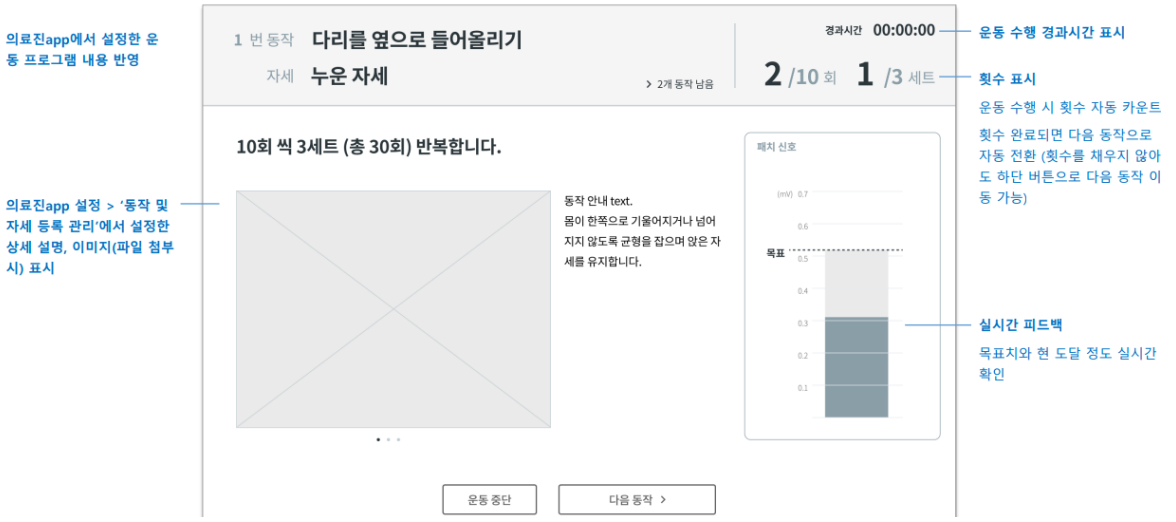

<어플리케이션 화면>

2) 측정 (소요시간: 약 2시간)

① 동의서 작성

실험에 참여한 지원자는 연구의 목적과 데이터의 활용에 대한 설명을 듣고 사전 검사 및 연구 동의서를 작성

② 임상 평가(clinical evaluation) 실시

리버미드 운동 평가 – 이동 능력 평가
도수근력검사 – 근력평가
브룬스트롬 기능회복 단계 – 뇌졸중 기능회복 평가
푸글마이어 척도-하지 - 뇌졸중 기능적 회복 정도 양적 평가
버그균형척도 – 균형능력 평가
기능적 보행 분류 – 보행능력 평가
수정 랭킨 척도 – 장애 정도 혹은 의존도의 정도를 평가
수정 바델 지수 – 일상생활 독립성 평가
피츠버그 재활 참여 척도 – 재활참여도 평가
Short-form Health Survery 36 version 2 – 삶의 질 평가

③ 센서 신호 측정

가쪽넓은근
앞정강근육

④ 기타

약제: 항경직약, 항우울제, 항경련제 등
정신상태검사
보조기 착용 여부
운동유발전위검사

3) 연구 대상자가 준수해야 할 사항

① 사용 전에는 반드시 사용자 매뉴얼을 숙지해야 한다.
② 사용목적 이외의 사용을 금한다.
③ 배터리 충전 중에는 기기가 작동하지 않는다.
④ 운동이나 샤워 직후에는 측정을 피한다.
⑤ 기타 질환이나 의료적인 문제가 발생하거나 의심될 경우에는 즉시 의료전문가나 담당 의사와 상의한다.
⑥ 사이버 보안 위협 탐지 시 사용설명서에 기재된 제조원 연락처로 연락한다.

7. 대안 치료 (임상시험 이외의 다른 대체 가능한 치료법)

일반적인 운동 재활치료로서 치료사가 환자를 일대일로 지도하며 환자가 스스로 앉고, 서고, 보행을 할 수 있도록 하며, 적절한 보조를 함께 제공하는 치료가 있음.

8. 연구대상자에게 예견되는 부작용, 위험과 불편함

임상 평가는 임상 현장에서 뇌졸중 환자 대상으로 흔히 수행되는 도수근력검사, 브룬스트롬 기능회복 단계, 근육경직척도, 버그균형척도, 푸글마이어 척도, 수정 랭킨 척도, 기능적 보행 분류, 수정 바델 지수, Short-form Health Survery 36 version 2, 피츠버그 재활 참여 스케일, 리버미드 운동

평가 등이 활용되고 있어 기존 진료 프로세스에 비하여 추가적인 위험도는 매우 낮을 것으로 판단됩니다. 근전도와 관성 센서 신호를 측정할 때 수행하는 개별관절운동, 균형 조절, 보행 등 또한 임상 현장에서 흔히 수행되고 있는 뇌졸중 환자의 재활 프로세스이며, 이 동작을 수행할 때 환자의 몸통과 다리에 무선 센서만 부착되는 것이므로 이로 인한 추가적인 위험도는 매우 낮을 것으로 판단됩니다. 운동유발전위검사는 신경생리학적 검사 중 하나로 경두개 자기자극술을 두부에 시행하여 뇌에서 하지 말초에 이르는 피질척수로의 상태를 평가하는 검사로 임상에서 뇌졸중 환자의 예후 판정을 위하여 일상적으로 활용되고 있어 기존 진료 프로세스에 비하여 추가적인 위험도는 낮을 것으로 판단됩니다. 임상에서 아래에 해당하는 환자들은 위험도가 있다고 판단하여 운동유발전위검사를 시행하지 않고 있어 동일한 기준을 적용할 예정입니다.
- 심박조율기 등 이식된 전기자극기 등이 있는 환자
- 간질, 발작의 병력 또는 위험도가 높은 환자
- 두부에 금속성 이물질이 삽입되어 있는 환자

9. 연구대상자에게 예견되는 이득

시험군: 표준 하지재활 치료와 함께 추가적으로 근전도 피드백에 기반한 감독하 자가운동을 진행하기 때문에 충분한 운동량과 횟수를 제공받을 수 있으며, 시험군은 근전도 센서로부터 바이오피드백을 제공받기 때문에 보다 정밀하게 근육의 활성도를 확인하여 효과적으로 자가운동을 할 수 있습니다. 각 시점 별 세부적인 평가를 통해 신체 상태 및 회복의 정도를 확인할 수 있습니다.

대조군: 표준 하지재활 치료에 더하여 각 시점 별 세부적인 평가를 통해 신체 상태 및 회복의 정도를 확인할 수 있습니다.

10. 비용 및 보상

병원 내에서 행해지는 뇌졸중 재활 치료에 대한 사례비는 제공하지 않습니다. 다만 각 측정마다 평가비용을 포함하여 20 만원의 측정 사례비 지급과 12 주차에는 퇴원 후 외래로 방문하셔야 할 경우 추가적으로 교통비 20 만원을 연구진이 연구대상자에게 지원합니다. 입원상태인 경우 교통비를 지급하지 않습니다. 임상시험 참여 후 12 주차측정시점까지 모두 참여하시는 분께는 3 주차 측정이 끝난 후 2 주 이내에 평가비용을 포함하여 40 만원, 12 주차측정이 끝난 후 2 주 이내에 평가비용을 포함하여 20 만원 총 60 만원을 연구진이 연구대상자에게 지급할 예정입니다. 만약 중지 및 탈락이 발생하여 연구를 끝까지 진행하지 못하는 경우 완료된 측정에 대하여 초기 측정 평가비용 포함 20 만원, 3 주차 측정 평가비용 포함 20 만원을 계산하여 3 주차 측정이 완료된 후 2 주일 내에 연구진이 연구대상자에게 지급합니다. 완료지 않은 측정에 대해서는 평가비용과 교통비만을 제공합니다. 단 교통비 제공은 퇴원 후 외래로 방문하였을 경우에만 해당합니다. 본 연구에서 실시되는 모든 연구 비용은 연구진이 지불합니다.

11. 자발적 참여/연구의 중단

연구의 참여 여부는 자발적으로 결정하실 수 있으며, 언제든지 연구 참여를 중도에 포기하실 수 있습니다. 또한 연구자가 연구의 지속이 어렵다고 판단할 경우에도 연구가 중단될 수 있습니다.

12. 연구 관련 새로운 정보의 지속적 제공

연구대상자의 참여에 영향을 줄 수 있는 새로운 정보를 취득할 시 지체 없이 대상자 또는 대상자의 대리인에게 정보를 전달할 것입니다.

13. 손상 및 보상

연구 참여로 인해 예기치 않게 발생하는 부작용 및 손상에 대해 피해자보상규약에 근거하여 보상합니다. 모든 치료의 경비뿐만 아니라 보상을 위해 임상시험 배상보험에 가입하여 대비하고 있습니다.

연락처: 서울대학교병원 재활의학과 연구원 전소연 010-2145-4198

14. 비밀 보장

연구의 대상자의 신상을 파악할 수 있는 개인정보에 관한 기록은 비밀로 보호될 것이며, 연구 데이터는 연구종료 후 3 년간 보관하며, 보관기간이 지난 문서는 개인정보보호법 시행령 제 16 조에 따라 종이 형태의 자료는 파쇄, 컴퓨터 저장 자료는 복원이 불가능한 방법으로 영구 파기합니다. 연구 결과가 출판될 경우 대상자의 신상은 비밀로 보호될 것입니다. 본 연구의 목적으로 수집된 임상 정보 및 센서 신호 정보들은 분석을 위하여 익명화한 상태로 관리됩니다. 임상연구의 모니터요원, 점검을 실시하는 사람, IRB 및 보건복지부장관이 관계 법령에 따라 연구의 절차와 자료의 품질을 검증하기 위하여 대상자의 신상에 관한 비밀이 보호되는 범위에서 대상자의 연구기록을 열람할 수 있으며, 이는 대상자 또는 대상자의 대리인이 서명한 동의서에 의하여 이러한 자료의 열람이 허용됩니다.

15. 담당자 연락처

임상시험에서 발생한 문제, 우려, 질문에 대하여 상의할 담당자의 연락처

서울대학교병원 재활의학과 연구원 전소연 010-2145-4198

연구대상자의 권익에 대한 문제, 우려, 질문이 있을 때 상의할 의학연구윤리심의위원회(IRB) 연락처 (02-2072-0694) 또는 임상연구윤리센터 연락처 (02-2072-3509)

대상자 일련번호	

연구대상자 동의서

1. 본인은 임상시험에 대해 구두로 설명을 받고 상기 연구 설명문을 읽었으며 담당 연구원과 이 연구에 대하여 충분히 의논하였습니다.
2. 본인은 연구의 위험과 이득에 관하여 들었으며 나의 질문에 만족할 만한 답변을 얻었습니다.
3. 본인은 이 연구에 참여하는 것에 대하여 자발적으로 동의합니다.
4. 본인은 이후의 치료에 영향을 받지 않고 언제든지 연구의 참여를 거부하거나 연구의 참여를 중도에 철회할 수 있고 이러한 결정이 나에게 어떠한 해가 되지 않을 것이라는 것을 알고 있습니다.
5. 본인은 이 설명서 및 동의서에 서명함으로써 의학 연구 목적으로 나의 개인정보가 현행 법률과 규정이 허용하는 범위 내에서 연구자가 수집하고 처리하는데 동의합니다.
6. 본인은 연구 설명문 및 동의서의 사본을 받을 것을 알고 있습니다.

______________	______________	______________
연구대상자 성명	서명	날짜(년/월/일)
______________	______________	______________
시험자/연구자 성명	서명	날짜(년/월/일)
______________	______________	______________
법정대리인 성명	서명	날짜(년/월/일)
______________	______________	
(대상자와 대리인과의 관계)	(대리인 동의 사유)	

[별첨 3] 증례기록서

**Screening & Enrollment**	날짜: _ _ _ _ 년 _ _ 월 _ _ 일

Subject Initials: _ _ _

Subject No: _ _ _ _ _ _ _ _ _ _ _ _

**서면동의서**		
● **대상자 또는 대리인에게 서면 동의서를 받았습니까?**	☐ 예	☐ 아니오
● **동의서 서명일 :** _ _ _ _ 년 _ _ 월 _ _ 일		

**선정기준 및 제외기준**			
**선정 기준**	예	아니오	비고
1. 만 19 세 이상의 성인	☐	☐	
2. 뇌졸중 발생 후 3 개월 이내	☐	☐	
3. 뇌졸중으로 인한 하지 위약(MMT =< 4 grade)	☐	☐	
4. 뇌졸중으로 인한 수정랭킨척도(mRS) 2-5 점	☐	☐	
5. 관절 운동, 균형 및 보행 훈련에 참여할 수 있을 정도의 이해 및 명령에 따를 수 있는 인지 능력을 갖춘 사람	☐	☐	
**제외 기준**	예	아니오	비고
1. 연구 기간 내 새로운 신경학적 이상을 유발할 정도의 뇌졸중의 재발	☐	☐	
2. 균형 및 보행 기능에 영향을 줄 정도의 다른 신경학적 이상 (예. 파킨슨 증후군 등)	☐	☐	
3. 균형 및 보행 평가 및 수행이 어려울 정도의 인지기능 저하	☐	☐	
4. 심각한 내과적 상태 (예. 활동성 암)	☐	☐	
5. 체내 전기자극기(인공심박기, 심부뇌자극기 등)을 삽입하여 근전도 측정이 어려운 경우	☐	☐	

MMT & mRS

- **도수근력검사(Manual muscle test, MMT):**
  - **Hip flexor**

    Right - grade 0 ☐ / 1 ☐ / 2 ☐ / 3 ☐ / 4 ☐ / 5 ☐

    Left - grade 0 ☐ / 1 ☐ / 2 ☐ / 3 ☐ / 4 ☐ / 5 ☐
  - **Hip abductor**

    Right - grade 0 ☐ / 1 ☐ / 2 ☐ / 3 ☐ / 4 ☐ / 5 ☐

    Left - grade 0 ☐ / 1 ☐ / 2 ☐ / 3 ☐ / 4 ☐ / 5 ☐

- **Knee extensor**

Right - grade 0 □ / 1 □ / 2 □ / 3 □ / 4 □ / 5 □

Left - grade 0 □ / 1 □ / 2 □ / 3 □ / 4 □ / 5 □

- **Ankle plantar flexor**

Right - grade 0 □ / 1 □ / 2 □ / 3 □ / 4 □ / 5 □

Left - grade 0 □ / 1 □ / 2 □ / 3 □ / 4 □ / 5 □

- **Ankle dorsiflexor**

Right - grade 0 □ / 1 □ / 2 □ / 3 □ / 4 □ / 5 □

Left - grade 0 □ / 1 □ / 2 □ / 3 □ / 4 □ / 5 □

- **수정 랭킨 척도(modified Rankin scale, mRS):**

Score 0 □ / 1 □ / 2 □ / 3 □ / 4 □ / 5 □ / 6 □

인구학적 정보

- **생년월일** : _ _ _ _ 년 _ _ 월 _ _ 일
- **성별** : □ 남자 □ 여자
- **키** _ _ _ cm / **몸무게** _ _ _ kg
- **우세손** : □ 왼손 □ 오른손
- **타질환 동방 유무** : □ 고혈압 □ 당뇨 □ 고지혈증 □ 심방세동
- **Charlson 동반질환지수(Charlson comorbidity index)** :
- 0 □ 1-2 □ 3-4 □ 5+
- **교육수준** : □ 초등학교 □ 중학교 □ 고등학교 졸업
- **돌봄제공자** : □ 배우자 □ 자녀 □ 간병인 □ 기타

뇌졸중 관련정보

- **초기 NIH 뇌졸중 척도(NIHSS)** :

1. 여부 : O / X
2. 검사 시기 : _ _ _ _ 년 _ _ 월 _ _ 일
3. 점수 : _____/42

- **뇌졸중 치료 :**

□ thrombolysis □ thrombectomy

- **뇌졸중 발생 기간** : _ _ 개월
- **뇌졸중 종류 :**

□ Infarction □ Hemorrhage □ N/A

- **뇌졸중 위치 :**

□ 좌 □ 우 / □ 천막상부 □ 천막하부 /

□ 운동영역피질침범(M1) □ 내포침범(internal capsule)

- **뇌졸중 원인 :** TOAST classification

□ large-arteryAtheros-clerosis □ small-vessel occlusion

□ Cardioembolism □ other determined etiology

□ Un-determined etiology

기타 정보

- **약제 :** 항경직약 (O / X), 항우울제 (O / X), 항경련제 (O / X)
- **인지기능 : 간이정신상태검사(Mini-Mental State Examination, MMSE)**

1. 여부 : O / X
2. 검사 시기 : _ _ _ _ 년 _ _ 월 _ _ 일
3. 점수 : _____/30

- **우울 및 불안 : 병원 불안-우울 척도(Hostpial Anxiety and Depression Scale, HADs)**

1. 여부 : O / X
2. 검사 시기 : _ _ _ _ 년 _ _ 월 _ _ 일
3. 점수 : _____/52

- **근육경직척도(Modified Ashworth scale, MAS):**
  - **Hip flexor**

  Right - grade 0 □ / 1 □ / 1+ □ / 2 □ / 3 □ / 4 □

  Left - grade 0 □ / 1 □ / 1+ □ / 2 □ / 3 □ / 4 □

  - **Hip adductor**

  Right - grade 0 □ / 1 □ / 1+ □ / 2 □ / 3 □ / 4 □

  Left - grade 0 □ / 1 □ / 1+ □ / 2 □ / 3 □ / 4 □

  - **Knee extensor**

  Right - grade 0 □ / 1 □ / 1+ □ / 2 □ / 3 □ / 4 □

  Left - grade 0 □ / 1 □ / 1+ □ / 2 □ / 3 □ / 4 □

  - **Ankle plantar flexor**

  Right - grade 0 □ / 1 □ / 1+ □ / 2 □ / 3 □ / 4 □

  Left - grade 0 □ / 1 □ / 1+ □ / 2 □ / 3 □ / 4 □

  - **Ankle dorsiflexor**

  Right - grade 0 □ / 1 □ / 2 □ / 3 □ / 4 □ / 5 □

  Left - grade 0 □ / 1 □ / 2 □ / 3 □ / 4 □ / 5 □

- **보조기** : (O, X)

MEP

- **Affected side**
  - **Response (+ / -)**
    - ◆ **Latency: ____ ms**
    - ◆ **Amplitude: ____ mV**
  - **Resting motor threshold**
- **Unaffected side**
  - **Response (+ / -)**
    - ◆ **Latency: ____ ms**
    - ◆ **Amplitude: ____ mV**
  - **Resting motor threshold**

연구종료
● 날짜: _ _ _ _ 년 _ _ 월 _ _ 일<br>● 연구 종료사유:<br><br>● 이상반응:

**Baseline**			날짜: _ _ _ _ 년 _ _ 월 _ _ 일		
**연구자 성명**		**서명**		**서명일**	
**Evaluation 1: at 1 day**			날짜: _ _ _ _ 년 _ _ 월 _ _ 일		

평가항목

- **리버미드 운동 평가(Rivermead motor assessment, RMA):**

Gross function: ____/13, Leg and trunk: ____/10

- **기능적 보행 분류(Functional ambulation category, FAC):**

Stage 0 □ / 1 □ / 2 □ / 3 □ / 4 □ / 5 □

- **수정 랭킨 척도(modified Rankin scale, mRS):**

Score 0 □ / 1 □ / 2 □ / 3 □ / 4 □ / 5 □ / 6 □

- **버그균형척도(Berg balance scale, BBS):**

Total: ____/56

0~20: high fall risk □ 21~40: medium fall risk □ 41~56: low fall risk □

- **도수근력검사(Manual muscle test, MMT):**
  - **Hip flexor**

Right - grade 0 □ / 1 □ / 2 □ / 3 □ / 4 □ / 5 □

Left - grade 0 □ / 1 □ / 2 □ / 3 □ / 4 □ / 5 □

  - **Hip abductor**

Right - grade 0 □ / 1 □ / 2 □ / 3 □ / 4 □ / 5 □

Left - grade 0 □ / 1 □ / 2 □ / 3 □ / 4 □ / 5 □

  - **Knee extensor**

Right - grade 0 □ / 1 □ / 2 □ / 3 □ / 4 □ / 5 □

Left - grade 0 □ / 1 □ / 2 □ / 3 □ / 4 □ / 5 □

  - **Ankle plantar flexor**

Right - grade 0 □ / 1 □ / 2 □ / 3 □ / 4 □ / 5 □

Left - grade 0 □ / 1 □ / 2 □ / 3 □ / 4 □ / 5 □

  - **Ankle dorsiflexor**

Right - grade 0 □ / 1 □ / 2 □ / 3 □ / 4 □ / 5 □

Left - grade 0 □ / 1 □ / 2 □ / 3 □ / 4 □ / 5 □

- **브룬스트롬 기능회복 단계(brunnstrom stages of motor recovery):**

Grade 1 □ / 2 □ / 3 □ / 4 □ / 5 □ / 6 □

- **근육경직척도(Modified Ashworth scale, MAS):**
  - **Hip flexor**

Right - grade 0 □ / 1 □ / 1+ □ / 2 □ / 3 □ / 4 □

Left - grade 0 □ / 1 □ / 1+ □ / 2 □ / 3 □ / 4 □

  - **Hip adductor**

Right - grade 0 □ / 1 □ / 1+ □ / 2 □ / 3 □ / 4 □

Left - grade 0 □ / 1 □ / 1+ □ / 2 □ / 3 □ / 4 □

  - **Knee extensor**

Right - grade 0 □ / 1 □ / 1+ □ / 2 □ / 3 □ / 4 □

Left - grade 0 □ / 1 □ / 1+ □ / 2 □ / 3 □ / 4 □

- **Ankle plantar flexor**

Right - grade 0 □ / 1 □ / 1+ □ / 2 □ / 3 □ / 4 □

Left - grade 0 □ / 1 □ / 1+ □ / 2 □ / 3 □ / 4 □

- **Ankle dorsiflexor**

Right - grade 0 □ / 1 □ / 2 □ / 3 □ / 4 □ / 5 □

Left - grade 0 □ / 1 □ / 2 □ / 3 □ / 4 □ / 5 □

- **푸글마이어 척도-하지(Fugl-Meyer assessment of lower extremity, FMS-LE):**

Lower extremity Total (max=34) Rt. _____ Lt. _____

- **수정 바델 지수(modified Barthel index, MBI):** _ _ _ / 100
    - Chair/Bed transfer : 0 □ / 3 □ / 8 □ / 12 □ / 15 □
    - Ambulation : 0 □ / 3 □ / 8 □ / 12 □ / 15 □
    - Wheel chair : 0 □ / 1 □ / 3 □ / 4 □ / 5 □
    - Stair climbing : 0 □ / 2 □ / 5 □ / 8 □ / 10 □
    - Toilet transfer : 0 □ / 2 □ / 5 □ / 8 □ / 10 □
    - Bowel control : 0 □ / 2 □ / 5 □ / 8 □ / 10 □
    - Bladder control : 0 □ / 2 □ / 5 □ / 8 □ / 10 □
    - Bathing : 0 □ / 1 □ / 3 □ / 4 □ / 5 □
    - Dressing : 0 □ / 2 □ / 5 □ / 8 □ / 10 □
    - Personal hygiene : 0 □ / 1 □ / 3 □ / 4 □/ 5 □
    - Feeding : 0 □ / 2 □ / 5 □ / 8 □ / 10 □
- **Short-form Health Survery 36 version 2(SF-36v2)**:
    - your health : _ _
    - your health in general : _ _
    - your health now limit : _ _
    - past 4 weeks, had any of physical problems : _ _
    - past 4 weeks, had any of emotional problems : _ _
    - past 4 weeks, has your problems interfered with family, fiends : _ _
    - past 4 weeks, bodily pain : _ _
    - past 4 weeks, how you feel : _ _
    - past 4 weeks, has problems interfered with your social activities : _ _
    - statements for you : _ _
- **피츠버그 재활 참여 척도(Pittsburgh Rehabilitation Participation Scale, PRPS):**

Score 1 □ / 2 □ / 3 □ / 4 □ / 5 □ / 6 □

연구종료
● 날짜: _ _ _ _ 년 _ _ 월 _ _ 일<br>● 연구 종료사유:<br><br>● 이상반응:

1Weeks			날짜: _ _ _ _ 년 _ _ 월 _ _ 일		
**연구자 성명**		**서명**		**서명일**	
**Evaluation 2: at 1 week**			날짜: _ _ _ _ 년 _ _ 월 _ _ 일		

Subject Initials: _ _ _

Subject No: _ _ _ _ _ _ _ _ _ _ _ _

운동프로토콜 적용을 위한 평가 지표
● **도수근력검사(Manual muscle test, MMT):**<br>• **Hip flexor**<br>Right - grade 0 □ / 1 □ / 2 □ / 3 □ / 4 □ / 5 □<br>Left - grade 0 □ / 1 □ / 2 □ / 3 □ / 4 □ / 5 □<br>• **Hip abductor**<br>Right - grade 0 □ / 1 □ / 2 □ / 3 □ / 4 □ / 5 □<br>Left - grade 0 □ / 1 □ / 2 □ / 3 □ / 4 □ / 5 □<br>• **Knee extensor**<br>Right - grade 0 □ / 1 □ / 2 □ / 3 □ / 4 □ / 5 □<br>Left - grade 0 □ / 1 □ / 2 □ / 3 □ / 4 □ / 5 □<br>• **Ankle plantar flexor**<br>Right - grade 0 □ / 1 □ / 2 □ / 3 □ / 4 □ / 5 □<br>Left - grade 0 □ / 1 □ / 2 □ / 3 □ / 4 □ / 5 □<br>• **Ankle dorsiflexor**<br>Right - grade 0 □ / 1 □ / 2 □ / 3 □ / 4 □ / 5 □<br>Left - grade 0 □ / 1 □ / 2 □ / 3 □ / 4 □ / 5 □<br>● **브룬스트롬 기능회복 단계(brunnstrom stages of motor recovery):**<br>Grade 1 □ / 2 □ / 3 □ / 4 □ / 5 □ / 6 □<br>● **피츠버그 재활 참여 척도(Pittsburgh Rehabilitation Participation Scale, PRPS):**<br>Score 1 □ / 2 □ / 3 □ / 4 □ / 5 □ / 6 □

연구종료
● 날짜: _ _ _ _ 년 _ _ 월 _ _ 일<br>● 연구 종료사유:<br><br>● 이상반응:

2Weeks			날짜: _ _ _ _ 년 _ _ 월 _ _ 일		
**연구자 성명**		**서명**		**서명일**	
**Evaluation 3: at 2 week**			날짜: _ _ _ _ 년 _ _ 월 _ _ 일		

Subject Initials: _ _ _

Subject No: _ _ _ _ _ _ _ _ _ _ _ _

**운동프로토콜 적용을 위한 평가 지표**
● **도수근력검사(Manual muscle test, MMT):**<br>• **Hip flexor**<br>Right - grade 0 □ / 1 □ / 2 □ / 3 □ / 4 □ / 5 □<br>Left - grade 0 □ / 1 □ / 2 □ / 3 □ / 4 □ / 5 □<br>• **Hip abductor**<br>Right - grade 0 □ / 1 □ / 2 □ / 3 □ / 4 □ / 5 □<br>Left - grade 0 □ / 1 □ / 2 □ / 3 □ / 4 □ / 5 □<br>• **Knee extensor**<br>Right - grade 0 □ / 1 □ / 2 □ / 3 □ / 4 □ / 5 □<br>Left - grade 0 □ / 1 □ / 2 □ / 3 □ / 4 □ / 5 □<br>• **Ankle plantar flexor**<br>Right - grade 0 □ / 1 □ / 2 □ / 3 □ / 4 □ / 5 □<br>Left - grade 0 □ / 1 □ / 2 □ / 3 □ / 4 □ / 5 □<br>• **Ankle dorsiflexor**<br>Right - grade 0 □ / 1 □ / 2 □ / 3 □ / 4 □ / 5 □<br>Left - grade 0 □ / 1 □ / 2 □ / 3 □ / 4 □ / 5 □<br>● **브룬스트롬 기능회복 단계(brunnstrom stages of motor recovery):**<br>Grade 1 □ / 2 □ / 3 □ / 4 □ / 5 □ / 6 □<br>● **피츠버그 재활 참여 척도(Pittsburgh Rehabilitation Participation Scale, PRPS):**<br>Score 1 □ / 2 □ / 3 □ / 4 □ / 5 □ / 6 □

**연구종료**
● 날짜: _ _ _ _ 년 _ _ 월 _ _ 일<br>● 연구 종료사유:<br><br>● 이상반응:

3Weeks			날짜: _ _ _ _ 년 _ _ 월 _ _ 일		
**연구자 성명**		**서명**		**서명일**	
**Evaluation 4: at 3week**			날짜: _ _ _ _ 년 _ _ 월 _ _ 일		

평가항목

- **리버미드 운동 평가(Rivermead motor assessment, RMA):**

Gross function: ____/13, Leg and trunk: ____/10

- **기능적 보행 분류(Functional ambulation category, FAC):**

Stage 0 □ / 1 □ / 2 □ / 3 □ / 4 □ / 5 □

- **수정 랭킨 척도(modified Rankin scale, mRS):**

Score 0 □ / 1 □ / 2 □ / 3 □ / 4 □ / 5 □ / 6 □

- **버그균형척도(Berg balance scale, BBS):**

Total: ____/56

0~20: high fall risk □ 21~40: medium fall risk □ 41~56: low fall risk □

- **도수근력검사(Manual muscle test, MMT):**
  - **Hip flexor**

Right - grade 0 □ / 1 □ / 2 □ / 3 □ / 4 □ / 5 □

Left - grade 0 □ / 1 □ / 2 □ / 3 □ / 4 □ / 5 □

  - **Hip abductor**

Right - grade 0 □ / 1 □ / 2 □ / 3 □ / 4 □ / 5 □

Left - grade 0 □ / 1 □ / 2 □ / 3 □ / 4 □ / 5 □

  - **Knee extensor**

Right - grade 0 □ / 1 □ / 2 □ / 3 □ / 4 □ / 5 □

Left - grade 0 □ / 1 □ / 2 □ / 3 □ / 4 □ / 5 □

  - **Ankle plantar flexor**

Right - grade 0 □ / 1 □ / 2 □ / 3 □ / 4 □ / 5 □

Left - grade 0 □ / 1 □ / 2 □ / 3 □ / 4 □ / 5 □

  - **Ankle dorsiflexor**

Right - grade 0 □ / 1 □ / 2 □ / 3 □ / 4 □ / 5 □

Left - grade 0 □ / 1 □ / 2 □ / 3 □ / 4 □ / 5 □

- **브룬스트롬 기능회복 단계(brunnstrom stages of motor recovery):**

Grade 1 □ / 2 □ / 3 □ / 4 □ / 5 □ / 6 □

- **근육경직척도(Modified Ashworth scale, MAS):**
  - **Hip flexor**

Right - grade 0 □ / 1 □ / 1+ □ / 2 □ / 3 □ / 4 □

Left - grade 0 □ / 1 □ / 1+ □ / 2 □ / 3 □ / 4 □

  - **Hip adductor**

Right - grade 0 □ / 1 □ / 1+ □ / 2 □ / 3 □ / 4 □

Left - grade 0 □ / 1 □ / 1+ □ / 2 □ / 3 □ / 4 □

  - **Knee extensor**

Right - grade 0 □ / 1 □ / 1+ □ / 2 □ / 3 □ / 4 □

Left - grade 0 □ / 1 □ / 1+ □ / 2 □ / 3 □ / 4 □

- **Ankle plantar flexor**

Right - grade 0 □ / 1 □ / 1+ □ / 2 □ / 3 □ / 4 □

Left - grade 0 □ / 1 □ / 1+ □ / 2 □ / 3 □ / 4 □

- **Ankle dorsiflexor**

Right - grade 0 □ / 1 □ / 2 □ / 3 □ / 4 □ / 5 □

Left - grade 0 □ / 1 □ / 2 □ / 3 □ / 4 □ / 5 □

- **푸글마이어 척도-하지(Fugl-Meyer assessment of lower extremity, FMS-LE):**

Lower extremity Total (max=34) Rt. _____ Lt. _____

- **수정 바델 지수(modified Barthel index, MBI):** _ _ _ / 100
  - Chair/Bed transfer : 0 □ / 3 □ / 8 □ / 12 □ / 15 □
  - Ambulation : 0 □ / 3 □ / 8 □ / 12 □ / 15 □
  - Wheel chair : 0 □ / 1 □ / 3 □ / 4 □ / 5 □
  - Stair climbing : 0 □ / 2 □ / 5 □ / 8 □ / 10 □
  - Toilet transfer : 0 □ / 2 □ / 5 □ / 8 □ / 10 □
  - Bowel control : 0 □ / 2 □ / 5 □ / 8 □ / 10 □
  - Bladder control : 0 □ / 2 □ / 5 □ / 8 □ / 10 □
  - Bathing : 0 □ / 1 □ / 3 □ / 4 □ / 5 □
  - Dressing : 0 □ / 2 □ / 5 □ / 8 □ / 10 □
  - Personal hygiene : 0 □ / 1 □ / 3 □ / 4 □/ 5 □
  - Feeding : 0 □ / 2 □ / 5 □ / 8 □ / 10 □
- **Short-form Health Survery 36 version 2(SF-36v2)**:
  - your health : _ _
  - your health in general : _ _
  - your health now limit : _ _
  - past 4 weeks, had any of physical problems : _ _
  - past 4 weeks, had any of emotional problems : _ _
  - past 4 weeks, has your problems interfered with family, fiends : _ _
  - past 4 weeks, bodily pain : _ _
  - past 4 weeks, how you feel : _ _
  - past 4 weeks, has problems interfered with your social activities : _ _
  - statements for you : _ _
- **피츠버그 재활 참여 척도(Pittsburgh Rehabilitation Participation Scale, PRPS):**

Score 1 □ / 2 □ / 3 □ / 4 □ / 5 □ / 6 □

연구종료
● 날짜: _ _ _ _ 년 _ _ 월 _ _ 일<br>● 연구 종료사유:<br><br>● 이상반응:

12Weeks			날짜: _ _ _ _ 년 _ _ 월 _ _ 일		
연구자 성명		서명		서명일	
Evaluation 5: at 12week			날짜: _ _ _ _ 년 _ _ 월 _ _ 일		

평가항목

- **리버미드 운동 평가(Rivermead motor assessment, RMA):**

Gross function: ____/13, Leg and trunk: ____/10

- **기능적 보행 분류(Functional ambulation category, FAC):**

Stage 0 □ / 1 □ / 2 □ / 3 □ / 4 □ / 5 □

- **수정 랭킨 척도(modified Rankin scale, mRS):**

Score 0 □ / 1 □ / 2 □ / 3 □ / 4 □ / 5 □ / 6 □

- **버그균형척도(Berg balance scale, BBS):**

Total: ____/56

0~20: high fall risk □ 21~40: medium fall risk □ 41~56: low fall risk □

- **도수근력검사(Manual muscle test, MMT):**
  - **Hip flexor**

Right - grade 0 □ / 1 □ / 2 □ / 3 □ / 4 □ / 5 □

Left - grade 0 □ / 1 □ / 2 □ / 3 □ / 4 □ / 5 □

  - **Hip abductor**

Right - grade 0 □ / 1 □ / 2 □ / 3 □ / 4 □ / 5 □

Left - grade 0 □ / 1 □ / 2 □ / 3 □ / 4 □ / 5 □

  - **Knee extensor**

Right - grade 0 □ / 1 □ / 2 □ / 3 □ / 4 □ / 5 □

Left - grade 0 □ / 1 □ / 2 □ / 3 □ / 4 □ / 5 □

  - **Ankle plantar flexor**

Right - grade 0 □ / 1 □ / 2 □ / 3 □ / 4 □ / 5 □

Left - grade 0 □ / 1 □ / 2 □ / 3 □ / 4 □ / 5 □

  - **Ankle dorsiflexor**

Right - grade 0 □ / 1 □ / 2 □ / 3 □ / 4 □ / 5 □

Left - grade 0 □ / 1 □ / 2 □ / 3 □ / 4 □ / 5 □

- **브룬스트롬 기능회복 단계(brunnstrom stages of motor recovery):**

Grade 1 □ / 2 □ / 3 □ / 4 □ / 5 □ / 6 □

- **근육경직척도(Modified Ashworth scale, MAS):**
  - **Hip flexor**

Right - grade 0 □ / 1 □ / 1+ □ / 2 □ / 3 □ / 4 □

Left - grade 0 □ / 1 □ / 1+ □ / 2 □ / 3 □ / 4 □

  - **Hip adductor**

Right - grade 0 □ / 1 □ / 1+ □ / 2 □ / 3 □ / 4 □

Left - grade 0 □ / 1 □ / 1+ □ / 2 □ / 3 □ / 4 □

  - **Knee extensor**

Right - grade 0 □ / 1 □ / 1+ □ / 2 □ / 3 □ / 4 □

Left - grade 0 □ / 1 □ / 1+ □ / 2 □ / 3 □ / 4 □

- **Ankle plantar flexor**

Right - grade 0 □ / 1 □ / 1+ □ / 2 □ / 3 □ / 4 □

Left - grade 0 □ / 1 □ / 1+ □ / 2 □ / 3 □ / 4 □

- **Ankle dorsiflexor**

Right - grade 0 □ / 1 □ / 2 □ / 3 □ / 4 □ / 5 □

Left - grade 0 □ / 1 □ / 2 □ / 3 □ / 4 □ / 5 □

- **푸글마이어 척도-하지(Fugl-Meyer assessment of lower extremity, FMS-LE):**

Lower extremity Total (max=34) Rt. _____ Lt. _____

- **수정 바델 지수(modified Barthel index, MBI):** _ _ _ / 100
  - Chair/Bed transfer : 0 □ / 3 □ / 8 □ / 12 □ / 15 □
  - Ambulation : 0 □ / 3 □ / 8 □ / 12 □ / 15 □
  - Wheel chair : 0 □ / 1 □ / 3 □ / 4 □ / 5 □
  - Stair climbing : 0 □ / 2 □ / 5 □ / 8 □ / 10 □
  - Toilet transfer : 0 □ / 2 □ / 5 □ / 8 □ / 10 □
  - Bowel control : 0 □ / 2 □ / 5 □ / 8 □ / 10 □
  - Bladder control : 0 □ / 2 □ / 5 □ / 8 □ / 10 □
  - Bathing : 0 □ / 1 □ / 3 □ / 4 □ / 5 □
  - Dressing : 0 □ / 2 □ / 5 □ / 8 □ / 10 □
  - Personal hygiene : 0 □ / 1 □ / 3 □ / 4 □/ 5 □
  - Feeding : 0 □ / 2 □ / 5 □ / 8 □ / 10 □
- **Short-form Health Survery 36 version 2(SF-36v2)**:
  - your health : _ _
  - your health in general : _ _
  - your health now limit : _ _
  - past 4 weeks, had any of physical problems : _ _
  - past 4 weeks, had any of emotional problems : _ _
  - past 4 weeks, has your problems interfered with family, fiends : _ _
  - past 4 weeks, bodily pain : _ _
  - past 4 weeks, how you feel : _ _
  - past 4 weeks, has problems interfered with your social activities : _ _
  - statements for you : _ _
- **피츠버그 재활 참여 척도(Pittsburgh Rehabilitation Participation Scale, PRPS):**

Score 1 □ / 2 □ / 3 □ / 4 □ / 5 □ / 6 □

연구종료
● 날짜: _ _ _ _ 년 _ _ 월 _ _ 일<br>● 연구 종료사유:<br><br>● 이상반응:

[별첨 4] 연구대상자 모집 공고문

아급성기 뇌졸중 환자에서 근전도 센서 피드백에 기반한 침상내 자가운동의 기능적 이동성에 대한 효과연구

연구 대상자 모집

연구 개요

연구목적 : 아급성기 뇌졸중 환자에서 근전도 센서 피드백에 기반한 침상내 자가운동의 기능적 이동성에 대한 효과확인

연구책임자 : 서울대학교병원 재활의학과 임상조교수 이우형

연구 방법

- 시험군 또는 대조군에 1:1 비율로 무작위 배정
- 참여기간은 12주로 예상
- 임상시험 기간 동안 기준시점- 3주 -12주 총 3번의 평가를 진행
- 시험군에게는 자가운동프로그램 수행 시 근전도 장치를 부착하여 근수축 여부 및 정도의 정보를 30분씩 하루 2회를 제공, 대조군은 표준하지 재활만을 수행

대상자 조건

<아급성기 뇌졸중 환자>

✓ **선정기준**
- 만 19세 이상
- 뇌졸중 발생 3개월 이내
- 도수근력검사(MMT) 4등급 이하
- 수정랭킨척도(mRS) 2-5점
- 훈련에 참여할 수 있을 정도의 이해 및 명령에 따를 수 있는 인지 능력

✓ **제외기준**
- 연구 기간 내 새로운 뇌졸중의 재발
- 다른 신경학적 이상 (파킨슨 증후군 등)
- 수행이 어려울 정도의 인지기능 저하
- 심각한 내과적 상태 (예. 활동성 암)
- 체내 전기자극기(인공심박기 등)을 삽입하여 근전도 측정이 어려운 경우

장소, 참여 기간 및 보상

- 장소: 서울대학교병원, 국립교통재활병원 재활의학과
- 뇌졸중 환자 12주(기준시점 – 3주 – 12주)
- 시험 참여시 연구대상자 평가비용 지급

연락처

서울대학교병원 재활의학과 연구원 전소연 (010-2145-4198)

SNUH 서울대학교병원

[별첨 5] 임상시험 피해자 보상규약

피해보상에 대한 규약

본 연구는 아급성기 뇌졸중 환자에서 근전도 센서 피드백에 기반한 침상내 자가운동의 기능적 이동성에 대한 효과 연구로서, 기존 서울대학교병원에 진행되고 있는 뇌졸중환자 재활치료에 추가적으로 자가 침상운동 적용에 대한 연구입니다. 의학적으로 판단하여, 연구로 인한 추가적 위험이 기존 일반적인 뇌졸중환자 재활보다 추가적인 위험이 현저하지 않으므로, 이 연구로 인해 연구대상자가 추가적으로 입게 되는 신체적, 정신적, 위해 및 특이 손상은 없을 것으로 예측됩니다.
연구대상자들에게는 통상적인 진료과정에서 이루어지는 안전 보호 대책이 적용될 것이며, 연구시작 전 연구대상자들에게 해당 연구의 목적과 방법 등에 대한 충분한 정보가 제공될 것입니다.
또한, 임상시험 도중, 기존 진료과정 외 임상시험 참여로 추가된 절차 및 개입으로 인해, 예측한 또는 예측되지 못한 이상반응 등이 발생할 경우, 연구대상자가 적절한 의학적 처치를 받을 수 있도록 가능한 최선의 치료를 시행할 것이며, 가입된 임상시험 배상보험을 통해 임상시험과 손상 사이의 합리적인 인과관계가 있는 경우 시험대상자에게 보상하겠습니다.

1. 피해 보상 기준

1) 임상시험의 참여로 인한 손상에 의한 보상 기준은 임상시험계획서에 따라 행해진 자가운동에 의한 손상임.
2) 환자 또는 보호자는 자가운동 도중 발생할 수 있는 위험을 방지하고자 노력하였음에도 불구하고 발생한 손상이어야 함
3) 임상시험의 참여로 인하여 대상자의 신체적인 손상(사망포함)이 발생한 경우 대상자에게 보상함.
4) 임상시험의 참여로 인한 대상자의 신체적 손상이 발생할 경우, 금전적 보상이 확정되기 이전에 책임연구자를 통한 적절한 치료를 우선적으로 제공함.

5) 이상반응이나 이상반응 처치 과정에서 발생된 손상이 있는 경우도 보상의 대상으로 고려함.

2. 피해 보상 제외 기준

1) 임상시험의 참여로 인한 신체적인 손상(사망포함)이 아닌 경우는 보상의 대상에서 제외함.
2) 임상시험 중이 아니어도 일어났을 것이라고 생각되는 사고 등에 기인한 것은 보상의 대상에서 제외함.
3) 대조군인 표준 하지재활만을 시행한 대상자에게 치료상의 이익을 제공할 수 없는 경우에도 보상의 대상에서 제외함.
4) 질병의 악화 또는 진행 결과 중 발생하는 통상적 합병증에 의한 손상은 제외함
5) 대상자 또는 그 보호자의 시험자의 지시사항 및 임상시험계획서 미준수, 고의 또는 중대한 과실로 손상이 발생한 경우 보상액을 줄이거나 보상의 대상에서 제외함.

2022 년 07 월 26 일

책임연구자: 이 우 형 (인)

[별첨 6] 책임연구자의 이력

I. 인적사항 및 학.경력

(1) 인적사항

성명	국 문	이 우 형			전 공	재활의학
	영 문	Woo Hyung Lee				
소속	기관명	서울대학교병원	직위	임상조교수	연락처	02-2072-4178
	주소	서울 종로구 대학로 101<br>서울대학교병원 재활의학과			FAX	02-6072-5244
주민등록번호		850909-1155610			E-mail	whlee909@snu.ac.kr

(2) 학력

구분	학교	전공	졸업년도	학위취득여부
대학교	서울대학교	의학과	2011	학사
대학원	서울대학교	의공학과	2020	박사<br>(석박사통합과정)
최종학위<br>논문명	뇌졸중과 파킨슨병 환자의 삼킴에 대한 운동학적 특징 분석 및 뇌졸중 후 삼킴곤란의 예후 예측 모델 개발			

(3) 주요 최근 경력

연도 (부터 – 까지)	기 관	직 위
2011-2012	서울대학교병원	인턴

2012 - 2016	서울대학교병원	전공의
2016 - 2017	서울대학교병원	전임의
2020 - 현재	서울대학교병원	임상조교수

II. 수상경력

- 2020, *Best Scientific Award*, 14 Nov 2020, The Korean Dysphagia Society
- 2020, *Young Scientist Award*, 13 Oct 2020, The Korean Academy of Rehabilitation Medicine
- 2018, *Best Scientific Award*, The Korean Geriatric Society, 24 Mar 2018, Seo Jung Yun, Hyun Haeng Lee, **Woo Hyung Lee**, Seung Hak Lee, Byung-Mo Oh, Han Gil Seo, "Effect of Robot-assisted Gait Training on Gait Automaticity in Parkinson's Disease: A Pilot Study"
- 2017, *Bronze Paper Award*, IEEE Seoul Section, 2 Dec 2017, Honggeun Cho, Da Jung Park, Sharon Choi, Nhayoung Hong, **Woo Hyung Lee**, and Sungwan Kim, "Development of Vision and Endoscope Control System of Surgical Robots Implemented with Virtual Reality Headset and Attitude Heading Reference System"
- 2016, *Best Resident Researcher*, 22 Feb 2016, Seoul National University Hospital
- 2016, *Best Resident Researcher*, 16 Feb 2016, Department of Rehabilitation Medicine, Seoul National University Hospital
- 2014, *Best Resident*, 6 Dec 2014, Department of Rehabilitation Medicine, Seoul National University College of Medicine

III. 논문실적

- **In 2021**

1. **Lee WH,** Lim MH, Seo HG, Oh BM, Kim S. Hyoid kinematic features for poor swallowing prognosis in patients with post-stroke dysphagia. Sci Rep. 2021 Jan

14;11(1):1471.

2. Yun SJ, Lee HH, **Lee WH**, Lee SH, Oh BM, Seo HG. Effect of robot-assisted gait training on gait automaticity in Parkinson disease: A prospective, open-label, single-arm, pilot study. Medicine (Baltimore). 2021 Feb 5;100(5):e24348.
3. Lim MH, **Lee WH**, Jeon B, Kim S. A Blood Glucose Control Framework Based on Reinforcement Learning With Safety and Interpretability: In Silico Validation. IEEE ACCESS. 2021 Aug

- **In 2020**

1. **Lee WH**, Lim MH, Seo HG, Seong MY, Oh BM, Kim S. Development of a Novel Prognostic Model to Predict 6-Month Swallowing Recovery After Ischemic Stroke. Stroke. 2020 Feb;51(2):440-448.
2. Lee D, **Lee WH (Co-first author)**, Seo HG, Oh BM, Lee JC, Kim HC. Online Learning for the Hyoid Bone Tracking During Swallowing With Neck Movement Adjustment Using Semantic Segmentation. IEEE Access. 2020, 8, 157451-157461.
3. Jung Y, Kim Y, **Lee WH**, Bang MS, Kim Y, Kim S. Path Planning Algorithm for an Autonomous Electric Wheelchair in Hospitals. IEEE ACCESS. 2020; 208199 – 208213

- **In 2019**

1. **Lee WH(Co-first author)**, Lim MH, Nam HS, Kim YJ, Seo HG, Bang MS, Seong MY, Oh BM, Kim S. Differential kinematic features of the hyoid bone during swallowing in patients with Parkinson's disease. J Electromyogr Kinesiol. 2019 Aug;47:57-64.
2. **Lee WH**, Park CK, Park HP, Kim SM, Oh BM, Kim K, Choi YD, Seo HG. Effect of Dexmedetomidine Combined Anesthesia on Motor evoked Potentials During Brain Tumor Surgery. World Neurosurg. 2019 Mar;123:e280-e287.
3. **Lee WH**, Kim E, Seo HG, Oh BM, Nam HS, Kim YJ, Lee HH, Kang MG, Kim S, Bang MS. Target-oriented motor imagery for grasping action: different characteristics of brain activation between kinesthetic and visual imagery. Sci Rep. 2019 Sep; 4;9(1):12770.

4. Nam HS, **Lee WH**, Seo HG, Kim YJ, Bang MS, Kim S. Inertial Measurement Unit Based Upper Extremity Motion Characterization for Action Research Arm Test and Activities of Daily Living. Sensors (Basel). 2019 Apr 14;19(8):1782.
5. Kim YJ, Nam HS, **Lee WH**, Seo HG, Leigh JH, Oh BM, Bang MS, Kim S. Vision-aided brain-machine interface training system for robotic arm control and clinical application on two patients with cervical spinal cord injury. Biomed Eng Online. 2019 Feb 11;18(1):14.

- **In 2018**

1. Oh BM, Lee JH, Seo HG, **Lee WH**, Han TR, Jeong SU, Jeong HJ, Sim YJ. Changes in Hyolaryngeal Movement During Swallowing in the Lateral Decubitus Posture. Ann Rehabil Med. 2018 Jun 27;42(3):416-424.
2. Yang EJ, Kim SY, **Lee WH**, Lim JY, Lee J. Diagnostic Accuracy of Clinical Measures Considering Segmental Tissue Composition and Volume Changes of Breast Cancer-Related Lymphedema. Lymphat Res Biol. 2018 Aug;16(4):368-376.
3. Kim S, Hwang J, Kim MJ, Lim JY, **Lee WH**, Choi JE. SYSTEMATIC REVIEW WITH NETWORK META-ANALYSIS OF RANDOMIZED CONTROLLED TRIALS OF ROTATOR CUFF TEAR TREATMENT. Int J Technol Assess Health Care. 2018 Feb 22:1-9.
4. Lee HH, Seo HG, Kim KD, Lee SH, **Lee WH**, Oh BM, Lee WW, Kim Y, Kim A, Kim HJ, Jeon B, Han TR. Characteristics of Early Oropharyngeal Dysphagia in Patients with Multiple System Atrophy. Neurodegener Dis. 2018;18(2-3):84-90.

- **In 2017**

1. **Lee WH**, Chun C, Seo HG, Lee SH, Oh BM. STAMPS: development and verification of swallowing kinematic analysis software. Biomed Eng Online. 2017 Oct 17;16(1):120.
2. Seo HG, **Lee WH**, Lee SH, Yi Y, Kim KD, Oh BM. Robotic-assisted gait training combined with transcranial direct current stimulation in chronic stroke patients: A pilot double-blind, randomized controlled trial. Restor Neurol Neurosci. 2017;35(5):527-536.

3. Nam HS, Koh S, Kim YJ, Beom J, **Lee WH**, Lee SU, Kim S. Biomechanical Reactions of Exoskeleton Neurorehabilitation Robots in Spastic Elbows and Wrists. IEEE Trans Neural Syst Rehabil Eng. 2017 Nov;25(11):2196-2203.
4. Seo HG, Kim JG, Nam HS, **Lee WH**, Han TR, Oh BM. Swallowing Function and Kinematics in Stroke Patients with Tracheostomies. Dysphagia. 2017 Jun;32(3):393-400.
5. Lee HH, **Lee WH**, Seo HG, Han D, Kim Y, Oh BM. Current State and Prospects of Development of Blood-based Biomarkers for Mild Traumatic Brain Injury. Brain Neurorehabil. 2017 Mar; 10(1): e3

- **In 2016**

1. **Lee WH**, Chun C, Seo HG, Lee SH, Oh BM. STAMPS: development and verification of swallowing kinematic analysis software. Biomed Eng Online. 2017 Oct 17;16(1):120. _
2. **Lee WH**, Lee SU, Jung SH. Ischemic Cervical Myelopathy Caused by Vertebral Artery Dissection: The Clinical Utility of a Motor-evoked Potential Study. Neurologist. 2016 Jan;21(1):8-10.
3. **Lee WH**, Do HK, Lee JH, Kim BR, Noh JH, Choi SH, Chung SG, Lee SU, Choi JE, Kim S, Kim MJ, Lim JY. Clinical Outcomes of Conservative Treatment and Arthroscopic Repair of Rotator Cuff Tears: A Retrospective Observational Study. Ann Rehabil Med. 2016 Apr;40(2):252-62.
4. Lee JC, Seo HG, **Lee WH**, Kim HC, Han TR, Oh BM. Computer-assisted detection of swallowing difficulty. Comput Methods Programs Biomed. 2016 Oct;134:79-88.
5. Lee WK, Yeom J, **Lee WH**, Seo HG, Oh BM, Han TR. Characteristics of Dysphagia in Severe Traumatic Brain Injury Patients: A Comparison With Stroke Patients. Ann Rehabil Med. 2016 Jun;40(3):432-9.
6. Hwang H, Park J, Lee WK, **Lee WH**, Leigh JH, Lee JJ, Chung SG, Lim C, Park SJ, Kim K. Crystallization of Local Anesthetics When Mixed With Corticosteroid Solutions. Ann Rehabil Med. 2016 Feb;40(1):21-7.

IV. 특허출원 및 등록

No	발명의 명칭	출원일	출원번호	등록일	등록번호	발명자
1	뇌-기계 인터페이스 로봇 제어 시스템	2016.11.15	10-2016-0151968	10-19342720000	2018.12.26	방문석, 김성완, 오병모, 서한길
2	온라인 기반의 건강 관리 방법 및 장치	2017.12.27	10-2017-0181156	10-21166640000	2020.05.25	이우형, 임민혁, 최재현
3	학습 기반의 증상 및 질환 관리 장치 및 방법	2019.4.22	10-2019-0046587	10-23389640000	2021.11.15	이우형, 임민혁
4	뇌졸중 환자의 연하곤란 예후 예측을 위한 정보제공방법	2020.02.12	10-2020-0016837			김성완, 오병모, 이우형, 서한길
5	자동 설골 추적 알고리즘	2021.2.9	10-2021-0019295			오병모, 이정찬, 이우형, 이동헌,
6	침상 재활기기	2021.9.1	10-2021-0116588			이우형, 신형익, 성관식, 위수현
7	영아의 자발적 움직임의 복잡도 자동 평가 방법	2021.10.7	10-2021-0133324			이우형, 신형익, 방문석, 신현이

[별첨 7] 평가도구 1

리버미드 운동 평가(Rivermead motor assessment, RMA)

Rivermead Motor Assessment

General instructions: Go through the items in order of difficulty. Score '1' if patient can perform activity, ' 0' if he cannot. Three tries are allowed for each item. You may stop the 'Gross function' section and 'Arm' section after 3 consecutive '0' scores for 3 consecutive items.

In the 'Leg and Trunk' section all items should be tested, even if there are three consecutive '0' scores. Give no feed-back of whether correct or incorrect, just give general encouragement.

Repeat instructions and demonstrate them to the patient if necessary. All exercises to be carried out independently unless otherwise stated. All armtests refer to the affected side unless otherwise stated.

'Gross function' section can be assessed simply by asking, which makes it a rapid measure.

Section Item	Score
**A. Gross function**	
**1. Sit unsupported**<br>*Without holding on, on edge of bed, feet unsupported.*	
**2. Lying to sitting on side of bed**<br>*Using any method.*	
**3. Sitting to standing**<br>*May use hands to push up. Must stand up in 15 sec and stand for 15 sec, with an aid if necessary*	
**4. Transfer from weelchair to chair towards unaffected side**<br>*May use hands.*	
**5. Transfer from weelchair to chair towards affected side**<br>*May use hands.*	
**6. Walk 10 m indoors with an aid**<br>*Any walking aid. No stand-by help.*	
**7. Climb stairs independently**<br>*Any method. May use bannister and aid--must be a full flight of stairs.*	
**8. Walk 10 m indoors without an aid**<br>*No stand-by help. No caliper, splint or walking aid.*	
**9. Walk 10m , pick up bean bag from floor, turn and carry back**<br>*Bend down any way, may use aid to walk if necessary. No stand-by help. May use either hand to pick up bean bag.*	
**10. Walk outside 40 m**<br>*May use walking aid, caliper or splint. No stand-by help.*	
**11.Walk up and down four steps**<br>*Patient may use an aid if he would normally use one, but may not hold on to rail. This is included to test ability to negotiate curb or stairs without a rail.*	
**12. Run 10 m**<br>*Must be symmetrical.*	
**13. Hop on affected leg five times on the spot**<br>*Must hop on ball of foot without stopping to regain balance. No help with arms.*	
**Gross function Total**	

Section Item	Score
**B. Leg and trunk**	
**1. Roll to affected side**<br>*Starting position schould be lying, not crook lying.*	
**2. Roll to unaffected side**<br>*Starting position should be lying, not crook lying.*	
**3. Half-bridging**<br>*Starting position -- half-crook lying. Patient must put some weight through affected leg to lift hip on affected side. Therapist may position leg, but patient must maintain position even after movement is completed.*	
**4. Sitting to standing**<br>*May not use arms-- feet must be flat on floor--must put weight through both feet.*	
**5. Half-crook lying: lift affected leg over side of bed and return it to the same position.**<br>*Affected leg in half-crook position. Lift leg off bed on to support; for example, box, stool, floor, so that hip is in neutral and knee at 90 degrees while resting on support. Must keep affected knee flexed throughout movement. Do not allow external rotation at hip. This tests control of hip and knee.*	
**6. Standing, step unaffected leg on and off block**<br>*Without retraction of pelvis or hyperextension of knee. This tests knee and hip control while weight bearing through the affected leg.*	
**7. Standing, tap ground lightly five times with unaffected foot**<br>*Without retraction of pelvis or hyperextension of knee. Weight must stay on leg. This again tests knee and hip control while weight bearing through the affected leg but is more difficult than in 6.*	
**8. Lying, dorsiflex affected ankle with leg flexed**<br>*Physiotherapist may hold affected leg in position, knee at 90 degrees. Do not allow inversion. Must have half range of movement of unaffected foot.*	
**9. Lying, dorsiflex affected ankle with leg extended**<br>*Same conditios as in 8, with leg extended. Do not allow inversion or knee flexion. Foot must reach plantigrade (90°).*	
**10. Stand with affected hip in neutral position, flex affected knee**<br>*Therapist may not position leg. This is extremely difficult for most hemiplegic patients, but is included to assess minimal dysfunction.*	
**Leg and trunk function total**	

Section Item	Score
**C. Arm**	
**1. Lying, protract schoulder girdle with arm in elevation**<br>*Arm may be supported.*	
**2. Lying, hold extended arm in elevation (some external rotation) for at least 2 sec**<br>*Therapist should place arm in position and patient must maintain position with some external rotation. Do not allow pronation. Elbow must be held within 30 degrees of full extension.*	
**3. Flexion and extension of elbow, with arm as in 2 above**<br>*Elbow must extend to at least 20 degrees full extension. Palm should not face out during any part of movement.*	
**4. Sitting, elbow into side, pronation and supination**<br>*Three-quarters range is acceptable, with elbow unsupported and at right angles.*	
**5. Reach forward, pick up large ball with both hands and place down again**<br>*Ball should be on table so far in front of patient that he has to extend arms fully to reach it. Shoulders must be protracted, elbows extended, wrist neutral or extended, and fingers extended throughout movement. Palms should be kept in contact with the ball.*	
**6. Stretch arm forward, pick up tennis ball from table, release on affected side, return to table, then release again on table. Repeat five times**<br>*Shoulder must be protracted, elbow extended and wrist neutral or extended during each phase.*	
**7. Same exercise as in 6 above with pencil**<br>*Patients must use thumb and fingers to grip.*	
**8. Pick up a piece of paper from table in front and release five times**<br>*Patient must use thumb and fingers to pick up paper and not to pull it to edge of table. Arm position as in 6 above.*	
**9. Cut putty with a knife and fork on plate with non-slip mat and put pieces into container at side of plate**<br>*Bite-size pieces.*	
**10. Stand on spot, maintain upright position, pat large ball on floor with palm of hand for 5 continuous bounces**	
**11. Continuous opposition of thumb and each finger more than 14 times in 10 sec**<br>*Must do movement in consistent sequence. Do not allow thumb to slide from one finger to the other.*	

12. Supination and pronation on to palm of unaffected hand 20 times in 10 sec
Arm must be away from body, the palm and dorsum of hand must touch palm of good hand. Each tap counts as one. This is similar to 4 above, but introduces speed.

13. Standing, with affected arm abducted to 90 degrees with palm flat against wall. Maintain arm in position. Turn body towards wall and as far as possible towards arm, i.e. rotate body beyond 90 degrees
Do not allow flexion at elbow, and wrist must be extended with palm of hand fully in contact with wall.

14. Place string around head and tie bow at back
Do not allow neck to flex. Affected hand must be used for more than just supporting string. This tests function of hand without help of sight.

15. 'Pat- a-cake' seven times in 15 sec
Mark crosses on wall at schoulder level. Clap both hands together (both hands touch crosses.) Each sentence counts as one. Give patients three tries. This is a complex pattern which involves co-ordination, speed,and memory, as well as good arm function.

Arm function total

[별첨 7] 평가도구 2

도수근력검사(Manual muscle test, MMT)

MANUAL MUSCLE TESTING (MMT)

Manual Muscle Testing

16 muscle groups/ motions will be tested (not individual muscles). 14 of these are tested bilaterally. Grading will be based on the isometric **"Break"** test. It is essential that a grade of 3 be established before proceeding to application of resistance for grades above 3 or to alternate gravity minimized tests for grades below 3.

Muscle Group	Abbreviation
Shoulder abductors	SA
Elbow flexors	EF
Wrist extensors	WE
Wrist flexors	WF
Common finger extensors	CFE
Thumb flexors	TF
Hip flexors	HF
Knee extensors	KE
Ankle dorsiflexors	AD
Hip abductors	HAB
Hip adductors	HAD
Elbow extensors	EE
Shoulder external rotators	SER
Hip extensors	HE
Knee flexors	KF
Ankle plantar flexors	AP
Neck flexion	NF
Neck extension	NE

The criteria for assigning specific grades are as follows:

Grade	Criteria
5	Normal strength
5-	Uncertain muscle weakness
4+	Ability to move through full range of motion and hold against strong pressure
4	Ability to move through full range of motion and hold against moderate pressure
4-	Ability to move through full range of motion and hold against slight pressure; or breaks abruptly with pressure
3	Ability to move through full range of motion against gravity
3-	Ability to move through partial range of motion against gravity
2	Ability to move through any range of motion only with gravity eliminated
1	A flicker of movement is seen or felt in the muscle
0	No contraction palpable

For each test:

- Explain or demonstrate the movement that is required of the patient.
- Ask the patient to perform the movement through the full range against gravity.
- If the patient does not perform or is unable to perform the complete movement, check for these limiting factors:
    - Understanding of the required task
    - Availability of the appropriate range of motion - limitations may be due to soft tissue.
    - Weakness
- Based on the limiting factor(s):
    - instruct and demonstrate again
    - decide if joint restriction is affecting the performance
    - repeat the test using the alternative gravity eliminated position
- If the patient completes the movement through full available range against gravity, place the joint at the appropriate angle and apply resistance gradually. Appropriate feedback and encouragement will generate best effort on the part of the patient.
- Grade according to criteria described.

If the patient experiences pain, cramps, or spasms during the testing, the clinical evaluator must determine whether the patient feels that he/she is able to give his best effort in spite of his discomfort. If yes, continue the testing but note under comments that the patient complained of pain. If the patient feels that discomfort prohibits him from giving a maximal effort, do not test that muscle and code appropriately.

The precise order of testing, in terms of whether the evaluator should test R/L, or complete one side in each position is left to the discretion of the CE. However, it is important that the evaluator always be on the same side as the muscle being tested.

NOTE: It is necessary that the testing room allows for space all around the table and that the table is placed away from a wall.

Certain muscles can be graded from 0-5 in one position only. For other muscles, the first testing position will indicate a grading range of 3- to 5. If the muscle cannot be graded in this range, then it will need to be tested again in an alternate position.

A. POSITION I: SITTING

Seat patient comfortably on the examination table with shoes off and feet unsupported. The edge of the examining table should support the patient's thigh to a level proximal to the knee joint.

The following tests are performed in the sitting position:

Shoulder Abduction (3- thru 5)
1. Ask patient to abduct the shoulder as much as they are able to with the elbow extended. If patient able to abduct greater than 90 degrees, ask the patient to position the shoulder to 90° with the elbow flexed and the forearm pronated.

2. Provide stabilization proximal to the shoulder or on the opposite shoulder to prevent any tendency to lean in the opposite direction.

3. With shoulder in 90° of abduction, apply resistance at the elbow.

4. If AROM against gravity is present, but less than 90 degrees, score 3-/5.

5. If patient is unable to abduct the arm at all, retest in the supine position.

Elbow Flexion (0 thru 5)
1. Ask patient to bend the elbow and touch the shoulder with the forearm supinated.

(Observe for substitution, specifically rotation of forearm to mid position - brachioradialis).

2. Provide stabilization under elbow or anterior to the shoulder at proximal end of the humerus.

3. Position elbow at 120° of flexion and apply resistance at wrist to straighten the elbow.

4. If patient is unable to bend the elbow against gravity, support the patient's upper arm in abduction and elbow in extension with forearm supinated. Ask patient to bend the elbow – bringing hand to mouth with forearm in supination.

Wrist Extension (0 thru 5)
1. Place patient's arm by his side, elbow flexed to 90° forearm pronated.

2. Ask patient to extend his wrist from flexed position keeping fingers relaxed.

3. Support forearm proximal to the wrist to maintain 90°. Apply resistance on dorsum of hand.

4. If patient cannot extend the wrist against gravity, place forearm in neutral position, provide support proximal to the wrist and ask patient to extend wrist from flexed position.

Wrist Flexion (0 thru 5)
1. Position patient's arm by his side, elbow flexed to 90°, forearm supinated.

2. Ask patient to flex wrist with fingers relaxed.

3. Support forearm proximal to wrist to maintain 90° and give resistance on the palm of the hand.

4. If patient cannot flex the wrist against gravity, place forearm in neutral position, provide support proximal to wrist and ask patient to flex wrist from full extension keeping fingers flexed.

Common Finger Extensors

1. Place patient's arm by his side, elbow flexed to 90° forearm pronated.
2. Ask patient to extend all digits with fingers adducted.
3. Support wrist
4. Apply resistance just distal to the PIP joints

Long Finger Flexors Thumb (Flexor Pollicis Longus**)**

1. Patient in sitting with elbow flexed and forearm supinated as much as possible.

2. Stabilize the metacarpal bone and proximal phalanx of the thumb in extension.
3. Ask the patient to flex the interphalangeal joint of the thumb. Using index finger
4. Apply pressure at the palmar surface of the distal phalanx of the thumb in the direction of extension.

Hip Flexion (3- thru 5)

1. Ask patient to sit up straight and support trunk with arms supporting trunk with no greater than 20 degree of trunk extension. 2. Ask patient to bring his knee towards his chest. Patient should be able to flex to 30°. During this test, the patient should be discouraged from leaning sideways and the thigh should remain in neutral rotation. Do not allow patient to maintain hip flexion by pressing the belly of the calf muscle on the edge of the exam table.

3. Provide stabilization or counter pressure against the shoulder.

4. Apply resistance on distal surface of the thigh in the direction of hip extension.

5. To achieve a grade of 3-, the patient should be able to flex the hip enough that the examiner can slide the hand clearly under the distal thigh. If the patient can lift the thigh, but cannot maintain neutral rotation, grade as 3-. If the patient cannot lift the thigh, test in the alternate position.

Knee Extension (3-thru 5)

1. Ask patient to sit up straight and support trunk with arms propped with no greater than 20 degree of trunk extension. 2. The examiner may put his hand or a rolled towel under the distal end of the thigh to cushion it.

3. Patient is then asked to extend the knee.

4. Place the knee in 20° of flexion from full extension to avoid mechanical locking of the joint.

5. Apply resistance just proximal to the ankle.

6. For a grade of 3- patient should be able to actively extend the knee from 90° of flexion without a swinging motion secondary to flexing the knee and creating momentum. If the patient cannot move his leg against gravity, do not grade in this position. Retest in the side-lying position.

7. For a grade of 3, the patient must extend the knee to full extension. If a 'lag' is noted, grade a 3-.

Ankle Dorsiflexion (3- thru 5)

1. Ask patient to bend the foot up from plantar flexed position. (It is helpful to watch posterior ankle joint for movement; ankle inversion or great toe extension may appear with attempts at substitution.

2. Stabilize leg proximal to ankle.

3. If AROM is full within available ROM, apply resistance on dorsum of the foot at maximum available ROM.

4. If patient is unable to dorsiflex the foot, retest in the side-lying position.

B. <u>POSITION 2: SIDE-LYING</u>

Hip Abduction (3- thru 5)

1. Ask patient to lie on his side, bottom leg slightly flexed to increase the base of support. Patient may hold on to table to provide more stability.

2. Stand behind the patient, place the top leg in extension and stabilize the pelvis with one hand to prevent forward or backward rotation.

3. Ask patient to lift leg as high as possible without bringing it forward or back or rotating it.

4. Apply resistance at knee.

5. If patient cannot lift leg against gravity, retest in supine position.

Hip Adduction (3- thru 5)

1. Ask patient to lie on his side.
2. Stand behind patient, support the top leg in abduction (cradled in arm), and ask patient to lift his bottom leg.
3. If patient is able to lift leg approximately 75% off of the table, apply resistance at the knee. If less than 75% of antigravity ROM noted, score 3-/5.
4. If patient cannot lift leg against gravity, retest in supine position.

POSITION 3: PRONE

Neck Extension (3- thru 5)
To ensure patient safety make sure patient does not have pain or stiffness related to neck movements. Use clinical judgement to determine whether to perform the test.

1. Patient lying prone with arms at their side. Examiner holds the shoulders stable.

2. Patient is asked to lift his head up as high as he can. Patient should be able to get his face perpendicular to the table.

3. Place one hand over the occiput. As a safety measure, keep the other hand below the patient's chin and apply pressure in the direction of neck flexion.

4. If the patient cannot lift his head against gravity, retest in the side-lying position.

* to differentiate between 3 and 3-, drop the table to assess full ROM.

Shoulder External Rotation

1. Patient is lying prone with head in neutral (if possible). Arm is placed in 90 degrees of shoulder abduction, elbow flexed, and forearm pronated.
2. Examiner stabilizes under the distal humerus.
3. Ask the patient to rotate his shoulder, assess for full ROM
4. If full ROM antigravity, apply resistance proximal to the wrist and in the direction of internal rotation.
5. If patient cannot externally rotate against gravity, retest in the sitting position.

Hip Extension (3- thru 5)

1. Check the available range of hip extension and demonstrate the required motion.

2. Ask patient to bend his knee to 90° and then extend the hip.

3. Stabilize the lower trunk with one hand.

4. Apply resistance on the thigh with the other hand.

5. If the patient has limited range of motion or is unable to lift the hip against gravity, or barely lifts it, retest with the patient in the side lying position and finally in standing position to minimize changes of position.

Knee Flexion (3- thru 5)

1. Place patient prone on the table with a pillow under his head and head turned to either side.

2. Ask patient to bend the knee to 90°. Observe ankle to make sure that ankle dorsiflexion is not used to initiate movement.

3. Then place the knee in 70° of flexion.

4. Stabilize the thigh proximal to the knee.

5. Apply resistance just proximal to ankle.

6. For a grade of 3- patient must be able to lift the foot and ankle off the table and not just plantar flex the foot. If the patient cannot lift the leg, retest in side lying position

Ankle Plantar Flexion (3- thru 4+)

1. Maintain Knee flexion at 90° passively.

2. Ask patient to bend foot towards the ceiling.

3. Stabilize the ankle by holding the leg around the malleoli.

4. With the other hand give downwards pressure on the foot.

5. In order to score a 5, patient must be able to perform a single limb heel raise through full ROM*. If unable to perform full ROM in standing, score 5-/5.

*this test may be used for grade 5 if patient is unable to perform single limb stand secondary to proximal weakness.

Alternate tests

Hip Flexion (2 thru 0)
Support the patient's leg under the thigh and lower leg (with knee flexed) and place hip in extension and knee in flexion.
Ask patient to bring his knee towards the chest- flexing the hip.

Hip Extension (2 thru 0)
Support the patient's leg under the thigh and lower leg (with knee flexed) and place hip in flexion.
Ask patient to push the leg back - extending the hip.

Knee Flexion (2 thru 0)
Support the patient's leg under the thigh and lower leg and place knee in extension.

Ask patient to bend/flex the knee.

Knee Extension (2 thru 0)
Support the patient's leg under the thigh and lower leg and place knee in flexion.
Ask patient to straighten/extend the knee.

Ankle Dorsiflexion (2 thru 0)
Support the patient's leg proximal to the ankle and under the foot.
Ask patient to bend the foot towards his face.

Ankle Plantar Flexion (2 thru 0)
Support the patient's leg proximal to the ankle and under the foot.
Ask patient to move the foot down and back towards you.

D. POSITION 4: SUPINE

Neck Flexion (3- thru 5)

1. Passively perform the motion, thus checking for range and any discomfort

2. Ask patient to flex the head and neck until the chin touches the chest.

3. Apply pressure with one hand on the patient's forehead. For safety, hold the other hand just under the patient's head.

4. If unsure between 3/5 and 3-/5 drop the end of the table and check anti-gravity ROM. Pt must be able to move through full anti-gravity ROM for 3/5 strength.

4. If the patient cannot flex the cervical spine so that the chin moves toward the sternum, do not grade. Re-test in the side lying position.

Elbow Extension (3- thru 5)

1. Support the patient's upper arm in 90° of shoulder flexion so that the elbow is pointing toward the ceiling. The forearm is in neutral position and lies across the patient's chest.

2. Ask patient to straighten/extend the elbow.

3. Place the elbow in 20° of flexion and apply resistance just proximal to the wrist.

4. If the patient is unable to move the forearm against gravity, re-test in the sitting position.

Alternate test

Hip Abduction (2 thru 0)
Support the patient's leg under the knee and ankle.
Ask patient to abduct/ move the leg sideways towards you.

Hip Adduction (2 thru 0)
Support the patient's leg under the knee and ankle, bring it out into abduction.
Ask patient to abduct/ move the leg sideways towards midline.

NOTE: Do not allow external rotation, hip flexion, or knee flexion.

E. POSITION 5: REPEAT SIDE-LYING

Neck Flexion (2 thru 0)
Support the weight of the patient's head if necessary.
Ask patient to bend his neck forward bringing chin to chest.

Repeat alternate tests for hip flexion/ extension, knee flexion/extension and ankle dorsiflexion/ plantar flexion as necessary

F. POSITION 6: SITTING

Alternate test

Elbow Extension (2 thru 0)
Place patient's arm in 90° of shoulder flexion, elbow in full flexion and forearm in neutral position. Stabilize the arm proximal to elbow.
Ask patient to straighten the elbow.

Elbow Flexion (2 thru 0)
If patient is unable to bend the elbow against gravity, support the patient's upper arm in abduction and elbow in extension with forearm supinated. Ask patient to bend the elbow – bringing hand to mouth with forearm in supination.

Shoulder External Rotation
Patient is positioned with arm at his side with the elbow flexed to 90 degrees and forearm in neutral. The patient is then asked to rotate the shoulder externally. If the patient has week biceps, you may support the forearm.

G. POSITION 7: STANDING

Ankle Plantar flexion (5)

Have patient stand next to a table; single limb stand by bending one knee. Once the patient is steady (can steady themselves using table), ask them to go up onto their toes. Assess for complete plantar flexion of the foot.

Assign (5-) if unable to fully plantar flex.

Alternate tests

Hip Extension (3)

Ask patient to stand leaning over the table so that his entire trunk is supported and his feet are just touching the floor, let him hold on to the table for added stability.

Ask him to shift his weight onto one leg. Support the leg being tested in 90° of knee flexion, and ask the patient to lift the thigh away from the table. Grade as 3- if the patient lifts the thigh. If the patient cannot lift the thigh from the resting vertical position (with the examiner supporting the knee in flexion), go ahead and assign grade 2.

NOTE: To avoid having the patient change position more than necessary, assessing for a 3- grade can be done as the last muscle tested, as the patient gets on or off from the exam table.

[별첨 7] 평가도구 3

푸글마이어 척도-하지(Fugl Meyer assessment of lower extremity, FMS-LE)

Patient last name: .... Date of birth: ..../..../........
Patient first name: .... Date: ..../..../........

Fugl-Meyer Assessment Lower Extremity (FMA-LE)

Assessment of sensorimotor function:

LOWER EXTREMITY					
**I. Reflex activity, supine position**			None	Can be elicited	
Flexors: knee flexors			0	2	
Extensors: patellar, Achilles			0	2	
		Subtotal I		/4	
**II. Volitional movement within synergies**, supine position			None	Partial	Full
**Flexor synergy**: Maximal hip flexion (abduction/external rotation), maximal flexion in knee and ankle joint (palpate distal tendons to ensure active knee flexion).		Hip flexion	0	1	2
		Knee flexion	0	1	2
		Ankle dorsiflexion	0	1	2
**Extensor synergy**: From flexor synergy to the hip extension/adduction, knee extension and ankle plantar flexion. Resistance is applied to ensure active movement, evaluate both movement and strength		Hip extension	0	1	2
		Hip adduction	0	1	2
		Knee extension	0	1	2
		Ankle plantar flexion	0	1	2
		Subtotal II		/ 14	
**III. Volitional movement mixing synergies**, sitting position, knee 10 cm from the edge of the chair/bed			None	Partial	Full
**Knee flexion** from actively or passively extended knee	no active motion		0		
	no flexion beyond 90°, palpate tendons of hamstrings			1	
	knee flexion beyond 90°, palpate tendons of hamstrings				2
**Ankle dorsiflexion** compare with unaffected side	no active motion		0		
	limited dorsiflexion			1	
	complete dorsiflexion				2
		Subtotal III		/ 4	
**IV. Volitional movement with little or no synergy**, standing position, hip at 0°			None	Partial	Full
**Knee flexion to 90°** hip at 0°, balance support is allowed	no active motion/ immediate and simultaneous hip flexion		0		
	less than 90° knee flexion or hip flexion during movement			1	
	at least 90° knee flexion without simultaneous hip flexion				2
**Ankle dorsiflexion** compare with unaffected side	no active motion		0		
	limited dorsiflexion			1	
	complete dorsiflexion				2
		Subtotal IV		/ 4	
**V. Normal reflex activity** supine position, evaluated only if full score of 4 points achieved on earlier part IV, compare with unaffected side			None	Partial	Full
**Reflex activity** knee flexors, Achilles, patellar	0 points on part IV or 2 of 3 reflexes markedly hyperactive		0		
	1 reflex markedly hyperactive or at least 2 reflexes lively			1	
	maximum of 1 reflex lively, none hyperactive				2
		Subtotal V		/ 2	
		Total		/ 28	

[별첨 7] 평가도구 4

버그균형척도(Berg balance scale, BBS)

BERG BALANCE TESTS AND RATING SCALE

Patient Name ____
Date ____
Location ____
Rater ____

ITEM DESCRIPTION SCORE (0-4) Sitting to standing _____ Standing unsupported _____ Sitting unsupported _____ Standing to sitting _____ Transfers _____ Standing with eyes closed _____ Standing with feet together _____ Reaching forward with outstretched arm _____ Retrieving object from floor _____ Turning to look behind _____ Turning 360 degrees _____ Placing alternate foot on stool _____ Standing with one foot in front _____ Standing on one foot _____ TOTAL _____

GENERAL INSTRUCTIONS

Please demonstrate each task and/or give instructions as written. When scoring, please record the lowest response category that applies for each item.

In most items, the subject is asked to maintain a given position for a specific time. Progressively more points are deducted if the time or distance requirements are not met, if the subject's performance warrants supervision, or if the subject touches an external support or receives assistance from the examiner. Subjects should understand that they must maintain their balance while attempting the tasks. The choices of which leg to stand on or how far to reach are left to the subject. Poor judgment will adversely influence the performance and the scoring.

Equipment required for testing are a stopwatch or watch with a second hand, and a ruler or other indicator of 2, 5 and 10 inches (5, 12 and 25 cm). Chairs used during testing should be of reasonable height. Either a step or a stool (of average step height) may be used for item #12.

1. SITTING TO STANDING

INSTRUCTIONS: Please stand up. Try not to use your hands for support.
( ) 4 able to stand without using hands and stabilize independently
( ) 3 able to stand independently using hands
( ) 2 able to stand using hands after several tries
( ) 1 needs minimal aid to stand or to stabilize
( ) 0 needs moderate or maximal assist to stand

2. STANDING UNSUPPORTED

INSTRUCTIONS: Please stand for two minutes without holding.
( ) 4 able to stand safely 2 minutes
( ) 3 able to stand 2 minutes with supervision
( ) 2 able to stand 30 seconds unsupported
( ) 1 needs several tries to stand 30 seconds unsupported
( ) 0 unable to stand 30 seconds unassisted

If a subject is able to stand 2 minutes unsupported, score full points for sitting unsupported. Proceed to item #4.

3. SITTING WITH BACK UNSUPPORTED BUT FEET SUPPORTED ON FLOOR OR ON A STOOL

INSTRUCTIONS: Please sit with arms folded for 2 minutes.

( ) 4 able to sit safely and securely 2 minutes
( ) 3 able to sit 2 minutes under supervision
( ) 2 able to sit 30 seconds
( ) 1 able to sit 10 seconds
( ) 0 unable to sit without support 10 seconds

4. STANDING TO SITTING

INSTRUCTIONS: Please sit down.

( ) 4 sits safely with minimal use of hands
( ) 3 controls descent by using hands
( ) 2 uses back of legs against chair to control descent
( ) 1 sits independently but has uncontrolled descent
( ) 0 needs assistance to sit

5. TRANSFERS

INSTRUCTIONS: Arrange chairs(s) for a pivot transfer. Ask subject to transfer one way toward a seat with armrests and one way toward a seat without armrests. You may use two chairs (one with and one without armrests) or a bed and a chair.

( ) 4 able to transfer safely with minor use of hands
( ) 3 able to transfer safely definite need of hands
( ) 2 able to transfer with verbal cueing and/or supervision
( ) 1 needs one person to assist
( ) 0 needs two people to assist or supervise to be safe

6. STANDING UNSUPPORTED WITH EYES CLOSED

INSTRUCTIONS: Please close your eyes and stand still for 10 seconds.

( ) 4 able to stand 10 seconds safely
( ) 3 able to stand 10 seconds with supervision
( ) 2 able to stand 3 seconds
( ) 1 unable to keep eyes closed 3 seconds but stays steady
( ) 0 needs help to keep from falling

7. STANDING UNSUPPORTED WITH FEET TOGETHER

INSTRUCTIONS: Place your feet together and stand without holding.

( ) 4 able to place feet together independently and stand 1 minute safely
( ) 3 able to place feet together independently and stand for 1 minute with supervision
( ) 2 able to place feet together independently but unable to hold for 30 seconds
( ) 1 needs help to attain position but able to stand 15 seconds with feet together
( ) 0 needs help to attain position and unable to hold for 15 seconds

8. REACHING FORWARD WITH OUTSTRETCHED ARM WHILE STANDING

INSTRUCTIONS: Lift arm to 90 degrees. Stretch out your fingers and reach forward as far as you can. (Examiner places a ruler at end of fingertips when arm is at 90 degrees. Fingers should not touch the ruler while reaching forward. The recorded measure is the distance forward that the finger reaches while the subject is in the most forward lean position. When possible, ask subject to use both arms when reaching to avoid rotation of the trunk.)

( ) 4 can reach forward confidently >25 cm (10 inches)
( ) 3 can reach forward >12 cm safely (5 inches)
( ) 2 can reach forward >5 cm safely (2 inches)
( ) 1 reaches forward but needs supervision
( ) 0 loses balance while trying/requires external support

9. PICK UP OBJECT FROM THE FLOOR FROM A STANDING POSITION

INSTRUCTIONS: Pick up the shoe/slipper which is placed in front of your feet.

( ) 4 able to pick up slipper safely and easily
( ) 3 able to pick up slipper but needs supervision
( ) 2 unable to pick up but reaches 2-5cm (1-2 inches) from slipper and keeps balance independently
( ) 1 unable to pick up and needs supervision while trying
( ) 0 unable to try/needs assist to keep from losing balance or falling

10. TURNING TO LOOK BEHIND OVER LEFT AND RIGHT SHOULDERS WHILE STANDING

INSTRUCTIONS: Turn to look directly behind you over toward left shoulder. Repeat to the right. Examiner may pick an object to look at directly behind the subject to encourage a better twist turn.

( ) 4 looks behind from both sides and weight shifts well
( ) 3 looks behind one side only other side shows less weight shift
( ) 2 turns sideways only but maintains balance
( ) 1 needs supervision when turning
( ) 0 needs assist to keep from losing balance or falling

11. TURN 360 DEGREES

INSTRUCTIONS: Turn completely around in a full circle. Pause. Then turn a full circle in the other direction.

( ) 4 able to turn 360 degrees safely in 4 seconds or less
( ) 3 able to turn 360 degrees safely one side only in 4 seconds or less
( ) 2 able to turn 360 degrees safely but slowly
( ) 1 needs close supervision or verbal cueing
( ) 0 needs assistance while turning

12. PLACING ALTERNATE FOOT ON STEP OR STOOL WHILE STANDING UNSUPPORTED

INSTRUCTIONS: Place each foot alternately on the step/stool. Continue until each foot has touched the step/stool four times.

( ) 4 able to stand independently and safely and complete 8 steps in 20 seconds
( ) 3 able to stand independently and complete 8 steps in >20 seconds
( ) 2 able to complete 4 steps without aid with supervision
( ) 1 able to complete >2 steps needs minimal assist
( ) 0 needs assistance to keep from falling/unable to try

13. STANDING UNSUPPORTED ONE FOOT IN FRONT

INSTRUCTIONS: (DEMONSTRATE TO SUBJECT) Place one foot directly in front of the other. If you feel that you cannot place your foot directly in front, try to step far enough ahead that the heel of your forward foot is ahead of the toes of the other foot. (To score 3 points, the length of the step should exceed the length of the other foot and the width of the stance should approximate the subject's normal stride width)

( ) 4 able to place foot tandem independently and hold 30 seconds
( ) 3 able to place foot ahead of other independently and hold 30 seconds
( ) 2 able to take small step independently and hold 30 seconds
( ) 1 needs help to step but can hold 15 seconds
( ) 0 loses balance while stepping or standing

14. STANDING ON ONE LEG

INSTRUCTIONS: Stand on one leg as long as you can without holding.

( ) 4 able to lift leg independently and hold >10 seconds
( ) 3 able to lift leg independently and hold 5-10 seconds
( ) 2 able to lift leg independently and hold = or >3 seconds
( ) 1 tries to lift leg unable to hold 3 seconds but remains standing independently
( ) 0 unable to try or needs assist to prevent fall

TOTAL SCORE (Maximum = 56: _______

***References**

Wood-Dauphinee S, Berg K, Bravo G, Williams JI: The Balance Scale: Responding to clinically meaningful changes. Canadian Journal of Rehabilitation, 10: 35-50,1997.

Berg K, Wood-Dauphinee S, Williams JI: The Balance Scale: Reliability assessment for elderly residents and patients with an acute stroke. Scand J Rehab Med, 27:27-36, 1995.

Berg K, Maki B, Williams JI, Holliday P, Wood-Dauphinee S: A comparison of clinical and laboratory measures of postural balance in an elderly population. Arch Phys Med Rehabil, 73: 1073-1083, 1992.

Berg K, Wood-Dauphinee S, Williams JI, Maki, B: Measuring balance in the elderly: Validation of an instrument. Can. J. Pub. Health, July/August supplement 2:S7-11, 1992.

Berg K, Wood-Dauphinee S, Williams JI, Gayton D: Measuring balance in the elderly: Preliminary development of an instrument. Physiotherapy Canada, 41:304-311, 1989.

기능적 보행 분류(Functional ambulation category, FAC)

Functional Ambulation Classification (FAC)

Level	Ambulation Description	Definition
1	Non-functional	- Unable to ambulate<br>- Ambulates only in parallel bars<br>- Requires supervision or physical assistance from > 1 person
2	Dependent, Level II	- Requires manual contact of one person during ambulation on level surfaces<br>- Manual contact is continuous and necessary to support body weight and/or to maintain balance or assist coordination
3	Dependent, Level I	- Requires manual contact of one person during ambulation on level surfaces<br>- Manual contact is continuous or intermittent light touch to assist balance or coordination
4	Dependent, Supervision	- Ambulation occurs on level surfaces without manual contact of another person<br>- Requires stand-by guarding of one person because of poor judgment, questionable cardiac status, or the need for verbal cuing to complete the task
5	Independent, Level Surfaces Only	- Ambulate is independent on level surfaces<br>- Requires supervision/physical assistance to negotiate stairs, inclines, or unlevel surfaces
6	Independent, Level and Non-Level Surfaces	- Ambulation is independent on unlevel and level surfaces, stairs, and inclines

Patient last name: ....   Date of birth: ..../..../........

Patient first name: ....   Date: ..../..../........

[별첨 7] 평가도구 6

수정 랭킨 척도(modified Rankin scale, mRS)

University of North Carolina Hospitals
Chapel Hill, NC 27514
Department of Neurology

MODIFIED RANKIN SCALE (MRS)
MIM # 721

Rater Name: ___________________________

Date: ___________________________

Score Description

0	No symptoms at all
1	No significant disability despite symptoms; able to carry out all usual duties and activities
2	Slight disability; unable to carry out all previous activities, but able to look after own affairs without assistance
3	Moderate disability; requiring some help, but able to walk without assistance
4	Moderately severe disability; unable to walk without assistance and unable to attend to own bodily needs without assistance
5	Severe disability; bedridden, incontinent and requiring constant nursing care and attention
6	Dead

TOTAL (0–6): _______

Rater Sign / pager:____

Provider Sign / MD# / pager:____

HDF 536 / 4/03

References

Rankin J. "Cerebral vascular accidents in patients over the age of 60." *Scott Med J* 1957;2:200-15
Bonita R, Beaglehole R. "Modification of Rankin Scale: Recovery of motor function after stroke." *Stroke* 1988 Dec;19(12):1497-1500
Van Swieten JC, Koudstaal PJ, Visser MC, Schouten HJ, van Gijn J. "Interobserver agreement for the assessment of handicap in stroke patients." *Stroke* 1988;19(5):604-7

수정 바델 지수(modified Barthel index, MBI)

Appendix 3

MODIFIED BARTHEL INDEX (SHAH VERSION) : SELF CARE ASSESSMENT

INDEX ITEM	SCORE	DESCRIPTION
CHAIR/BED TRANSFERS	0	Unable to participate in a transfer. Two attendants are required to transfer the patient with or without a mechanical device.
	3	Able to participate but maximum assistance of one other person is require in all aspects of the transfer.
	8	The transfer requires the assistance of one other person. Assistance may be required in any aspect of the transfer.
	12	The presence of another person is required either as a confidence measure, or to provide supervision for safety.
	15	The patient can safely approach the bed walking or in a wheelchair, lock brakes, lift footrests, or position walking aid, move safely to bed, lie down, come to a sitting position on the side of the bed, change the position of the wheelchair, transfer back into it safely and/or grasp aid and stand. The patient must be independent in all phases of this activity.
AMBULATION	0	Dependent in ambulation.
	3	Constant presence of one or more assistant is required during ambulation.
	8	Assistance is required with reaching aids and/or their manipulation. One person is required to offer assistance.
	12	The patient is independent in ambulation but unable to walk 50 metres without help, or supervision is needed for confidence or safety in hazardous situations.
	15	The patient must be able to wear braces if required, lock and unlock these braces assume standing position, sit down, and place the necessary aids into position for use. The patient must be able to crutches, canes, or a walkarette, and walk 50 metres without help or supervision.
AMBULATION/WHEELCHAIR	0	Dependent in wheelchair ambulation.
	1	Patient can propel self short distances on flat surface, but assistance is required for all other steps of wheelchair management.
*** (If unable to walk)**	3	Presence of one person is necessary and constant assistance is required to manipulate chair to table, bed, etc.
**Only use this item if the patient is rated "0" for Ambulation, and then only if the patient has been trained in wheelchair management.**	4	The patient can propel self for a reasonable duration over regularly encountered terrain. Minimal assistance may still be required in "tight corners" or to negotiate a kerb 100mm high.
	5	To propel wheelchair independently, the patient must be able to go around corners, turn around, manoeuvre the chair to a table, bed, toilet, etc. The patient must be able to push a chair at least 50 metres and negotiate a kerb.

INDEX ITEM	SCORE	DESCRIPTION
STAIR CLIMBING	0	The patient is unable to climb stairs.
	2	Assistance is required in all aspects of chair climbing, including assistance with walking aids.
	5	The patient is able to ascend/descend but is unable to carry walking aids and needs supervision and assistance.
	8	Generally no assistance is required. At times supervision is required for safety due to morning stiffness, shortness of breath, etc.
	10	The patient is able to go up and down a flight of stairs safely without help or supervision. The patient is able to use hand rails, cane or crutches when needed and is able to carry these devices as he/she ascends or descends.
TOILET TRANSFERS	0	Fully dependent in toileting.
	2	Assistance required in all aspects of toileting.
	5	Assistance may be required with management of clothing, transferring, or washing hands.
	8	Supervision may be required for safety with normal toilet. A commode may be used at night but assistance is required for emptying and cleaning.
	10	The patient is able to get on/off the toilet, fasten clothing and use toilet paper without help. If necessary, the patient may use a bed pan or commode or urinal at night, but must be able to empty it and clean it.
BOWEL CONTROL	0	The patient is bowel incontinent.
	2	The patient needs help to assume appropriate position, and with bowel movement facilitatory techniques.
	5	The patient can assume appropriate position, but cannot use facilitatory techniques or clean self without assistance and has frequent accidents. Assistance is required with incontinence aids such as pad, etc.
	8	The patient may require supervision with the use of suppository or enema and has occasional accidents.
	10	The patient can control bowels and has no accidents, can use suppository, or take an enema when necessary.
BLADDER CONTROL	0	The patient is dependent in bladder management, is incontinent, or has indwelling catheter.
	2	The patient is incontinent but is able to assist with the application of an internal or external device.
	5	The patient is generally dry by day, but not at night and needs some assistance with the devices.
	8	The patient is generally dry by day and night, but may have an occasional accident or need minimal assistance with internal or external devices.
	10	The patient is able to control bladder day and night, and/or is independent with internal or external devices.

INDEX ITEM	SCORE	DESCRIPTION
BATHING	0	Total dependence in bathing self.
	1	Assistance is required in all aspects of bathing, but patient is able to make some contribution.
	3	Assistance is required with either transfer to shower/bath or with washing or drying; including inability to complete a task because of condition or disease, etc.
	4	Supervision is required for safety in adjusting the water temperature, or in the transfer.
	5	The patient may use a bathtub, a shower, or take a complete sponge bath. The patient must be able to do all the steps of whichever method is employed without another person being present.
DRESSING	0	The patient is dependent in all aspects of dressing and is unable to participate in the activity.
	2	The patient is able to participate to some degree, but is dependent in all aspects of dressing.
	5	Assistance is needed in putting on, and/or removing any clothing.
	8	Only minimal assistance is required with fastening clothing such as buttons, zips, bra, shoes, etc.
	10	The patient is able to put on, remove, corset, braces, as prescribed.
PERSONAL HYGIENE ***(Grooming)***	0	The patient is unable to attend to personal hygiene and is dependent in all aspects.
	1	Assistance is required in all steps of personal hygiene, but patient able to make some contribution.
	3	Some assistance is required in one or more steps of personal hygiene.
	4	Patient is able to conduct his/her own personal hygiene but requires minimal assistance before and/or after the operation.
	5	The patient can wash his/her hands and face, comb hair, clean teeth and shave. A male patient may use any kind of razor but must insert the blade, or plug in the razor without help, as well as retrieve it from the drawer or cabinet. A female patient must apply her own make-up, if used, but need not braid or style her hair.
FEEDING	0	Dependent in all aspects and needs to be fed, nasogastric needs to be administered.
	2	Can manipulate an eating device, usually a spoon, but someone must provide active assistance during the meal.
	5	Able to feed self with supervision. Assistance is required with associated tasks such as putting milk/sugar into tea, salt, pepper, spreading butter, turning a plate or other "set up" activities.
	8	Independence in feeding with prepared tray, except may need meat cut, milk carton opened or jar lid etc. The presence of another person is not required.
	10	The patient can feed self from a tray or table when someone puts the food within reach. The patient must put on an assistive device if needed, cut food, and if desired use salt and pepper, spread butter, etc.

SCORE	INTERPRETATION
00 - 20	Total Dependence
21 - 60	Severe Dependence
61 - 90	Moderate Dependence
91 - 99	Slight Dependence
- 100	Independence

SCORE	PREDICTION
Less Than 40	Unlikely to go home<br>- Dependent in Mobility<br>- Dependent in Self Care
60	Pivotal score where patients move from dependency to assisted independence.
60 - 80	If living alone will probably need a number of community services to cope.
More Than 85	Likely to be discharged to community living<br>- Independent in transfers and able to walk or use wheelchair independently.

REFERENCES

1. Shah, S., Vanclay, F., & Cooper, B. (1989a). Improving the sensitivity of the Barthel Index for stroke rehabilitation. *Journal of Clinical Epidemiology, 42,* 703 - 709.

2. Shah, S., & Cooper, B. (1991). Documentation for measuring stroke rehabilitation outcomes. *Australian Medical Records Journal, 21, 88* - 95.

3. Shah, S., Cooper, B., & Maas, F. (1992). The Barthel Index and A D L evaluation in stroke rehabilitation in Australia, Japan, the U K and the U S A. *Australian Occupational Therapy Journal, 39,* 5 - 13.

4. Granger, V., Dewis, L., Peters, W., Sherwood, C., & Barrett, J. (1979). Stroke rehabilitation analysis of repeated Barthel Index measures. *Archives of Physical and Medical Rehabilitation, 60, 14* - 17.

5. Hasselkus, B., (1982). Barthel self-care index and geriatric home care patients. *Physical and Occupational Therapy in Geriatrics, 1,* 11 - 22.

6. Leonard, R., & McGovern, L. (1992). The Barthel Index in an acute geriatric setting. *American Journal of Occupational Therapy, 39, 41* - 43.

[별첨 7] 평가도구 8

Short-form Health Survery 36 version 2(SF-36v2)

BMT CTN Protocol #0102
Multiple Myeloma

Your Health and Well-Being
HEALTH STATUS SURVEY SF-36

Participant ID: ______________________ **Protocol** 0102

Center: ______________________ **Visit Number:** ______________________

This survey asks for your views about your health. This information will help keep track of how you feel and how well you are able to do your usual activities. ***Thank you for completing this survey!***

For each of the following questions, please mark an ☒ in the one box that best describes your answer.

1. In general, would you say your health is:

Excellent	Very good	Good	Fair	Poor
▼	▼	▼	▼	▼
☐ 1	☐ 2	☐ 3	☐ 4	☐ 5

2. Compared to one year ago, how would you rate your health in general now?

Much better now than one year ago	Somewhat better now than one year ago	About the same as one year ago	Somewhat worse now than one year ago	Much worse now than one year ago
▼	▼	▼	▼	▼
☐ 1	☐ 2	☐ 3	☐ 4	☐ 5

SF-36v2™ Health Survey © 1996, 2000 by QualityMetric Incorporated and Medical Outcomes Trust. All Rights Reserved.
SF-36® is a registered trademark of Medical Outcomes Trust.
(SF-36v2 Standard, US Version 2.0)

3. The following questions are about activities you might do during a typical day. Does your health now limit you in these activities? If so, how much?

	Yes, limited a lot	Yes, limited a little	No, not limited at all
a Vigorous activities, such as running, lifting heavy objects, participating in strenuous sports	☐ 1	☐ 2	☐ 3
b Moderate activities, such as moving a table, pushing a vacuum cleaner, bowling, or playing golf	☐ 1	☐ 2	☐ 3
c Lifting or carrying groceries	☐ 1	☐ 2	☐ 3
d Climbing several flights of stairs	☐ 1	☐ 2	☐ 3
e Climbing one flight of stairs	☐ 1	☐ 2	☐ 3
f Bending, kneeling, or stooping	☐ 1	☐ 2	☐ 3
g Walking more than a mile	☐ 1	☐ 2	☐ 3
h Walking several hundred yards	☐ 1	☐ 2	☐ 3
i Walking one hundred yards	☐ 1	☐ 2	☐ 3
j Bathing or dressing yourself	☐ 1	☐ 2	☐ 3

SF-36v2™ Health Survey © 1996, 2000 by QualityMetric Incorporated and Medical Outcomes Trust. All Rights Reserved.
SF-36® is a registered trademark of Medical Outcomes Trust.
(SF-36v2 Standard, US Version 2.0)

4. During the past 4 weeks, how much of the time have you had any of the following problems with your work or other regular daily activities as a result of your physical health?

	All of the time	Most of the time	Some of the time	A little of the time	None of the time
a Cut down on the amount of time you spent on work or other activities	☐ 1	☐ 2	☐ 3	☐ 4	☐ 5
b Accomplished less than you would like	☐ 1	☐ 2	☐ 3	☐ 4	☐ 5
c Were limited in the kind of work or other activities	☐ 1	☐ 2	☐ 3	☐ 4	☐ 5
d Had difficulty performing the work or other activities (for example, it took extra effort)	☐ 1	☐ 2	☐ 3	☐ 4	☐ 5

5. During the past 4 weeks, how much of the time have you had any of the following problems with your work or other regular daily activities as a result of any emotional problems (such as feeling depressed or anxious)?

	All of the time	Most of the time	Some of the time	A little of the time	None of the time
a Cut down on the amount of time you spent on work or other activities	☐ 1	☐ 2	☐ 3	☐ 4	☐ 5
b Accomplished less than you would like	☐ 1	☐ 2	☐ 3	☐ 4	☐ 5
c Did work or other activities less carefully than usual	☐ 1	☐ 2	☐ 3	☐ 4	☐ 5

SF-36v2™ Health Survey © 1996, 2000 by QualityMetric Incorporated and Medical Outcomes Trust. All Rights Reserved.
SF-36® is a registered trademark of Medical Outcomes Trust.
(SF-36v2 Standard, US Version 2.0)

6. During the <u>past 4 weeks</u>, to what extent has your <u>physical health or emotional problems</u> interfered with your normal social activities with family, friends, neighbors, or groups?

Not at all	Slightly	Moderately	Quite a bit	Extremely
☐ 1	☐ 2	☐ 3	☐ 4	☐ 5

7. How much <u>bodily</u> pain have you had during the <u>past 4 weeks</u>?

None	Very mild	Mild	Moderate	Severe	Very Severe
☐ 1	☐ 2	☐ 3	☐ 4	☐ 5	☐ 6

8. During the <u>past 4 weeks</u>, how much did <u>pain</u> interfere with your normal work (including both work outside the home and housework)?

Not at all	A little bit	Moderately	Quite a bit	Extremely
☐ 1	☐ 2	☐ 3	☐ 4	☐ 5

SF-36v2™ Health Survey © 1996, 2000 by QualityMetric Incorporated and Medical Outcomes Trust. All Rights Reserved.
SF-36® is a registered trademark of Medical Outcomes Trust.
(SF-36v2 Standard, US Version 2.0)

9. These questions are about how you feel and how things have been with you <u>during the past 4 weeks</u>. For each question, please give the one answer that comes closest to the way you have been feeling. How much of the time during the <u>past 4 weeks</u>...

	All of the time	Most of the time	Some of the time	A little of the time	None of the time
a Did you feel full of life?	☐ 1	☐ 2	☐ 3	☐ 4	☐ 5
b Have you been very nervous?	☐ 1	☐ 2	☐ 3	☐ 4	☐ 5
c Have you felt so down in the dumps that nothing could cheer you up?	☐ 1	☐ 2	☐ 3	☐ 4	☐ 5
d Have you felt calm and peaceful?	☐ 1	☐ 2	☐ 3	☐ 4	☐ 5
e Did you have a lot of energy?	☐ 1	☐ 2	☐ 3	☐ 4	☐ 5
f Have you felt downhearted and depressed?	☐ 1	☐ 2	☐ 3	☐ 4	☐ 5
g Did you feel worn out?	☐ 1	☐ 2	☐ 3	☐ 4	☐ 5
h Have you been happy?	☐ 1	☐ 2	☐ 3	☐ 4	☐ 5
i Did you feel tired?	☐ 1	☐ 2	☐ 3	☐ 4	☐ 5

10. During the <u>past 4 weeks</u>, how much of the time has your <u>physical health or emotional problems</u> interfered with your social activities (like visiting friends, relatives, etc.)?

All of the time	Most of the time	Some of the time	A little of the time	None of the time
☐ 1	☐ 2	☐ 3	☐ 4	☐ 5

SF-36v2™ Health Survey © 1996, 2000 by QualityMetric Incorporated and Medical Outcomes Trust. All Rights Reserved.
SF-36® is a registered trademark of Medical Outcomes Trust.
(SF-36v2 Standard, US Version 2.0)

11. How TRUE or FALSE is <u>each</u> of the following statements for you?

	Definitely true	Mostly true	Don't know	Mostly false	Definitely false
a I seem to get sick a little easier than other people	☐ 1	☐ 2	☐ 3	☐ 4	☐ 5
b I am as healthy as anybody I know	☐ 1	☐ 2	☐ 3	☐ 4	☐ 5
c I expect my health to get worse	☐ 1	☐ 2	☐ 3	☐ 4	☐ 5
d My health is excellent	☐ 1	☐ 2	☐ 3	☐ 4	☐ 5

THANK YOU FOR COMPLETING THESE QUESTIONS!

SF-36v2™ Health Survey © 1996, 2000 by QualityMetric Incorporated and Medical Outcomes Trust. All Rights Reserved.
SF-36® is a registered trademark of Medical Outcomes Trust.
(SF-36v2 Standard, US Version 2.0)

[별첨 7] 평가도구 9

피츠버그 재활 참여 척도(Pittsburgh Rehabilitation Participation Scale, PRPS)

PITTSBURGH REHABILITATION PARTICIPATION SCALE

Patient name: ________________________________

Admission date: ______________________________

Instructions to therapist: for each therapy session, please circle one of each of the following to assess the patient's participation (effort and motivation as perceived by you) in the therapy session. Please rate as follows: None: patient refused entire session, or did not participate in any exercises in session. (see Note below)

Poor: patient refused or did not participate in at least half of session.

Fair: patient participated in most or all of exercises*, but did not show maximal effort or finish most exercises*, or required much encouragement to finish exercises*.

Good: patient participated in all exercises* with good effort and finished most but not all exercises* and passively followed directions (rather than actively taking interest in exercises* and future therapy).

Very good: patient participated in all exercises* with maximal effort and finished all exercises, but passively followed directions (rather than actively taking interest in exercises* and future therapy).

Excellent: patient participated in all exercises* with maximal effort, finished all exercises*, and actively took interest in exercises* and/or future therapy sessions.

> Note: if patient was unable to attend therapy because of medical test, bed rest order, illness, or scheduling conflict, do not mark any score.
>
> Note: in cases of doubt, choose the lower rating, eg, "good" rather than "very good."

PARTICIPATION:

Session Number	Date	Therapist Initials		None	Poor	Fair	Good	Very good	Excellent
1				1	2	3	4	5	6
2				1	2	3	4	5	6
3				1	2	3	4	5	6
4				1	2	3	4	5	6
5				1	2	3	4	5	6
6				1	2	3	4	5	6
7				1	2	3	4	5	6
8				1	2	3	4	5	6
9				1	2	3	4	5	6
10				1	2	3	4	5	6

NOTE. Available as an electronic file from the corresponding author by request.

*This version is specifically for PT. For the OT form, "exercises" should be replaced by "activities."

간이정신상태검사(Mini-Mental State Examination, MMSE)

Mini-Mental State Examination (MMSE)

Patient's Name: ______________________________ Date: ____________

Instructions: ***Ask the questions in the order listed. Score one point for each correct response within each question or activity.***

Maximum Score	Patient's Score	Questions
5		"What is the year? Season? Date? Day of the week? Month?"
5		"Where are we now: State? County? Town/city? Hospital? Floor?"
3		The examiner names three unrelated objects clearly and slowly, then asks the patient to name all three of them. The patient's response is used for scoring. The examiner repeats them until patient learns all of them, if possible. Number of trials: ___________
5		"I would like you to count backward from 100 by sevens." (93, 86, 79, 72, 65, …) Stop after five answers.<br>Alternative: "Spell WORLD backwards." (D-L-R-O-W)
3		"Earlier I told you the names of three things. Can you tell me what those were?"
2		Show the patient two simple objects, such as a wristwatch and a pencil, and ask the patient to name them.
1		"Repeat the phrase: 'No ifs, ands, or buts.'"
3		"Take the paper in your right hand, fold it in half, and put it on the floor." (The examiner gives the patient a piece of blank paper.)
1		"Please read this and do what it says." (Written instruction is "Close your eyes.")
1		"Make up and write a sentence about anything." (This sentence must contain a noun and a verb.)
1		"Please copy this picture." (The examiner gives the patient a blank piece of paper and asks him/her to draw the symbol below. All 10 angles must be present and two must intersect.)<br>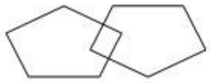
30		TOTAL

(Adapted from Rovner & Folstein, 1987)

Source: www.medicine.uiowa.edu/igec/tools/cognitive/MMSE.pdf

Provided by NHCQF, 0106-410

Instructions for administration and scoring of the MMSE

Orientation (10 points):
- Ask for the date. Then specifically ask for parts omitted (e.g., "Can you also tell me what season it is?"). One point for each correct answer.
- Ask in turn, "Can you tell me the name of this hospital (town, county, etc.)?" One point for each correct answer.

Registration (3 points):
- Say the names of three unrelated objects clearly and slowly, allowing approximately one second for each. After you have said all three, ask the patient to repeat them. The number of objects the patient names correctly upon the first repetition determines the score (0-3). If the patient does not repeat all three objects the first time, continue saying the names until the patient is able to repeat all three items, up to six trials. Record the number of trials it takes for the patient to learn the words. If the patient does not eventually learn all three, recall cannot be meaningfully tested.
- After completing this task, tell the patient, "Try to remember the words, as I will ask for them in a little while."

Attention and Calculation (5 points):
- Ask the patient to begin with 100 and count backward by sevens. Stop after five subtractions (93, 86, 79, 72, 65). Score the total number of correct answers.
- If the patient cannot or will not perform the subtraction task, ask the patient to spell the word "world" backwards. The score is the number of letters in correct order (e.g., dlrow=5, dlorw=3).

Recall (3 points):
- Ask the patient if he or she can recall the three words you previously asked him or her to remember. Score the total number of correct answers (0-3).

Language and Praxis (9 points):
- Naming: Show the patient a wrist watch and ask the patient what it is. Repeat with a pencil. Score one point for each correct naming (0-2).
- Repetition: Ask the patient to repeat the sentence after you ("No ifs, ands, or buts."). Allow only one trial. Score 0 or 1.
- 3-Stage Command: Give the patient a piece of blank paper and say, "Take this paper in your right hand, fold it in half, and put it on the floor." Score one point for each part of the command correctly executed.
- Reading: On a blank piece of paper print the sentence, "Close your eyes," in letters large enough for the patient to see clearly. Ask the patient to read the sentence and do what it says. Score one point only if the patient actually closes his or her eyes. This is not a test of memory, so you may prompt the patient to "do what it says" after the patient reads the sentence.
- Writing: Give the patient a blank piece of paper and ask him or her to write a sentence for you. Do not dictate a sentence; it should be written spontaneously. The sentence must contain a subject and a verb and make sense. Correct grammar and punctuation are not necessary.
- Copying: Show the patient the picture of two intersecting pentagons and ask the patient to copy the figure exactly as it is. All ten angles must be present and two must intersect to score one point. Ignore tremor and rotation.

(Folstein, Folstein & McHugh, 1975)

Interpretation of the MMSE

Method	Score	Interpretation
Single Cutoff	<24	Abnormal
Range	<21	Increased odds of dementia
	>25	Decreased odds of dementia
Education	21	Abnormal for 8th grade education
	<23	Abnormal for high school education
	<24	Abnormal for college education
Severity	24-30	No cognitive impairment
	18-23	Mild cognitive impairment
	0-17	Severe cognitive impairment

Sources:

- Crum RM, Anthony JC, Bassett SS, Folstein MF. Population-based norms for the mini-mental state examination by age and educational level. *JAMA*. 1993;269(18):2386-2391.
- Folstein MF, Folstein SE, McHugh PR. "Mini-mental state": a practical method for grading the cognitive state of patients for the clinician. *J Psychiatr Res*. 1975;12:189-198.
- Rovner BW, Folstein MF. Mini-mental state exam in clinical practice. *Hosp Pract*. 1987;22(1A):99, 103, 106, 110.
- Tombaugh TN, McIntyre NJ. The mini-mental state examination: a comprehensive review. *J Am Geriatr Soc*. 1992;40(9):922-935.

[별첨 7] 평가도구 11

불안-우울 척도(Hostpial Anxiety and Depression Scale, HADs)

Hospital Anxiety and Depression Scale (HADS)

Tick the box beside the reply that is closest to how you have been feeling in the past week.
Don't take too long over you replies: your immediate is best.

D	A		D	A	
		**I feel tense or 'wound up':**			**I feel as if I am slowed down:**
	3	Most of the time	3		Nearly all the time
	2	A lot of the time	2		Very often
	1	From time to time, occasionally	1		Sometimes
	0	Not at all	0		Not at all
		**I still enjoy the things I used to enjoy:**			**I get a sort of frightened feeling like 'butterflies' in the stomach:**
0		Definitely as much		0	Not at all
1		Not quite so much		1	Occasionally
2		Only a little		2	Quite Often
3		Hardly at all		3	Very Often
		**I get a sort of frightened feeling as if something awful is about to happen:**			**I have lost interest in my appearance:**
	3	Very definitely and quite badly	3		Definitely
	2	Yes, but not too badly	2		I don't take as much care as I should
	1	A little, but it doesn't worry me	1		I may not take quite as much care
	0	Not at all	0		I take just as much care as ever
		**I can laugh and see the funny side of things:**			**I feel restless as I have to be on the move:**
0		As much as I always could		3	Very much indeed
1		Not quite so much now		2	Quite a lot
2		Definitely not so much now		1	Not very much
3		Not at all		0	Not at all
		**Worrying thoughts go through my mind:**			**I look forward with enjoyment to things:**
	3	A great deal of the time	0		As much as I ever did
	2	A lot of the time	1		Rather less than I used to
	1	From time to time, but not too often	2		Definitely less than I used to
	0	Only occasionally	3		Hardly at all
		**I feel cheerful:**			**I get sudden feelings of panic:**
3		Not at all		3	Very often indeed
2		Not often		2	Quite often
1		Sometimes		1	Not very often
0		Most of the time		0	Not at all
		**I can sit at ease and feel relaxed:**			**I can enjoy a good book or radio or TV program:**
	0	Definitely	0		Often
	1	Usually	1		Sometimes
	2	Not Often	2		Not often
	3	Not at all	3		Very seldom

Please check you have answered all the questions

Scoring:
Total score: Depression (D) ___________ Anxiety (A) ______________
0-7 = Normal
8-10 = Borderline abnormal (borderline case)
11-21 = Abnormal (case)

[별첨 7] 평가도구 12

근육경직척도(Modified Ashworth scale, MAS)

Modified Ashworth Scale Instructions

General Information (derived Bohannon and Smith, 1987):

- Place the patient in a supine position
- If testing a muscle that primarily flexes a joint, place the joint in a maximally flexed position and move to a position of maximal extension over one second (count "one thousand one")
- If testing a muscle that primarily extends a joint, place the joint in a maximally extended position and move to a position of maximal flexion over one second (count "one thousand one")
- Score based on the classification below

Scoring (taken from Bohannon and Smith, 1987):

0 No increase in muscle tone

1 Slight increase in muscle tone, manifested by a catch and release or by minimal resistance at the end of the range of motion when the affected part(s) is moved in flexion or extension

1+ Slight increase in muscle tone, manifested by a catch, followed by minimal resistance throughout the remainder (less than half) of the ROM

2 More marked increase in muscle tone through most of the ROM, but affected part(s) easily moved

3 Considerable increase in muscle tone, passive movement difficult

4 Affected part(s) rigid in flexion or extension

Patient Instructions:

The patient should be instructed to relax.

Downloaded from www.rehabmeasures.org
Test instructions provided courtesy of Richard Bohannon PT, PhD and Melissa Smith, PT

Modified Ashworth Scale Testing Form

Name:_______________________________________ Date:__________

Muscle Tested	Score
________________	_____
________________	_____
________________	_____
________________	_____
________________	_____
________________	_____
________________	_____
________________	_____
________________	_____

Downloaded from www.rehabmeasures.org
Test instructions provided courtesy of Richard Bohannon PT, PhD and Melissa Smith, PT

Reference for test instructions:

Bohannon, R. and Smith, M. (1987). "Interrater reliability of a modified Ashworth scale of muscle spasticity." Physical Therapy 67(2): 206.

Downloaded from www.rehabmeasures.org
Test instructions provided courtesy of Richard Bohannon PT, PhD and Melissa Smith, PT

[별첨 7] 평가도구 13

브룬스트롬 기능회복 단계 (brunnstrom stages of motor recovery, BRS)

Stages	Hand	Arm	Leg
1	Flaccidity	Flaccidity	No motor function
2	Gross grasp; very little finger flexion possible	the basic synergies of the limbs manifest as weakly associated reactions	A bit voluntary movement; minimal increase in spasticity
3	Gross grasp; grasping objects in hook form without releasing	Maximum spasticity; the voluntary performing of basic limb synergies	Maximum spasticity; the flexion and extension of the hip, knee, and ankle joints' flexion and extension in sitting and standing position
4	The ability of lateral prehension (grasping delicate objects with the thumb and other fingers); a small degree of finger extension and limited thumb movements	Decrease in muscle spasticity; the ability to perform combination movements deviating from limb synergies	Ninety-degree flexion of the knee in sitting position, while the foot sliding backward on the ground; dorsiflexion of the foot with the heel on the ground & 90-degree flexion of the knee
5	Spherical and cylindrical grasp while releasing possible; palmar prehension	Synergies are not significant anymore; ability to perform more combinat ion movements deviating from limb synergies	Ankle dorsiflexion with the hip and knee extended; knee flexion with the hip extended in the standing position
6	All types of prehension; isolated finger movements in their full range of mot ion; voluntary flexion of the fingers at all angles	The disappearance of spasticity except while performing rapid movements; isolated joint movement easily possible	Hip abduction in sitting or standing position; bilateral hip internal and external rotation with ankle inversion and eversion in sitting position

[별첨 9] 생체현상 측정기기 의료기기 제조 및 품질관리 기준 적합인정서

인정번호(No.) : KTL-AABA-10962

의료기기 제조 및 품질관리 기준 적합인정서
(Certificate of GMP)

■ **업소명/허가번호 (Company name of Applicant / License No.)**

(주)에스엠디솔루션/제 6498 호
SMDsolutions Co., Ltd.

■ **업소 소재지 (Company address of Applicant)**

서울특별시 종로구 대학로 101 서울대학교 치의학대학원 생체재료동 107-1호(연건동)
107-1, SEOUL NATIONAL UNIVERSITY SCHOOL OF DENTISTRY 101, Daehak-ro, Jongno-gu, Seoul , Republic of Korea

■ **제조소명 (Name of Manufacturer)**

제 조 자 : (주)에스엠디솔루션(SMDsolutions Co., Ltd.)

■ **제조소 소재지 (Address of Manufacturer)**

제 조 자 : 서울특별시 종로구 대학로 101 서울대학교 치의학대학원 생체재료동 107-1호(연건동)
107-1, SEOUL NATIONAL UNIVERSITY SCHOOL OF DENTISTRY 101, Daehak-ro, Jongno-gu, Seoul , Republic of Korea

■ **품목군 (Category)**

생체현상 측정기기(Physiological Monitoring Device)

의료기기 제조 및 품질관리기준에 적합함을 인정합니다.
(We hereby certify that the above manufacturer complies with Korea Good Manufacturing Practices of Medical Devices for the product group listed above)

발행일자(Date of Issue) : 2021. 04. 14
유효기간(Date of Expiration) : 2024. 04. 13

한 국 산 업 기 술 시 험 원 장
Korea Testing Laboratory

[별첨 10] 소프트웨어 의료기기 제조 및 품질관리 기준 적합인정서

인정번호(No.) : KTL-ACAA-13361

의료기기 제조 및 품질관리 기준 적합인정서
(Certificate of GMP)

■ **업소명/허가번호 (Company name of Applicant / License No.)**

(주)에스엠디솔루션/제 6498 호
SMDsolutions Co., Ltd.

의료기기 제조 및 품질관리기준
GMP
MINISTRY OF FOOD AND DRUG SAFETY

■ **업소 소재지 (Company address of Applicant)**

서울특별시 종로구 대학로 101 서울대학교 치의학대학원 생체재료동 107-1호(연건동)
107-1, SEOUL NATIONAL UNIVERSITY SCHOOL OF DENTISTRY 101, Daehak-ro, Jongno-gu, Seoul , Republic of Korea

■ **제조소명 (Name of Manufacturer)**

제 조 자 : (주)에스엠디솔루션(SMDsolutions Co., Ltd.)

■ **제조소 소재지 (Address of Manufacturer)**

제 조 자 : 서울특별시 종로구 대학로 101 서울대학교 치의학대학원 생체재료동 107-1호(연건동)
107-1, SEOUL NATIONAL UNIVERSITY SCHOOL OF DENTISTRY 101, Daehak-ro, Jongno-gu, Seoul , Republic of Korea

■ **품목군 (Category)**

소프트웨어(Software as a Medical Device)

의료기기 제조 및 품질관리기준에 적합함을 인정합니다.
(We hereby certify that the above manufacturer complies with Korea Good Manufacturing Practices of Medical Devices for the product group listed above)

발행일자(Date of Issue) : 2022. 02. 07
유효기간(Date of Expiration) : 2024. 04. 13

ktl

한 국 산 업 기 술 시 험 원 장
Korea Testing Laboratory

[별첨 11] 연구비 내역서

연구비실행예산계획 (1단계-3차년도): 2022.01.01~2022.12.31

비목	세목	지급	액수	비율
인건비	내부인건비	지급	50640000	41.63%
	외부인건비	지급	20000000	16.44%
	소계		70640000	58.07%
연구재료비	-		0	0.00%
	소계		0	0.00%
연구시설장비비	-		0	0.00%
	소계		1000000	0.00%
연구활동비	외장하드		500000	0.41%
	노트북		1500000	1.23%
	데스크탑/모니터		2000000	1.64%
	벤치사용료		800000	0.66%
	영문교정료		1000000	0.82%
	논문 게재료		2000000	1.64%
	학회/세미나 참가비		777200	0.64%
	사용적합성테스트비		14000000	11.51%
	임상시험비		9000000	7.40%
	MRCC 의뢰비		1000000	0.82%
	소프트웨어 활용비		500000	0.41%
	회의비		2000000	1.64%
	국내여비		1000000	0.82%
	사무용품비		1000000	0.82%
	소계		37077200	30.48%
연구수당			1000000	0.82%
직접비 소계			108717200	89.38%
간접비			12922800	10.62%
연구비총액			121640000	100.00%

연구비실행예산계획 (2단계-1차년도): 2023.01.01~2023.12.31

비목	세목	지급	액수	비율
인건비	내부인건비	지급	32160000	17.65%
	외부인건비	지급	75000000	41.17%
	소계		107160000	58.83%
연구재료비	-		0	0.00%
	소계		0	0.00%
연구활동비	외장하드		600000	0.33%
	노트북		2500000	1.37%
	CPU 등 컴퓨터 부품		3500000	1.92%
	영상촬영장비		700000	0.38%
	센서		1000000	0.55%
	임상시험용 물품		500000	0.27%
	장비관리 및 유지비		400000	0.22%
	벤치사용료		2000000	1.10%
	영문교정료		1000000	0.55%
	논문 게재료		3000000	1.65%
	특허비용		2000000	1.10%
	도서구입비		1000000	0.55%
	인쇄,슬라이드제작, 복사비		500000	0.27%
	우편		100000	0.05%
	학회/세미나 참가비		4620000	2.54%
	소프트웨어 활용비		700000	0.38%
	회의비		3000000	1.65%
	국외여비		8100000	4.45%
	국내여비		600000	0.33%
	야근식대비		1000000	0.55%
	사무용품비		400000	0.22%
	피험자사례비		10000000	5.49%
	소계		37220000	20.43%
연구수당			6000000	3.29%
직접비 소계			160380000	88.04%
간접비			21780000	11.96%
연구비총액			182160000	100.00%

[별첨 12] 임상시험배상보험 가입 증권

KB손해보험

ORIGINAL

일반보험증권(영문)
No Fault Compensation Insurance (14144)

Policy Basic Information

Policy Number	2023-0299025 (000)	Date	2023년 01월 16일
Policy Period	2023년 02월 01일 00:00 부터 2026년 01월 31일 24:00 까지 (1096일간)		

Policyholder	이우형 (850909-1******)
Insured	이우형 (850909-1******)

Premium	WON 2,943,000

Details

■ Covered Business/Items

No(1) Effects of in-bed self-exercises based on EMG-sensor feedback on functional mobility in subacute stroke patients: a prospective, multicenter, single-blind, open-label, exploratory pilot clinical trial

■ Premium Basis

No(1) Persons 24 명

■ Location

No(1) 030-80 Daehak-ro, Jongno-gu, Seoul 101 , Korea.

■ Terms & Conditions

No(1) Human Clinical Trial

1) No Fault Compensation Policy
2) A.I.D.S. and HEPATITIS Exclusion Clause
3) Communicable Disease Exclusion Clause (LMA5399)
4) Cyber Incident Exclusion Clause (LMA5272)
5) Legal Liability Extension
6) Millennium Exclusion Clause
7) Sanction Limitation and Exclusion Clause
8) Terrorism Exclusion Clause
9) Costs & Expenses Endorsement
10) Extended Discovery Period Extension for 2months
11) Policy Territory : KOREA
12) Policy Jurisdiction : KOREA
13) Retroactive Date : 2023-02-01
14) Human Clinical Trial to Infants or younger people under 3 years : 아니오

■ L.O.L & Ded

No(1) Human Clinical Trial

Coverage	Limit	Cur	Limit of Liability			Deductible
			any one person	any one claim	aggregate	any one claim
No Fault Compensation Policy		WON	200,000,000	200,000,000	200,000,000	1,000,000

Premium Installments

No	Due Date	Portion	Installment Premium		No	Due Date	Portion	Installment Premium	
1	2023-02-01	100 %	WON	2,943,000					

Co-Insurance [Leader : KB]

KB												
100.00%												

고객콜센터 (상담및문의)	1544-0114	지점	법인영업4부 1지점 (☎02-6900-3549)	영업담당자	제이투케이 보험대리점(김진성) (☎010-6617-7753)

- 1/2 -

발 급 일2023년 01월 16일
21029182023011616560

KB손해보험

ORIGINAL

일반보험증권(영문)
No Fault Compensation Insurance (14144)

IN WITNESS WHERE OF KB Insurance Co., Ltd. SEOUL, KOREA has caused this policy to be signed by its President or authorized representative, and countersigned on the Declarations page by a duly authorized representative.

KB Insurance Co.,Ltd

Kim, Ki Hwan

Kim Ki Hwan
President & CEO

[기타사항]
이 보험가입증서(보험증권)는 계약체결의 증거로서 발행된 것으로 실제 계약 내용은 청약서 및 해당 상품의 보험약관에 따르며, 「보험업감독규정」 제4-35조의2·제7-45조, 「보험업감독업무시행세칙」 제5-11조 및 「금융소비자 보호에 관한 법률」 제19조, 동법 시행령 제13조·제14조 및 동법 감독규정 제12조·제13조에 의거하여 "KB손해보험"의 내부통제 기준에 따른 절차를 거쳐 제공되는 자료입니다.

고객콜센터 (상담및문의)	1544-0114	지점	법인영업4부 1지점 (☎02-6900-3549)	영업담당자	제이투케이 보험대리점(김진성) (☎010-6617-7753)

발 급 일2023년 01월 16일
210291820230116165607

[별첨 13] 식약처 임상시험계획승인서

열 람 용

제 1445 호

의료기기 임상시험계획승인서

신 청 인<br>(대표자)	성 명	김헌정	생년월일	1967년 09월 02일
	주 소			
제조(수입)<br>업 소	명칭(상호)	(주)에스엠디솔루션	업허가번호	제 6498 호
	소 재 지	서울특별시 종로구 대학로 101 서울대학교 치의학대학원 생체재료동 107-1호 (연건동)		
제 조 원<br>(수입 또는 제조공정 전부 위탁의 경우)	명칭(상호)		제 조 국	
	소 재 지			
임 상 시 험<br>개 요	명칭(제품명, 품목명, 모델명)	근전도분석소프트웨어	분류번호<br>(등급)	E10010.01(2)
	임상시험계획 승인번호	제 1445 호		
	임상시험의 제목	아급성기 뇌졸중 환자에서 근전도 센서 피드백에 기반한 침상내 자가운동의 기능적 이동성에 대한 효과 : 전향적, 다기관, 단일눈가림, 개방형, 탐색적 예비임상시험		

「의료기기법」 제 10조 및 같은 법 시행규칙 제20조제4항에 따라 위와 같이 임상시험계획을 승인합니다.

※ 붙임 : 의료기기 임상시험계획승인신청서 1부.

2023 년 01 월 06 일

식 품 의 약 품 안 전 처 장 (인)

열 람 용

변경 및 처분 사항 등	
년 월 일	내 용
2023-01-06	최초승인

모델명(형명)	
일련번호	형 명
1	Myoverse s/w

포장단위	
일련번호	포장단위
1	ea

'본 문서는 열람용으로서, 무단 복제와 도용을 금지함'

[별첨 14] 근전도계 의료기기 제조 인증서

열 람 용

제인 22-4998 호

의료기기 제조 인증서

(업 허가번호 : 제 6498 호)

구 분	[✔] 제조 / [ ] 수입	[✔] 품목 / [ ] 품목류	
명 칭 (제품명, 품목명, 모델명)	Myoverse, 근전도계, [illegible]	분류번호(등급)	A30010.01 (2)
모 양 및 구 조	별첨		
원 재 료	별첨		
제 조 방 법	별첨		
성 능	별첨		
사 용 목 적	별첨		
사 용 방 법	별첨		
사용 시 주의사항	별첨		
포 장 단 위	별첨		
저장방법 및 사용기간	저장방법 : 별첨, 사용기간 : 별첨		
시 험 규 격	제 [illegible] (2022.12.06) 한국기계전기전자시험연구원		
제조(수입)업자 정부	제조(수입)업자 : (주)[illegible], 서울특별시 종로구 대학로 101 서울대학교 의과대학 [illegible] 107-1호(연건동)<br>제조원 : 상동		
인 증 조 건	없음		
유 효 기 간	2022.12.15 ~ 2027.12.14		
소 재 지	서울특별시 종로구 대학로 101 서울대학교 의과대학 [illegible] 107-1호(연건동)		
비 고			

「의료기기법」 제6조·제15조 및 같은 법 시행 규칙 제6조제2항·제34조에 따라 위와 같이 인증합니다.

2022 년 12 월 15 일

한국의료기기안전정보원장 (인)

열 람 용

변경 및 처분 사항 등	
년 월 일	내 용
2022-12-15	최초허가

제 품 명
Myoverse

모 델 명	
일련번호	모 델 명
1	HSN2[illegible]

포장단위	
일련번호	포장단위
1	자사 포장단위

제조원	
일련번호	제조의뢰자
1	
일련번호	제조자

본 문서는 열람용으로써, 무단 복제와 도용을 금지함

[별첨 15] 변경된 근전도계 의료기기 제조 인증서

열 람 용

제인 22-4998 호

의료기기 제조 인증서

(업 허가번호 : 제 6498 호)

구 분	[✔] 제조 / [ ] 수입	[✔] 품목 / [ ] 품목류	
명 칭<br>(제품명, 품목명, 모델명)	Myoverse,근전도계,HSN2ERE	분류번호(등급)	A30010.01(2)
모 양 및 구 조	별첨		
원 재 료	별첨		
제 조 방 법	별첨		
성 능	별첨		
사 용 목 적	별첨		
사 용 방 법	별첨		
사 용 시 주 의 사 항	별첨		
포 장 단 위	별첨		
저장방법 및 사용기간	저장방법 : 별첨, 사용기간 : 별첨		
시 험 규 격	제 의기변 10-03-20230147-0189호(2023.4.20.),<br>한국기계전기전자시험연구원		
제조(수입)업자 정보	제조(수입)업자 : (주)에스엠디솔루션, 서울특별시 종로구 대학로 101 서울대학교 치의학대학원 생체재료동 107-1호(연건동)<br>제조원 : 상동		
인 증 조 건	없음		
유 효 기 간	2022.12.15 ~ 2027.12.14		
소 재 지	서울특별시 종로구 대학로 101 서울대학교 치의학대학원 생체재료동 107-1호(연건동)		
비 고			

「의료기기법」제6조·제15조 및 같은 법 시행 규칙 제6조제2항·제34조에 따라 위와 같이 인증합니다.

2022 년 12 월 15일

한국의료기기안전정보원장 (인)

열 람 용

변경 및 처분 사항 등	
년 월 일	내 용
2022-12-15	최초허가
2023-05-02	사용시 주의사항(적용상의 주의 삭제)
2023-05-02	사용방법(사용 전 준비사항, 사용 후 보관 및 관리방법의 USB Cable 삭제, 사용방법 중 본체와 패치 연결, LED 표시 및 충전 사용 변경, 앱 사용방법의 EMG Filter 주파수 범위 변경)
2023-05-02	원재료(제어부의 Main PCB 변경, 전원부의 USB Cable 삭제, 장착부(패치)의 전극 수량 변경, 연결부 추가)
2023-05-02	모양및구조-작용원리(USB Cable 삭제)
2023-05-02	모양및구조-특성(작동계통도 변경, 작동계통도 설명(본체 및 패치: 표시부, 센서부 변경, 본체 및 패치: 결합부 추가, 본체(패치) 및 모바일 앱: 패치 및 본체 변경), 전기회로도 변경(전원부(Power on circuit), 통신부(Antenna interface)))
2023-05-02	성능(Sampling Rate의 '최대'삭제, 독립전원 구동시간 변경(20시간 유지->24시간 유지), LED 표시상태의 전원 ON/OFF 모드 추가, Input Referred Noise Level의 단위 변경, Dynamic range의 'TYP'삭제, Input impedance 변경(3MΩ 이상->5M(Ω) 이상), EMG Filter(Hz) 변경(20Hz~240Hz->20Hz~450Hz), Sensitivity의 '최소 값'추가)
2023-05-02	모양및구조-치수(본체 및 패치 변경, USB Cable 삭제)
2023-05-02	모양및구조-외형(외관사진 본체 및 패치 변경, USB Cable 삭제, 외관설명 본체 설명 변경(본체, 본체연결부, USB Connector, 패치연결부, LED표시등), 패치 설명 변경(패치, 패치커버), 어플리케이션 화면 설명(신호보기: EMG Filter 주파수 범위 변경))
2023-05-02	시험규격(시험기준 및 시험방법 변경, 독립전원 구동 시간 변경, LED 표시상태 추가, Input impedance 변경, EMG Filter(Hz) 변경)

제품명
Myoverse

모 델 명		
일련번호	모델명	수출용여부
1	HSN2ERE	아니오

열 람 용

포장단위	
일련번호	포장단위
1	자사 포장단위

제조원	
일련번호	제조의뢰자
일련번호	제조자

'본 문서는 열람용으로써, 무단 복제와 도용을 금지함

[별첨 16] 한국의료기기안전정보원 변경인증

"의료기기 산업 글로벌 경쟁력 강화, 한국의료기기안전정보원이 만들어가겠습니다."

NIDS 한국의료기기안전정보원

한국의료기기안전정보원

수신 (주)에스엠디솔루션, 대표:김현정 귀하 (우 03080 서울특별시 종로구 대학로 101 서울대학교 치의학대학원 생체재료동 107-1호(연건동))
(경유)
제목 의료기기 제조 인증사항 변경인증[(주)에스엠디솔루션]

1. 귀 사에서 2023.04.25자로 우리 원에 신청(접수번호 제20230066802호)하신 의료기기 제조인증변경 - (주)에스엠디솔루션·근전도계(HSN2ERE)와 관련됩니다.

2. 위 호와 관련하여 「의료기기법」 제12조, 같은 법 시행규칙 제26조제3항 및 '의료기기 허가·신고·심사 등에 관한 규정' 에 따라 제출된 변경 인증신청서 및 첨부자료를 검토한 결과,

3. 그 신청내용 및 첨부자료가 「의료기기법」 및 인증 관련규정에 적합하므로, 「의료기기법」 제12조 및 같은 법 시행규칙 제26조제3항에 따라 아래와 같이 변경인증 합니다.

제조업 허가번호	품목별 제조 인증번호	변경 인증일자	품목명 (분류번호 및 등급)	변경내용 구분		변경 전	변경 후
제6498호	제인 22-4998호	시행일자와 동일	근전도계 (A30010.01, 2)	모양 및 구조	작용원리	기인증사항과 동일	USB Cable 삭제
					외형		외관사진 본체 및 패치 변경, USB Cable 삭제, 외관설명 본체 설명 변경(본체, 본체연결부, USB Connector, 패치연결부, LED표시등), 패치 설명 변경(패치, 패치커버), 어플리케이션 화면 설명(신호보기: EMG Filter 주파수 범위 변경)
					치수		본체 및 패치 변경, USB Cable 삭제
					특성		작동계통도 변경, 작동계통도 설명(본체 및 패치: 표시부, 센서부 변경, 본체 및 패치: 결합부 추가, 본체(패치) 및 모바일 앱: 패치 및 본체 변경), 전기회로도 변경(전원부(Power on circuit), 통신부(Antenna interface))
				원재료			제어부의 Main PCB 변경, 전원부의 USB Cable 삭제, 장착부(패치)의 전극 수량 변경, 연결부 추가
				성 능			Sampling Rate의 '최대'삭제,

						독립전원 구동시간 변경(20시간 유지->24시간 유지), LED 표시상태의 전원 ON/OFF 모드 추가, Input Referred Noise Level의 단위 변경, Dynamic range의 'TYP'삭제, Input impedance 변경(3MΩ 이상->5M(Ω) 이상), EMG Filter(Hz) 변경(20Hz~240Hz->20Hz~450 Hz), Sensitivity의 '최소 값'추가
				사용방법		사용 전 준비사항, 사용 후 보관 및 관리방법의 USB Cable 삭제, 사용방법 중 본체와 패치 연결, LED 표시 및 충전 사용 변경, 앱 사용방법의 EMG Filter 주파수 범위 변경
				사용시 주의사항		적용상의 주의 삭제
				시험규격		시험기준 및 시험방법 변경, 독립전원 구동 시간 변경, LED 표시상태 추가, Input impedance 변경, EMG Filter(Hz) 변경

4. 아울러, 동 민원처리에 대하여 이의가 있을 때에는 「민원 처리에 관한 법률」 제35조제1항 및 같은 법 시행령 제40조제1항에 따라 [붙임2]의 '거부처분 이의신청서'를 작성하여 우리 원에 이의신청을 할 수 있음을 알려드립니다.

붙임 1. 의료기기 제조 인증서 1부(온라인수령).
2. 거부처분 이의신청서 1부. 끝.

한 국 의 료 기 기 안 전 정 보 원 장

연구원 **박혜진** 팀장 **정아름** 전결 05.02 본부장 **안만호**

시행 인증본부-MFDS-12899 (2023.05.02) 접수 20230066802 (2023.04.25)

우 08389 서울특별시 구로구 디지털로30길 28 마리오타워 303-1호 / http://www.nids.or.kr
전화 02-860-4462 전송 02-860-4479 / phj509@nids.or.kr / 비공개

■ 민원 처리에 관한 법률 시행규칙 [별지 제11호서식]
■ 수수료: 없음

거부처분 이의신청서

제출서류: 없음

접수번호:	접수일:	처리기간: 10일

1. 신청인 정보

**신청인**	이름(법인명):	연락처:
	주소(소재지):	

2. 신청 내용

**이의신청 대상 민원사항**	
**거부처분을 받은 날**	
**거부처분의 내용**	
**이의신청의 취지 및 이유**	

3. 서명 및 날인

「민원 처리에 관한 법률」 제35조제1항 및 같은 법 시행령 제40조제1항에 따라 귀 기관의 거부처분에 대하여 위와 같이 이의를 신청합니다.

년 월 일

신청인 (서명 또는 인)

접수기관 귀하

처리절차

이의신청	→	접수	→	검토 확인	→	결과통지
신청인		접수기관		접수기관		접수기관

210mm×297mm[백상지(80g/㎡) 또는 중질지(80g/㎡)]

[별첨 17] 식약처 임상시험계획 변경보고서

[별지 제1호서식]

의료기기 임상시험계획 변경보고서

보고인 (대표자)	성명 : 김현정	생년월일: 1967년 9월 2일
	주소	
제조(수입) 업소	명칭(상호) : ㈜에스엠디솔루션	업허가번호
	소재지 : 서울특별시 종로구 대학로 101 서울대학교 치의학대학원 생체재료동 107-1호 (연건동)	
임상시험계획 제목 및 승인번호	아급성기 뇌졸중 환자에서 근전도 센서 피드백에 기반한 침상내 자가운동의 기능적 이동성에 대한 효과 : 전향적, 다기관, 단일눈가림, 개방형, 탐색적 예비임상시험 (승인번호: 제 1445호)	
제품명 (품목명 및 모델명)	Myoverse s/w (근전도분석소프트웨어,Myoverse s/w)	
품목 분류번호 (등급)	E10010.01(2)	

변경대비표	변경항목	승인받은 사항	변경한 사항	비고
	사용방법	근전도계 (제인 22-4998) 허가번호만 기재	근전도계 사용방법 추가 (별첨1)	IRB 기관요청

「의료기기법」 제10조제7항 및 같은 법 시행규칙 제26조제5항에 따라 위와 같이 의료기기 임상시험계획의 변경사항을 보고합니다.

2023 년 5 월 17 일

보고인 : ㈜에스엠디솔루션 대표이사 김현정 (인)

담당자 성명 : 정지영

담당자 전화번호 : 010-2707-4317

식품의약품안전처장 귀하

210mm×297mm[일반용지 60g/㎡(재활용품)]

별첨 1

① 의료기기 사용방법

A. 제품 착용 방법

i. 필름제거

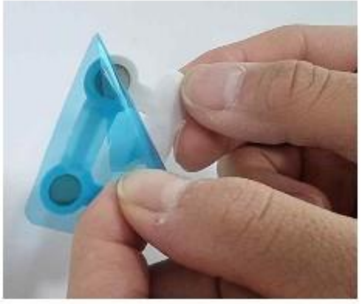

그림 27 패치 필름제거

ii. 부착

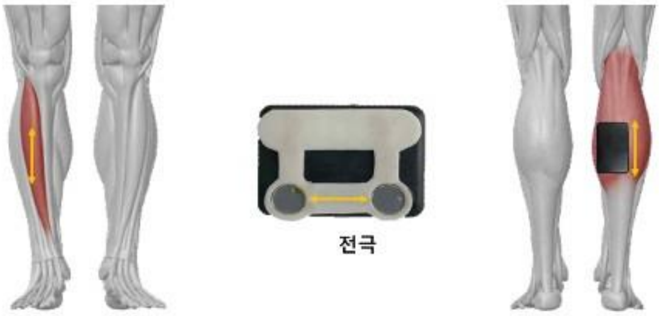

그림 28 부착 방법

패치의 두 전극을 가리키는 노란색 화살표 방향과 부착하고자 하는 근육의 노란색 화살표 방향이 평행하도록 부착함.

B. 제품 조작법

i. 결합

스냅버튼 형태의 패치(②)를 본체(①)와 결합한다.

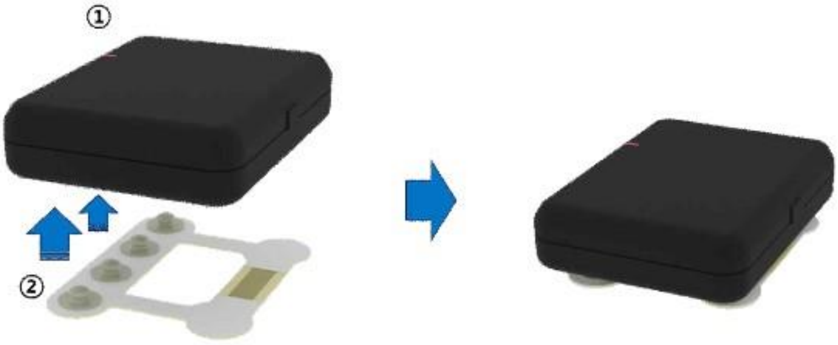

그림 29 결합 방법

ii. 전원

본체의 전원 스위치를 1초 이상 눌러 전원을 켜면 광고모드가 되어 파란색 LED가 점멸된다.

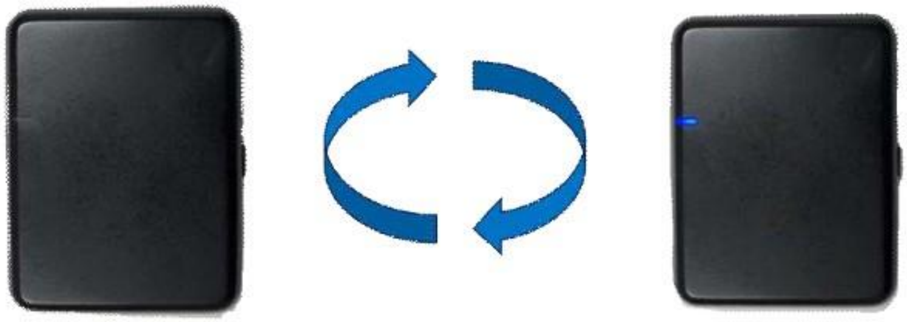

그림 30 전원 조작방법

iii. 충전

- 본체 USB Connector에 USB Cable을 연결하여 충전한다. (DC 3.7V / 450mAh)
- 충전이 진행되면 보라색 불이 들어온다.
- 충전이 완료되면 초록색 불이 들어온다.
- USB Cable을 분리한다.

충전 중에는 신호 측정이 불가능하다.

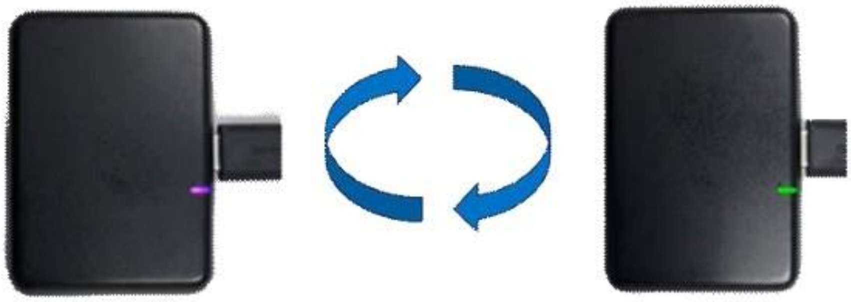

그림 31 충전 방법

② 근전도 기기의 사용 후 보관 및 관리방법

A. 본체에 묻는 오염물질을 잘 닦은 후 보관.

B. USB cable을 이용하여 충전.

③ 병용요법(adjunctive therapy)

A. 자가 운동은 표준 하지재활에 병용요법으로서 적용됨
